# Supplementary material for: Synthesis and Biological Evaluation of Oleanolic Acid Derivatives as Selective Vascular Endothelial Growth Factor Promoter i-Motif Ligands
Source: Int J Mol Sci. 2021 Feb 8;22(4):1711. doi: 10.3390/ijms22041711 (PMC7915724; doi:10.3390/ijms22041711)
Supplement: Supplementary file 1 [file ijms-22-01711-s001.pdf]

Supporting Information

Synthesis and biological evaluation of oleanolic acid derivatives as selective vascular endothelial growth factor promoter i-motif ligands

Huang Zeng [1,†], Shuangshuang Kang [1,†], Yu Zhang [1], Ke Liu [1], Qian Yu [1], Ding Li [1,*] and Lin-Kun An [1, 2, *]

[1] School of Pharmaceutical Sciences, Sun Yat-sen University, Guangzhou 510006, China; zenghuang8006@163.com (H. Zeng); kangshsh@163.com (S. Kang); zhangy553@mail2.sysu.edu.cn (Y. Zhang); ke.liu.19@ucl.ac.uk (K. Liu); yuxi024@163.com (Q. Yu); liding@mail.sysu.edu.cn (D. Li); lssalk@mail.sysu.edu.cn (L.-K. An) [2]

[2] Guangdong Provincial Key Laboratory of New Drug Design and Evaluation, Guangzhou, 510006, China; lssalk@mail.sysu.edu.cn (L.-K. An)

† The first two authors made equal contribution to this work.

* Correspondence: liding@mail.sysu.edu.cn (D. Li); lssalk@mail.sysu.edu.cn (L.-K. An); Tel.: +86-20 3994 3058 (D. Li); +86-20 3994 3413 (L.-K. An)

Table of contents

Scheme S1 3

Scheme S2 3

Figure S1 4

Figure S2 4

Figure S3 5

Figure S4 5

Figure S5 6

Figure S6 6

Figure S7 7

Figure S8 7

Figure S9 7

Figure S10 8

Figure S11 8

Figure S12 8

Table S1. Oligomers, primers or inserted clones used in this study 9

Synthesis and characterization 12

^{1}H-NMR, ^{13}C-NMR, HRMS spectra of Synthetic Compounds 44

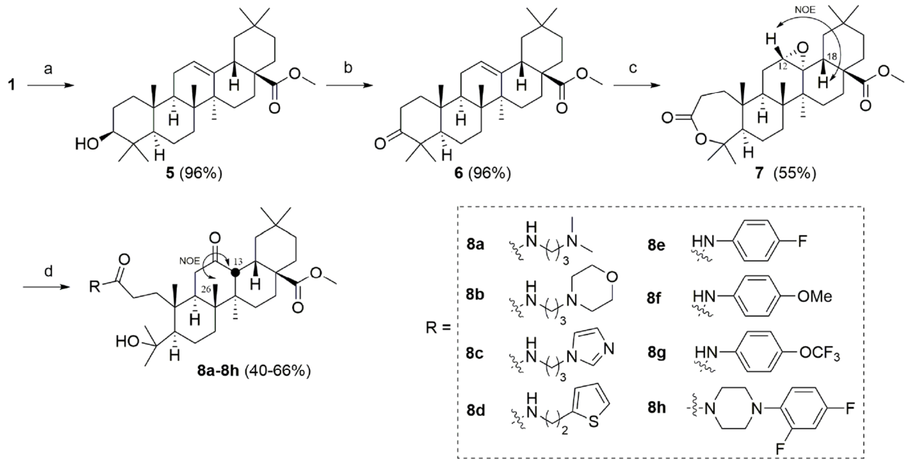

Scheme S1. Synthetic route of type II compounds **8a**–**8h**. Reagents and conditions: (a) CH_3I, DMF, K_2CO_3, r.t., 12 h (yield 96%). (b) Jones reagent, acetone, 0℃, 1h (yield 96%).(c) *m*-CPBA, $NaHCO_3$, DCM, r.t., 12 h (yield 55%). (d) amines, $AlCl_3$, Et_3N, DCM, 0 ℃, 1 h (yield 40–66%).

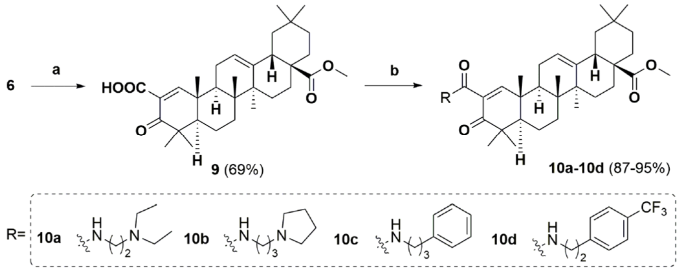

Scheme S2. Synthetic route of type III compounds **10a**–**10d**. Reagents and condition: (a) 1) Stiles's reagent, 110 ℃, 1 h; 2) DDQ, toluene, r.t., 0.5 h (yield 69%). (b) HATU, DIPEA, DMF, r.t., 1 h (yield 87–95%).

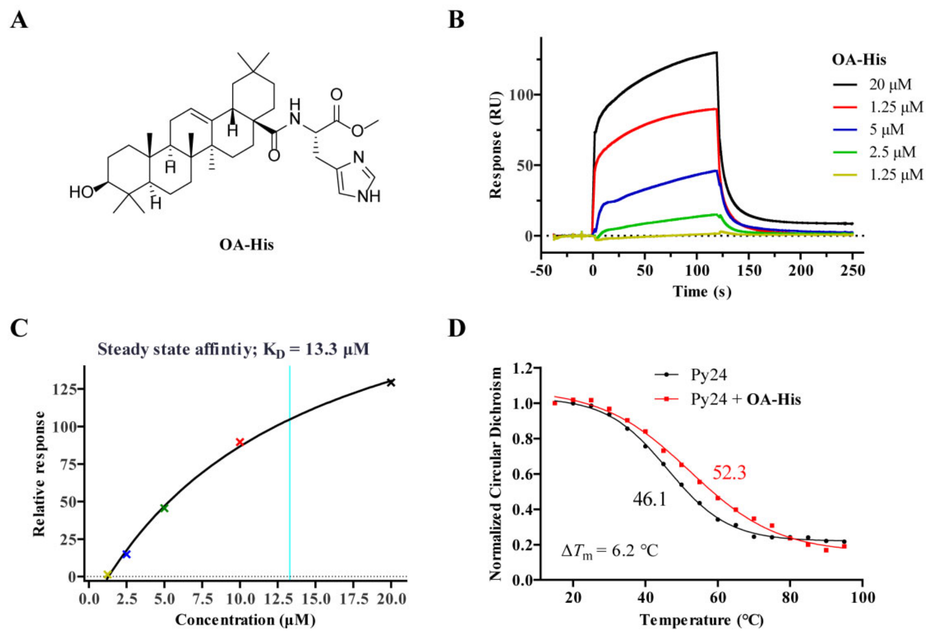

Figure S1. (**A**) The chemical structure of compound **OA-His**. (**B**, **C**) Sensorgrams (**B**) and the corresponding curve fittings (**C**) for the binding of **OA-His** to Py24 detected by using SPR assay in MES buffer at pH 5.5. (**D**) The molar ellipticity of CD melting spectra for Py24 without and with addition of 5.0 equivalent of **OA-His** in 1×BPES under pH 5.5 condition.

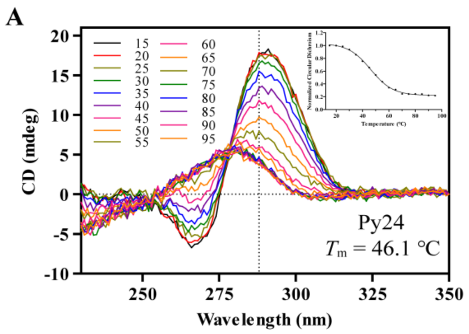

Figure S2. The CD melting spectrum for Py24 in the absence (**A**) and presence (**B**) of 5 molar equivalents of **3c** in 1× BPES buffer at pH 5.5. The spectrum was recorded with an interval of 5 °C. The insets showed melting curves and T_m values were calculated from the CD signals at 288 nm.

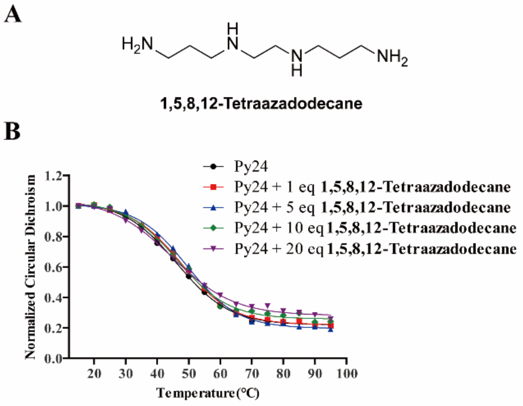

ΔT_m (°C)	1 eq	5 eq	10 eq	20 eq
**1,5,8,12-Tetraazadodecane**	1.62	2.37	0.36	-1.43

Figure S3. (**A**) The chemical structure of **1,5,8,12-tetraazadodecane**. (**B**) The molar ellipticity of CD melting spectra for Py24 without and with addition of 1, 5, 10, 20 equivalents of **1,5,8,12-Tetraazadodecane** respectively in 1×BPES at pH 5.5.

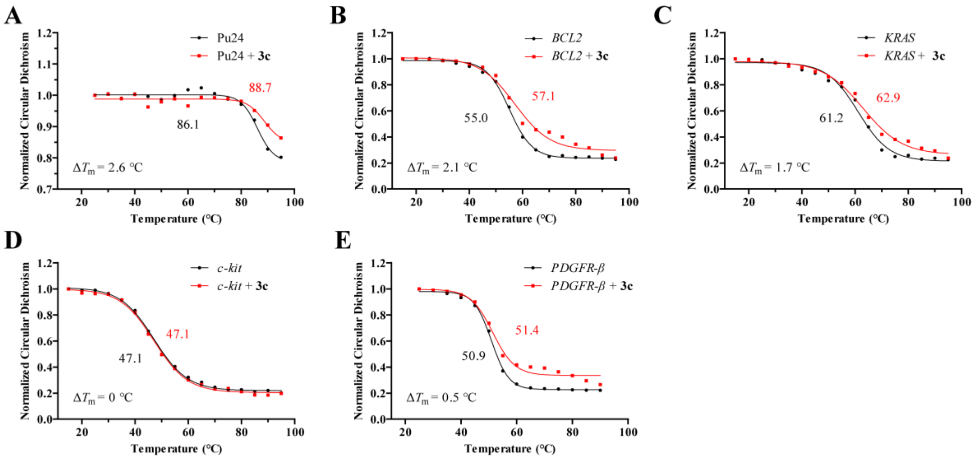

Figure S4. The molar ellipticity of CD melting spectra for oligomers without and with addition of 5 equivalents of **3c**. Data was recorded at 266 nm for Pu24 (**A**)**.** Data was recorded at 288 nm for *BCL2* i-motif (**B**)*, KRAS* i-motif (**C**), *c-kit* i-motif (**D**) and *PDGFR-β* i-motif (**E**).

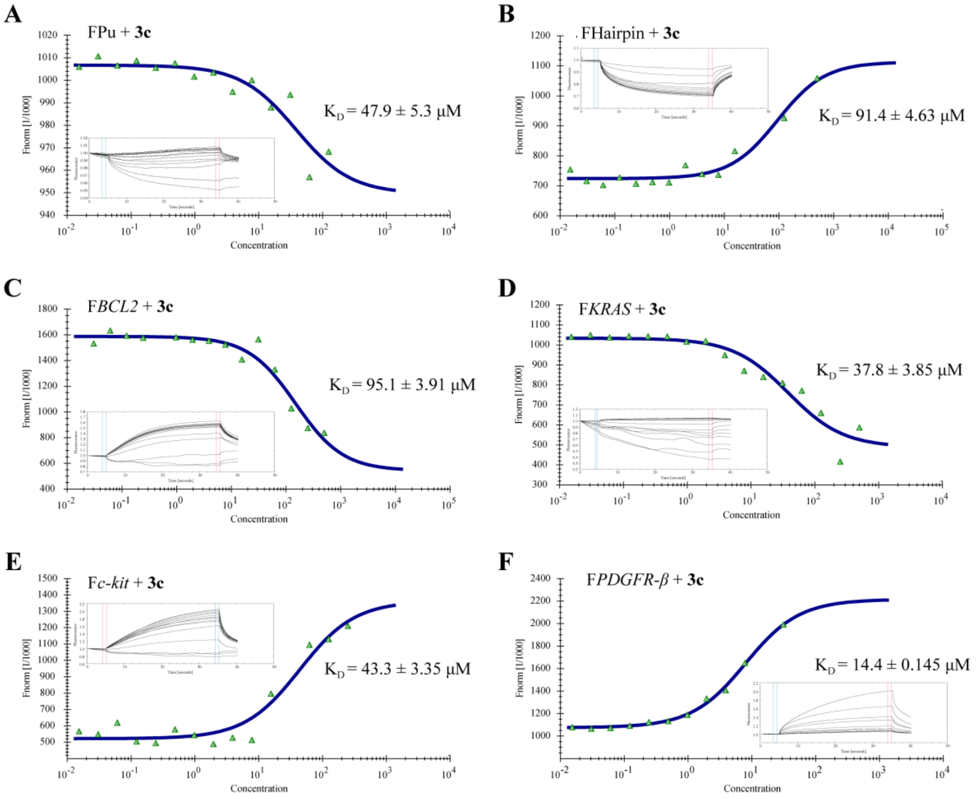

Figure S5. The K_D values were determined to be 47.9 μM, 91.4 μM, 95.1 μM, and 37.8 μM, 43.3 μM, 14.4 μM, respectively through MST for interactions of FPu24 + **3c** (**A**), FHairpin + **3c** (**B**), F*BCL2* + **3c** (**C**), F*KRAS* + **3c** (**D**), F*c-kit* + **3c** (**E**), F*PDGFR-β* + **3c** (**F**).

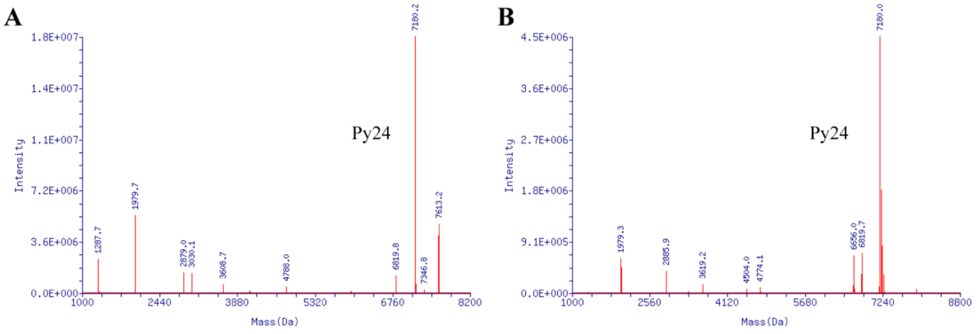

Figure S6. ESI-MS spectra of Py24 without (**A**) and with (**B**) addition of **3c** at pH 7.5.

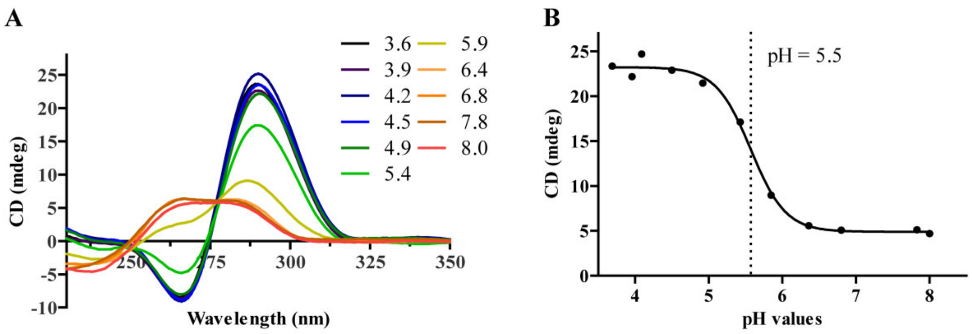

Figure S7. (**A**) CD spectra of Py24 at various pH values. (**B**) The molar ellipticity of CD spectra for Py24 versus pH values at 288 nm, which was used to determine the transitional pH.

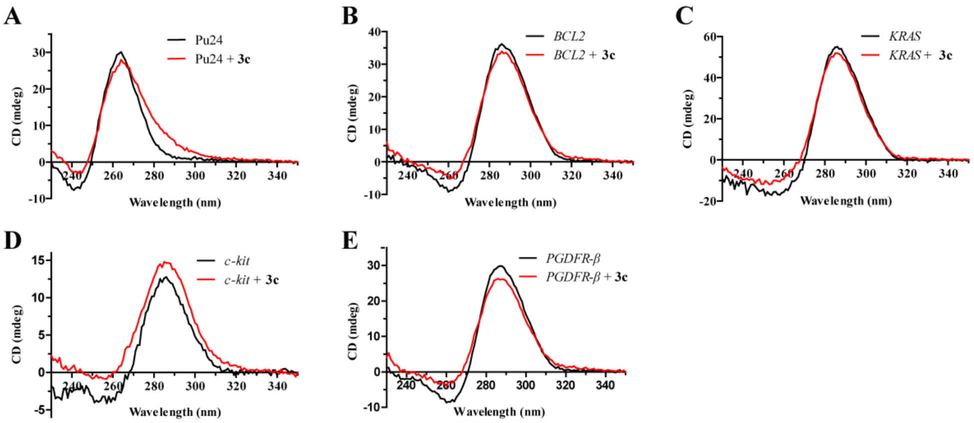

Figure S8. CD titration experiments of *VEGF* G-quadruplex and various i-motifs in the absence and presence of **3c**. (**A**) *VEGF* G-quadruplex (Pu24); (**B**) *BCL2* i-motif; (**C**) *KRAS* i-motif; (**D**) *c-kit* i-motif; (**E**) *PDGFR-β* i-motif.

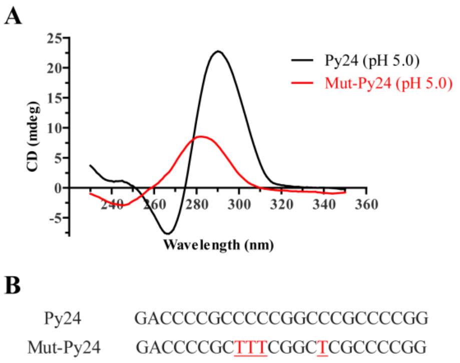

Figure S9. Comparison of *VEGF* i-motif-forming (Py24) and Mutated *VEGF* sequences (Mut-Py24). (**A**) CD spectra of Py24 and Mut-Py24 sequence at pH 5.0. (**B**) Sequences of Py24 and Mut-Py24.

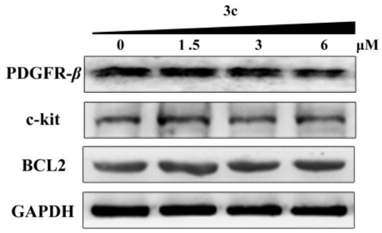

Figure S10. Effects of **3c** on protein expressions of *BCL2, c-kit*, *PDGFR-β* in MCF-7 cells in the presence or absence of **3c**.

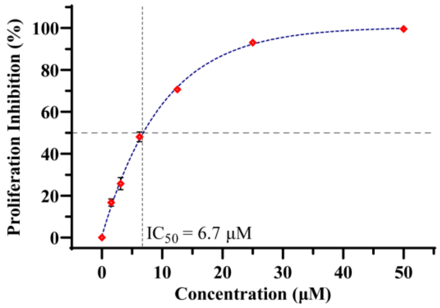

Figure S11. The cell growth inhibition curves of MCF-7 cells after a 24 h treatment with **3c** at different concentrations.

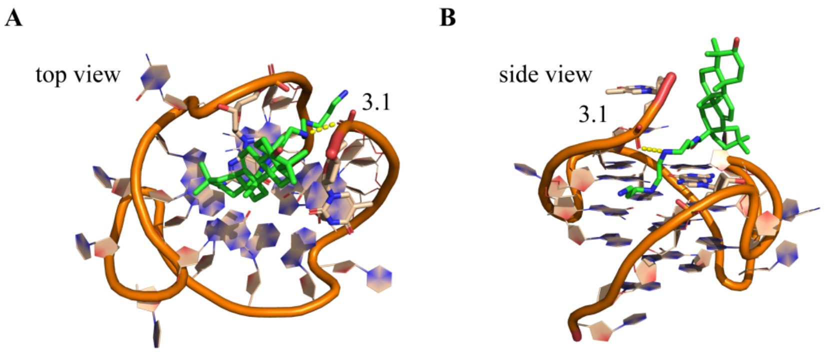

Figure S12. Hypothetical binding mode of **3c** to *VEGF* G-quadruplex structures. Top views (**A**) and side-views (**B**) of the interactions of **3c** with *VEGF* G-quadruplex (PDB ID: 2M27).

Table S1. Oligomers, primers or inserted clones used in this study

**Oligomer**	**Sequence (from 5′ to 3′)**
Py24	5′-GACCCCGCCCCCGGCCCGCCCCGG-3′
Pu24	5′-CCGGGGCGGGCCGGGGGCGGGGTC-3′
PDGFR-β	5′-GCGTCCACCCTCCCTGCCCCGCCGCCCCCCCTTCTCCCAGC-3′
BCL2	5′-CAGCCCCGCTCCCGCCCCCTTCCTCCCGCGCCCGCCCCT-3′
KRAS	5′-GCCCGGCCCCCGCTCCTCCCCCGCCGGCCCGGCCCGGCCCCCTCCTTCTCCCCG -3′
c-kit	5′-CCCCTCCCTCGCGCCCGCCCG-3′
Mut-Py24	5′-GACCCCGCTTTCGGCTCGCCCCGG-3′
FPy24	5′-FAM-GACCCCGCCCCCGGCCCGCCCCGG-3′
FPu24	5′-FAM-CCGGGGCGGGCCGGGGGCGGGGTC-3′
FHairpin	5’-FAM-TATAGCTATA-HEG-TATAGCTATA-3’
F*BCL2*	5′-FAM-CAGCCCCGCTCCCGCCCCCTTCCTCCCGCGCCCGCCCCT-3′
F*KRAS*	5′-FAM-GCCCGGCCCCCGCTCCTCCCCCGCCGGCCCGGCCCGGCCCCCTCCTTCTC CCCG -3′
F*c-kit*	5′-FAM-CCCCTCCCTCGCGCCCGCCCG-3′
F*PDGFR-β*	5′-FAM-GCGTCCACCCTCCCTGCCCCGCCGCCCCCCCTTCTCCCAGC-3′
biotin- Py24	5’-biotin-GACCCCGCCCCCGGCCCGCCCCGG- 3’
biotin- Pu24	5’-biotin-CCGGGGCGGGCCGGGGGCGGGGTC - 3’
biotin-Hairpin	5’-biotin- TATAGCTATA-HEG-TATAGCTATA- 3’
FPy24T	5′-FAM-GACCCCGCCCCCGGCCCGCCCCGG-TAMRA-3′

Primer	Sequence **(from 5′ to 3′)**
VEGF - forward primer	CTTCACCACTTCGTGATGATT
VEGF - reverse primer	CTGCTCTACCTCCACCAT
GAPDH - forward primer	GCTGTAGCCAAATTCGTTGTC
GAPDH - reverse primer	GATGACATCAAGAAGGTGGTG
BCL2 - forward primer	TGTTGTTCAAACGGGATTCA
BCL2 - reverse primer	CTCAGCCCATCTTCTTCCAG
KRAS - forward primer	GGTTGCGCTGACCTAGGAAT
KRAS - reverse primer	TCCATTTCGGGGCAAACAGT
c-kit - forward primer	TATACAACCCTGGCATTATGT
c-kit - reverse primer	TGCGAAGGAGGCTAAACCTA
PDGFR-β - forward primer	AGGACAACCGTACCTTGGGTGACT

PDGFR-β - reverse primer	CAGTTCTGACACGTACCGGGTCTC

Inserted clones	Sequences for reporter luciferase constructs (from 5′ to 3′) [a]
Wild-type VEGF [1] (582bp)	**CTCGAG**GAGCGAGCAGCGTCTTCGAGAGTGAGGACGTGTG<br>TGTCTGTGTGGGTGAGTGAGTGTGTGCGTGTGGGGTTGAGGGCG<br>TTGGAGCGGGGAGAAGGCCAGGGGTCACTCCAGGATTCCAATA<br>GATCTGTGTGTCCCTCTCCCCACCCGTCCCTGTCCGGCTCTCCGC<br>CTTCCCCTGCCCCCTTCAATATTCCTAGCAAAGAGGGAACGGCT<br>CTCAGGCCCTGTCCGCACGTAACCTCACTTTCCTGCTCCCTCCTC<br>GCCAATGCCCCGCGGGCGCGTGTCTCTGGACAGAGTTTCCGGGG<br>GCGGATGGGTAATTTTCAGGCTGTGAACCTTGGTGGGGGTCGAG<br>CTTCCCCTTCATTGCGGCGGGCTGCGGGCCAGGCTTCACTGAGC<br>GTCCGCAGAGCCCGGGCCCGAGCCGCGTGTGGAAGGGCTGAGG<br>CTCGCCTGTCCCCGCCCCCC**GGGGCGGGCCGGGGGCGGGGTC**<br>**CCGGCGGGGCGGAG**CCATGCGCCCCCCCCTTTTTTTTTAAAA<br>GTCGGCTGGTAGCGGGGAGGATCGCGGAGGCTTGGGGCAGCCG<br>GGTAGCTCGGAGGTCGTGGCGCTG**AAGCTT**
Devoid VEGF [1] (546bp)	**CTCGAG**GAGCGAGCAGCGTCTTCGAGAGTGAGGACGTGTG<br>TGTCTGTGTGGGTGAGTGAGTGTGTGCGTGTGGGGTTGAGGGCG<br>TTGGAGCGGGGAGAAGGCCAGGGGTCACTCCAGGATTCCAATA<br>GATCTGTGTGTCCCTCTCCCCACCCGTCCCTGTCCGGCTCTCCGC<br>CTTCCCCTGCCCCCTTCAATATTCCTAGCAAAGAGGGAACGGCT<br>CTCAGGCCCTGTCCGCACGTAACCTCACTTTCCTGCTCCCTCCTC<br>GCCAATGCCCCGCGGGCGCGTGTCTCTGGACAGAGTTTCCGGGG<br>GCGGATGGGTAATTTTCAGGCTGTGAACCTTGGTGGGGGTCGAG<br>CTTCCCCTTCATTGCGGCGGGCTGCGGGCCAGGCTTCACTGAGC<br>GTCCGCAGAGCCCGGGCCCGAGCCGCGTGTGGAAGGGCTGAGG<br>CTCGCCTGTCCCCGCCCCCCCCATGCGCCCCCCCCTTTTTTTTTA<br>AAAGTCGGCTGGTAGCGGGGAGGATCGCGGAGGCTTGGGGCAG<br>CCGGGTAGCTCGGAGGTCGTGGCGCTG**AAGCTT**
Mutant VEGF (582bp)	**CTCGAG**GAGCGAGCAGCGTCTTCGAGAGTGAGGACGTGTG<br>TGTCTGTGTGGGTGAGTGAGTGTGTGCGTGTGGGGTTGAGGGCG<br>TTGGAGCGGGGAGAAGGCCAGGGGTCACTCCAGGATTCCAATA<br>GATCTGTGTGTCCCTCTCCCCACCCGTCCCTGTCCGGCTCTCCGC<br>CTTCCCCTGCCCCCTTCAATATTCCTAGCAAAGAGGGAACGGCT

	CTCAGGCCCTGTCCGCACGTAACCTCACTTTCCTGCTCCCTCCTC<br>GCCAATGCCCCGCGGGCGCGTGTCTCTGGACAGAGTTTCCGGGG<br>GCGGATGGGTAATTTTCAGGCTGTGAACCTTGGTGGGGGTCGAG<br>CTTCCCCTTCATTGCGGCGGGCTGCGGGCCAGGCTTCACTGAGC<br>GTCCGCAGAGCCCGGGCCCGAGCCGCGTGTGGAAGGGCTGAGG<br>CTCGCCTGTCCCCGCCCCCCGGGGCGAGCCGAAAGCGGGGTCCC<br>GGCGGGGCGGAGCCATGCGCCCCCCCCTTTTTTTTTAAAAGTC<br>GGCTGGTAGCGGGGAGGATCGCGGAGGCTTGGGGCAGCCGGGT<br>AGCTCGGAGGTCGTGGCGCTG**AAGCTT**

[a] Sequence cloned into pGL4.10 vector (The grey highlighted sequence is the complementary strand of i-motif-forming sequence in *VEGF* promoter. The underlined base among grey highlighted sequence is mutant base. Bold and underlined sequence with six-nucleotide corresponds to the XhoI and HindIII restriction sites)

Synthesis and characterization

1. The synthesis of 1-benzotriazolyl oleanolate (**2**)

The intermediate **2** was synthesized according to the reported method [2]. White solid, m.p. 184.8–186.1 °C (lit. 184.4–185.6 °C [3]). ^{1}H NMR spectrum was similar to the reported data [3].

2. The general synthetic procedure of **3a-3b** and **3d-3q**.

According to the reported method [3], intermediate **2** reacted with the corresponding alkyl amines to give the target products.

2.1. N-propargyl oleanolamide (**3a).**

Yellow solid, yield 93%. m.p. 201.0–202.9 °C (lit. 201.3–202.7 °C [3]). ESI-MS (*m/z*) 494 [M + H]$^+$. ^{1}H NMR spectrum was similar to the reported data [3].

2.2. N-(6-hydroxyhexyl) oleanolamide (**3b).**

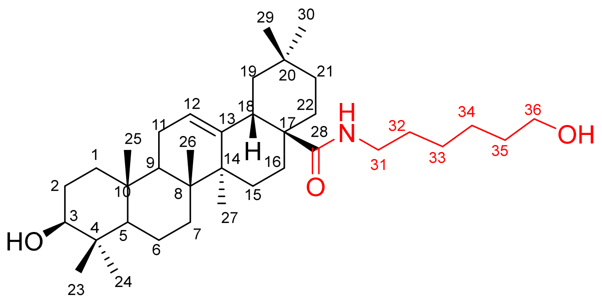

White solid, yield 88%. m.p. 183.3–184.6 °C (lit. 183.8–184.5 °C [4]). ^{1}H NMR spectrum was similar to the reported data [4].

2.3. N-(2-(1H-imidazol-4-yl)ethyl) oleanolamide (**3d**).

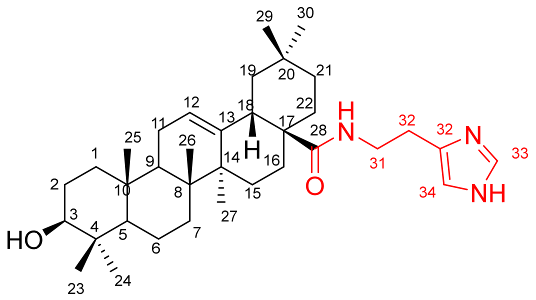

White solid, yield 89%. m.p. 170.6–172.2 °C. IR (KBr): ν = 3356, 2924, 1637, 1463, 754 cm^{-1}. ^{1}H NMR (CDCl3) δ 7.56 (s, 1H, H-33), 6.80 (s, 1H, H-34), 6.48 (br s, 1H, NH), 5.29 (dd, J = 3.1, 3.1 Hz, 1H, H-12), 3.70 – 3.64 (m, 1H, H-31a), 3.29 (dd, J = 10.9, 4.5 Hz, 1H, H-3), 3.35 – 3.26 (m, 1H, H-31b), 2.80 – 2.75 (m, 2H, H-32), 2.01-1.15 (m, 20H, other aliphatic ring protons), 1.14 (s, 3H, CH_3), 1.08 – 1.02 (m, 1H, H-15b), 0.98 (s, 3H, CH_3), 0.96 – 0.90 (m, 1H, H-1b), 0.90 (s, 3H, CH_3), 0.88 (s, 6H, 2×CH_3), 0.78 (s, 3H, CH_3), 0.73 – 0.68 (m, 1H, H-5), 0.67 (s, 3H, CH_3). ^{13}C NMR (CDCl3) δ 178.67 (C-28), 144.43 (C-13), 134.77 (C-33), 122.99 (C-12), 78.95 (C-3), 55.10 (C-5), 47.55 (C-9), 46.77 (C-17), 46.30 (C-19), 42.03 (C-18), 41.94 (C-14), 39.33 (C-8), 39.11 (C-31), 38.77 (C-4), 38.47 (C-1), 36.95 (C-10), 34.14 (C-21), 32.99 (C-7), 32.57 (C-29), 32.33 (C-22), 30.72 (C-20), 28.10 (C-23), 27.28 (C-15), 27.13 (C-2), 26.80 (C-32), 25.77 (C-27), 23.76 (C-16), 23.56 (C-11), 23.50 (C-30), 18.28 (C-6), 16.83 (C-24), 15.61 (C-25), 15.37 (C-26). HRMS (ESI; *m/z*). Calcd for $C_{35}H_{56}N_3O_2$, $[M + H]^+$, 550.4367; found, 550.4379.

2.4. N-(2-(morpholine-4-sulfonamido)ethyl) oleanolamide (**3e**).

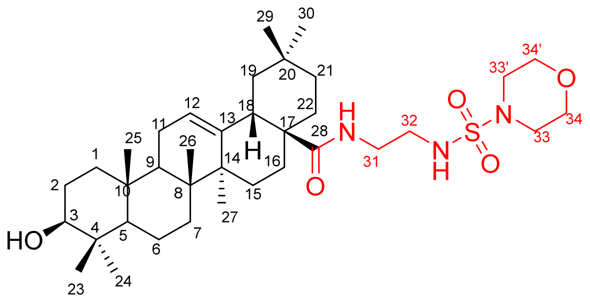

White solid, yield 71%; m.p. 126.9–128.1 °C. IR (KBr): ν = 3403, 2925, 1634, 1153, 940, 727, 525 cm^{-1}. ^{1}H NMR ($CDCl_3$) δ 6.35 (t, J = 6.0 Hz, 1H, NH), 5.41 (t, J = 4.0 Hz, 1H, H-12), 5.37 (t, J = 6.0 Hz, 1H, NH), 3.78 – 3.70 (m, 4H, H-34, H-34’), 3.29 (dd, J = 10.9, 4.5 Hz, 1H, H-3), 3.33 – 3.07 (m, 8H, H-31, H-32, H-33, H-33’), 2.06 – 1.17 (m, 22H, other aliphatic ring protons), 1.17 (s, 3H, CH_3), 1.08 – 1.02 (m, 1H, H-15b), 0.99 (s, 3H, CH_3), 0.91

(s, 9H, 3×CH_3), 0.78 (s, 3H, CH_3), 0.76 (s, 3H, CH_3); ^{13}C NMR ($CDCl_3$) δ 180.15 (C-28), 144.61 (C-13), 123.19, (C-12) 78.93 (C-3), 66.20 (C-34,C-34'), 55.08 (C-5), 47.51 (C-9), 46.74 (C-17), 46.42 (C-33, C-33'), 46.21 (C-19), 44.27 (C-32), 44.22 (C-18), 42.00 (C-14), 39.83 (C-31), 39.38 (C-8), 38.77 (C-4), 38.44 (C-1), 36.97 (C-10), 34.10 (C-21), 32.98 (C-7), 32.53 (C-29), 32.26 (C-22), 30.73 (C-20), 28.09 (C-23), 27.27 (C-15), 27.13 (C-2), 25.80 (C-27), 23.79 (C-16), 23.56 (C-11), 23.49 (C-30), 18.28 (C-6), 16.94 (C-24), 15.58 (C-25), 15.36 (C-26). HRMS (ESI; *m/z*). Calcd for $C_{36}H_{62}N_3O_5S$, $[M + H]^+$, 648.4405; found, 648.4401.

2.5. N-(2-(2,4-dimethoxybenzamido)ethyl) oleanolamide (**3f**).

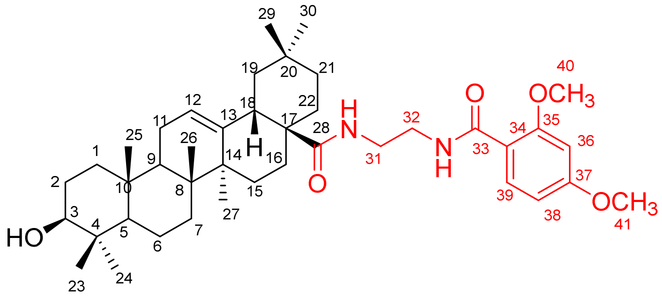

White solid, yield 90%; m.p. 144.2–146.1 °C. IR (KBr): v = 3395, 2926, 1631, 1604, 1521, 1264, 1026 cm^{-1}. ^{1}H NMR ($CDCl_3$) δ 8.19 (d, *J* = 8.8 Hz, 1H, H-39), 8.10 (t, *J* = 5.4 Hz, 1H, NH), 6.61 (t, *J* = 5.4 Hz, 1H, NH), 6.59 (dd, *J* = 8.8, 2.3 Hz, 1H, H-38), 6.48 (d, *J* = 2.2 Hz, 1H, H-36), 5.35 (dd, *J* = 3.1, 3.1 Hz, 1H, H-12), 3.95 (s, 3H, H-40), 3.85 (s, 3H, H-41), 3.72 – 3.48 (m, 3H, H-31, H-32a), 3.28 – 3.16 (m, 2H, H-3 H-32b), 2.60 (d, *J* = 16.0 Hz, 1H, H-18), 1.99 – 1.15 (m, 21H, other aliphatic ring protons), 1.11 (s, 3H, CH_3), 0.97 (s, 3H, CH_3), 0.94 – 0.92 (m, 1H, H-15b), 0.88 (s, 3H, CH_3), 0.88 (s, 3H, CH_3), 0.81 (s, 3H, CH_3), 0.76 (s, 3H, CH_3), 0.70 – 0.69 (m, 1H, H-5), 0.67 (s, 3H, CH_3). ^{13}C NMR ($CDCl_3$) δ 178.80 (C-28), 166.30 (C-33), 163.57 (C-37), 159.02 (C-35), 144.27 (C-12), 134.06 (C-34), 122.95 (C-12), 113.92 (C-39), 105.27 (C-38), 98.45 (C-36), 78.96 (C-3), 55.87 (C-40), 55.54 (C-41), 55.07 (C-5), 47.54 (C-9), 46.70 (C-17), 46.18 (C-19), 41.81 (C-18), 41.64 (C-14), 40.77 (C-31), 39.27 (C-8), 39.00 (C-32), 38.73 (C-4), 38.40 (C-1), 36.92 (C-10), 34.16 (C-21), 33.05 (C-7), 32.78 (C-29), 32.24 (C-22), 30.73 (C-20), 28.08 (C-23), 27.36 (C-15), 27.15 (C-2), 25.86 (C-27), 23.60 (C-16), 23.54 (C-11), 23.34 (C-30), 18.30 (C-6), 16.72 (C-24), 15.57 (C-25), 15.26 (C-26). HRMS (ESI; m/z). Calcd for $C_{41}H_{63}N_2O_5$, $[M + H]^+$, 663.4731; found, 663.4752.

2.6. N-(2-(2-(phenylamino)benzamido)ethyl) oleanolamide (**3g**).

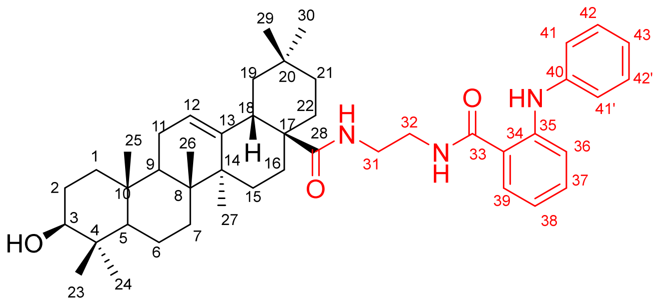

White solid, yield 86%. m.p. 130.7–131.8 °C. IR (KBr): ν = 3342, 2924, 1627, 1519, 1463, 748 cm^{-1}. ^{1}H NMR ($CDCl_3$) δ 7.55 (br s, 1H, NH), 7.49 (d, *J* = 7.1 Hz, 1H, H-36), 7.35 (d, *J* = 8.4 Hz, 1H, H-39), 7.32 – 7.23 (m, 4H, H-37, H-42, H-42’,NH), 7.19 (d, *J* = 7.6 Hz, 2H, H-41, H-41’), 6.99 (t, *J* = 7.3 Hz, 1H, H-43), 6.76 (t, *J* = 7.5 Hz, 1H, H-38), 6.47 (t, *J* = 5.4 Hz, 1H, NH), 5.38 (dd, *J* = 3.1, 3.1 Hz, 1H, H-12), 3.66 – 3.55 (m, 1H, H-32), 3.55 – 3.48 (m, 2H, H-31, H-31’) 3.46 – 3.32 (m, 1H, H-32’), 3.18 (dd, *J* = 10.9, 4.5 Hz, 1H, H-3), 2.60 (d, *J* = 15.0 Hz, 1H, H-18), 2.05 – 1.23 (m, 20H, other aliphatic ring protons), 1.13 (s, 3H, CH_3), 0.94 – 0.92 (m, 1H, H-15b), 0.96 (s, 3H, CH_3), 0.88 (s, 3H, CH_3), 0.85 (s, 3H, CH_3), 0.84 (s, 3H, CH_3), 0.75 (s, 3H, CH_3), 0.72 (s, 3H, CH_3). ^{13}C NMR (101 MHz, $CDCl_3$) δ 180.70 (C-28), 170.13 (C-33), 145.75 (C-35), 144.48 (C-13), 141.64 (C-40), 132.16 (C-37), 129.25 (C-42, C-42’), 127.93 (C-39), 123.17 (C-43), 122.27 (C-12), 120.74 (C-41, C-41’), 117.96 (C-33), 117.25 (C-36), 115.22 (C-38), 78.96 (C-3), 55.07 (C-5), 47.49 (C-9), 46.76 (C-17), 46.43 (C-19), 42.00 (C-18, C-32), 41.67 (C-14), 39.46 (C-31), 39.30 (C-8), 38.74 (C-4), 38.44 (C-1), 36.93 (C-10), 34.07 (C-21), 32.95 (C-7), 32.61 (C-29), 32.24 (C-22), 30.69 (C-20), 28.07 (C-23), 27.21 (C-15), 27.15 (C-2), 25.75 (C-27), 23.82 (C-16), 23.48 (C-11), 23.45 (C-30), 18.21 (C-6), 16.82 (C-24), 15.53 (C-25), 15.34 (C-26). HRMS (ESI; m/z). Calcd for $C_{45}H_{64}N_3O_3$, $[M + H]^+$,694.4942; found, 694.4936.

2.7. (E)-N-(2-(3-(3,4-dihydroxyphenyl)acrylamido)ethyl) oleanolamide (**3h**).

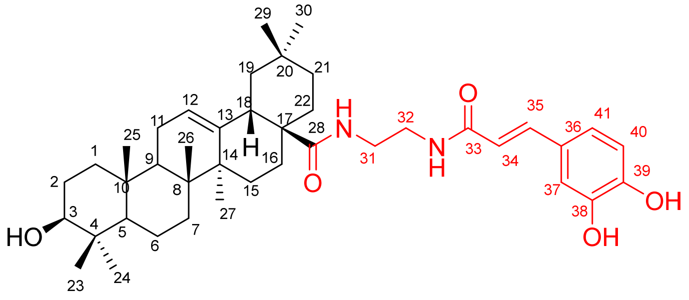

White solid, yield 71%. m.p. 180.4–181.9 °C. IR (KBr): ν = 3357, 2928, 1674, 1522, 1464, 1124, 1045 cm^{-1}. ^{1}H

NMR ($CDCl_3$) δ 7.44 (d, J = 15.6 Hz, 1H, H-35), 7.06 (s, 1H, H-37), 6.92 – 6.78 (m, 2H, H-40, H-41), 6.75 (br s, 1H, NH), 6.21 (d, J = 15.6 Hz, 1H, H-34), 5.40 (dd, J = 3.1, 3.1 Hz, 1H, H-12), 3.60 – 3.44 (m, 2H, H-31), 3.43 – 3.32 (m, 1H, H-3), 3.31 – 3.09 (m, 2H, H-32), 2.57 (d, J = 15.0 Hz, 1H, H-18), 2.19 – 1.13 (m, 24H, other aliphatic ring protons), 1.12 (s, 3H, CH_3), 0.95 (s, 3H, CH_3), 0.88 (s, 6H, 2×CH_3), 0.81 (s, 3H, CH_3), 0.74 (s, 3H, CH_3), 0.69 (s, 3H, CH_3). ^{13}C NMR (126 MHz, $CDCl_3$) δ 180.44 (C-28), 167.97 (C-33), 146.83 (C-39), 144.69 (C-13), 143.95 (C-38), 141.64 (C-35), 127.15 (C-36), 123.25 (C-12), 121.82 (C-34), 117.29 (C-41), 115.31 (C-40), 113.68 (C-37), 78.90 (C-3), 55.03 (C-5), 47.46 (C-9), 46.57 (C-17), 46.36 (C-19), 41.80 (C-18), 41.55 (C-14), 40.10 (C-30), 39.26 (C-29), 38.66 (C-4), 38.33 (C-1), 36.86 (C-10), 34.04 (C-21), 32.92 (C-7), 32.70 (C-29), 32.21 (C-22), 30.64 (C-20), 28.00 (C-23), 27.22 (C-15), 26.86 (C-2), 25.77 (C-27), 23.50 (C-16), 23.45 (C-11,C-30), 18.19 (C-6), 16.77 (C-24), 15.53 (C-25), 15.22 (C-26). HRMS (ESI; *m/z*). Calcd for $C_{41}H_{61}N_2O_5$, $[M + H]^+$, 661.4575; found, 661.4586.

2.8. N-(3-((2-((3-(2-(5-methyl-2,4-dioxo-3,4-dihydropyrimidin-1(2H)-yl)acetamido)propyl)amino)ethyl)amino) propyl) oleanolamide (**3i**)

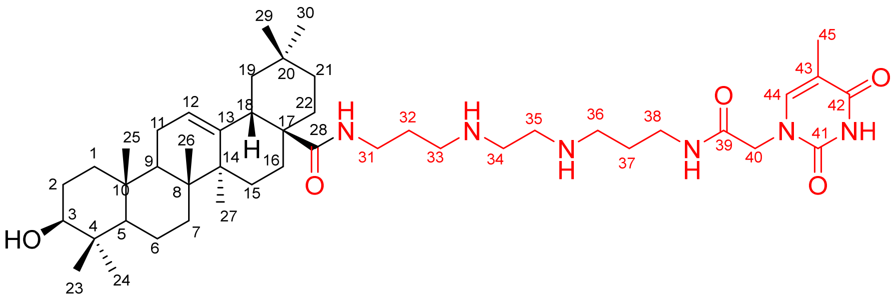

White solid, yield 80%. m.p. 127.0–128.2 ℃. IR (KBr): ν = 3395, 2926, 1675, 1521, 1464, 1242, 1027 cm^{-1}. ^1H NMR (500 MHz, $CDCl_3$) δ 7.99 (s, 1H, NH), 7.27 (s, 1H, NH), 7.11 (s, 1H, H-44), 6.31 (s, 1H, NH), 5.37 (s, 1H, H-12), 4.31 (s, 2H, H-34), 3.50 – 3.31 (m, 4H, H-32, H-38), 3.29 – 3.18 (m, 1H, H-3), 2.91 – 2.61 (m, 8H, H-33, H-34, H-35, H-36), 2.54 (d, J = 15.0 Hz, 1H, H-18), 1.90 (s, 3H, H-45), 1.81 – 1.18 (m, 28H, the protons of other aliphatic ring and chain), 1.16 (s, 3H, CH_3), 1.07 – 1.03 (m, 1H, H-15b), 0.99 (s, 3H, CH_3), 0.90 (s, 9H, 3 × CH_3), 0.78 (s, 3H, CH_3), 0.74 (s, 3H, CH_3). ^{13}C NMR (126 MHz, $CDCl_3$) δ 179.05 (C-28), 166.71 (C-39), 164.68 (C-42), 151.64 (C-41), 144.83 (C-13), 140.76 (C-44), 122.86 (C-12), 111.04 (C-43), 78.91 (C-3), 55.09 (C-5), 50.89 (C-40), 48.51 (C-34), 48.38 (C-35), 47.94 (C-36), 47.52 (C-9), 46.75 (C-17), 46.51 (C-33), 46.33 (C-19), 42.13 (C-18), 42.04 (C-14), 39.36 (C-8), 38.82 (C-31), 38.76 (C-4), 38.47 (C-1), 37.19 (C-38), 36.97 (C-10), 34.13

(C-21), 32.97 (C-7), 32.76 (C-29), 32.34 (C-22), 30.73 (C-20), 29.13 (C-32, C-37), 28.10 (C-23), 27.28 (C-15), 27.14 (C-2), 25.78 (C-27), 23.74 (C-16), 23.62 (C-11), 23.53 (C-30), 18.28 (C-6), 16.97 (C-24), 15.60 (C-25), 15.37 (C-26), 12.38 (C-45). HRMS (ESI; *m/z*). Calcd for $C_{39}H_{61}N_4O_5$, $[M + H]^+$, 665.4636; found, 665.4637. HRMS (ESI; *m/z*). Calcd for $C_{45}H_{75}N_6O_5$, $[M + H]^+$, 779.5793; found, 779.5790.

2.9. 1'-(oleanoloyl)-(1,4'-bipiperidin) (**3j**).

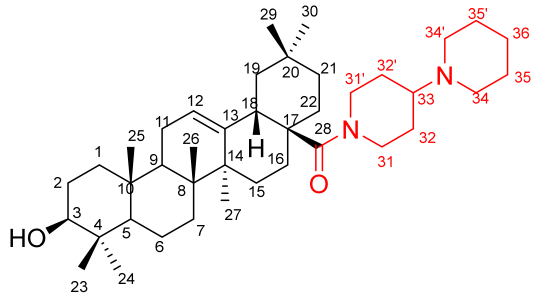

White solid, yield 91%. m.p. 220.1–222.3 °C. IR (KBr): ν = 3496, 2926, 1602, 1464, 1416, 1017 cm^{-1}. ^1H NMR ($CDCl_3$) δ 5.26 (dd, J = 3.1, 3.1 Hz, 1H, H-12), 3.21 (d, J = 7.7 Hz, 1H, H-3), 3.08 (d, J = 12.4 Hz, 1H, H-18), 2.69 (t, J = 11.9 Hz, 2H, H-31), 2.58 – 2.44 (m, 4H, H-31', H-34), 2.16 – 2.15 (m, 33H, other aliphatic ring protons), 1.13 (s, 3H, CH_3), 0.99 (s, 3H, CH_3), 0.92 (s, 3H, CH_3), 0.90 (s, 3H, CH_3), 0.89 (s, 3H, CH_3), 0.78 (s, 3H, CH_3), 0.74 (s, 3H, CH_3). ^{13}C NMR (101 MHz, $CDCl_3$) δ 174.82 (C-28), 144.96 (C-13), 121.42 (C-12), 79.06 (C-33), 62.88 (C-34, C-34'), 55.35 (C-5), 50.27 (C-34,C-34'), 47.82 (C-9), 47.40 (C-17), 46.57 (C-19), 45.47 (C-9), 44.89 (C-17), 43.59 (C-14), 41.87 (C-18), 39.12 (C-8), 38.77 (C-4), 38.43 (C-1), 37.13 (C-10), 34.11 (C-21), 33.10 (C-29), 32.93 (C-7), 30.41 (C-22), 29.91 (C-20), 28.66 (C-32, C-32'), 28.12 (C-15), 27.25 (C-23), 26.35 (C-35,C-35'), 25.96 (C-2), 24.74 (C-27), 24.12 (C-16), 23.41 (C-30), 22.86 (C-11), 18.37 (C-6), 16.98 (C-24), 15.57 (C-25), 15.33 (C-26). HRMS (ESI; *m/z*). Calcd for $C_{40}H_{67}N_2O_2$, $[M + H]^+$, 607.5197; found, 607.5183.

2.10. 1-(oleanoloyl)-(4-(pyrimidin-2-yl)piperazin) (**3k)**.

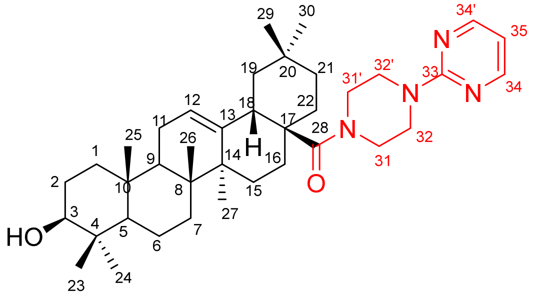

White solid, yield 81%. m.p. 140.4–141.5 °C. IR (KBr): v = 3444, 2925, 1584, 1456, 982, 728 cm^{-1}. ^1H NMR ($CDCl_3$) δ 8.33 (d, J = 15.0 Hz, 2H, H-34, H-34'), 6.54 (t, J = 15.0 Hz, 1H, H-35), 5.29 (td, J = 12.4 Hz, 1H, H-12), 3.94 – 3.63 (m, 8H, H-31, H-31', H-32, H-32'), 3.20 (dd, J = 11.0, 4.5 Hz, 1H, H-3), 3.11 (d, J = 15.0 Hz, 1H, H-18), 2.20 – 1.17 (m, 22H, other aliphatic ring protons), 1.15 (s, 3H, CH_3), 0.98 (s, 3H, CH_3), 0.94 (s, 3H, CH_3), 0.91 (s, 3H, CH_3), 0.90 (s, 3H, CH_3), 0.77 (s, 3H, CH_3), 0.74 (s, 3H, CH_3). ^{13}C NMR ($CDCl_3$) δ 175.36 (C-28), 161.68 (C-33), 157.75 (C-34,C-34'), 144.67 (C-13), 121.68 (C-12), 110.39 (C-35), 78.98 (C-3), 55.31 (C-5), 47.78 (C-32,C-32'), 47.48 (C-31,C-31'), 46.39 (C-19), 45.23 (C-9), 43.81 (C-17), 43.55 (C-14), 41.86 (C-18), 39.15 (C-8), 38.75 (C-4), 38.41 (C-1), 37.09 (C-10), 34.01 (C-21), 33.11 (C-29), 32.83 (C-7), 30.43 (C-22), 30.04 (C-20), 28.12 (C-15), 27.94 (C-23), 27.21 (C-2), 26.02 (C-27), 24.11 (C-16), 23.40 (C-30), 22.85 (C-11), 18.29 (C-6), 16.93 (C-24), 15.60 (C-25), 15.34 (C-26). HRMS (ESI; *m/z*). Calcd for $C_{38}H_{59}N_4O_2$, [M + H]$^+$, 603.4633; found, 603.4633.

2.11. 1-(oleanoloyl)-(4-(pyridin-2-yl)piperazin) (**3l**).

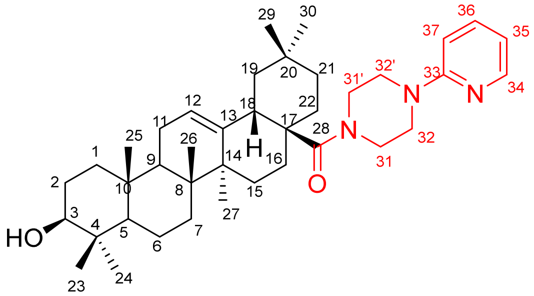

White solid, yield 84%. m.p. 153.3–156.0 °C. IR (KBr): v = 3444, 2925, 1611, 1507, 1462, 1122, 992, 727 cm^{-1}. ^1H NMR ($CDCl_3$) δ 8.20 (d, J = 3.1 Hz, 1H, H-34), 7.59 – 7.43 (m, 1H, H-36), 6.66 (dd, J = 10.2, 4.4 Hz, 2H, H-35, H-36), 5.28 (s, 1H, H-12),3.91 – 3.69 (m, 4H, H-32, H-32'), 3.65 – 3.41 (m, 4H, H-31, H-31'), 3.20 (dd, J = 15.0, 5.0 Hz, 1H, H-3), 3.11 (d, J = 20.0 Hz, 1H, H-18), 2.20 – 1.25 (m, 22H, other aliphatic ring protons), 1.14

(s, 3H, CH_3), 0.97 (s, 3H, CH_3), 0.94 (s, 3H, CH_3), 0.91 (s, 3H, CH_3), 0.89 (s, 3H, CH_3), 0.77 (s, 3H, CH_3), 0.74 (s, 3H, CH_3). ^{13}C NMR (101 MHz, $CDCl_3$) δ 175.31 (C-28), 159.33 (C-33), 147.96 (C-34), 144.71 (C-13), 137.63 (C-36), 121.64 (C-12), 113.84 (C-35), 107.28 (C-37), 79.03 (C-3), 55.30 (C-5), 47.78 (C-32,C-32'), 47.45 (C-31,C-31'), 46.39 (C-19), 45.35 (C-9), 45.12(C-17), 43.53 (C-14), 41.86 (C-18), 39.15 (C-8), 38.76 (C-4), 38.40 (C-1), 37.10 (C-10), 34.01 (C-21), 33.11 (C-29), 32.82 (C-7), 30.44 (C-22), 30.01 (C-20), 28.12 (C-15), 27.94 (C-23), 27.21 (C-2), 26.02 (C-27), 24.10 (C-16), 23.40 (C-30), 22.82 (C-11), 18.30 (C-6), 16.92 (C-24), 15.59 (C-25), 15.35 (C-26). HRMS (ESI; *m/z*). Calcd for $C_{39}H_{60}N_3O_2$, $[M + H]^+$, 602.4680; found, 602.4671.

2.12. 1-(oleanoloyl)-(4-(2-methoxyphenyl)piperazin) (**3m**).

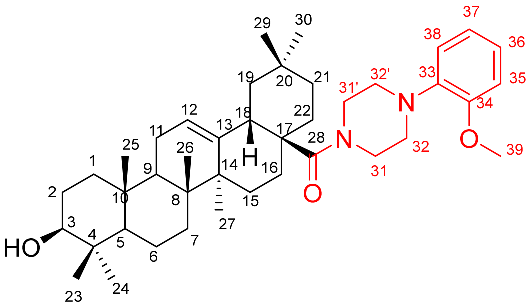

White solid, yield 87%. m.p. 143.9–145.4 °C. IR (KBr): v = 3444, 2926, 1615, 1507, 1456, 1023, 727 cm^{-1}. ^{1}H NMR ($CDCl_3$) δ 7.09 – 7.02 (m, 1H, H-37), 6.97 – 6.92 (m, 2H, H-35, H-38), 6.90 – 6.87 (m, 1H, H-36), 5.31 (dd, J = 3.1, 3.1 Hz, 1H, H-12), 3.89 (s, 3H, H-39), 3.87 – 3.79 (m, 4H, H-31, H-31'), 3.23 (dd, J = 15.0, 5.0 Hz, 1H, H-3), 3.13 (dd, J = 16.0, 4.0 Hz, 1H, H-18), 3.09 – 2.97 (m, 4H, H-32, H-32'), 2.20 – 1.29 (m, 21H, other aliphatic ring protons), 1.16 (s, 3H, CH_3), 1.00 (s, 3H, CH_3), 0.96 (s, 3H, CH_3), 0.92 (s, 6H, 2×CH_3), 0.80 (s,3H, CH_3), 0.78 (s, 3H, CH_3). ^{13}C NMR ($CDCl_3$) δ 175.07 (C-28), 152.25 (C-33), 144.82 (C-13), 140.80 (C-34), 123.36 (C-38), 121.55 (C-12), 121.04 (C-37), 118.28 (C-35), 111.34 (C-3), 79.06 (C-3), 55.42 (C-39), 55.35 (C-5), 50.94 (C-32,C-32'), 47.83 (C-31,C-31'), 47.49 (C-19), 46.46 (C-9), 45.62 (C-17), 43.62 (C-14), 41.91 (C-18), 39.17 (C-8), 38.77 (C-4), 38.43 (C-1), 37.13 (C-10), 34.05 (C-21), 33.11 (C-29), 32.90 (C-7), 30.43 (C-22), 30.07 (C-20), 28.12 (C-15), 27.99 (C-23), 27.24 (C-2), 26.02 (C-27), 24.13 (C-16), 23.42 (C-30), 22.84 (C-11), 18.35 (C-6), 16.97 (C-24), 15.57 (C-25), 15.35 (C-26). HRMS (ESI; *m/z*). Calcd for $C_{41}H_{63}N_2O_3$, $[M + H]^+$, 631.4833; found, 631.4815.

2.13. 1-(oleanoloyl)-(4-(3-(trifluoromethyl)phenyl)piperazin) (**3n**).

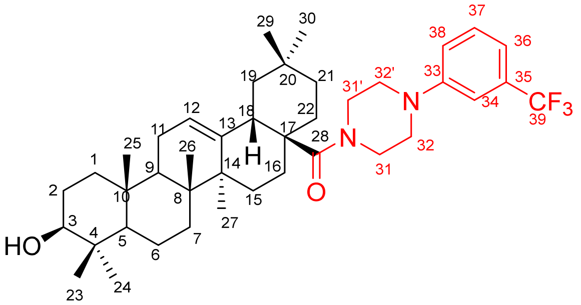

White solid, yield 90%. m.p. 124.3–126.6 °C. IR (KBr): v = 3444, 2926, 1611, 1507, 1462, 1122, 1004, 727 cm^{-1}. ^{1}H NMR ($CDCl_3$) δ 7.39 (t, J = 7.8 Hz, 1H, H-37), 7.17 – 7.10 (m, 2H, H-38, H-36), 7.08 (d, J = 8.8 Hz, 1H, H-34), 5.30 (dd, J = 3.1, 3.1 Hz, 1H, H-12), 3.88 – 3.76 (m, 3H, H-31, H-3), 3.53 – 3.46 (m, 1H, H-18), 3.34 – 3.07 (m, 6H, H-31',H-32, H-32'), 2.30 – 1.20 (m, 22H, other aliphatic ring protons), 1.17 (s, 3H, CH_3), 1.00 (s, 3H, CH_3), 0.96 (s, 3H, CH_3), 0.93 (s, 3H, CH_3), 0.92 (s, 3H, CH_3), 0.79 (s, 3H, CH_3), 0.76 (s, 3H, CH_3). ^{13}C NMR ($CDCl_3$) δ175.22 (C-28), 151.03 (C-33), 144.60 (C-13), 131.55(q, J = 31.6 Hz, C-35), 129.66 (C-38), 121.70 (C-12), 121.06 (q, J = 272.5 Hz, C-39), 118.77 (C-34), 116.27 (C-36), 112.12 (C-37), 79.03 (C-3), 55.32 (C-5), 48.82 (C-32,C-32'), 47.79(C-31), 47.48 (C-31'), 46.64 (C-9), 46.39 (C-19), 45.10 (C-17), 43.55 (C-14), 41.88 (C-18), 39.16 (C-8), 38.76 (C-4), 38.42 (C-1), 37.11 (C-10), 34.01 (C-21), 33.07 (C-29), 32.85 (C-7), 30.42 (C-22), 30.09 (C-20), 28.11 (C-15), 27.96 (C-23), 27.22 (C-2), 25.99 (C-27), 24.08 (C-16), 23.40 (C-30), 22.91 (C-11), 18.32 (C-6), 16.94 (C-24), 15.56 (C-25), 15.35 (C-26). HRMS (ESI; *m/z*). Calcd for $C_{41}H_{60}N_2O_2F_3$, $[M + H]^+$, 669.4601; found, 669.4601.

2.14. 1-(oleanoloyl)-(4-(2,4-difluorophenyl)piperazin) (**3o**).

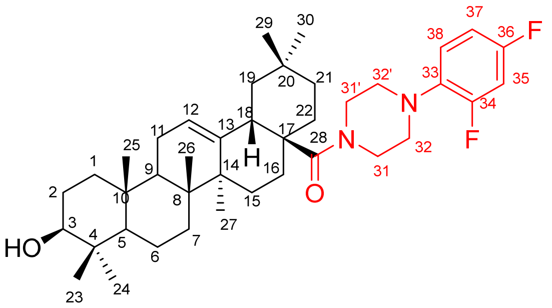

White solid, yield 90%. m.p. 140.1–142.2 °C. IR (KBr): v = 3444, 2942, 1623, 1507, 1463, 1387, 1276, 1138, 1004, 727 cm^{-1}. ^{1}H NMR ($CDCl_3$) δ 6.92 – 6.85 (m, 1H, H-35), 6.85 – 6.78 (m, 2H, H-37, H-38), 5.28 (dd, J = 3.1, 3.1 Hz, 1H, H-12), 3.80 (d, J = 3.8 Hz, 4H, H-31, H-31'), 3.21 (dd, J = 15.0, 5.0 Hz, 1H, H-3), 3.11 (dd, J = 16.0,

4.0 Hz, 1H, H-18), 3.06 – 2.92 (m, 4H, H-32, H-32'), 2.21 – 1.27 (m, 22H, other aliphatic ring protons), 1.15 (s, 3H, CH_3), 0.99 (s, 3H, CH_3), 0.94 (s, 3H, CH_3), 0.91 (s, 6H, 2×CH_3), 0.78 (s, 3H, CH_3), 0.75 (s, 3H, CH_3). ^{13}C NMR ($CDCl_3$) δ 175.17 (C-28), 158.24 (dd, J = 272.5, 11.3 Hz, C-34), 155.88 (dd, J = 272.5, 11.3 Hz, C-36), 136.38 (dd, J = 8.8, 2.5 Hz, C-33), 121.62 (C-12), 119.83 (dd, J = 8.8, 3.8 Hz, C-38), 110.92 (dd, J = 21.4, 3.8 Hz, C-37), 104.97 (dd, J = 25.2, 25.2 Hz, C-35), 79.05 (C-3), 55.44 (C-5), 51.30 (C-32,C-32'), 47.92 (C-31,C-31'), 47.62 (C-9), 46.54 (C-19), 45.65 (C-17), 43.73 (C-14), 42.01 (C-18), 39.27 (C-8), 38.89 (C-4), 38.53 (C-1), 37.24 (C-10), 34.16 (C-21), 33.21 (C-29), 33.00 (C-7), 30.55 (C-22), 30.20 (C-20), 28.24 (C-15), 28.09 (C-23), 27.34 (C-2), 26.13 (C-27), 24.21 (C-16), 23.53 (C-30), 22.95 (C-11), 18.47 (C-6), 17.09 (C-24), 15.70 (C-25), 15.48 (C-26). HRMS (ESI; *m/z*). Calcd for $C_{40}H_{59}N_2O_2F_2$, $[M + H]^+$, 637.4539; found, 637.4531.

2.15. 1-(2-(4-(oleanoloyl)piperazin-1-yl)-2-oxoethyl)-5-methylpyrimidine-2,4(1H,3H)-dione (**3p**).

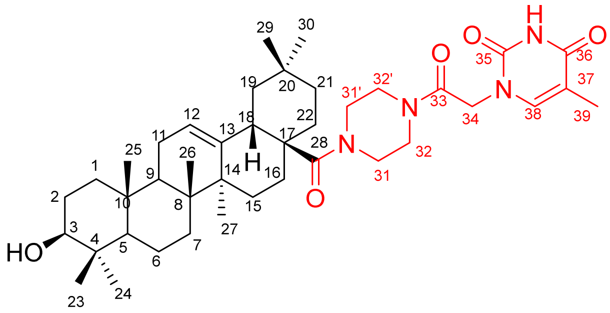

White solid, yield 69%. m.p. 207.8–209.1 °C. IR (KBr): ν = 3395, 2926, 1674, 1521, 1464, 1387, 1022 cm^{-1}. ^{1}H NMR ($CDCl_3$) δ 9.18 (s, 1H, NH), 7.01 (s, 1H, H-38), 5.27 (dd, *J* = 3.1, 3.1 Hz, 1H, H-12), 4.53 (d, J = 2.6 Hz, 2H, H-34), 4.00 – 3.41 (m, 9H, H-31, H-31', H-32, H-32', H-3), 3.11 (dd, *J* = 16.0, 4.0 Hz, 1H, H-18), 1.80 – 1.18 (m, 20H, other aliphatic ring protons), 1.90 (s, 3H, H-39), 1.14 (s, 3H, CH_3), 0.99 (s, 3H, CH_3), 0.93 (s, 3H, CH_3), 0.90 (s, 6H, 2×CH_3), 0.78 (s, 3H, CH_3), 0.72 (s,3H, CH_3). ^{13}C NMR ($CDCl_3$) δ 175.54 (C-28), 165.13 (C-33), 164.14 (C-36), 151.12 (C-35), 144.40 (C-13), 140.76 (C-38), 121.85 (C-12), 110.97 (C-37), 79.00 (C-3), 55.28 (C-5), 47.73 (C-32,C-32'), 47.57 (C-31,C-31'), 46.29 (C-34), 45.07 (C-19), 44.73 (C-9), 43.54 (C-17), 42.30 (C-14), 41.86 (C-18), 39.14 (C-8), 38.76 (C-4), 38.39 (C-1), 37.09 (C-10), 33.92 (C-21), 33.04 (C-29), 32.82 (C-7), 30.40 (C-22), 30.06 (C-20), 28.12 (C-15), 27.90 (C-23), 27.17 (C-2), 25.98 (C-27), 24.04 (C-16), 23.38 (C-30), 22.80 (C-11), 18.30 (C-6), 16.93 (C-24), 15.60 (C-25), 15.35 (C-26), 12.40 (C-39). HRMS (ESI; *m/z*). Calcd for $C_{41}H_{63}N_4O_5$, $[M + H]^+$, 691.4793; found, 691.4805.

2.16. 7-(2-(4-(oleanoloyl)piperazin-1-yl)-2-oxoethyl)-3,7-dihydro-1H-purine-2,6-dione (**3q**).

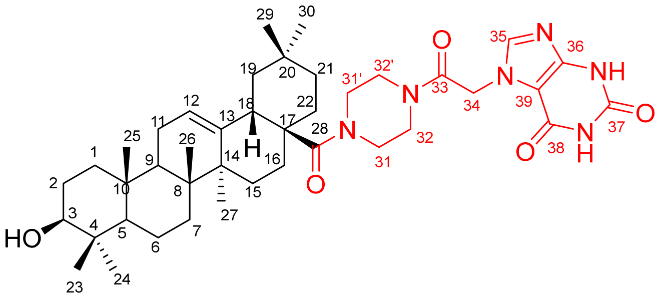

White solid, yield 80%. m.p. 246.1–247.9 °C. IR (KBr): ν = 3395, 2929, 1637, 1521, 1463, 1029, 729 cm^{-1}. ^{1}H NMR ($CDCl_3$) δ 7.71 (s, 1H, H-35), 5.32 – 5.19 (m, 2H, H-12, H-34), 3.84 – 3.42 (m, 8H, H-31, H-31', H-32, H-32'), 3.23 – 1.18 (m, 26H, other aliphatic ring protons), 1.09 (s, 3H, CH_3), 0.92 (s, 3H, CH_3), 0.87 (s, 3H, CH_3), 0.84 (s, 6H, 2×CH_3), 0.71 (s, 3H, CH_3), 0.66 (s, 3H, CH_3). ^{13}C NMR ($CDCl_3$) δ 175.83 (C-28), 164.74 (C-33), 155.93 (C-38), 151.84 (C-37), 148.82 (C-35), 144.31 (C-13), 143.65 (C-36), 121.85 (C-12), 107.33 (C-39), 78.81 (C-3), 55.23 (C-5), 47.65 (C-32,C-32'), 47.59 (C-31,C-31'), 46.82 (C-19), 46.22 (C-9), 45.02 (C-17), 43.53 (C-14), 42.49 (C-34), 41.81 (C-18), 39.09 (C-8), 38.67 (C-4), 38.37 (C-1), 37.00 (C-10), 33.84 (C-21), 32.95 (C-29), 32.76 (C-7), 30.32 (C-22), 29.96 (C-20), 28.00 (C-15), 27.83 (C-23), 26.85 (C-2), 25.93 (C-27), 23.93 (C-16), 23.34 (C-30), 22.67 (C-11), 18.24 (C-6), 16.79 (C-24), 15.55 (C-25), 15.27 (C-26). HRMS (ESI; *m/z*). Calcd for $C_{41}H_{61}N_6O_5$, $[M + H]^+$,715.4552; found, 715.4563.

3. The synthesis of N-(3-((2-((3-aminopropyl)amino)ethyl)amino)propyl) oleanolamide (**3c**)

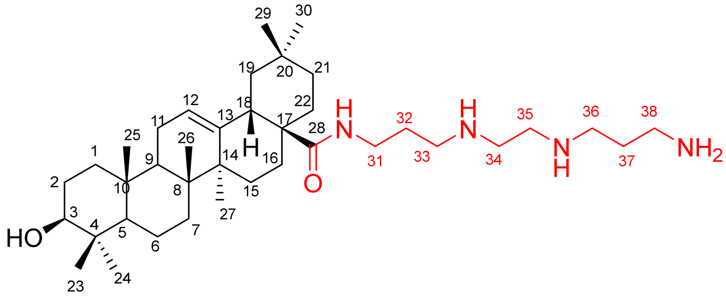

To a solution of **2** (0.50 g, 0.87 mmol) in DMF (6 mL), 1-Boc-1,5,8,12-Tetraazadodecane (0.29 g, 1.04 mmol) and potassium carbonate (0.18 g, 1.30 mmol) were added. The mixture was stirred at room temperature under nitrogen atmosphere for 1 hour, and then added with water (15 mL) and extracted with ethyl acetate (3 × 15 mL). The combined organic layer was washed with brine for three times, dried over anhydrous sodium sulfate, filtered,

and concentrated under reduced pressure. The residue was purified by using flush column chromatography on silica gel. The resulting white solid was added to ethyl acetate saturated with hydrogen chloride (5 mL). The mixture was stirred for 7 hours at room temperature. The reaction mixture was filtered. and the filter cake was dissolved in water. The solution was adjusted to pH 7.5 by addition of 1N $NaHCO_3$ solution. Then the mixture was extracted with diethyl ether (3 × 15 mL). The combined organic layer was dried over Na_2SO_4 and the solvent was evaporated to afford the target compound as white solid. Yield 80%. m.p. 106.6–107.9 °C. IR (KBr): v = 3315, 2940, 1676, 1540, 1463, 749, 700 cm^{-1}. ^{1}H NMR ($CDCl_3$) *δ* 6.46 (br s, 1H, NH), 5.35 (dd, *J* = 3.1, 3.1 Hz, 1H, H-12), 3.49 – 3.40 (m, 1H, H-31a), 3.21 (dd, *J* = 10.9, 4.5 Hz, 1H, H-3), 3.12 – 3.04 (m, 1H, H-31b), 2.89 – 2.59 (m, 10H, H-3, H-33, H-34, H-35, H-36, H-38), 2.01 – 1.18 (m, 30H, protons of other aliphatic ring and chain), 1.16 (s, 3H, CH_3), 0.99 (s, 3H, CH_3), 0.91 (s, 9H, 3×CH_3), 0.78 (s, 3H, CH_3), 0.76 (s, 3H, CH_3). ^{13}C NMR ($CDCl_3$) *δ* 178.26 (C-28), 144.92 (C-13), 122.65 (C-12), 78.91 (C-3), 55.12 (C-5), 49.38 (C-34), 49.28 (C-35), 47.82 (C-33), 47.78 (C-36), 47.56 (C-9), 46.77 (C-17), 46.22 (C-19), 42.10 (C-18), 42.01, 40.49 (C-14), 39.37 (C-8), 38.77 (C-4), 38.47 (C-1), 38.04 (C-38), 36.98 (C-10), 34.13 (C-21), 33.33 (C-37), 33.02 (C-7), 32.76 (C-29), 32.38 (C-22), 30.75 (C-20), 29.40 (C-32), 28.11 (C-15), 27.32 (C-23), 27.16 (C-2), 25.83 (C-27), 23.68 (C-16), 23.65 (C-11), 23.54 (C-30), 18.30 (C-6), 16.95 (C-24), 15.59 (C-25), 15.37 (C-26). HRMS (ESI; *m/z*). Calcd for $C_{38}H_{69}N_4O_2$, $[M + H]^+$, 613.5415; found, 613.5422.

4. General synthetic procedure of **4a**-**4o.**

4.1. N-[[1-(2-cyanoethyl)-1H-1,2,3-triazol-4-yl]methyl] oleanolamide (**4a**).

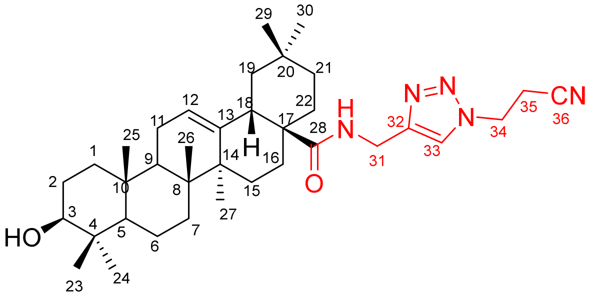

White solid, yield 85%. m.p. 137.6–139.3 °C. IR (KBr): v = 3395, 2928, 1636, 1521, 1463, 1029, 749 cm^{-1}. ^{1}H NMR (400 MHz, $CDCl_3$) δ 7.78 (s, 1H, H-33), 6.78 (br s, 1H, NH), 5.39 (dd, *J* = 3.1, 3.1 Hz, 1H, H-12), 4.64 (t, *J* = 6.6 Hz, 2H, H-34), 4.52 (dd, *J* = 15.0, 5.5 Hz, 1H, H-31a), 4.35 (dd, *J* = 15.0, 5.3 Hz, 1H, H-31b), 3.20 (dd, *J* = 10.2, 5.0 Hz, 1H, H-3), 3.05 (t, *J* = 6.7 Hz, 2H, H-35), 2.69 – 2.42 (m, 1H, H-18), 2.10 – 1.18 (m, 22H, other

aliphatic ring protons), 1.14 (s, 3H, CH_3), 0.98 (s, 3H, CH_3), 0.89 (s, 3H, CH_3), 0.88 (s, 3H, CH_3), 0.87 (s, 3H, CH_3), 0.77 (s, 3H, CH_3), 0.54 (s, 3H, CH_3). ^{13}C NMR ($CDCl_3$) δ 178.50 (C-28), 145.34 (C-32), 144.22 (C-13), 123.35 (C-12), 123.14 (C-33), 116.37 (C-36), 78.73 (C-3), 55.06 (C-5), 47.47 (C-19), 46.57 (C-9), 46.17 (C-17), 45.47 (C-34), 41.87 (C-31, C-14), 39.28 (C-18), 38.73 (C-8), 38.44 (C-4), 36.89 (C-1), 34.90 (C-10), 34.05 (C-21), 32.99 (C-29), 32.50 (C-7), 32.31 (C-22), 30.67 (C-20), 28.12 (C-15), 27.21 (C-23), 27.12 (C-2), 25.75 (C-27), 23.76 (C-16), 23.58 (C-30), 23.41 (C-11), 19.30 (C-35), 18.24 (C-6), 16.50 (C-24), 15.67 (C-25), 15.36 (C-26). HRMS (ESI; *m/z*). Calcd for $C_{36}H_{56}N_5O_2$, $[M + H]^+$, 590.4429; found, 590.4419.

4.2. 3-(4-((N-(oleanoloyl))aminomethyl)-1H-1,2,3-triazol-1-yl)propanoic acid (**4b**).

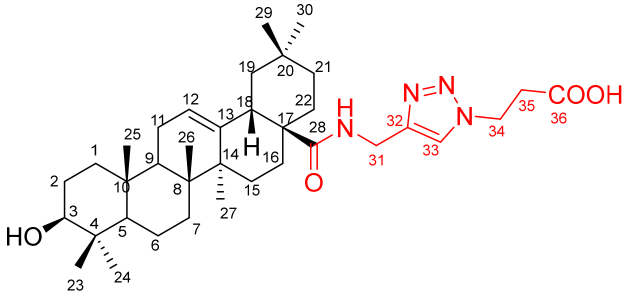

White solid, yield 70%. m.p. 150.3–152.2 °C. IR (KBr): ν = 3418, 2927, 1622, 1508, 1456, 1229, 1025 cm^{-1}. ^{1}H NMR ($CDCl_3$) δ 7.85 (s, 1H, H-33), 6.85 (t, J = 5.5 Hz, 1H, NH), 5.30 (dd, J = 3.1, 3.1 Hz, 1H, H-12), 4.65 (t, J = 6.1 Hz, 2H, H-34), 4.44 (ddd, J = 39.7, 14.9, 5.5 Hz, 2H, H-31), 3.21 (dd, J = 10.2, 5.0 Hz, 1H, H-3), 2.97 (t, J = 6.1 Hz, 2H, H-35), 2.59 – 2.46 (m, 1H, H-18), 2.10 – 1.17 (m, 23H, other aliphatic ring protons), 1.13 (s, 3H, CH_3), 0.97 (s, 3H, CH_3), 0.89 (s, 3H, CH_3), 0.87 (s, 6H, CH_3), 0.77 (s, 3H, CH_3), 0.47 (s, 3H, CH_3). ^{13}C NMR ($CDCl_3$) δ 178.89 (C-28), 172.70 (C-36), 144.13 (C-32), 143.84 (C-13), 124.12 (C-33), 123.26 (C-12), 79.01 (C-3), 55.08 (C-5), 47.47 (C-19), 46.81 (C-9), 46.51 (C-34), 46.23 (C-17), 45.84 (C-31), 41.82 (C-31), 41.77 (C-14), 39.27 (C-18), 38.71 (C-8), 38.45 (C-4), 36.89 (C-1), 34.62 (C-10), 34.53 (C-21), 34.04 (C-35), 32.99 (C-29), 32.47 (C-7), 32.35 (C-22), 30.67 (C-20), 28.09 (C-15), 27.18 (C-23), 26.99 (C-2), 25.77 (C-27), 23.56 (C-16), 23.55 (C-30)，23.41 (C-11), 18.25 (C-6), 16.35 (C-24), 15.64 (C-25), 15.35 (C-26). HRMS (ESI; *m/z*). Calcd for $C_{36}H_{57}N_4O_4$, $[M + H]^+$, 609.4374; found, 609.4369.

4.3. ethyl 2-(4-((N-(oleanoloyl))aminomethyl)-1H-1,2,3-triazol-1-yl)acetate (**4c**).

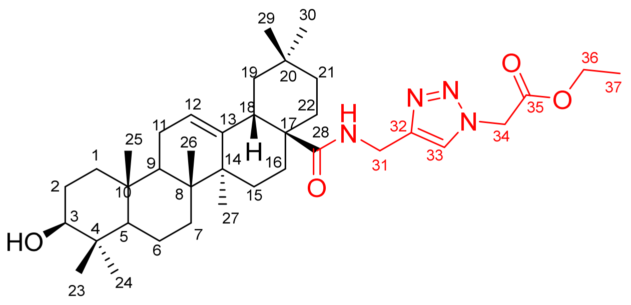

White solid, yield 81%. m.p. 120.3–123.1 °C. IR (KBr): ν = 3418, 2927, 1611, 1508, 1463, 1229, 1025, 727 cm^{-1}. ^{1}H NMR ($CDCl_3$) δ 7.68 (s, 1H, H-33), 6.65 (t, J = 5.3 Hz, 1H, NH), 5.40 (t, J = 3.2 Hz, 1H, H-12), 5.14 (t, J = 10.4 Hz, 2H, H-34), 4.56 (dd, J = 15.0, 5.6 Hz, 1H, H-31a), 4.37 (dd, J = 15.0, 5.3 Hz, 1H, H-31b), 4.26 (q, J = 7.1 Hz, 2H, H-36), 3.21 (dd, J = 11.0, 4.5 Hz, 1H, H-3), 2.73 – 2.44 (m, 1H, H-18), 2.10 – 1.18 (m, 25H, other aliphatic ring protons), 1.15 (s, 3H, CH_3), 0.98 (s, 3H, CH_3), 0.91 – 0.86 (m, 9H, 3 × CH_3), 0.77 (s, 3H, CH_3), 0.57 (s, 3H, CH_3). ^{13}C NMR ($CDCl_3$) δ 178.51 (C-28), 166.13 (C-35), 145.11 (C-32), 144.31 (C-13), 123.96 (C-33), 123.28 (C-12), 78.95 (C-3), 62.44 (C-36), 55.09 (C-5), 50.83 (C-34), 47.54 (C-19), 46.65 (C-9), 46.24 (C-17), 42.02 (C-31), 41.95 (C-14), 39.33 (C-18), 38.75 (C-8), 38.46 (C-4), 36.94 (C-1), 34.98 (C-10), 34.10 (C-21), 33.00 (C-29), 32.42 (C-7), 32.31 (C-22), 30.72 (C-20), 28.09 (C-15), 27.25 (C-23), 27.16 (C-2), 25.77 (C-27), 23.87 (C-16), 23.60 (C-30), 23.46 (C-11), 18.26 (C-6), 16.54 (C-24), 15.57 (C-25), 15.35 (C-26), 14.09 (C-37). HRMS (ESI; *m/z*). Calcd for $C_{37}H_{59}N_4O_4$, $[M + H]^+$,623.4531; found, 623.4528.

4.4. N-[[1-(1-benzyl)-1H-1,2,3-triazol-4-yl]methyl] oleanolamide (**4d**).

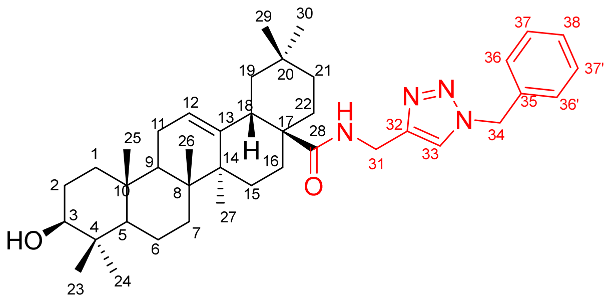

Light yellow solid, yield 79%. m.p. 120.1–122.9 °C. IR (KBr): ν = 3418, 2939, 1637, 1521, 1463, 1048, 698 cm^{-1}. ^{1}H NMR ($CDCl_3$) δ 7.48 (s, 1H, H-33), 7.38 – 7.32 (m, 2H, H-36, H-36', H-38), 7.30 – 7.23 (m, 2H, H-37, H-37'), 6.60 (s, 1H, NH), 5.48 (q, J = 14.8 Hz, 2H, H-34), 5.37 (dd, J = 3.1, 3.1 Hz, 1H, H-12), 4.48 (dd, J = 15.0, 5.5 Hz, 1H, H-31a), 4.35 (dd, J = 15.0, 5.3 Hz, 1H, H-31b), 3.20 (dd, J = 10.2, 5.0 Hz, 1H, H-3), 2.55 – 2.47 (m, 1H,

H-18), 2.00 – 1.23 (m, 22H, other aliphatic ring protons), 1.13 (s, 3H, CH_3), 0.98 (s, 3H, CH_3), 0.89 (s, 3H, CH_3), 0.87 (s, 3H, CH_3), 0.85 (s, 3H, CH_3), 0.78 (s, 3H, CH_3), 0.46 (s, 3H, CH_3). ^{13}C NMR ($CDCl_3$) δ 178.34 (C-28), 145.13 (C-32), 144.16 (C-13), 134.56 (C-35), 129.03 (C-37, C-37'), 128.72 (C-38), 128.06 (C-36, C-36'), 123.15 (C-12), 122.54 (C-33), 78.70 (C-3), 55.09 (C-5), 54.09 (C-34), 47.48 (C-19), 46.60 (C-9), 46.16 (C-17), 41.88 (C-14), 39.25 (C-31), 38.73 (C-18), 38.60 (C-8), 38.47 (C-4), 36.88(C-1), 34.97 (C-10), 34.09 (C-21), 33.00 (C-29), 32.45 (C-7), 32.32 (C-22), 30.67 (C-20), 28.14 (C-15), 27.18 (C-23), 27.13 (C-2), 25.72 (C-27), 23.75 (C-16), 23.58 (C-30), 23.41 (C-11), 18.28 (C-6), 16.46 (C-24), 15.69 (C-25), 15.37 (C-26). HRMS (ESI; *m/z*). Calcd for $C_{40}H_{59}N_4O_2$, $[M + H]^+$,627.4633; found, 627.4640.

4.5. N-[[1-(3-phenylpropyl)-1H-1,2,3-triazol-4-yl]methyl] oleanolamide (**4e**).

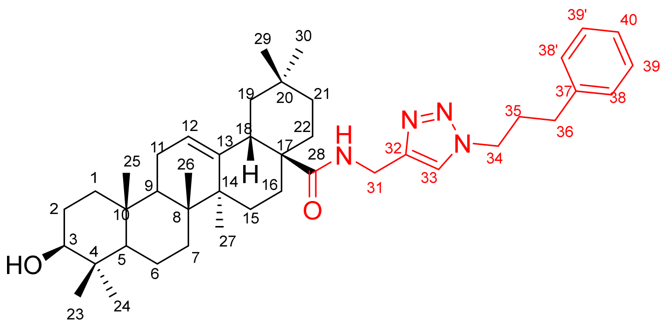

White solid, yield 90%. m.p. 121.3–122.8 °C. IR (KBr): ν = 3418, 2927, 1637, 1520, 1463, 1048, 698 cm^{-1}. ^{1}H NMR ($CDCl_3$) δ 7.53 (s, 1H, H-33), 7.41 – 7.01 (m, 5H, Ar-H), 6.63 (br s, 1H, NH), 5.39 (s, 1H, H-12), 4.50-4.80 (m, 2H, H-34), 4.46 – 4.23 (m, 2H, H-31), 3.48 (s, 1H, H-3), 3.20 (s, 1H, H-18), 3.00 – 1.41 (m, 25H, protons of other aliphatic ring and chain), 1.13 (s, 3H, CH_3), 0.97 (s, 3H, CH_3), 0.89 (s, 3H, CH_3), 0.88 (s, 3H, CH_3), 0.83 (s, 3H, CH_3), 0.75 (s, 3H, CH_3), 0.51 (s, 3H, CH_3). ^{13}C NMR ($CDCl_3$) δ 178.46 (C-28), 144.73 (C-32), 144.28 (C-13), 140.14 (C-35), 128.64 (C-39, C-39'), 128.41 (C-38, C-38'), 126.39 (C-40), 123.22 (C-12), 122.60 (C-33), 78.91 (C-3), 55.06 (C-5), 49.52 (C-34), 47.50 (C-19), 46.66 (C-9), 46.22 (C-17), 41.98 (C-14), 41.92 (C-18), 39.30 (C-8), 38.73 (C-4), 38.44 (C-1), 36.90 (C-31), 35.02 (C-10), 34.11 (C-21), 33.00 (C-29), 32.53 (C-36), 32.38 (C-7), 32.33 (C-22), 31.72 (C-36), 30.72 (C-20), 30.18 (C-35), 28.08 (C-15), 27.24 (C-23), 27.15 (C-2), 25.75 (C-27), 23.85 (C-16), 23.59 (C-30), 23.46 (C-11), 18.22 (C-6), 16.55 (C-24), 15.58 (C-25), 15.36 (C-26). HRMS (ESI; *m/z*). Calcd for $C_{42}H_{63}N_4O_2$, $[M + H]^+$, 655.4946; found, 655.4962.

4.6. N-[[1-(2-(dimethylamino)ethyl)-1H-1,2,3-triazol-4-yl]methyl] oleanolamide (**4f**).

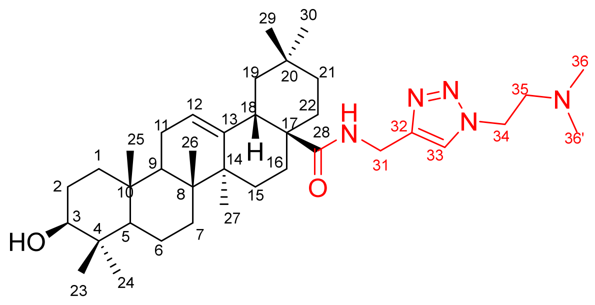

Light yellow solid, yield 70%. m.p. 121.7–123.3 °C. IR (KBr): ν = 3420, 2927, 1637, 1521, 1463, 1048, 729 cm^{-1}. ^{1}H NMR ($CDCl_3$) δ 7.64 (s, 1H, H-33), 6.63 (t, J = 5.2 Hz, 1H, NH), 5.39 (dd, J = 3.1, 3.1 Hz, 1H, H-12), 4.53 (dd, J = 14.9, 5.5 Hz, 1H, H-31a), 4.41 (t, J = 6.4 Hz, 2H, H-34), 4.35 (dd, J = 15.0, 5.2 Hz, 1H, H-31b), 3.21 (dd, J = 11.0, 4.4 Hz, 1H, H-3), 2.75 (t, J = 6.5 Hz, 1H, H-35), 2.56 (dd, J = 16.0, 4.0 Hz, 1H, H-18), 2.28 (s, 6H, H-36, H-36') , 2.08 – 1.18 (m, 24H, other aliphatic ring protons), 1.14 (s, 3H, CH_3), 0.98 (s, 3H, CH_3), 0.89 (s, 3H, CH_3), 0.88 (s, 3H, CH_3), 0.87 (s, 3H, CH_3), 0.77 (s, 3H, CH_3), 0.54 (s, 3H, CH_3). ^{13}C NMR ($CDCl_3$) δ 178.37 (C-28), 144.57 (C-13), 144.27 (C-32), 123.22 (C-12), 122.95 (C-33), 78.95 (C-3), 58.78 (C-35), 55.09 (C-5), 48.16 (C-34), 47.54 (C-19), 46.66 (C-9), 46.24 (C-17), 45.41 (C-36, C-36'), 42.01 (C-31), 41.94 (C-14), 39.33 (C-18), 38.75 (C-8), 38.46 (C-4), 36.95 (C-1), 35.00 (C-10), 34.12 (C-21), 33.00 (C-29), 32.48 (C-7), 32.36 (C-22), 30.72 (C-20), 28.08 (C-15), 27.26 (C-23), 27.16 (C-2), 25.76 (C-27), 23.85 (C-16), 23.58 (C-30), 23.46 (C-11), 18.27 (C-6), 16.54 (C-24), 15.57 (C-25), 15.42 (C-26). HRMS (ESI; *m/z*). Calcd for $C_{37}H_{62}N_5O_2$, $[M + H]^+$,608.4898; found, 608.4901.

4.7. N-[[1-(2-(pyrrolidin-1-yl)ethyl)-1H-1,2,3-triazol-4-yl]methyl] oleanolamide (**4g**).

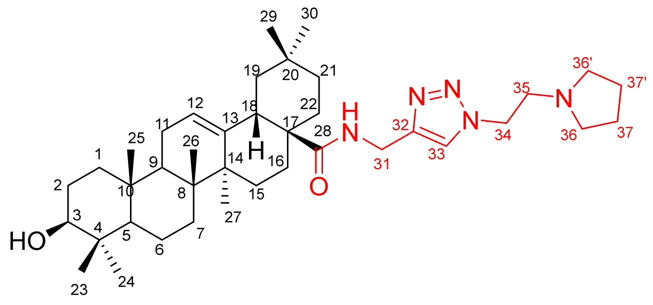

Light yellow solid, yield 70%. m.p. 116.3–117.9 °C. IR (KBr): ν = 3396, 2927, 1638, 1520, 1462, 1047, 731 cm^{-1}. ^{1}H NMR ($CDCl_3$) δ 7.64 (s, 1H, H-33), 6.62 (t, J = 5.3 Hz, 1H, NH), 5.39 (d, J = 3.1 Hz, 1H, H-12), 4.53 (dd, J = 15.0, 5.5 Hz, 1H, H-31a), 4.45 (t, J = 6.7 Hz, 2H, H-34), 4.35 (dd, J = 15.0, 5.3 Hz, 1H, H-31b), 3.21 (dd, J = 11.0,

4.6 Hz, 1H, H-3), 2.94 (t, *J* = 6.7 Hz, 2H, H-31) , 2.66 – 2.45 (m, 5H, H-18, H-36, H-36'), 2.09 – 1.18 (m, 26H, other aliphatic ring protons), 1.14 (s, 3H, CH_3), 0.98 (s, 3H, CH_3), 0.90 (s, 3H, CH_3), 0.88 (s, 3H, CH_3), 0.87 (s, 3H, CH_3), 0.77 (s, 3H, CH_3), 0.53 (s, 3H, CH_3). ^{13}C NMR ($CDCl_3$) δ 178.32 (C-28), 144.59 (C-13), 144.28 (C-32), 123.21 (C-12), 122.93 (C-33), 78.90 (C-3), 55.57 (C-35), 55.08 (C-5), 54.12 (C-36, C-36'), 49.32 (C-34), 47.52 (C-19), 46.65 (C-9), 46.22 (C-17), 42.01 (C-31), 41.93 (C-14), 39.32 (C-18), 38.75 (C-8), 38.45 (C-4), 36.94 (C-1), 34.98 (C-10), 34.12 (C-21), 33.01 (C-29), 32.47 (C-7), 32.35 (C-22), 30.72 (C-20), 28.10 (C-15), 27.26 (C-23), 27.17 (C-2), 25.76 (C-27), 23.83 (C-16), 23.60 (C-30), 23.57 (C-37, C-37'), 23.46 (C-11), 18.27 (C-6), 16.54 (C-24), 15.58 (C-25), 15.42 (C-26). HRMS (ESI; *m/z*). Calcd for $C_{39}H_{64}N_5O_2$, $[M + H]^+$,634.5055; found, 634.5058.

4.8. N-[[1-(3-(pyrrolidin-1-yl)propyl)-1H-1,2,3-triazol-4-yl]methyl] oleanolamide (**4h**).

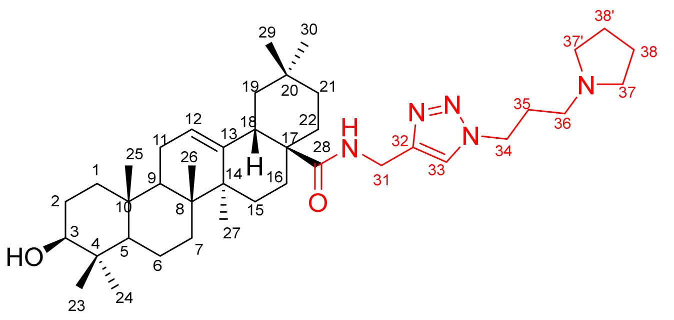

White solid, yield 78%. m.p. 120.9–122.5 °C. IR (KBr): ν = 3397, 2927, 1635, 1520, 1463, 1029, 730 cm^{-1}. ^{1}H NMR ($CDCl_3$) δ 7.55 (s, 1H, H-33), 6.64 (br s, 1H, NH), 5.39 (s, 1H, H-12), 4.64 - 4.28 (m, 4H, H-34, H-31), 3.28 (dd, *J* = 10.2, 5.0 Hz, 1H, H-3), 3.00 – 1.18 (m, 34H, protons of other aliphatic ring and chain), 1.14 (s, 3H, CH_3), 0.98 (s, 3H, CH_3), 0.89 (s, 9H, 3 × CH_3), 0.88 (s, 3H, CH_3), 0.87 (s, 1H), 0.77 (s, 3H, CH_3), 0.53 (s, 3H, CH_3). ^{13}C NMR ($CDCl_3$) δ 178.35 (C-28), 144.56 (C-13), 144.28 (C-32), 123.22 (C-12), 122.74 (C-33), 78.87 (C-3), 55.09 (C-5), 54.04 (C-37, C-37'), 52.66 (C-36), 48.31 (C-34), 47.53 (C-19), 46.65 (C-9), 46.22 (C-17), 42.00 (C-31), 41.93 (C-14), 39.32 (C-18), 38.75 (C-8), 38.46 (C-4), 36.93 (C-1), 35.01 (C-10), 34.11 (C-21), 33.00 (C-29), 32.48 (C-7), 32.36 (C-22), 30.71 (C-20), 29.47 (C-35), 28.10 (C-15), 27.26 (C-23), 27.17 (C-2), 25.75 (C-27), 23.84 (C-16), 23.60 (C-30), 23.48 (C-38, C-38', C-11), 18.27 (C-6), 16.54 (C-24), 15.58 (C-25), 15.39 (C-26). HRMS (ESI; *m/z*). Calcd for $C_{40}H_{66}N_5O_2$, $[M + H]^+$, 648.5211; found, 648.5200.

4.9. N-[[1-(2-morpholinoethyl)-1H-1,2,3-triazol-4-yl]methyl] oleanolamide (**4i**).

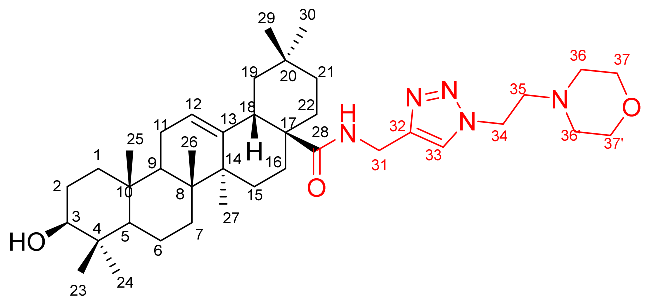

Light yellow solid, yield 78%. m.p. 130.1–131.8 °C. IR (KBr): ν = 3419, 2926, 2852, 1641, 1521, 1457, 1116, 757 cm^{-1}. ^{1}H NMR ($CDCl_3$) δ 7.67 (s, 1H, H-33), 6.67 (br s, 1H, NH), 5.39 (dd, J = 3.1, 3.1 Hz, 1H, H-12), 4.55 (dd, J = 15.0, 5.5 Hz, 1H, H-31a), 4.46 – 4.39 (m, 2H, H-34), 4.30 (dd, J = 15.0, 5.3 Hz, 1H, H-31b), 3.69 (s, 4H, H-37, H-37'), 3.48 (s, 1H, H-3), 3.21 (d, J = 7.6 Hz, 1H, H-18), 2.81 (dd, J = 8.1, 4.2 Hz, 2H, H-35), 2.56 (dd, J = 16.0, 4.0 Hz, 1H, H-18), 2.50 – 2.43(m, 4H, H-36, H-36'), 2.18 – 1.18 (m, 20H, other aliphatic ring protons), 1.14 (s, 3H, CH_3), 0.98 (s, 3H, CH_3), 0.93 – 0.84 (m, 9H, 3 × CH_3), 0.77 (s, 3H, CH_3), 0.54 (s, 3H, CH_3);^{13}C NMR ($CDCl_3$) δ 178.43 (C-28), 144.68 (C-13), 144.26 (C-32), 123.21 (C-12), 123.10 (C-33), 78.88 (C-3), 66.83 (C-37, C-37'), 57.93 (C-35), 55.08 (C-5), 53.51 (C-36, C-36'), 47.51 (C-19), 47.34 (C-34), 46.62 (C-9), 46.22 (C-17), 41.97 (C-31), 41.93 (C-14), 39.31 (C-18), 38.75 (C-8), 38.45 (C-4), 36.93 (C-1), 34.96 (C-10), 34.09 (C-21), 32.98 (C-29), 32.49 (C-7), 32.35 (C-22), 30.70 (C-20), 28.09 (C-15), 27.25 (C-23), 27.13 (C-2), 25.76 (C-27), 23.81 (C-16), 23.57 (C-30), 23.45 (C-11), 18.27 (C-6), 16.54 (C-24), 15.59 (C-25), 15.41 (C-26). HRMS (ESI; *m/z*). Calcd for $C_{39}H_{64}N_5O_2$, $[M + H]^+$, 650.5004; found, 650.4993.

4.10. N-[[1-(3-morpholinopropyl)-1H-1,2,3-triazol-4-yl]methyl] oleanolamide (**4j**).

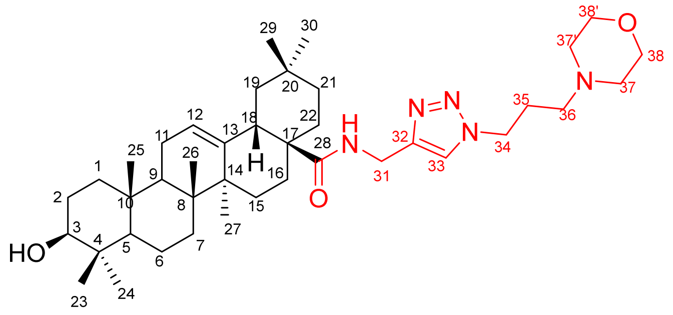

Light yellow solid, yield 79%. m.p. 116.7–118.3 °C. IR (KBr): ν = 3395, 2941, 1635, 1520, 1457, 1324, 1118, 749 cm^{-1}. ^{1}H NMR ($CDCl_3$) δ 7.64 – 7.45 (m, 1H, H-33), 6.62 (br s, 1H, NH), 5.38 (dd, J = 3.1, 3.1 Hz, 1H, H-12), 4.55 – 4.25 (m, 4H, H-31, H-34), 3.77 – 3.60 (m, 4H, H-38, H-38'), 3.70 (d, J = 3.7 Hz, 4H), 3.18 (s, 1H, H-3),

, 2.65 – 2.21 (m, 8H, H-35, H-36, H-37, H-37’), 2.18 – 1.20 (m, 23H, other aliphatic ring protons), 1.14 (s, 3H, CH_3), 0.98 (s, 3H, CH_3), 0.87 (s, 9H, 3 × CH_3), 0.77 (s, 3H, CH_3), 0.53 (s, 3H, CH_3). ^{13}C NMR ($CDCl_3$) δ 178.43 (C-28), 144.63 (C-13), 144.29 (C-32), 123.22 (C-12), 122.88 (C-33), 78.88 (C-3), 66.94 (C-38, C-38’), 55.07 (C-5), 54.99 (C-36), 53.54 (C-37, C-37’), 48.01 (C-34), 47.51 (C-19), 46.65 (C-9), 46.22 (C-17), 41.99 (C-31), 41.94 (C-14), 39.31 (C-18), 38.75 (C-8), 38.45 (C-4), 36.93 (C-1), 35.00 (C-10), 34.10 (C-21), 32.99 (C-29), 32.47 (C-7), 32.35 (C-22), 30.71 (C-20), 28.09 (C-15), 27.25 (C-23), 27.15 (C-2), 27.05 (C-35), 25.75 (C-27), 23.85 (C-16), 23.59 (C-30), 23.46 (C-11), 18.26 (C-6), 16.57 (C-24), 15.59 (C-25), 15.39 (C-26). HRMS (ESI; *m/z*). Calcd for $C_{40}H_{66}N_5O_3$, $[M + H]^+$, 664.5160; found, 664.5158.

4.11. N-[[1-(pyrimidin-2-yl)-1H-1,2,3-triazol-4-yl]methyl] oleanolamide (**4k**).

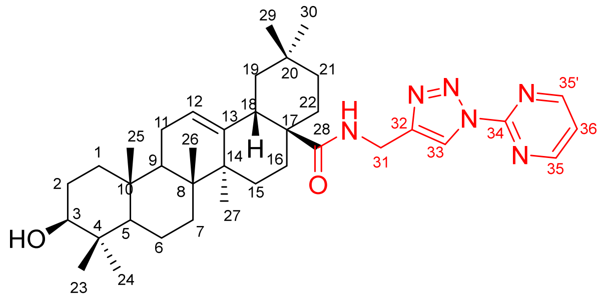

White solid, yield 83%. m.p. 120.9–122.2 °C. IR (KBr): v = 3393, 2927, 1635, 1521, 1462, 1324, 1127, 741 cm^{-1}. ^1H NMR ($CDCl_3$) δ 8.87 (d, J = 4.6 Hz, 2H, H-35, H-35’), 8.58 (s, 1H, H-36), 7.41 (d, J = 4.5 Hz, 1H, H-33), 6.69 (s, 1H, NH), 5.38 (dd, J = 3.1, 3.1 Hz, 1H, H-12), 4.66 (dd, J = 15.0, 5.1 Hz, 1H, H-31a), 4.47 (dd, J = 15.0, 4.7 Hz, 1H, H-31b), 3.20 (dd, J = 10.2, 5.0 Hz, 1H, H-3), 2.56 (dd, J = 16.0, 4.0 Hz, 1H, H-18), 2.18 – 1.20 (m, 23H, other aliphatic ring protons), 1.14 (s, 3H, CH_3), 0.97 (s, 3H, CH_3), 0.89 (s 3H, CH_3), 0.88 (s 3H, CH_3), 0.79 (s, 3H, CH_3), 0.75 (s, 3H, CH_3), 0.57 (s, 3H, CH_3). ^{13}C NMR ($CDCl_3$) δ 178.41 (C-28), 159.28 (C-35, C-35’), 154.40 (C-34), 145.21 (C-32), 144.24 (C-13), 123.30 (C-12), 121.70 (C-33), 120.75 (C-36), 78.89 (C-3), 55.06 (C-5), 47.53 (C-19), 46.60 (C-9), 46.27 (C-17), 42.07 (C-31), 41.92 (C-14), 39.33 (C-18), 38.72 (C-8), 38.43 (C-4), 36.91 (C-1), 34.85 (C-10), 34.09 (C-21), 33.01 (C-29), 32.49 (C-7), 32.32 (C-22), 30.71 (C-20), 28.08 (C-15), 27.29 (C-23), 27.15 (C-2), 25.75 (C-27), 23.81 (C-16), 23.59 (C-30), 23.45 (C-11), 18.23 (C-6), 16.64 (C-24), 15.56 (C-25), 15.24 (C-26). HRMS (ESI; *m/z*). Calcd for $C_{37}H_{55}N_6O_2$, $[M + H]^+$, 615.4381; found, 615.4361.

4.12. N-[[1-(4-(trifluoro methyl)benzyl)-1H-1,2,3-triazol-4-yl]methyl] oleanolamide (**4l**).

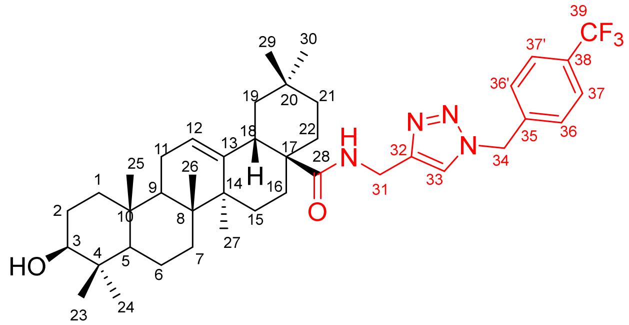

White solid, yield 76%. m.p. 129.9.1–131.4 °C. IR (KBr): ν = 3395, 2928, 1636, 1521, 1463, 1323, 1125, 741 cm^{-1}. ^{1}H NMR ($CDCl_3$) δ 7.62 (d, J = 7.9 Hz, 2H, H-37, H-37'), 7.57 (s, 1H, H-33), 7.37 (d, J = 7.9 Hz, 2H, H-36, H-36'), 6.64 (br s, 1H, NH), 5.55 (q, J = 15.2 Hz, 2H, H-34), 5.38 (s, 1H, H-12), 4.49 (dd, J = 15.0, 5.4 Hz, 1H, H-31a), 4.35 (dd, J = 15.0, 5.3 Hz, 1H, H-31b), 3.20 (dd, J = 10.2, 5.0 Hz, 1H, H-3) , 2.56 (dd, J = 16.0, 4.0 Hz, 1H, H-18), 2.10 – 1.20 (m, 23H, other aliphatic ring protons), 1.13 (s, 3H, CH_3), 0.98 (s, 3H, CH_3), 0.89 (s, 3H, CH_3), 0.87 (s, 3H, CH_3), 0.85 (s, 3H, CH_3), 0.77 (s, 3H, CH_3), 0.48 (s, 3H, CH_3). ^{13}C NMR ($CDCl_3$) δ 178.53 (C-28), 145.52 (C-13), 144.28 (C-32), 138.59 (C-35), 130.93 (q, J = 31.3 Hz, C-38), 128.21 (C-36, C-36'), 126.04 (q, J = 3.78 Hz, C-37, C-37'), 123.74 (q, J = 277.2 Hz, C-39), 123.19 (C-12), 122.86 (C-33), 78.84 (C-3), 55.06 (C-5), 53.40 (C-34), 47.47 (C-19), 46.63 (C-9), 46.20 (C-17), 41.97 (C-31), 41.93 (C-14), 39.27 (C-18), 38.73 (C-8), 38.45 (C-4), 36.89 (C-1), 35.02 (C-10), 34.07 (C-21), 32.96 (C-29), 32.43 (C-7), 32.30 (C-22), 30.68 (C-20), 28.07 (C-15), 27.17 (C-23), 27.13 (C-2), 25.70 (C-27), 23.85 (C-16), 23.53 (C-30), 23.43 (C-11), 18.23 (C-6), 16.55 (C-24), 15.57 (C-25), 15.36 (C-26). HRMS (ESI; *m/z*). Calcd for $C_{41}H_{58}N_4O_2F_3$, $[M + H]^+$, 695.4505; found, 695.4495.

4.13. N-[[1-((1H-benzo[d]imidazol-2-yl)methyl)-1H-1,2,3-triazol-4-yl] methyl] oleanolamide (**4m**).

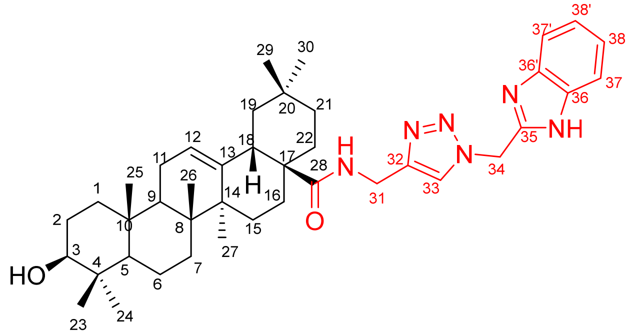

White solid, yield 81%. m.p. 174.0–175.2 °C. IR (KBr): ν = 3394, 2927, 1633, 1521, 1462, 1025, 742 cm^{-1}. ^{1}H

NMR ($CDCl_3$) δ 7.84 (s, 1H, H-33), 7.59 (s, 2H, H-37, H-37'), 7.28 –7.26 (m, 2H, H-38, H-38'), 6.66 (t, *J* = 5.2 Hz, 1H, NH), 5.98 – 5.72 (m, 2H, H-34), 5.32 (d, *J* = 21.1 Hz, 1H, H-12), 4.50 (dd, *J* = 15.1, 5.3 Hz, 1H, H-31a), 4.34 (dd, *J* = 15.1, 5.2 Hz, 1H, H-31b), 3.20 (dd, *J* = 11.3, 4.5 Hz, 1H, H-3), 2.52 (d, *J* = 9.9 Hz, 1H, H-18), 2.01 – 1.20 (m, 22H, other aliphatic ring protons), 1.11 (s, 3H, CH_3), 0.96 (s, 3H, CH_3), 0.88 (s, 3H, CH_3), 0.85 (s, 3H, CH_3), 0.74 (s, 3H, CH_3), 0.73 (s, 3H, CH_3), 0.45 (s, 3H, CH_3). ^{13}C NMR ($CDCl_3$) δ 178.91 (C-28), 144.33 (C-13), 127.41 (C-36, C-36'), 123.55 (C-38, C-38'), 123.35 (C-33), 123.25 (C-12), 100.09 (C-37, C-37'), 78.98 (C-3), 55.01 (C-5), 48.17 (C-34), 47.43 (C-19), 46.59 (C-9), 46.31 (C-17), 42.08 (C-31), 41.96 (C-14), 39.24 (C-18), 38.71 (C-8), 38.39 (C-4), 36.82 (C-1), 35.13 (C-10), 34.04 (C-21), 32.95 (C-29), 32.47 (C-7), 32.25 (C-22), 30.69 (C-20), 28.09 (C-15), 27.19 (C-23), 27.12 (C-2), 25.71 (C-27), 23.91 (C-16), 23.57 (C-30), 23.40 (C-11), 18.13 (C-6), 16.47 (C-24), 15.65 (C-25), 15.24 (C-26). HRMS (ESI; *m/z*). Calcd for $C_{41}H_{59}N_6O_2$, $[M + H]^+$, 667.4694; found, 667.4695.

4.14.

(2R,3R,4S,5R,6R)-2-(acetoxymethyl)-6-(4-((N-(oleanoloyl))aminomethyl)-1H-1,2,3-triazol-1-yl)tetrahydro-2H-pyran-3,4,5-triyl triacetate (**4n**).

White solid, yield 83%. m.p. 131.1–132.7 °C (lit. 129.8–131.7 °C [3]). ^1H NMR spectrum was similar to the reported data [3].

4.15.

N-((1-((2R,3R,4S,5S,6R)-3,4,5-trihydroxy-6-(hydroxymethyl)tetrahydro-2H-pyran-2-yl)-1H-1,2,3-triazol-4-yl)methyl) oleanolamide (**4o**)

White solid, yield 89%. m.p. 188.1–190.4 °C (lit. 190.0–191.8 °C [3]). ^1H NMR spectrum was similar to the reported data [3].

5. The synthesis of methyl oleanolate (**5**)

The intermediate **5** was synthesized according to the reported method [5]. White solid, yield 96%; m.p. 197.0–199.5 °C (lit. 198–200 °C [6]). ^1H NMR data spectrum was similar to the reported data [5].

6. The synthesis of 3-Oxoolean-12-en-28-oic acid methyl ester (**6**)

The intermediate **5** was synthesized according to the reported method [7]. White solid, yield 96%; m.p. 184.7–

186.0 °C (lit. 183–186 °C [8]). ^{1}H NMR spectrum was similar to the reported data [7].

7. 12α,13α-epoxy-3,4-seco-olean-3,28-dioic acid, 3,4-lactone ,28-methyl ester (**7**)

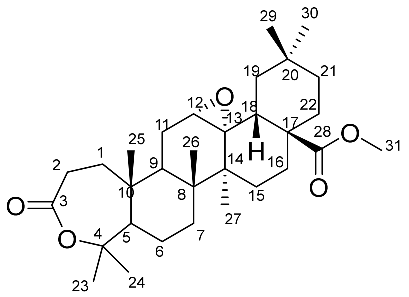

To a solution of **6** (0.10 g, 0.21 mmol) in CH_2Cl_2 (5 mL), 3-chloroperbenzoic acid (0.09 g, 0.53 mmol) and $NaHCO_3$ (0.03 g, 0.34 mmol) were added. The reaction mixture was then stirred at room temperature for 12 hours, and then filtered. The filtrate was evaporated under reduced pressure and purified by using silica gel column chromatography with EtOAc/hexanes (1/20) as eluent to afford **7** as white solid in yield 55%. m.p. 178.0–179.2 °C. IR (KBr): v = 3081, 1734, 1716, 1521, 1457, 757 cm^{-1}. ^{1}H NMR ($CDCl_3$) δ 3.69 (s, 3H, H-31), 3.19 (s, 1H, H-12), 2.72 – 2.55 (m, 1H, H-2), 2.54 – 2.38 (m, 1H, H-2'), 2.01 – 1.20 (m, 21H, other aliphatic ring protons), 1.45 (s, 3H, CH_3), 1.37 (s, 3H, CH_3), 1.15 (s, 3H, CH_3), 1.11 (s, 3H, CH_3), 0.93 (s, 3H, CH_3), 0.83 (s, 3H, CH_3), 0.80 (s, 3H, CH_3). ^{13}C NMR (126 MHz, $CDCl_3$) δ 178.32 (C-28), 175.23 (C-3), 85.86 (C-4), 67.09 (C-12), 63.55 (C-13), 53.06 (C-3), 51.80 (C-31), 47.82 (C-14), 44.21 (C-9), 40.87 (C-18), 40.41 (C-17), 39.80 (C-7), 39.19 (C-10), 38.86 (C-8), 38.02 (C-19), 34.02 (C-21), 33.19 (C-29), 32.85 (C-2), 32.42 (C-1), 32.38 (C-23), 31.20 (C-6), 30.38 (C-20), 29.15 (C-15), 26.59 (C-24), 24.65 (C-11), 23.34 (C-30), 23.04 (C-22), 22.54 (C-16), 22.41 (C-27), 19.74 (C-25), 19.69 (C-26). ESI-MS (*m/z*) 501.583 $[M + H]^+$. HRMS (ESI; *m/z*). Calcd for $C_{31}H_{48}O_5Na$, $[M + Na]^+$, 523.3394; found, 523.3417.

8. General Procedure for the preparation of **8a**-**8h**.

To a cooled solution of $AlCl_3$ (0.03 g, 0.22 mmol) in anhydrous CH_2Cl_2 (5 mL), triethylamine (0.03 g, 0.30 mmol) was slowly added. After being stirred for 15 min, the reaction mixture was added with **7** (0.05 g, 0.10 mmol) solution in CH_2CH_2 and alkyl amine (0.12 mmol). The mixture was stirred for 1 hours and quenched through the addition of ice water (10 mL) and saturated sodium bicarbonate solution (5 mL). The mixture was extracted with CH_2Cl_2 (3 × 20 mL). The combined organic layer was washed with brine, dried over Na_2SO_4, and concentrated.

The residue was purified by using column chromatography on silica gel using $CH_2Cl_2/MeOH/NH_3\cdot H_2O$ (50:1:0.1−20:1:0.1) as eluent to mainly afford the target compound.

8.1. 13β-4-hydroxy-12-oxo-3,4-seco-olean-3,28-dioic acid, 3-(3-(dimethylamino)propyl)amide,28-methyl ester (**8a**).

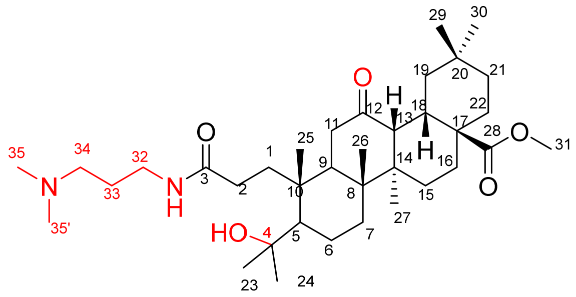

Light yellow solid, yield 55%. m.p. 122.2–123.8 °C. IR (KBr): ν = 3310, 2944, 1722, 1697, 1644, 1099 cm^{-1}. ^{1}H NMR ($CDCl_3$) δ 7.09 (s, 1H, NH), 3.66 (s, 3H, H-31), 3.33 – 3.07 (m, 2H, H-32), 2.79 (d, J = 13.5 Hz, 1H, H-18), 2.66 – 2.60 (m, 1H, H-13), 2.60 – 2.49 (m, 1H, H-1a), 2.33 (d, J = 5.6 Hz, 2H, H-34), 2.21 (s, 6H, H-35, H-35'), 2.15 – 1.20 (m, 23H, protons of other aliphatic ring and chain), 1.29 (s, 3H, CH_3), 1.21 (s, 3H, CH_3), 0.99 (s, 3H, CH_3), 0.96 (s, 6H, 2 × CH_3), 0.92 (s, 3H, CH_3), 0.89 (s, 3H, CH_3). ^{13}C NMR ($CDCl_3$) δ 211.78 (C-12), 178.39 (C-28), 174.56 (C-3), 75.48 (C-4), 58.18 (C-34), 51.86 (C-13), 51.77 (C-5), 50.72 (C-31), 47.32 (C-9), 45.37 (C-35, C-35'), 42.20 (C-17), 41.50 (C-14), 41.34 (C-8), 40.96 (C-32), 39.00 (C-10), 38.10 (C-19), 36.27 (C-11), 34.46 (C-1), 34.38 (C-18), 34.24 (C-7), 33.40 (C-21), 32.91 (C-2), 31.95 (C-29), 30.97 (C-30), 30.87 (C-16), 30.64 (C-22), 27.62 (C-20), 26.64 (C-33), 26.16 (C-15), 23.17 (C-24), 22.86 (C-23), 22.79 (C-6), 20.20 (C-27), 19.46 (C-26), 15.67 (C-25). HRMS (ESI; *m/z*). Calcd for $C_{36}H_{63}N_2O_5$, $[M + H]^+$, 603.4731; found, 603.4732.

8.2. 13β-4-hydroxy-12-oxo-3,4-seco-olean-3,28-dioic acid, 3-(3- morpholinopropyl)amide,28-methyl ester (**8b**).

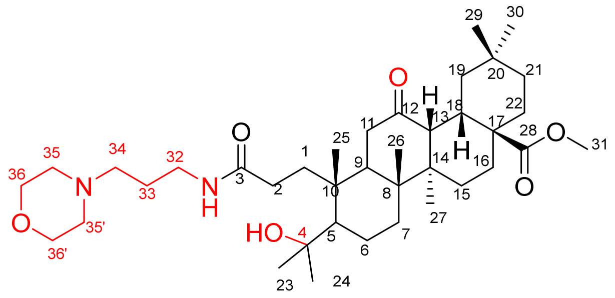

Light yellow solid, yield 40%. m.p. 108.9–110.1 °C. IR (KBr): ν = 3336, 2943, 1722, 1693, 1643, 1238, 1192,

1163, 691 cm^{-1}. ^{1}H NMR ($CDCl_3$) δ 6.66 (s, 1H, NH), 3.71 (s, 4H, H-36, H-36'), 3.68 (s, 3H, H-31), 3.29 (d, J = 5.2 Hz, 2H, H-32), 2.80 (d, J = 12.8 Hz, 1H, H-18), 2.65 (s, 1H, H-13), 2.62 – 2.54 (m, 1H, H-1a), 2.50 – 2.36 (m, 6H, H-34, H-35, H-35'), 2.01 – 1.20 (m, 25H, protons of other aliphatic ring and chain), 1.31 (s, 3H, CH_3), 1.23 (s, 3H, CH_3), 1.01 (s, 3H, CH_3), 0.98 (s, 6H, 2 × CH_3), 0.94 (s, 3H, CH_3), 0.91 (s, 3H, CH_3). ^{13}C NMR ($CDCl_3$) δ 211.66 (C-12), 178.36 (C-28), 174.33 (C-3), 75.63 (C-4), 66.95 (C-36, C-36'), 57.13 (C-34), 53.70 (C-35, C-35'), 51.85 (C-13), 51.76 (C-5), 50.83 (C-31), 47.31(C-9), 42.26 (C-17), 41.63 (C-14), 41.33 (C-8), 40.99 (C-32), 38.69 (C-10), 38.18 (C-19), 36.28 (C-11), 34.45 (C-1), 34.38 (C-18), 34.16 (C-7), 33.39 (C-21), 32.90 (C-2), 31.96 (C-29), 30.98 (C-30), 30.91 (C-16), 30.64 (C-22), 27.63 (C-20), 26.75 (C-33), 25.42 (C-15), 23.17 (C-24), 22.83 (C-23), 22.79 (C-6), 20.18 (C-27), 19.48 (C-26), 15.68 (C-25). HRMS (ESI; *m/z*). Calcd for $C_{38}H_{65}N_2O_6$, [M + H]$^+$, 645.4837; found, 645.4844.

8.3. 13β-4-hydroxy-12-oxo-3,4-seco-olean-3,28-dioic acid, 3-(3-(1H-imidazol-1-yl)propyl)amide,28-methyl ester **(8c)**.

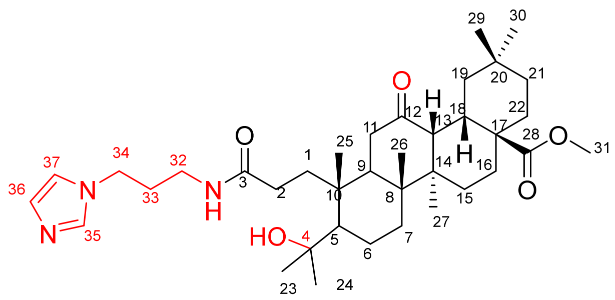

Light yellow solid, yield 47%. m.p. 120.1–121.7 °C. IR (KBr): ν = 3362, 2944, 1722, 1693, 1646, 1238, 1192, 1163, 692 cm^{-1}. ^{1}H NMR ($CDCl_3$) δ 7.47 (s, 1H, H-35), 7.04 (s, 1H, H-37), 6.94 (s, 1H, H-36), 6.53 (d, J = 5.5 Hz, 1H, NH), 3.97 (t, J = 6.8 Hz, 2H, H-34), 3.69 (s, 3H, H-31), 3.19 (dd, J = 12.5, 6.3 Hz, 2H, H-32), 2.80 (d, J = 13.3 Hz, 1H, H-18), 2.66 (d, J = 4.0 Hz, 1H, H-13), 2.56 – 2.40 (m, 1H, H-1a), 2.01 – 1.20 (m, 25H, protons of other aliphatic ring and chain), 1.33 (s, 3H, CH_3), 1.26 (s, 3H, CH_3), 1.01 (s, 3H, CH_3), 0.98 (s, 6H, 2 × CH_3), 0.93 (s, 3H, CH_3), 0.91 (s, 3H, CH_3). ^{13}C NMR ($CDCl_3$) δ 211.82 (C-12), 178.33 (C-28), 175.04 (C-3), 129.30 (C-36), 75.82 (C-4), 51.82 (C-34), 51.75 (C-13), 51.08 (C-5), 47.31 (C-9), 44.73, 42.25 (C-17), 41.75 (C-14), 41.32 (C-8), 41.00 (C-32), 38.06 (C-10), 36.68 (C-19), 36.30 (C-11), 34.83 (C-1), 34.50 (C-18), 34.44 (C-7), 33.40 (C-21), 32.90 (C-2), 31.98 (C-29), 31.01 (C-33), 30.97 (C-30), 30.93 (C-16), 30.62 (C-22), 27.64 (C-20), 26.77 (C-15),

23.18 (C-24), 22.79 (C-23), 22.70 (C-6), 20.19 (C-27), 19.59 (C-26), 15.75 (C-25). ESI-MS (*m/z*) 626 $[M + H]^+$. HRMS (ESI; *m/z*). Calcd for $C_{37}H_{59}N_3O_5Na$, $[M + Na]^+$, 648.4347; found, 648.4337.

8.4. 13β-4-hydroxy-12-oxo-3,4-seco-olean-3,28-dioic acid, 3-(2-(thiophen-2-yl)ethyl)amide,28-methyl ester **(8d).**

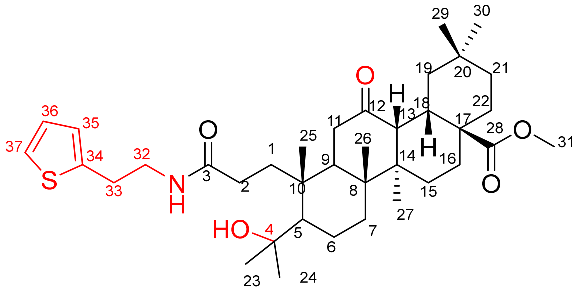

Light yellow solid, yield 55%. m.p. 120.0–121.5 °C. IR (KBr): ν = 3420, 2939, 1636, 1522, 1116, 997 cm^{-1}. ^1H NMR ($CDCl_3$) δ 7.20 – 7.15 (m, 1H, H-37), 6.95 (dd, *J* = 5.0, 3.5 Hz, 1H, H-36), 6.83 (d, *J* = 2.8 Hz, 1H, H-35), 6.20 (t, *J* = 5.5 Hz, 1H, NH), 3.67 (s, 3H, H-31), 3.62 – 3.42 (m, 2H, H-32), 3.01 (t, *J* = 6.2 Hz, 2H, H-33), 2.80 (d, *J* = 13.5 Hz, 1H, H-18), 2.63 (d, *J* = 4.1 Hz, 1H, H-13), 2.53 – 2.40 (m, 1H, H-1a), 2.30 – 1.20 (m, 23H, protons of other aliphatic ring and chain), 1.26 (s, 3H, CH_3), 1.19 (s, 3H, CH_3), 0.98 (s, 3H, CH_3), 0.97 (s, 3H, CH_3), 0.96 (s, 3H, CH_3), 0.90 (s, 6H, 2 × CH_3). ^{13}C NMR ($CDCl_3$) δ 211.63 (C-12), 178.33 (C-28), 174.65 (C-3), 141.66 (C-34), 127.01 (C-35), 125.41 (C-36), 123.93 (C-37), 75.73 (C-4), 51.83 (C-13), 51.73 (C-5), 50.66 (C-31), 47.29 (C-9), 42.15 (C-17), 41.57 (C-14), 41.30 (C-32), 40.94 (C-8), 40.67 (C-10), 38.01 (C-19), 36.25 (C-11), 34.54 (C-1), 34.44 (C-18, C-7), 33.41 (C-21), 32.90 (C-2), 31.94 (C-33), 30.95 (C-29), 30.90 (C-30), 30.63 (C-16), 29.81 (C-22), 27.61 (C-20), 26.72 (C-15), 23.17 (C-24), 22.78 (C-23), 22.75 (C-6), 20.21 (C-27), 19.49(C-26), 15.69 (C-25). ESI-MS (*m/z*) 628 $[M + H]^+$ HRMS (ESI; *m/z*). Calcd for $C_{37}H_{57}NO_5SNa$, $[M + Na]^+$, 650.3850; found, 650.3869.

8.5. 13β-4-hydroxy-12-oxo-3,4-seco-olean-3,28-dioic acid, 3-(4-fluorophenyl)amide,28-methyl ester (**8e**).

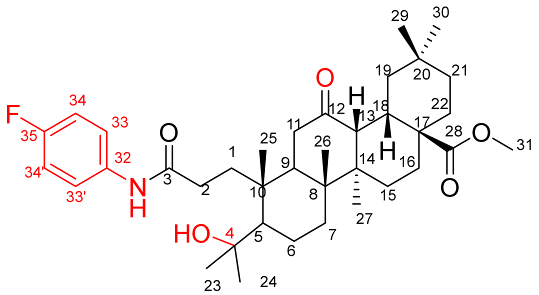

Light yellow solid, yield 56%. m.p. 146.6–148.1 °C. IR (KBr): ν = 3335, 2944, 1724, 1508, 1191, 1161, 832, 516 cm^{-1}. ^{1}H NMR ($CDCl_3$) δ 8.49 (s, 1H, NH), 7.46 (dd, J = 8.3, 4.8 Hz, 2H, H-33, H-33'), 6.95 (t, J = 8.5 Hz, 2H, H-34, H-34'), 3.68 (s, 3H, H-31), 3.23 (br s, 1H, OH), 2.80 (d, J = 12.8 Hz, 1H, H-18), 2.66 (s, 1H, H-13), 2.60 – 2.44 (m, 2H, H-1), 2.50 – 1.20 (m, 20H, protons of other aliphatic ring and chain), 1.35 (s, 3H, CH_3), 1.27 (s, 3H, CH_3), 1.00 (s, 3H, CH_3), 0.96 (s, 6H, 2 × CH_3), 0.93 (s, 3H, CH_3), 0.91 (s, 3H, CH_3). ^{13}C NMR ($CDCl_3$) δ 212.22 (C-12), 178.34 (C-28), 173.08 (C-3), 159.06 (d, J = 242.7 Hz, C-35), 134.41 (d, J = 2.5 Hz, C-32), 121.41 (d, J = 7.8 Hz, C-33, C-33'), 115.40 (d, J = 22.4 Hz, C-34, C-34'), 76.30 (C-4), 51.87 (C-13), 51.81 (C-5), 50.96(C-31), 47.28 (C-9), 42.16 (C-17), 41.77 (C-14), 41.36 (C-8), 40.96 (C-10), 38.02 (C-19), 36.28 (C-11), 34.79 (C-1), 34.67 (C-18), 34.41 (C-7), 33.46 (C-21), 32.88 (C-2), 32.00 (C-29), 31.98 (C-30), 30.97 (C-16), 30.64 (C-22), 27.60 (C-20), 26.79 (C-15), 23.19 (C-24), 22.75 (C-23), 22.69 (C-6), 20.26 (C-27), 19.56 (C-26), 15.76 (C-25). HRMS (ESI; *m/z*). Calcd for $C_{37}H_{53}NO_5F$, $[M - H]^-$, 610.3913; found, 610.3933.

8.6. 13β-4-hydroxy-12-oxo-3,4-seco-olean-3,28-dioic acid, 3-(4-methoxyphenyl)amide,28-methyl ester (**8f**).

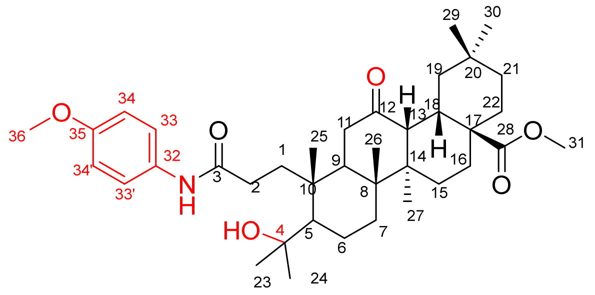

Light yellow solid, yield 61%. m.p. 138.8–140.3 °C. IR (KBr): ν = 3318, 2945, 1724, 1677, 1509, 1239, 1194, 1162 cm^{-1}. ^{1}H NMR ($CDCl_3$) δ 7.99 (s, 1H, NH), 7.39 (d, J = 6.5 Hz, 2H, H-33, H-33'), 6.82 (d, J = 6.5 Hz, 2H, H-34, H-34'), 3.77 (s, 3H, H-36), 3.68 (s, 3H, H-31), 2.92 (s, 1H, OH), 2.81 (d, J = 12.6 Hz, 1H, H-18), 2.66 (s, 1H, H-13), 2.51 (dd, J = 61.8, 11.4 Hz, 2H, H-1), 2.32 – 1.20 (m, 20H, protons of other aliphatic ring and chain), 1.34 (s, 3H, CH_3), 1.27 (s, 3H, CH_3), 1.01 (s, 3H, CH_3), 0.98 (s, 6H, 2 × CH_3), 0.95 (s, 3H, CH_3), 0.92 (s, 3H, CH_3). ^{13}C NMR ($CDCl_3$) δ 211.96 (C-12), 178.38 (C-28), 172.69 (C-3), 156.17 (C-35), 131.40 (C-32), 121.50 (C-33, C-33'), 114.07 (C-34, C-34'), 76.22 (C-4), 55.48 (C-36), 51.87 (C-13), 51.81 (C-5), 50.90 (C-31), 47.32 (C-9), 42.22 (C-17), 41.78 (C-14), 41.39 (C-8), 41.00 (C-10), 38.09 (C-19), 36.28 (C-11), 34.70 (C-1), 34.67 (C-18), 34.46 (C-7), 33.44 (C-21), 32.91 (C-2), 32.02 (C-29), 31.98 (C-30), 30.99 (C-16), 30.65 (C-22), 27.63

(C-20), 26.85 (C-15), 23.19 (C-24), 22.86 (C-23), 22.78 (C-6), 20.26 (C-27), 19.56 (C-26), 15.76 (C-25). HRMS (ESI; *m/z*). Calcd for $C_{38}H_{56}NO_6$, $[M - H]^-$, 622.4113; found, 622.4084.

8.7. 13β-4-hydroxy-12-oxo-3,4-seco-olean-3,28-dioic acid, 3-(4-(trifluoromethoxy)phenyl)amide,28-methyl ester (**8g**).

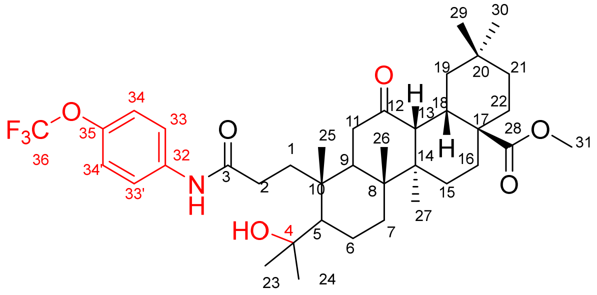

Light yellow solid, yield 65%. m.p. 135.6–137.1 °C. IR (KBr): ν = 3325, 2945, 1724, 1677, 1508, 1239, 1195, 1162 cm^{-1}. ^1H NMR ($CDCl_3$) δ 8.55 (d, J = 6.6 Hz, 1H, NH), 7.53 (d, J = 8.8 Hz, 2H, H-33, H-33'), 7.11 (d, J = 8.5 Hz, 2H, H-34, H-34'), 3.68 (s, 3H, H-31), 2.98 (s, 1H, OH), 2.81 (d, J = 13.4 Hz, 1H, H-18), 2.66 (d, J = 3.9 Hz, 1H, H-13), 2.59 – 2.43 (m, 2H, H-1), 2.31 – 1.41 (m, 19H, protons of other aliphatic ring and chain), 1.36 (s, 3H, CH_3), 1.28 (s, 3H, CH_3), 1.00 (s, 3H, CH_3), 0.96 (s, 6H, 2 × CH_3), 0.92 (s, 3H, CH_3), 0.91 (s, 3H, CH_3). ^{13}C NMR ($CDCl_3$) δ 212.14 (C-12), 178.36 (C-28), 173.20 (C-3), 144.88 (C-35), 137.12 (C-32), 121.61 (C-33, C-33'), 120.69 (C-34, C-34'), 119.48 (q, J = 5.04 Hz, C-36), 76.50 (C-4), 51.87 (C-13), 51.83 (C-5), 50.98 (C-31), 47.29 (C-9), 42.14 (C-17), 41.80 (C-14), 41.38 (C-8), 40.97 (C-10), 38.00 (C-19), 36.28 (C-11), 34.87 (C-1), 34.78 (C-18), 34.41 (C-7), 33.44 (C-21), 32.88 (C-2), 32.16 (C-29), 31.98 (C-30), 31.00 (C-16), 30.64 (C-22), 27.61 (C-20), 26.84 (C-15), 23.18 (C-24), 22.75 (C-23), 22.67 (C-6), 20.25 (C-27), 19.58 (C-26), 15.77 (C-25). HRMS (ESI; *m/z*). Calcd for $C_{38}H_{53}NO_6F_3$, $[M - H]^-$, 676.3830; found, 676.3843.

8.8. 13β-3-(4-(2,4-difluorophenyl)piperazin-1-yl)-4-hydroxy-12-oxo-3,4-seco-olean-3-one-28-oic acid methyl ester (**8h**).

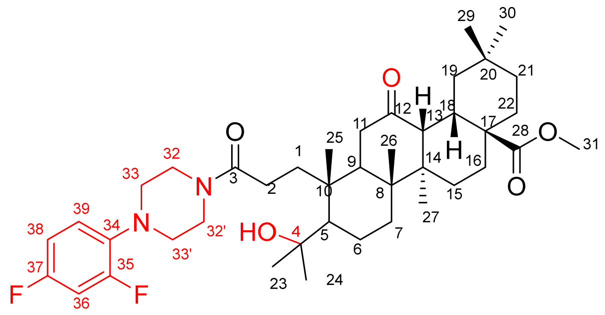

Light yellow solid, yield 66%. m.p. 117.5–118.9 °C. IR (KBr): ν = 3324, 2944, 1724, 1677, 1508, 1456, 1194, 1163, 700 cm^{-1}. ^1H NMR ($CDCl_3$) δ 6.96 – 6.68 (m, 3H, H-36, H-38, H-39), 3.88 –3.78 (m, 2H, H-32), 3.69 (s, 3H, H-31), 3.65 –3.57 (m, 2H, H-32'), 3.48 (s, 1H, OH), 3.11 – 2.90 (m, 4H, H-33, H-33'), 2.98 (s, 1H, H-18), 2.81 (d, J = 13.5 Hz, 1H, H-13), 2.65 (s, 1H, H-1a), 2.49 – 1.41 (m, 26H, protons of other aliphatic ring and chain), 1.32 (s, 3H, CH_3), 1.22 (s, 3H, CH_3), 1.03 (s, 3H, CH_3), 0.99 (s, 3H, CH_3), 0.98 (s, 3H, CH_3), 0.95 (s, 3H, CH_3), 0.90 (s, 3H, CH_3). ^{13}C NMR ($CDCl_3$) δ 211.66 (C-12), 178.33 (C-28), 173.25 (C-3), 158.29 (dd, J = 243.9, 11.5 Hz, C-35), 155.76 (dd, J = 249.6, 11.6 Hz, C-37), 136.09 (dd, J = 8.9, 3.3 Hz, C-39), 119.94 (dd, J = 9.2, 3.7 Hz, C-34), 110.85 (dd, J = 21.4, 3.4 Hz, C-38), 104.85 (t, J = 25.4 Hz, C-36), 75.37 (C-4), 51.85 (C-13), 51.75 (C-5), 51.52 (C-33), 50.81 (C-31), 50.68 (C-33'), 47.30(C-9), 46.25 (C-32), 42.26 (C-17), 41.87 (C-32'), 41.59 (C-14), 41.48 (C-8), 41.02 (C-10), 38.43 (C-19), 36.33 (C-11), 34.43 (C-1), 34.32 (C-18), 33.71 (C-7), 33.39 (C-21), 32.89 (C-2), 31.97 (C-29), 30.83 (C-30), 30.65 (C-16), 27.64 (C-22), 26.91 (C-20), 26.42 (C-15), 23.18 (C-24), 22.99 (C-23), 22.78 (C-6), 20.17 (C-27), 19.36 (C-26), 15.63 (C-25). HRMS (ESI; *m/z*). Calcd for $C_{41}H_{60}N_2O_5F_6Na$, $[M + Na]^+$, 721.4363; found, 721.4346.

9. Methyl 2-Carboxy-3-oxooleana-1,12-dien-28-oate (**9**).

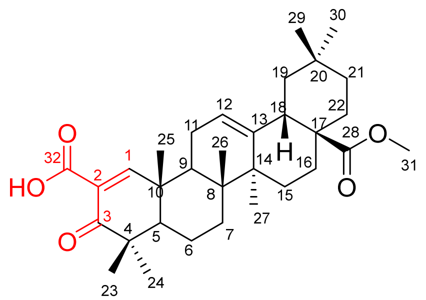

The intermediate **9** was synthesized according to the reported method [9]. White solid, yield 69%. m.p. 225.8–226.1 °C (lit: 230–231 °C [9]). ^1H NMR were consistent with reported data [9].

10. General Procedure for the preparation of **10a**–**10d**.

To a solution of **9** (0.30 g, 0.59 mmol) in DMF (5 mL), 2-(7-azabenzotriazol-1-yl)-N,N,N',N'-tetramethyluronium hexafluorophosphate (0.34 g, 0.88 mmol), N,N-diisopropylethylamine (0.23 g, 1.76 mmol) and corresponding alkyl amine (0.70 mmol) were added. The mixture was stirred for 0.5 hour at room temperature. The reaction mixture was added with water (15 mL) and was extracted with ethyl acetate (15 mL). The combined organic layer was washed with brine for three times, dried over anhydrous sodium sulfate, filtered, and concentrated. The residue was purified by using column chromatography on silica gel to give the target compound.

10.1. Methyl 2-(2-(diethylamino)ethyl)carbamoyl-3-oxooleana-1,12-dien-28-oate (**10a**).

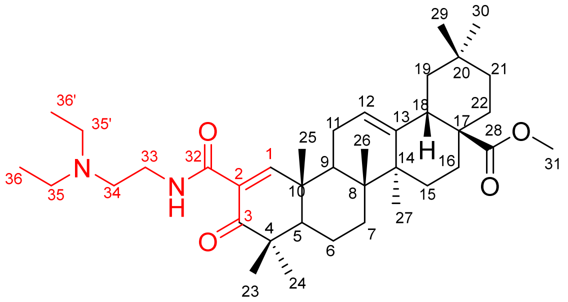

Light yellow solid, yield 90%. m.p. 134.1–136.5 °C. IR (KBr): v = 3323, 2944, 1724, 1674, 1524, 1461, 841, 730, 557 cm^{-1}. ^{1}H NMR ($CDCl_3$) δ 9.04 (s, 1H, NH), 8.22 (s, 1H, H-1), 5.37 (t, *J* = 3.2 Hz, 1H, H-12), 3.63 (s, 3H, H-31), 3.52 (d, *J* = 5.6 Hz, 2H, H-33), 3.00 – 2.73 (m, 7H, H-18, H-34, H-35, H-35'), 2.49 – 1.41 (m, 23H, protons of other aliphatic ring and chain), 1.18 (s, 3H, CH_3), 1.18 – 1.15 (m, 9H, 3 × CH_3), 1.14 (s, 6H, 2 × CH_3), 0.94 (s, 3H, CH_3), 0.91 (s, 3H, CH_3), 0.84 (s, 3H, CH_3). ^{13}C NMR ($CDCl_3$) δ 205.73 (C-3), 178.24 (C-28), 168.23 (C-32), 165.38 (C-1), 144.20 (C-13), 126.53 (C-2), 121.60 (C-12), 52.69 (C-34), 52.00 (C-5), 51.61 (C-31), 47.57 (C-35, C-35'), 46.83 (C-17), 45.82 (C-19), 45.59 (C-4), 42.13 (C-14), 41.57 (C-18), 41.10 (C-9), 40.25(C-1), 39.39 (C-8), 37.33 (C-33), 33.88 (C-21), 33.12 (C-7), 32.28 (C-22), 32.14 (C-20), 30.72 (C-24), 28.85 (C-15), 27.64 (C-29), 25.79 (C-27), 23.58 (C-30), 23.38 (C-11), 23.01 (C-16), 21.74 (C-23), 19.17 (C-6), 18.39 (C-26), 17.43 (C-25), 10.94 (C-36, C-36'). HRMS (ESI; *m/z*). Calcd for $C_{38}H_{61}N_2O_4$, $[M + H]^+$, 609.4626; found, 609.4624.

10.2 Ethyl 2-(3-(pyrrolidin-1-yl)propyl)carbamoyl-3-oxooleana-1,12-dien-28-oate (**10b**)

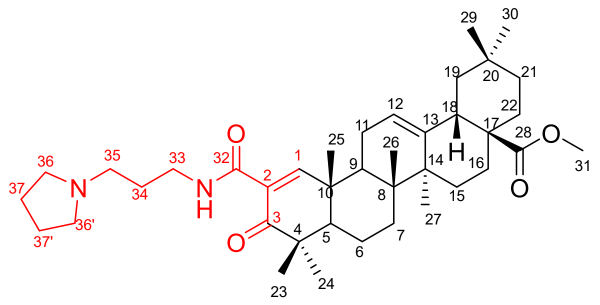

Light yellow solid, yield 89%. m.p. 134.0–136.0 °C. IR (KBr): v = 2946, 1721, 1669, 1536, 834, 729, 556 cm^{-1}. ^{1}H NMR ($CDCl_3$) δ 9.14 (t, J = 6.3 Hz, 1H, NH), 8.15 (s, 1H, H-1), 5.36 (s, 1H, H-12), 3.61 (s, 3H, H-31), 3.48 – 3.40 (m, 2H, H-33), 3.40 – 3.20 (m, 3H, H-36, H-18), 3.18 (t, J = 6.4 Hz, 2H, H-36'), 2.78 (s, 2H, H-35), 2.42 – 1.20 (m, 24H, protons of other aliphatic ring and chain), 1.18 (s, 3H, CH_3), 1.18 (s, 3H, CH_3), 1.16 (s, 6H, 2 × CH_3), 0.94 (s, 3H, CH_3), 0.91 (s, 3H, CH_3), 0.84 (s, 3H, CH_3). ^{13}C NMR ($CDCl_3$) δ 205.84 (C-3), 178.23 (C-28), 168.93 (C-32), 166.93 (C-1), 144.35 (C-13), 125.72 (C-2), 121.47 (C-12), 53.93 (C-36, C-36'), 51.84 (C-34), 51.78 (C-5), 51.63 (C-31), 46.80 (C-17), 45.81 (C-19), 45.65 (C-4), 42.14 (C-33), 41.53 (C-14), 41.04 (C-18), 40.31 (C-35), 41.10 (C-9), 39.58 (C-1), 38.61 (C-8), 35.68 (C-33), 33.82 (C-21), 33.10 (C-7), 32.25 (C-22), 32.08 (C-20), 30.70 (C-24), 28.64 (C-15), 27.62 (C-34), 26.31 (C-29), 25.80 (C-27), 23.60 (C-37, C-37'), 23.37 (C-30), 23.09 (C-11), 22.98 (C-16), 21.82 (C-23), 19.06 (C-6), 18.27 (C-26), 17.42 (C-25). HRMS (ESI; *m/z*). Calcd for $C_{39}H_{61}N_2O_4$, $[M + H]^+$, 621.4626; found, 621.4612.

10.3. Methyl 2-(3-phenylpropyl)carbamoyl-3-oxooleana-1,12-dien-28-oate (**10c**)

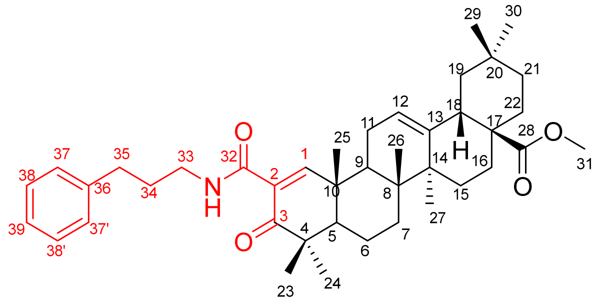

White solid, yield 87%. m.p. 135.3–137.0 °C. IR (KBr): v = 2944, 1725, 1676, 1524, 1163, 699 cm^{-1}. ^{1}H NMR ($CDCl_3$) *δ* 8.73 (s, 1H, NH), 8.24 (s, 1H, H-1), 7.27 (t, J = 7.1 Hz, 2H, H-37, H-37'), 7.18 (dd, J = 12.8, 7.3 Hz, 3H, H-38, H-38', H-39), 5.37 (s, 1H, H-12), 3.63 (s, 3H, H-31), 3.49 – 3.29 (m, 2H, H-33, H-33'), 2.89 (d, J = 12.2 Hz, 1H, H-18), 2.68 (t, J = 7.4 Hz, 2H, H-35), 2.42 – 1.20 (m, 20H, protons of other aliphatic ring and chain),

1.16 (s, 3H, CH_3), 1.15 (s, 3H, CH_3), 1.14 (s, 6H, 2 × CH_3), 0.94 (s, 3H, CH_3), 0.92 (s, 3H, CH_3), 0.83 (s, 3H, CH_3). ^{13}C NMR ($CDCl_3$) δ 206.32 (C-3), 178.23 (C-28), 167.91 (C-32), 163.85 (C-1), 144.08 (C-13), 141.49 (C-36), 128.40 (C-37, C-37', C-38, C-38'), 126.96 (C-39), 125.89 (C-2), 121.72 (C-12), 51.99 (C-5), 51.59 (C-31), 46.83 (C-17), 45.86 (C-19), 45.54 (C-4), 42.11 (C-14), 41.56 (C-18), 41.10 (C-9), 40.22 (C-1), 39.28 (C-8), 39.00 (C-33), 33.90 (C-21), 33.26 (C-35), 33.13 (C-7), 32.30 (C-22), 32.15 (C-20), 30.94 (C-34), 30.72 (C-24), 28.97 (C-15), 27.65 (C-29), 25.80 (C-27), 23.57 (C-30), 23.37 (C-11), 23.02 (C-16), 21.77 (C-23), 19.22 (C-6), 18.53 (C-26), 17.42 (C-25). HRMS (ESI; *m/z*). Calcd for $C_{41}H_{57}NO_4Na$, $[M + Na]^+$, 650.4180; found, 650.4163.

10.4. Methyl 2-(4-(trifluoromethyl)phenethyl)carbamoyl-3-oxooleana-1,12-dien-28-oate (**10d**).

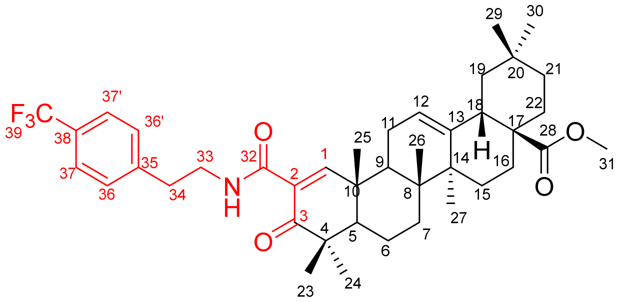

Light yellow solid, yield 95%. m.p. 138.6–140.1 °C. IR (KBr): ν = 2947, 1723, 1674, 1528, 1323, 1162, 1122, 1067, 730 cm^{-1}. ^{1}H NMR ($CDCl_3$) δ 8.76 (t, *J* = 5.6 Hz, 1H, NH), 8.23 (s, 1H, H-1), 7.55 (d, *J* = 8.1 Hz, 2H, H-37, H-37'), 7.33 (d, *J* = 8.0 Hz, 2H, H-36, H-36'), 5.37 (t, *J* = 3.2 Hz, 1H, H-12), 3.62 (d, *J* = 5.8 Hz, 3H, H-31), 3.61 – 3.55 (m, 2H, H-33), 2.99 – 2.84 (m, 3H, H-18, H-34), 2.38 – 1.34 (m, 19H, protons of other aliphatic ring and chain), 1.15 (s, 3H, CH_3), 1.14 (s, 3H, CH_3), 1.13 (s, 3H, CH_3), 1.12 (s, 3H, CH_3), 0.94 (s, 3H, CH_3), 0.92 (s, 3H, CH_3), 0.83 (s, 3H, CH_3). ^{13}C NMR ($CDCl_3$) δ 206.26 (C-3), 178.25 (C-28), 168.16 (C-32), 163.97 (C-1), 144.14 (C-13), 143.24 (C-35), 129.10 (C-36, C-36'), 128.91 (C-38), 128.59 (C-39), 126.81 (C-2), 125.47 (C-37), 125.43 (C-37'), 121.66 (C-12), 51.97 (C-5), 51.60 (C-31), 46.84 (C-17), 45.85 (C-19), 45.55 (C-4), 42.11 (C-14), 41.56 (C-18), 41.08 (C-9), 40.61 (C-1), 40.23 (C-33), 39.32 (C-8), 35.52 (C-34), 33.90 (C-21), 33.13 (C-7), 32.29 (C-22), 32.13 (C-20), 30.73 (C-24), 28.94 (C-15), 27.65 (C-29), 25.79 (C-27), 23.57 (C-30), 23.36 (C-11), 23.01 (C-16), 21.72 (C-23), 19.21 (C-6), 18.52 (C-26), 17.42 (C-25). HRMS (ESI; *m/z*). Calcd for $C_{41}H_{55}F_3NO_4$, $[M + H]^+$, 682.4078; found, 682.4069.

References

1. Jana, J.; Mondal, S.; Bhattacharjee, P.; Sengupta, P.; Roychowdhury, T.; Saha, P.; Kundu, P.; Chatterjee, S., Chelerythrine down regulates expression of VEGFA, BCL2 and KRAS by arresting G-Quadruplex structures at their promoter regions. *Sci. Rep.* **2017,** 7, 14.
2. Xiao, S.; Wang, Q.; Si, L.; Shi, Y.; Wang, H.; Yu, F.; Zhang, Y.; Li, Y.; Zheng, Y.; Zhang, C.; Wang, C.; Zhang, L.; Zhou, D., Synthesis and anti-HCV entry activity studies of β-cyclodextrin-pentacyclic triterpene conjugates. *ChemMedChem* **2014,** 9, (5), 1060–1070.
3. Su, Y. Q.; Meng, L. K.; Sun, J. Q.; Li, W. J.; Shao, L.; Chen, K. X.; Zhou, D. M.; Yang, F.; Yu, F., Design, synthesis of oleanolic acid-saccharide conjugates using click chemistry methodology and study of their anti-influenza activity. *Eur. J. Med. Chem.* **2019,** 182, 111–622.
4. Li, W.; Yang, F.; Meng, L.; Sun, J.; Su, Y.; Shao, L.; Zhou, D.; Yu, F., Synthesis, structure activity relationship and anti-influenza A virus evaluation of oleanolic acid-linear amino derivatives. *Chem. Pharm. Bull.* **2019,** 67, (11), 1201–1207.
5. Fu, L.; Gribble, G. W., Efficient and Scalable Synthesis of Bardoxolone Methyl (CDDO-methyl Ester). *Org. Lett.* **2013,** 15, (7), 1622–1625.
6. Loesche, A.; Köwitsch, A.; Lucas, S. D.; Al-Halabi, Z.; Sippl, W.; Al-Harrasi, A.; Csuk, R., Ursolic and oleanolic acid derivatives with cholinesterase inhibiting potential. *Bioorg. Chem.* **2019,** 85, 23–32.
7. Del Prete, D.; Taglialatela-Scafati, O.; Minassi, A.; Sirignano, C.; Cruz, C.; Bellido, M. L.; Munoz, E.; Appendino, G., Electrophilic Triterpenoid Enones: A Comparative Thiol-Trapping and Bioactivity Study. *J. Nat. Prod.* **2017,** 80, (8), 2276–2283.
8. Heller, L.; Schwarz, S.; Perl, V.; Koewitsch, A.; Siewert, B.; Csuk, R., Incorporation of a Michael acceptor enhances the antitumor activity of triterpenoic acids. *Eur. J. Med. Chem.* **2015,** 101, 391–399.
9. Honda, T.; Gribble, G. W.; Suh, N.; Finlay, H. J.; Rounds, B. V.; Bore, L.; Favaloro, F. G., Jr.; Wang, Y.; Sporn, M. B., Novel Synthetic Oleanane and Ursane Triterpenoids with Various Enone Functionalities in Ring A as Inhibitors of Nitric Oxide Production in Mouse Macrophages. *J. Med. Chem.* **2000,** 43, (9), 1866–1877.

^{1}H-NMR, ^{13}C-NMR, HRMS spectra of Synthetic Compounds

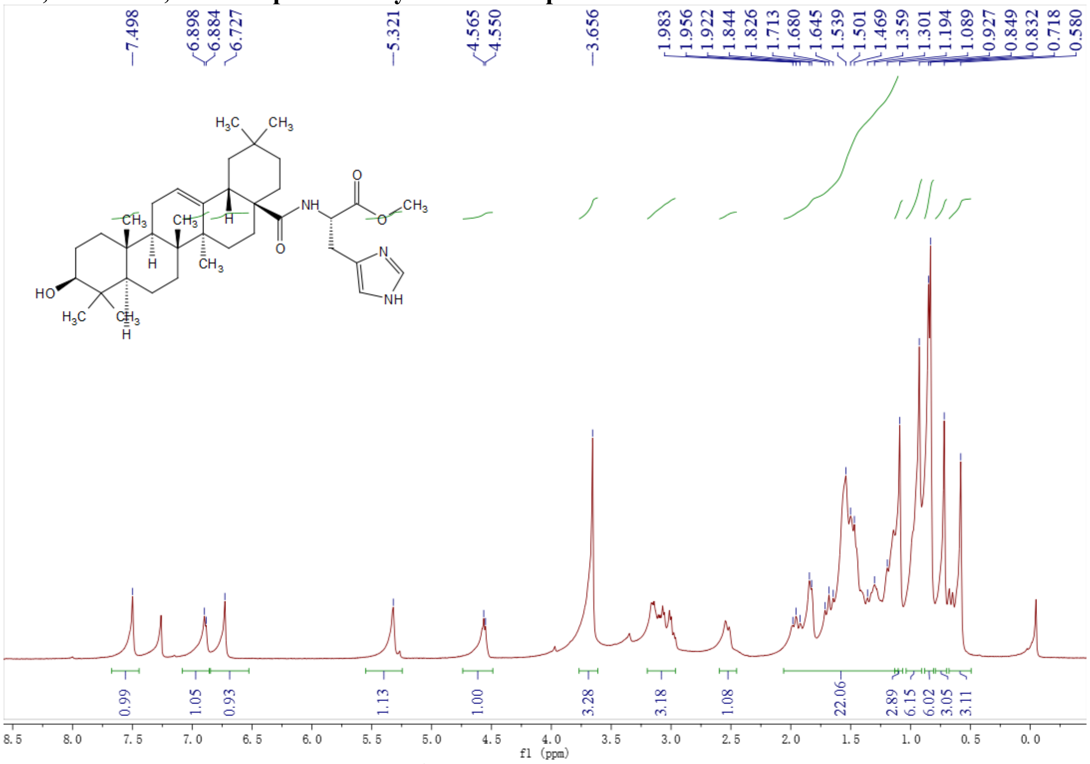

^{1}H spectrum of **OA-His**

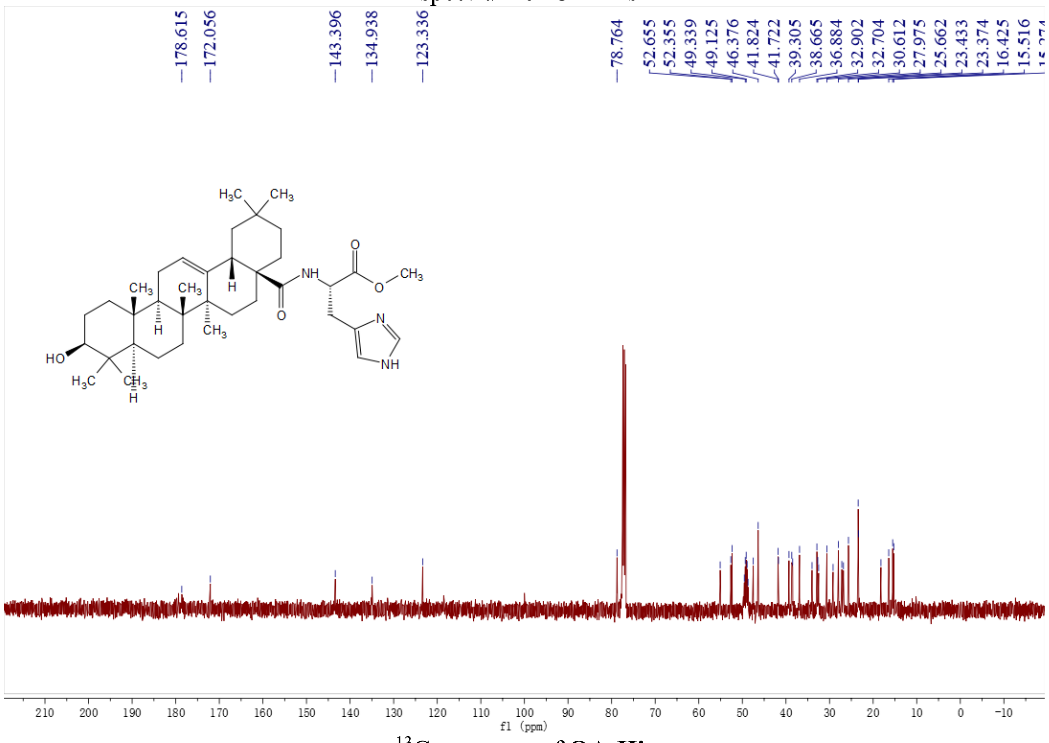

^{13}C spectrum of **OA-His**

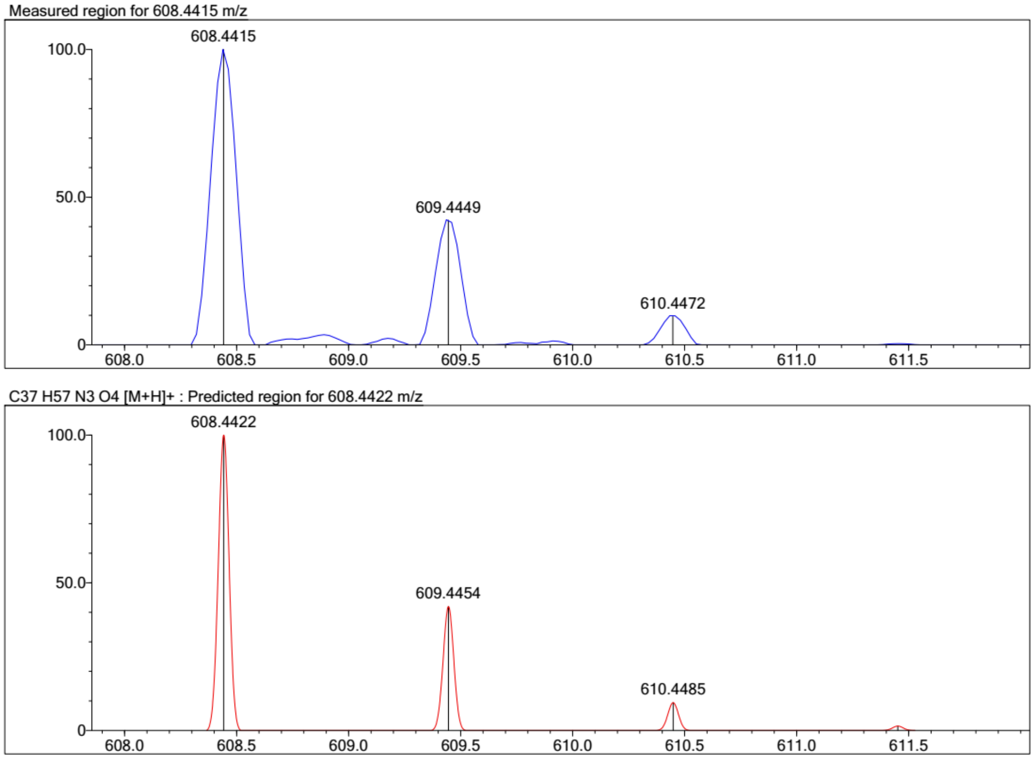

Rank	Score	Formula (M)	Ion	Meas. m/z	Pred. m/z	Df. (mDa)	Df. (ppm)	Iso	DBE
1	99.07	C37 H57 N3 O4	[M+H]+	608.4415	608.4422	-0.7	-1.15	99.44	11.0

HRMS spectrum of **OA-His**

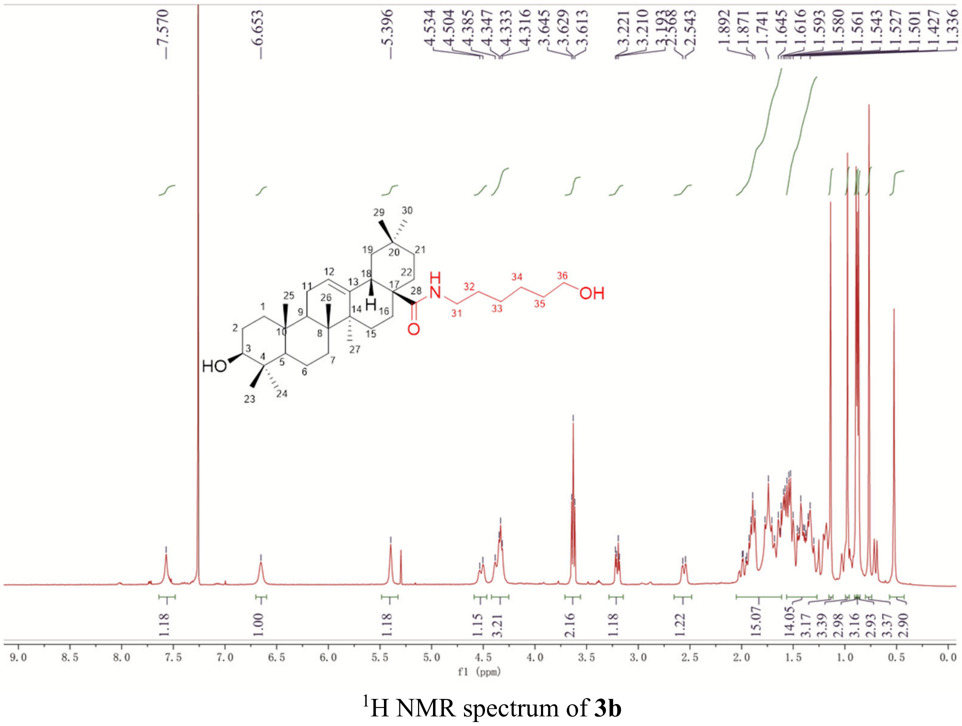

^{1}H NMR spectrum of **3b**

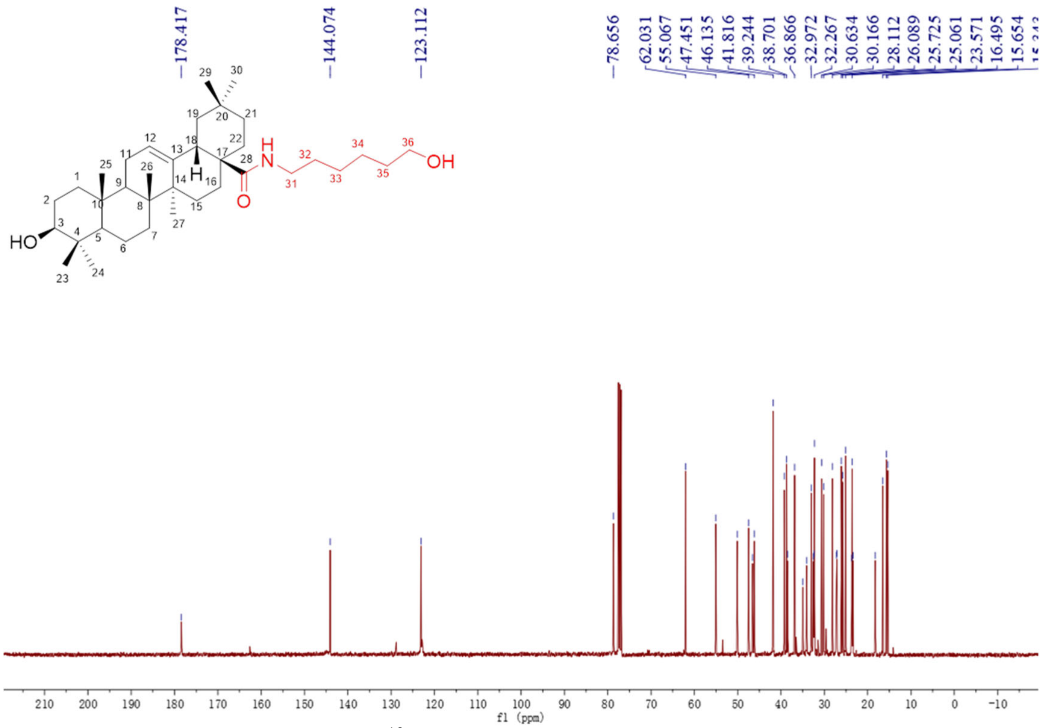

^{13}C NMR spectrum of **3b**

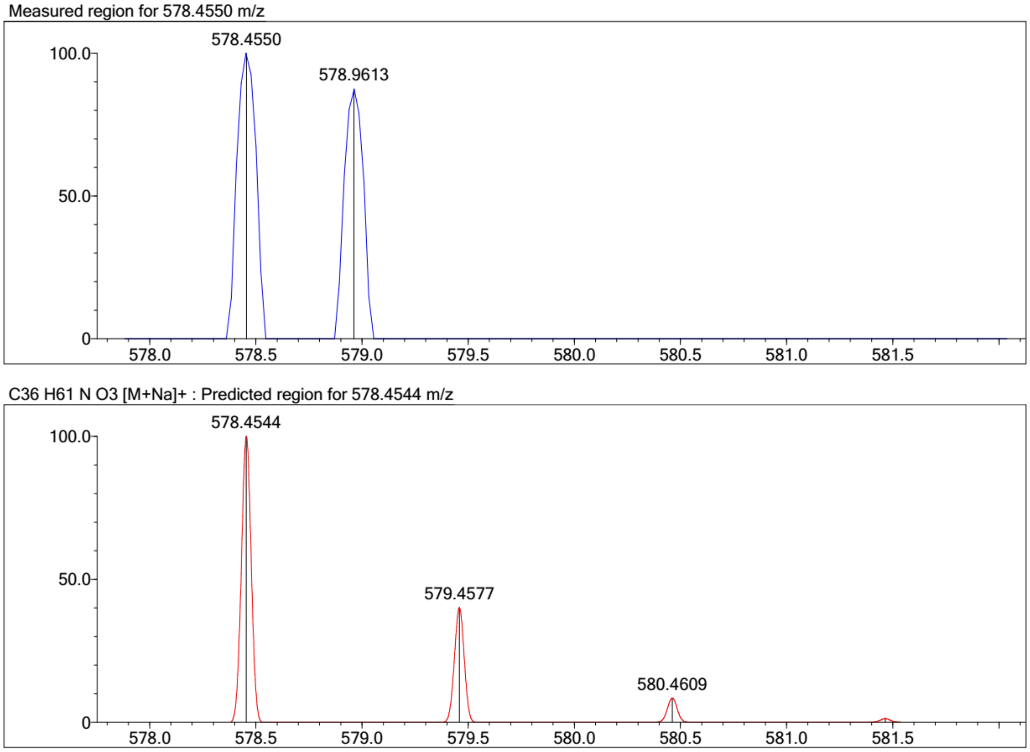

Rank	Score	Formula (M)	Ion	Meas. m/z	Pred. m/z	Df. (mDa)	Df. (ppm)	Iso	DBE
3	0.00	C36 H61 N O3	[M+Na]+	578.4550	578.4544	0.6	1.04	0.00	7.0

HRMS spectrum of **3b**

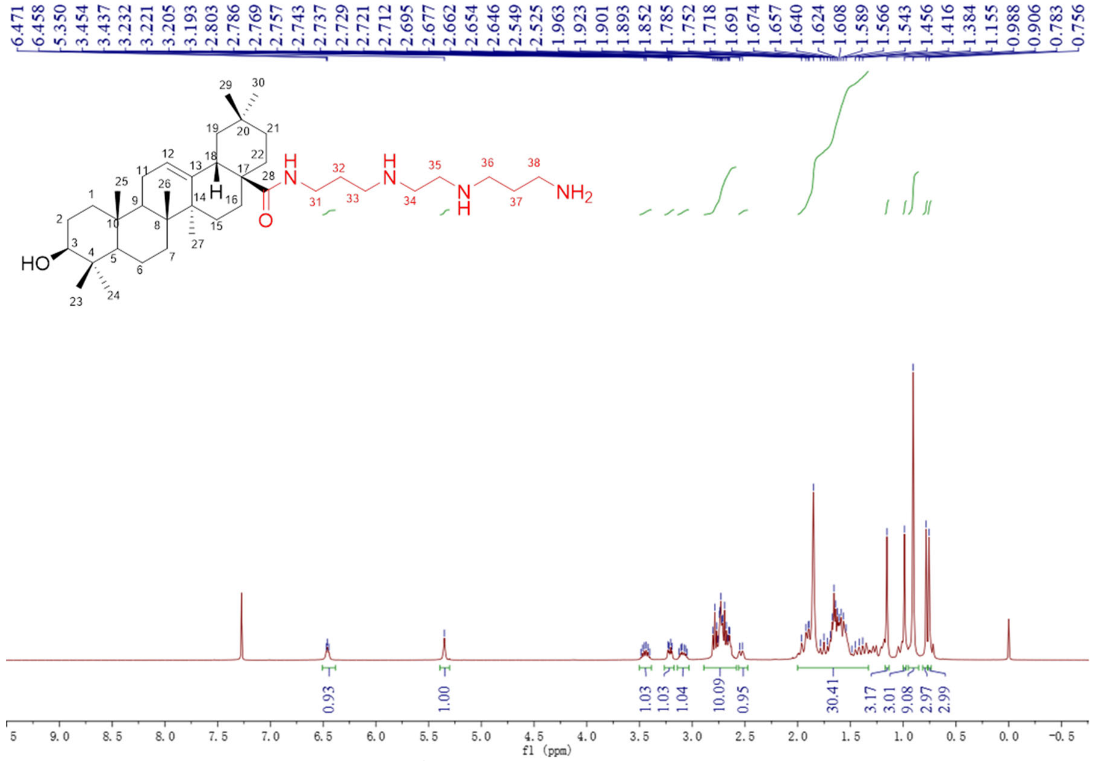

^{1}H NMR spectrum of **3c**

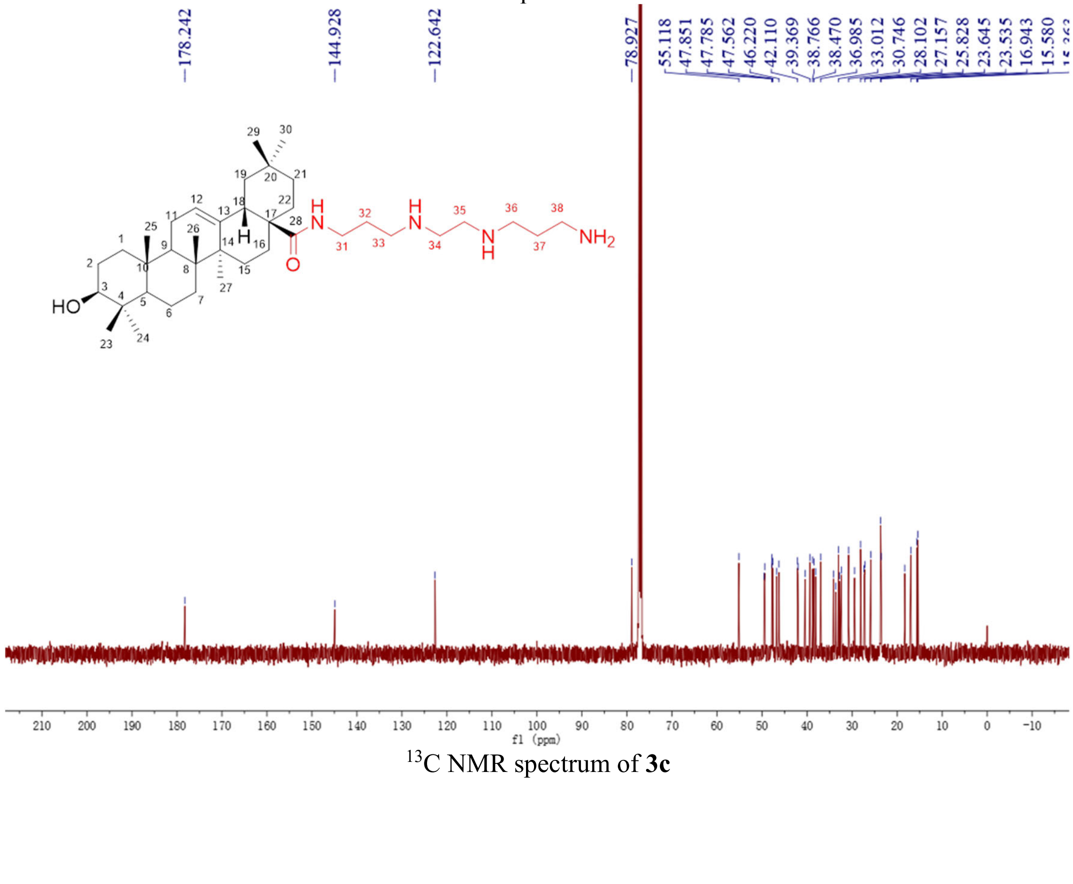

^{13}C NMR spectrum of **3c**

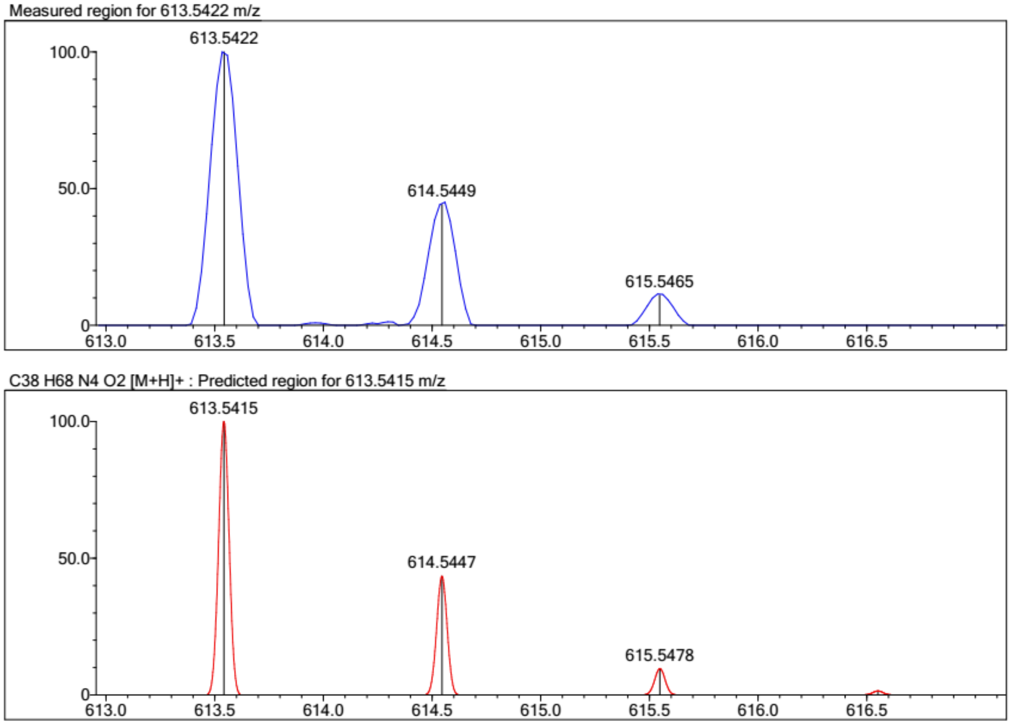

Rank	Score	Formula (M)	Ion	Meas. m/z	Pred. m/z	Df. (mDa)	Df. (ppm)	Iso	DBE
1	92.32	C38 H68 N4 O2	[M+H]+	613.5422	613.5415	0.7	1.14	92.64	7.0

HRMS spectrum of **3c**

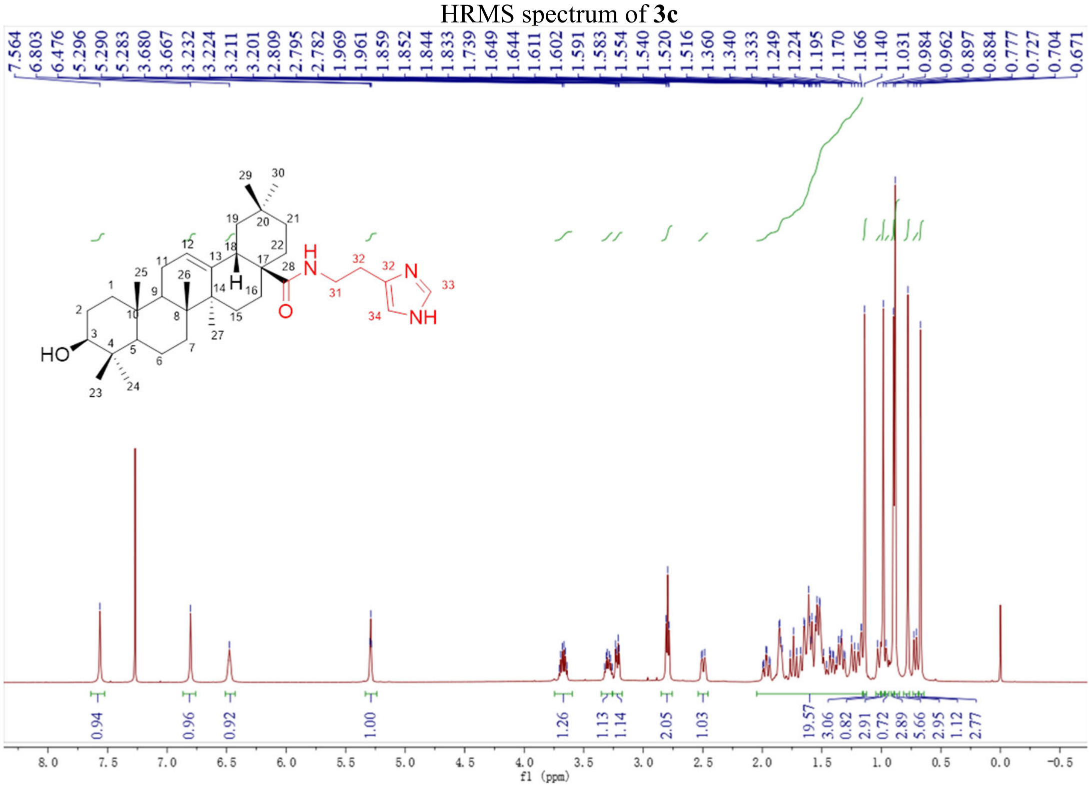

^{1}H spectrum of **3d**

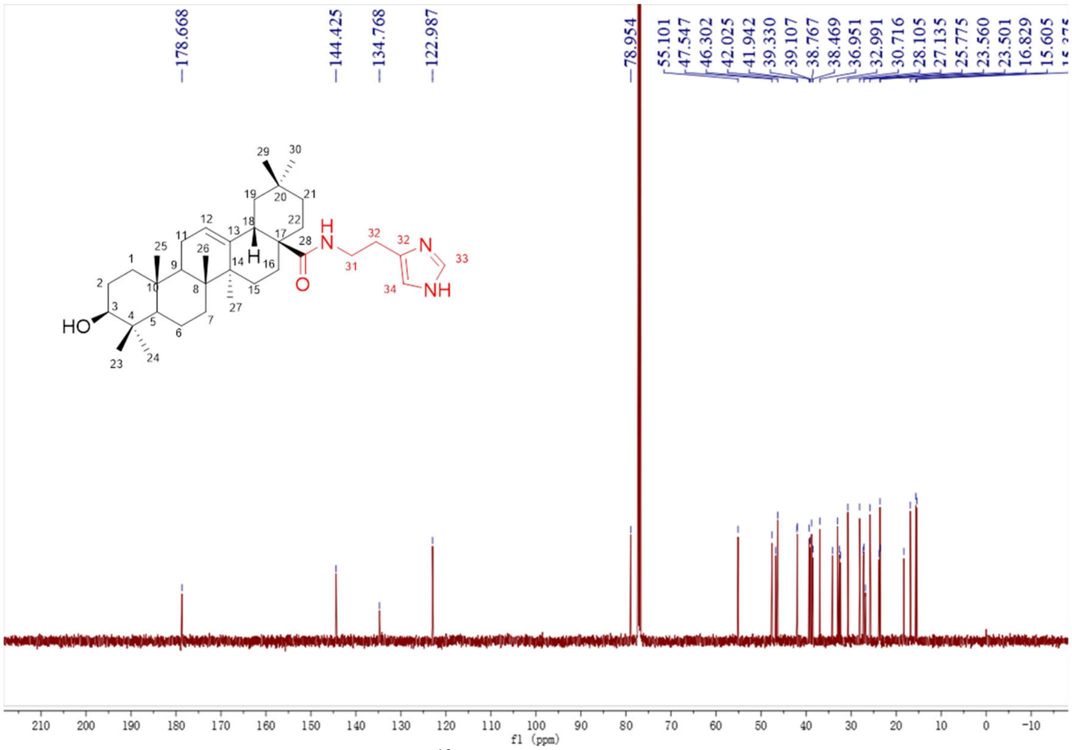

^{13}C spectrum of **3d**

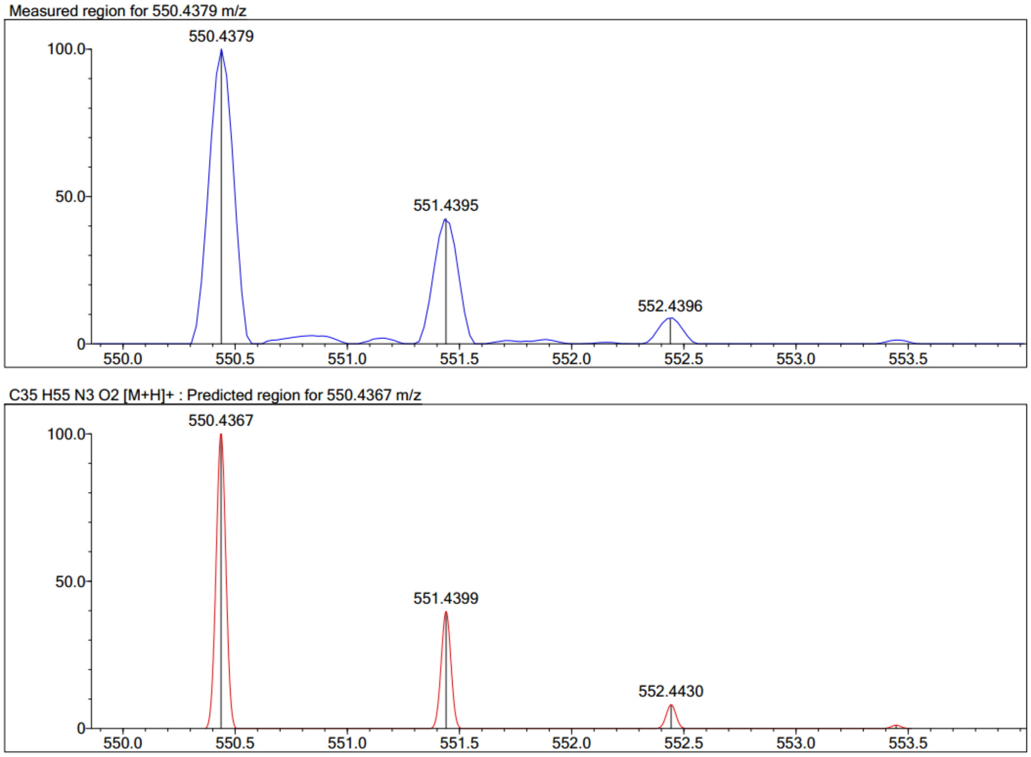

Rank	Score	Formula (M)	Ion	Meas. m/z	Pred. m/z	Df. (mDa)	Df. (ppm)	Iso	DBE
3	89.92	C35 H55 N3 O2	[M+H]+	550.4379	550.4367	1.2	2.18	92.65	10.0

HRMS spectrum of **3d**

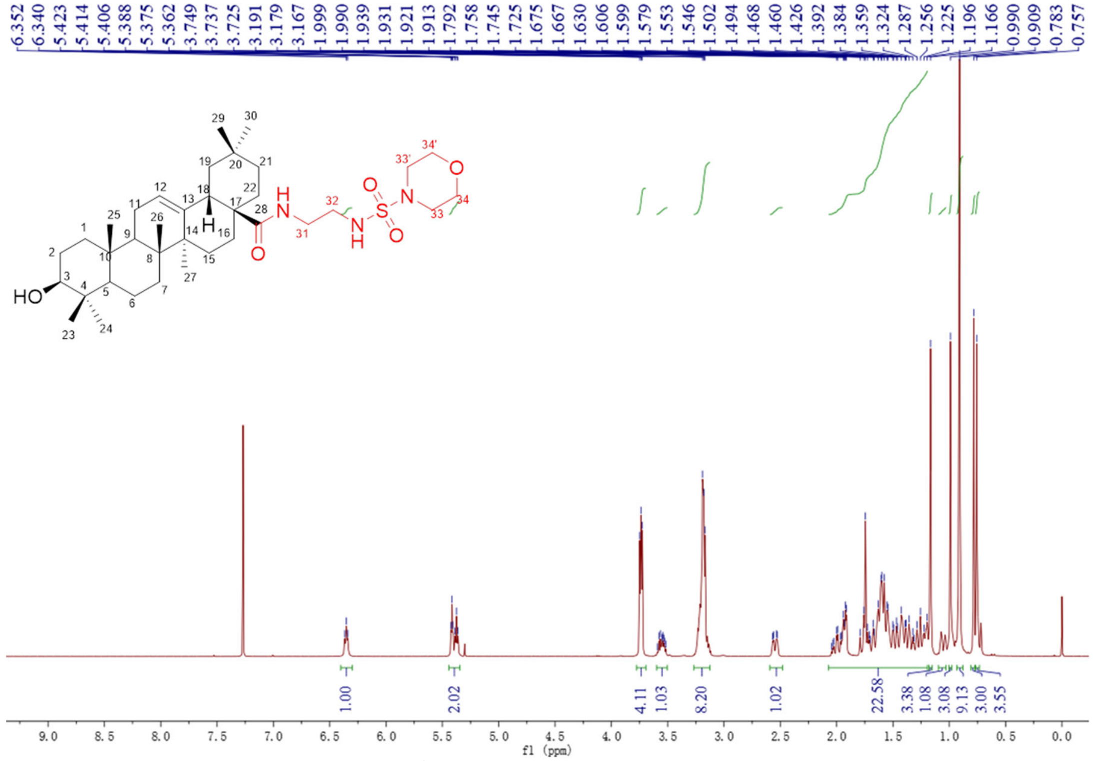

^{1}H NMR spectrum of **3e**

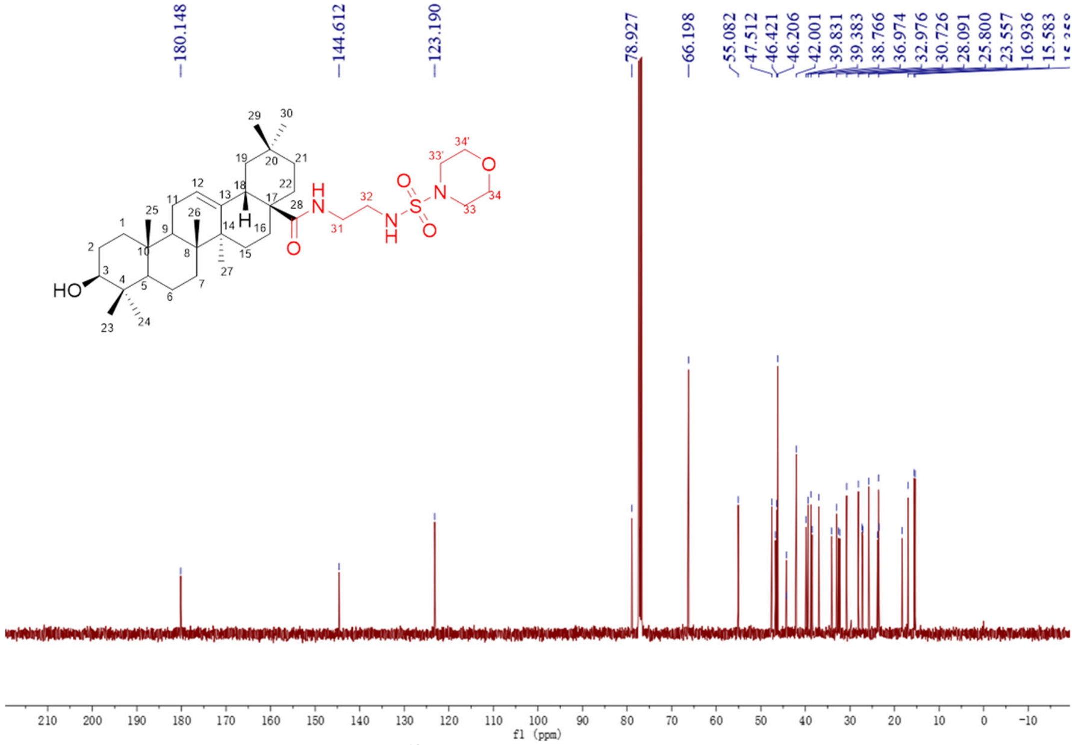

^{13}C NMR spectrum of **3e**

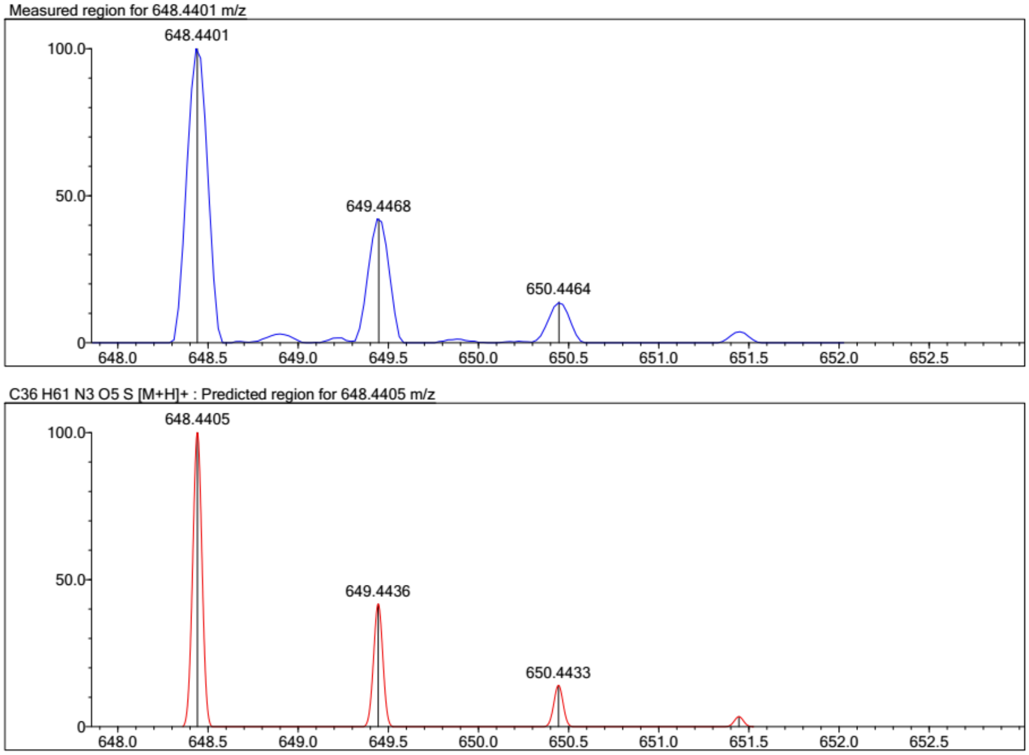

Rank	Score	Formula (M)	Ion	Meas. m/z	Pred. m/z	Df. (mDa)	Df. (ppm)	Iso	DBE
1	100.00	C36 H61 N3 O5 S	[M+H]+	648.4401	648.4405	-0.4	-0.62	100.00	8.0

HRMS spectrum of **3e**

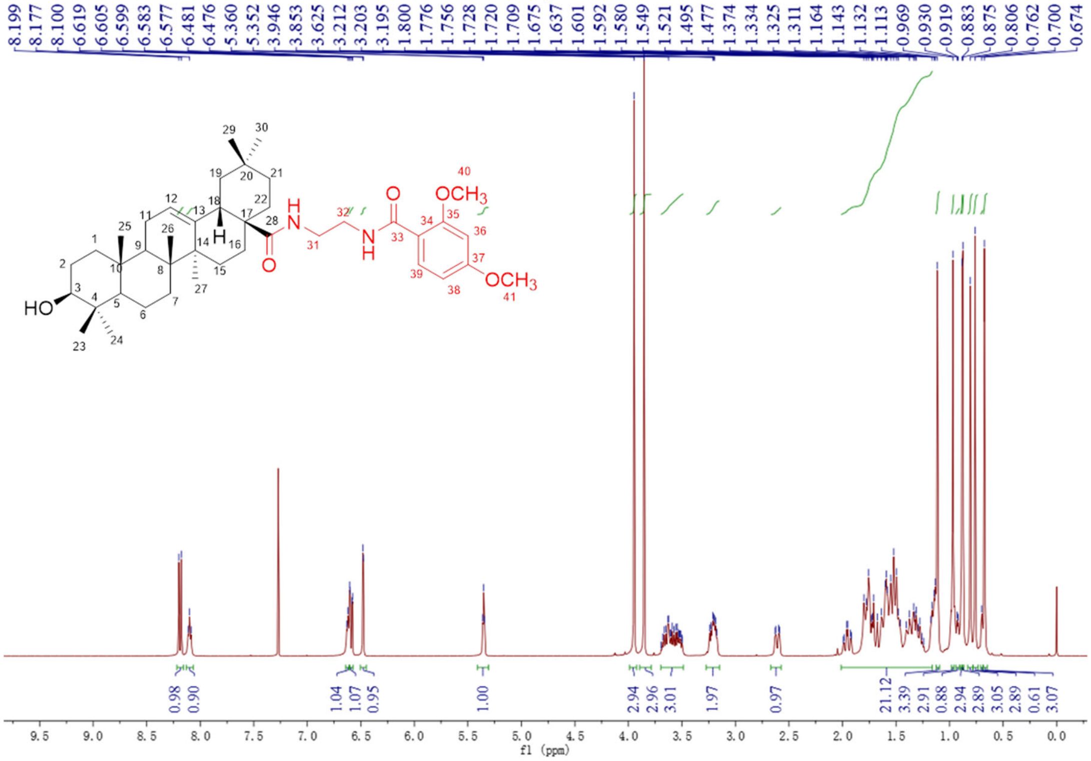

^{1}H spectrum of **3f**

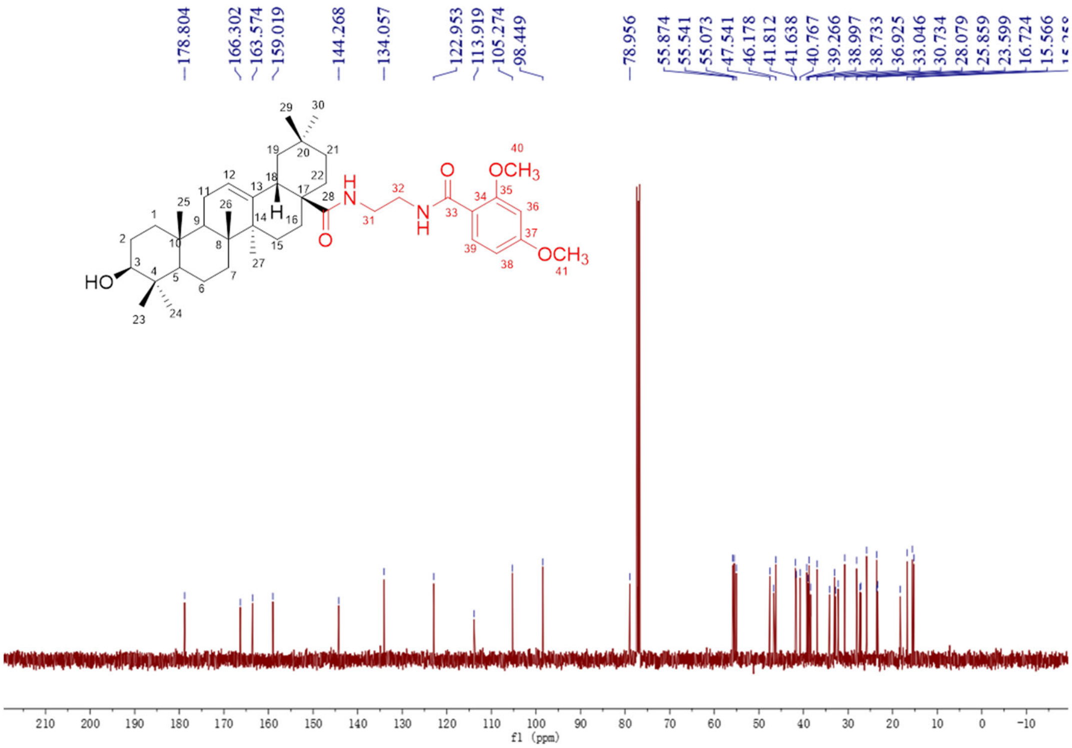

^{13}C spectrum of **3f**

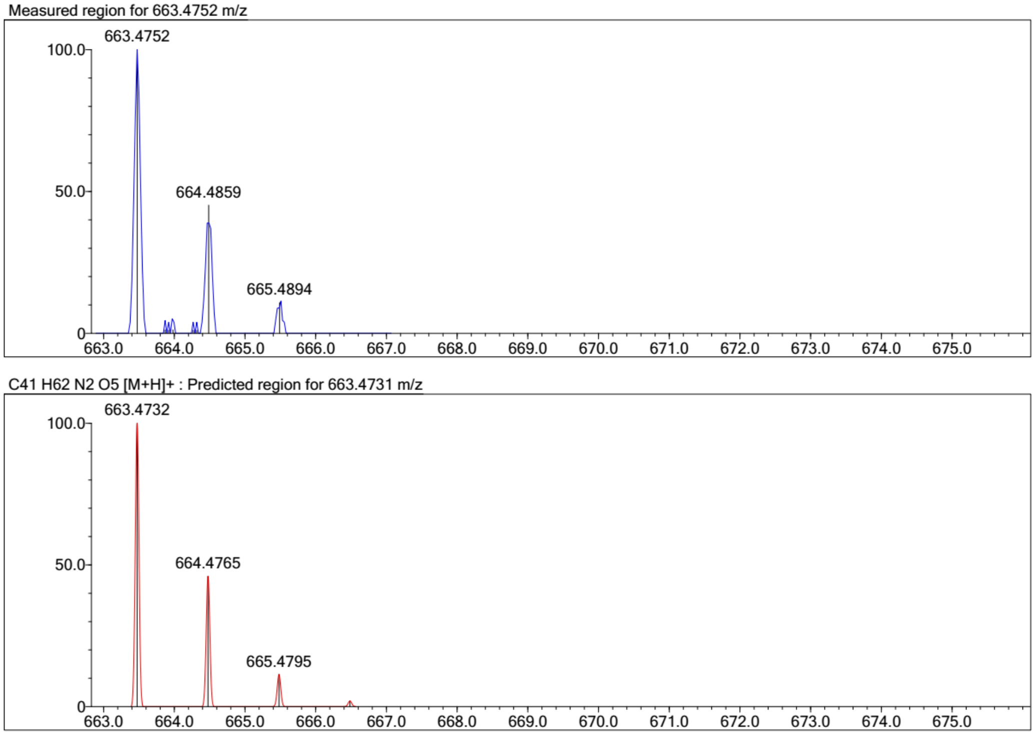

Rank	Score	Formula (M)	Ion	Meas. m/z	Pred. m/z	Df. (mDa)	Df. (ppm)	Iso	DBE
2	26.42	C41 H62 N2 O5	[M+H]+	663.4752	663.4731	2.1	3.17	27.94	12.0

HRMS spectrum of **3f**

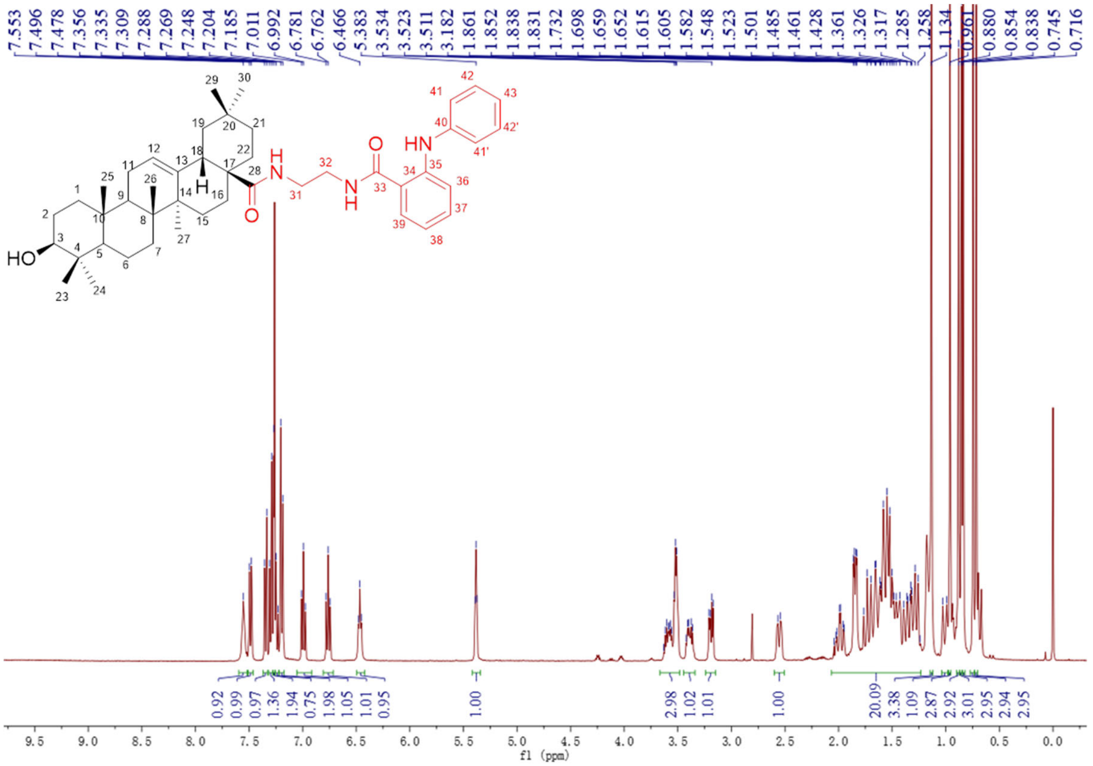

^{1}H spectrum of **3g**

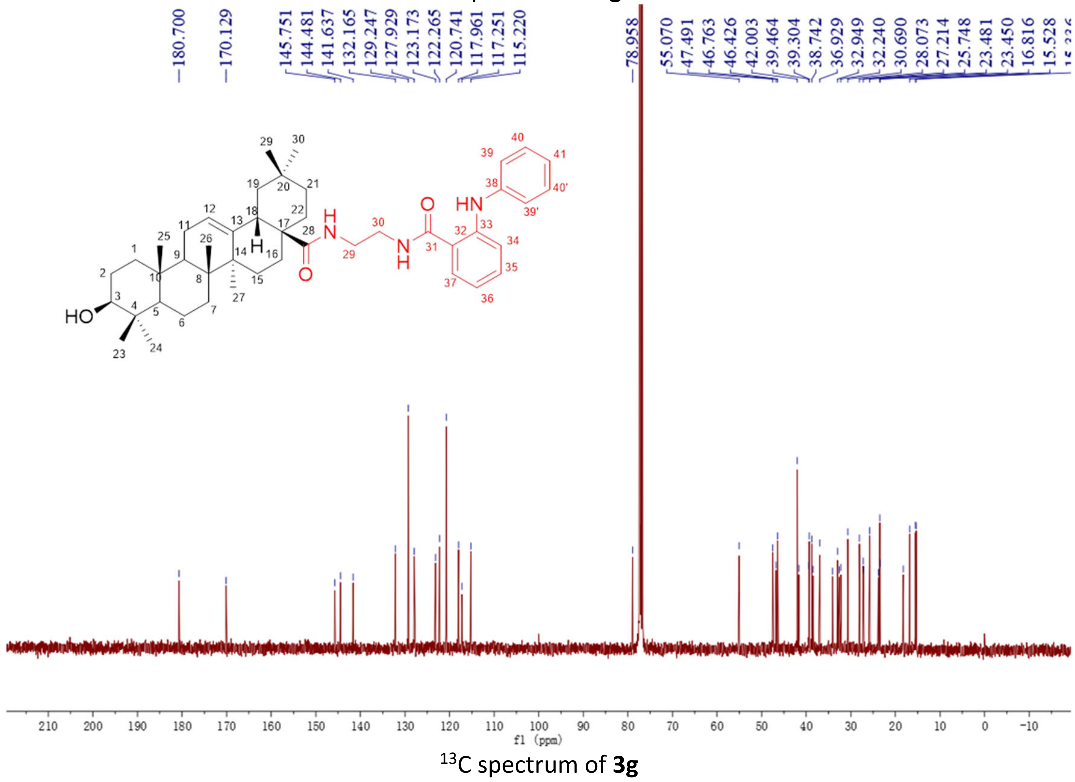

^{13}C spectrum of **3g**

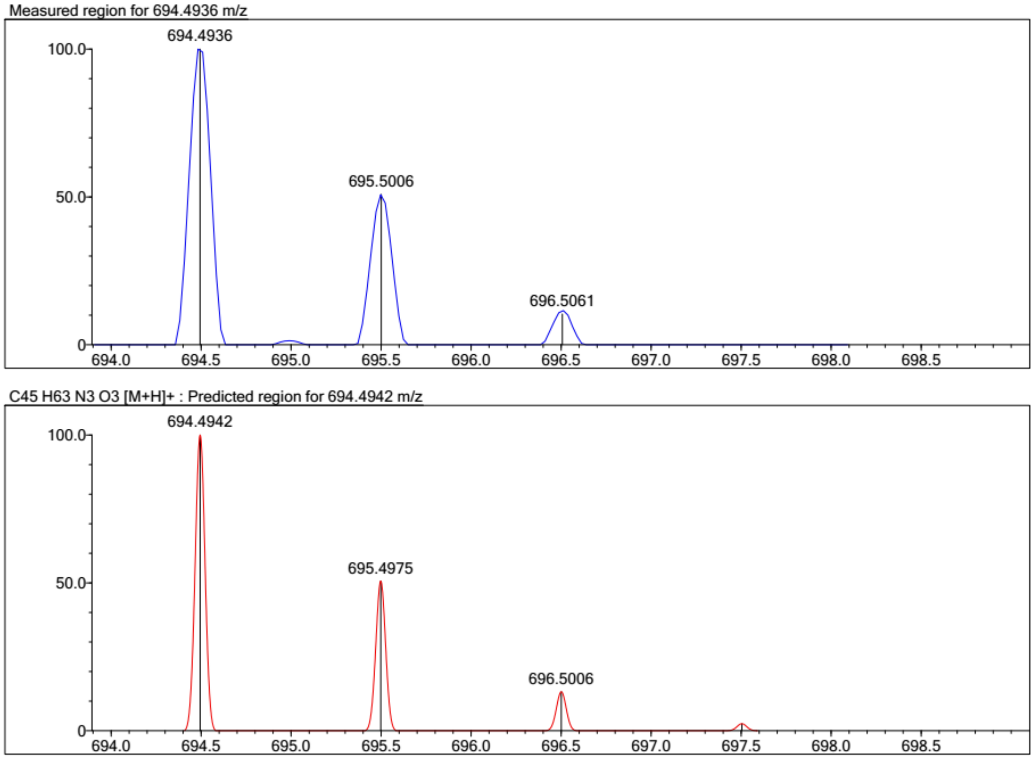

Rank	Score	Formula (M)	Ion	Meas. m/z	Pred. m/z	Df. (mDa)	Df. (ppm)	Iso	DBE
1	100.00	C45 H63 N3 O3	[M+H]+	694.4936	694.4942	-0.6	-0.86	100.00	16.0

HRMS spectrum of **3g**

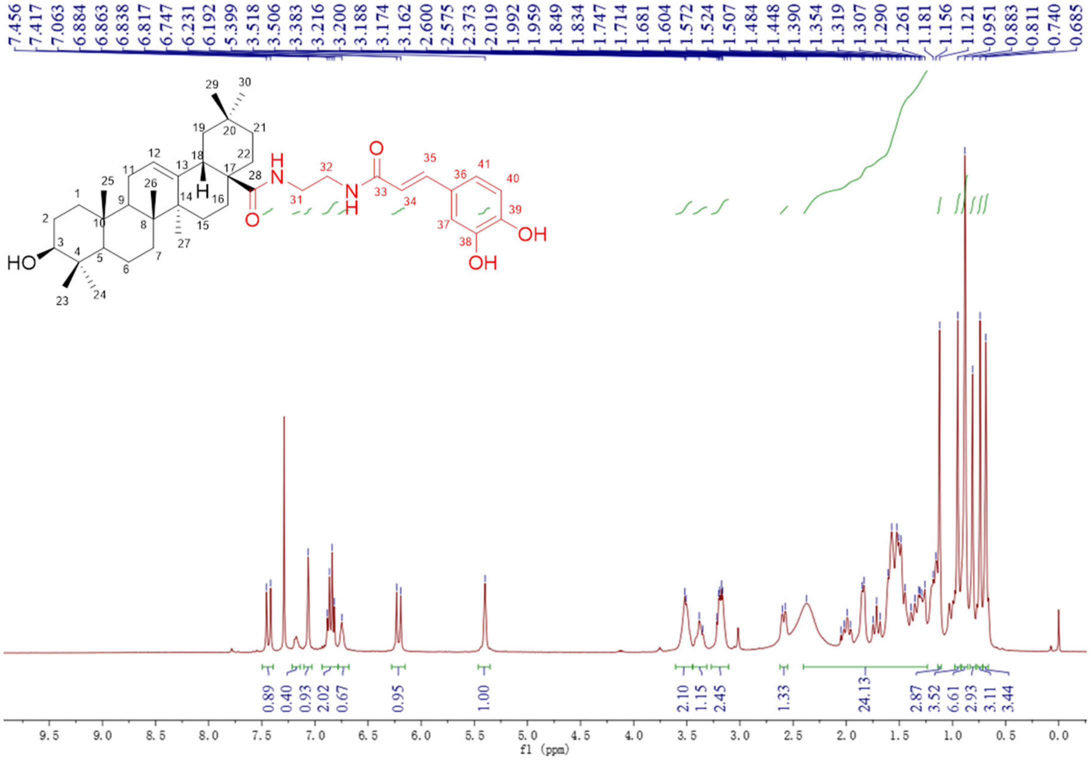

^{1}H NMR spectrum of **3h**

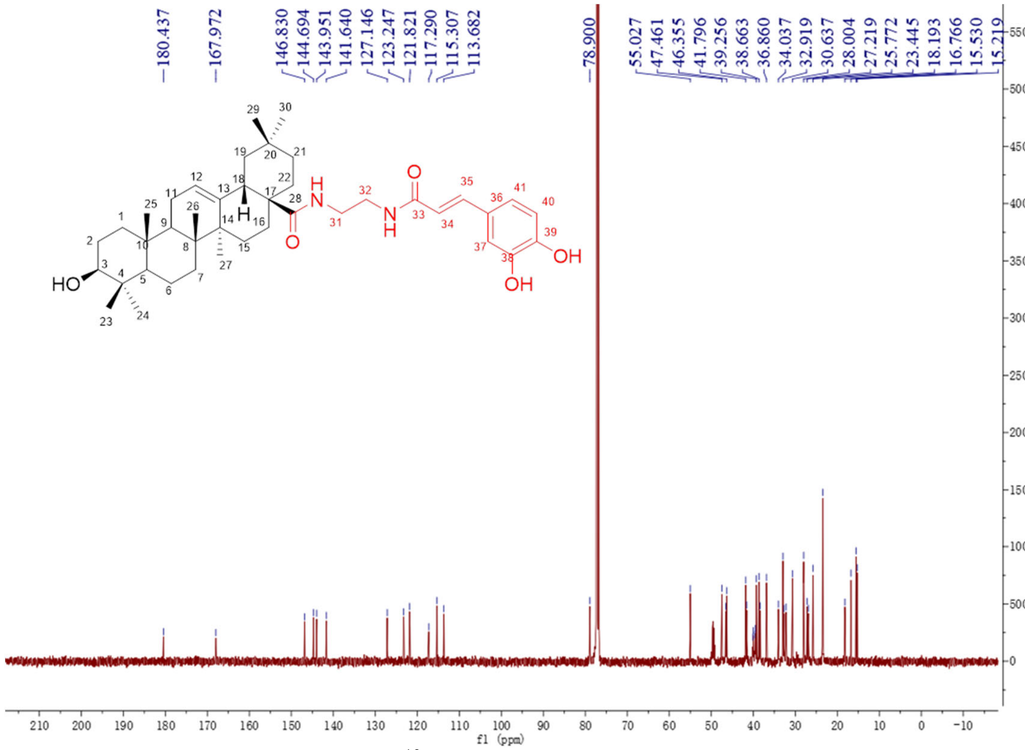

^{13}C NMR spectrum of **3h**

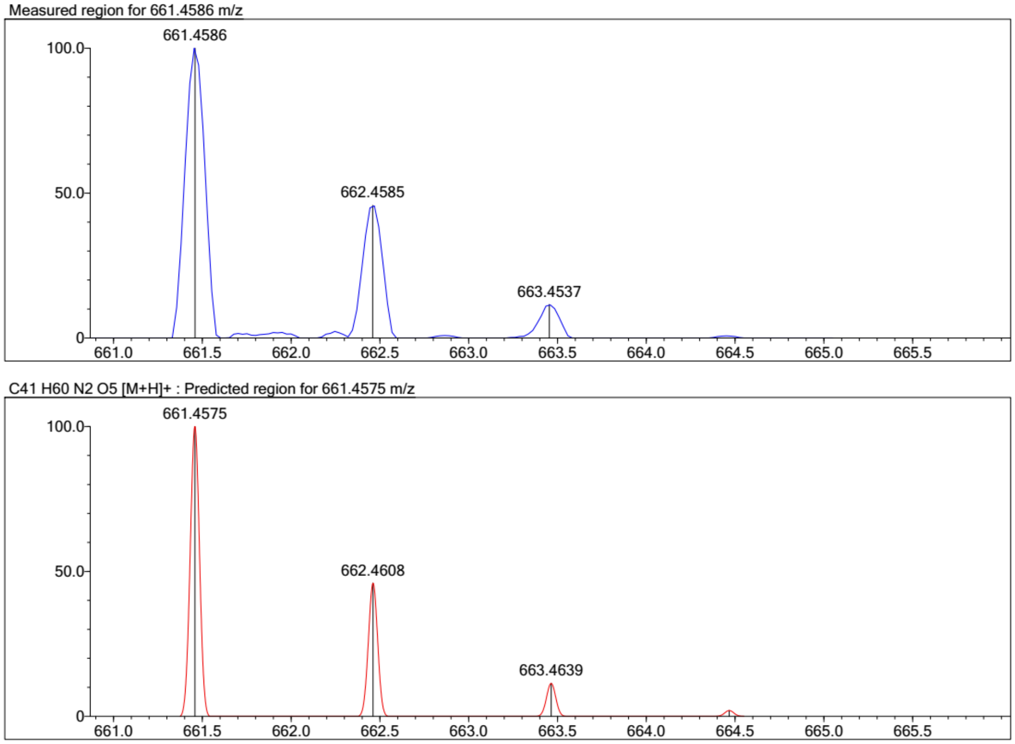

Rank	Score	Formula (M)	Ion	Meas. m/z	Pred. m/z	Df. (mDa)	Df. (ppm)	Iso	DBE
3	82.86	C41 H60 N2 O5	[M+H]+	661.4586	661.4575	1.1	1.66	84.25	13.0

HRMS spectrum of **3h**

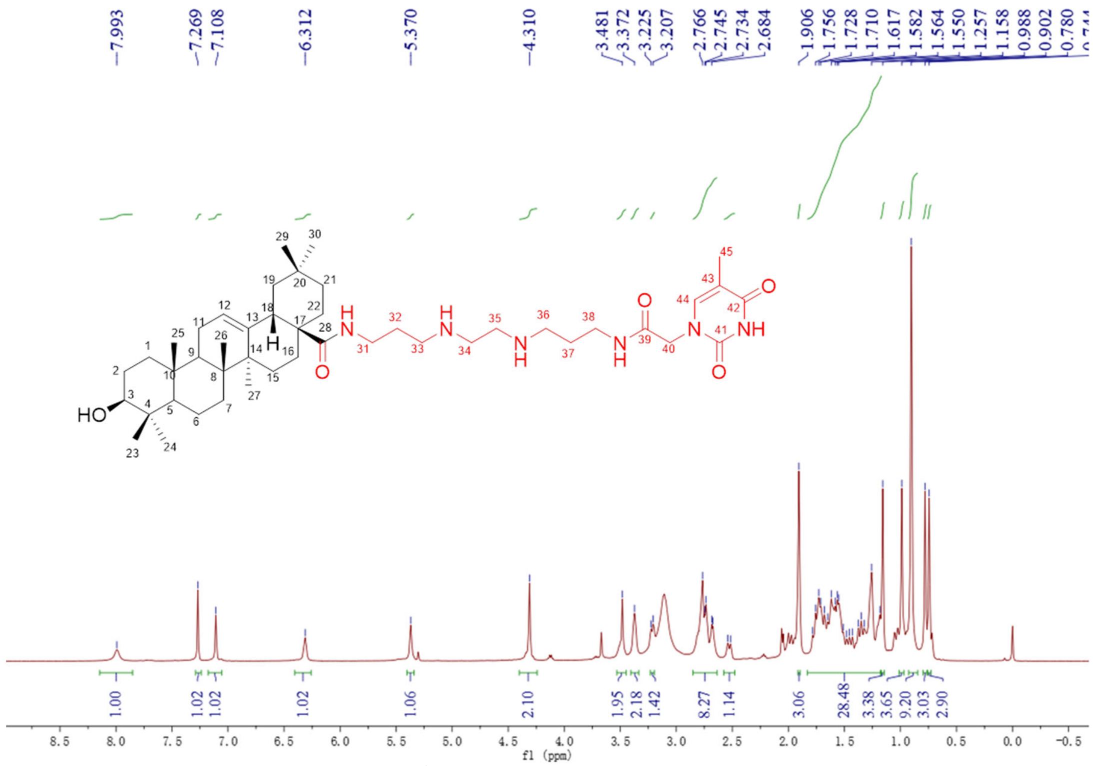

^{1}H NMR spectrum of **3i**

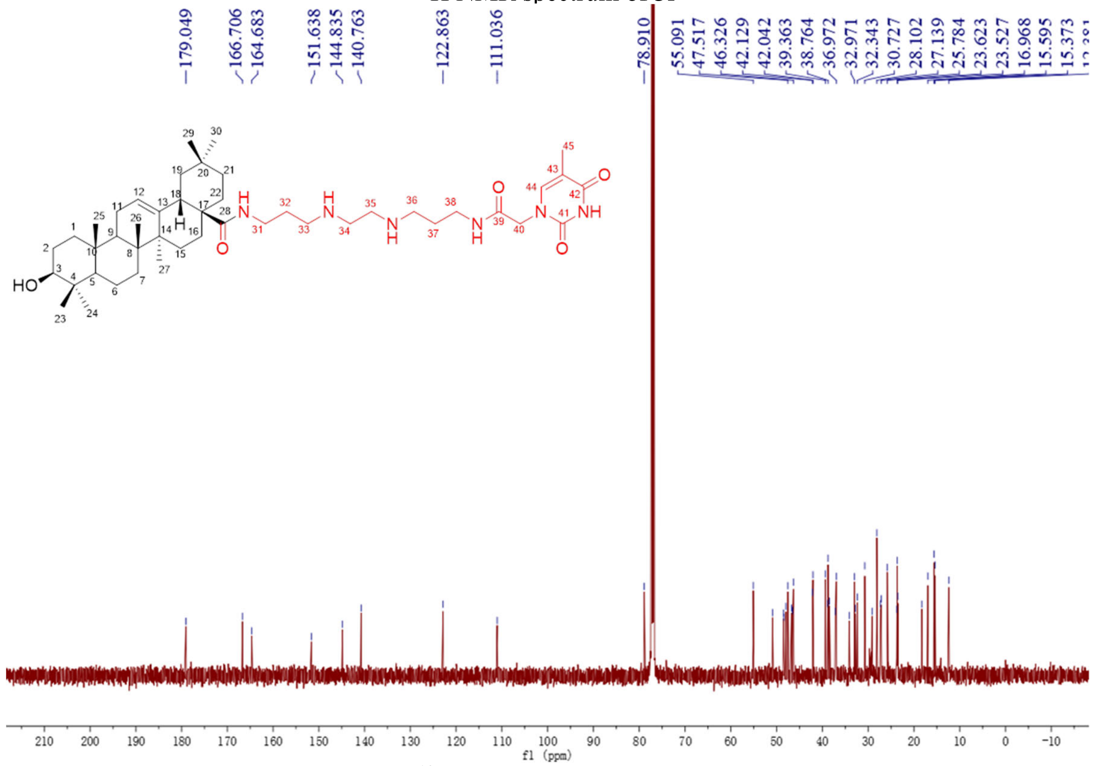

^{13}C NMR spectrum of **3i**

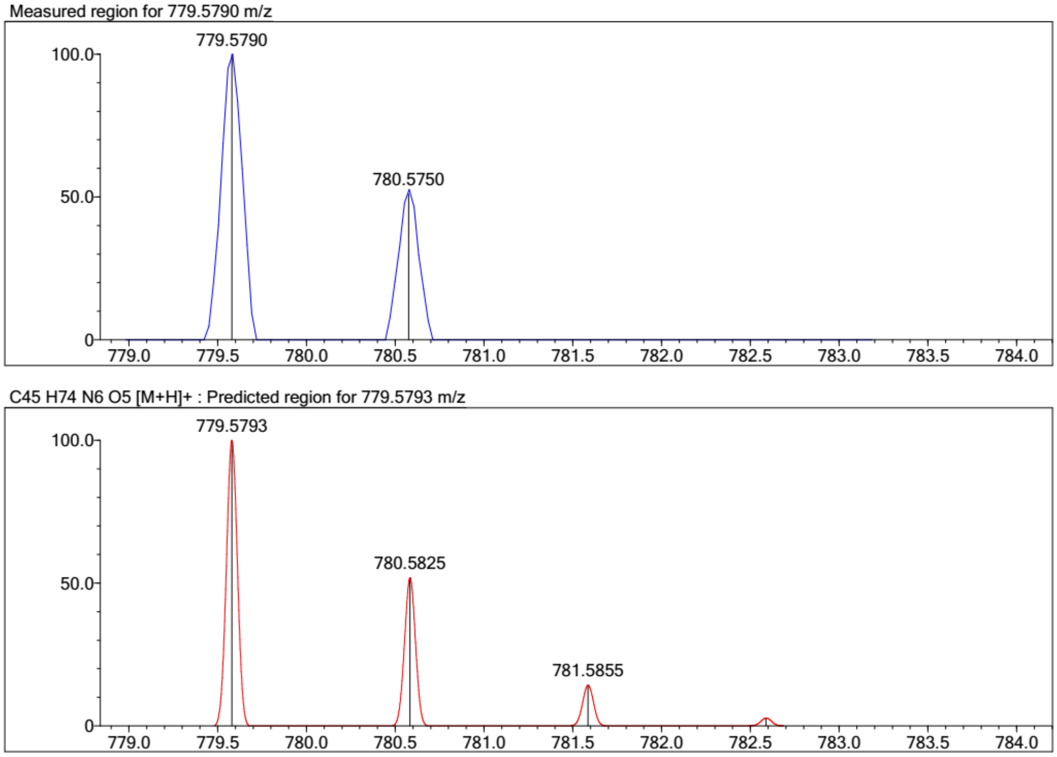

Rank	Score	Formula (M)	Ion	Meas. m/z	Pred. m/z	Df. (mDa)	Df. (ppm)	Iso	DBE
1	0.00	C45 H74 N6 O5	[M+H]+	779.5790	779.5793	-0.3	-0.38	0.00	12.0

HRMS spectrum of 3**i**

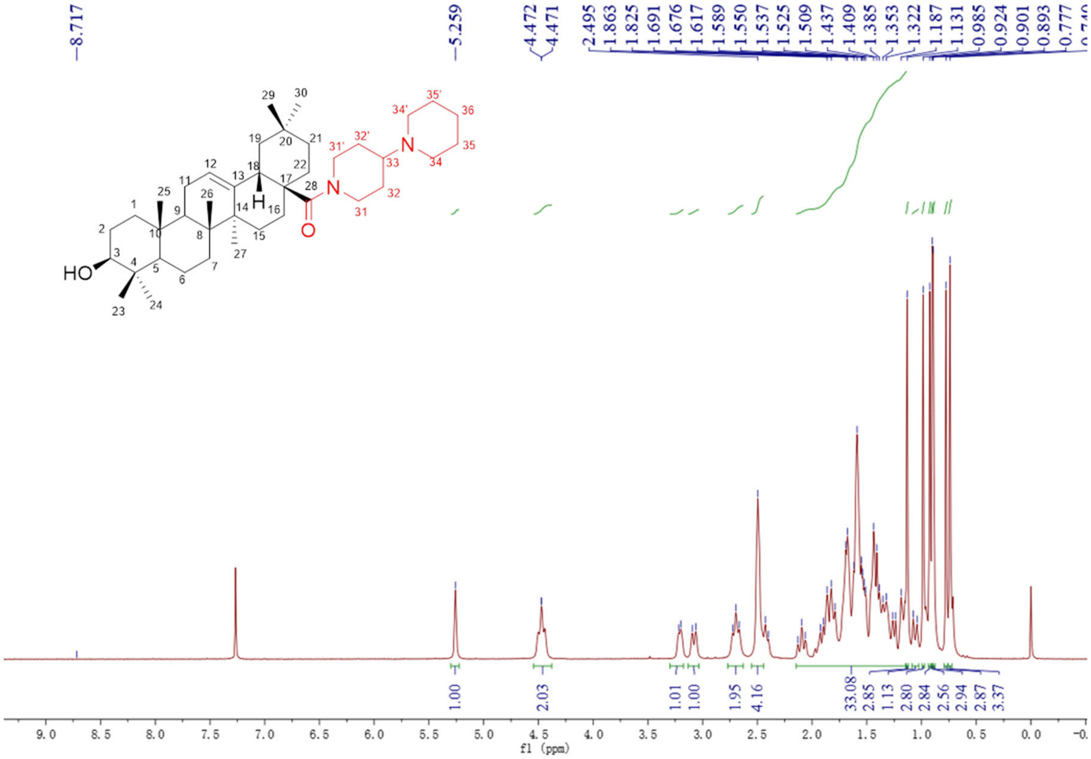

^{1}H NMR spectrum of **3j**

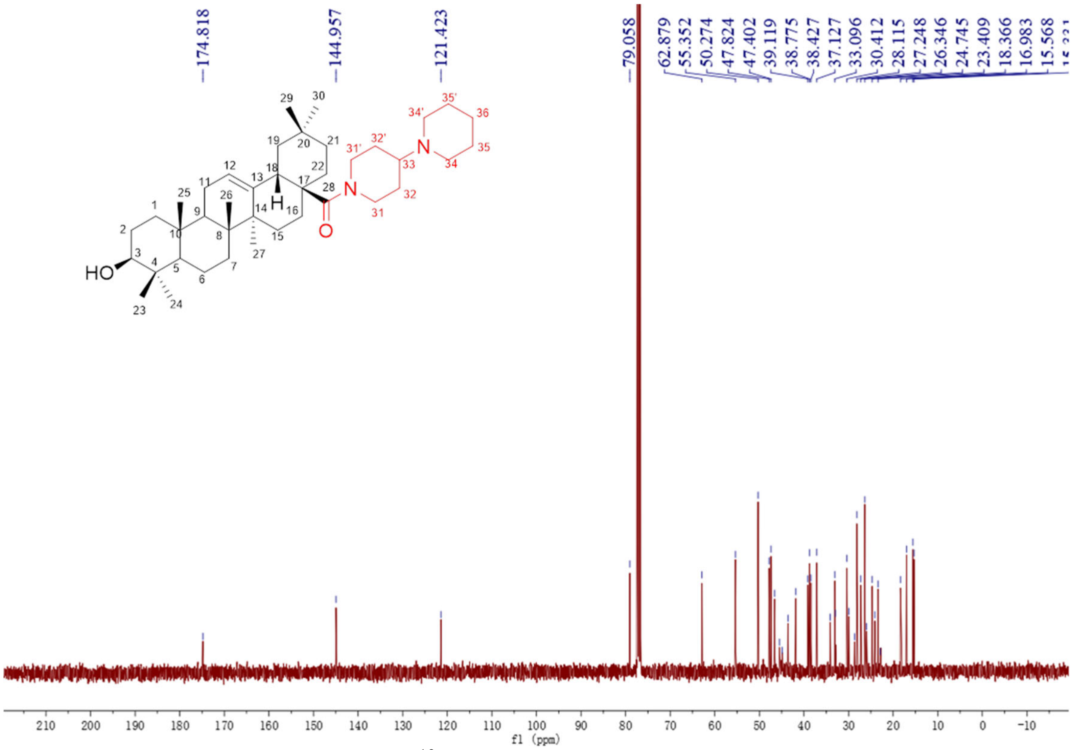

^{13}C NMR spectrum of **3j**

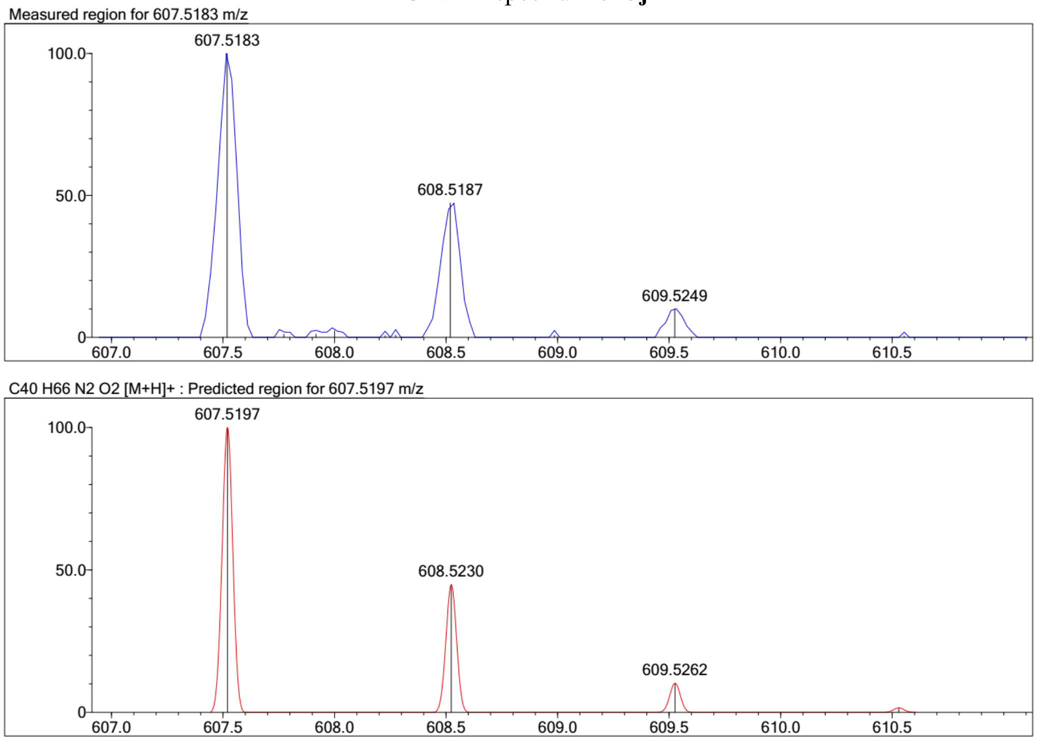

Rank	Score	Formula (M)	Ion	Meas. m/z	Pred. m/z	Df. (mDa)	Df. (ppm)	Iso	DBE
2	96.75	C40 H66 N2 O2	[M+H]+	607.5183	607.5197	-1.4	-2.30	100.00	9.0

HRMS spectrum of **3j**

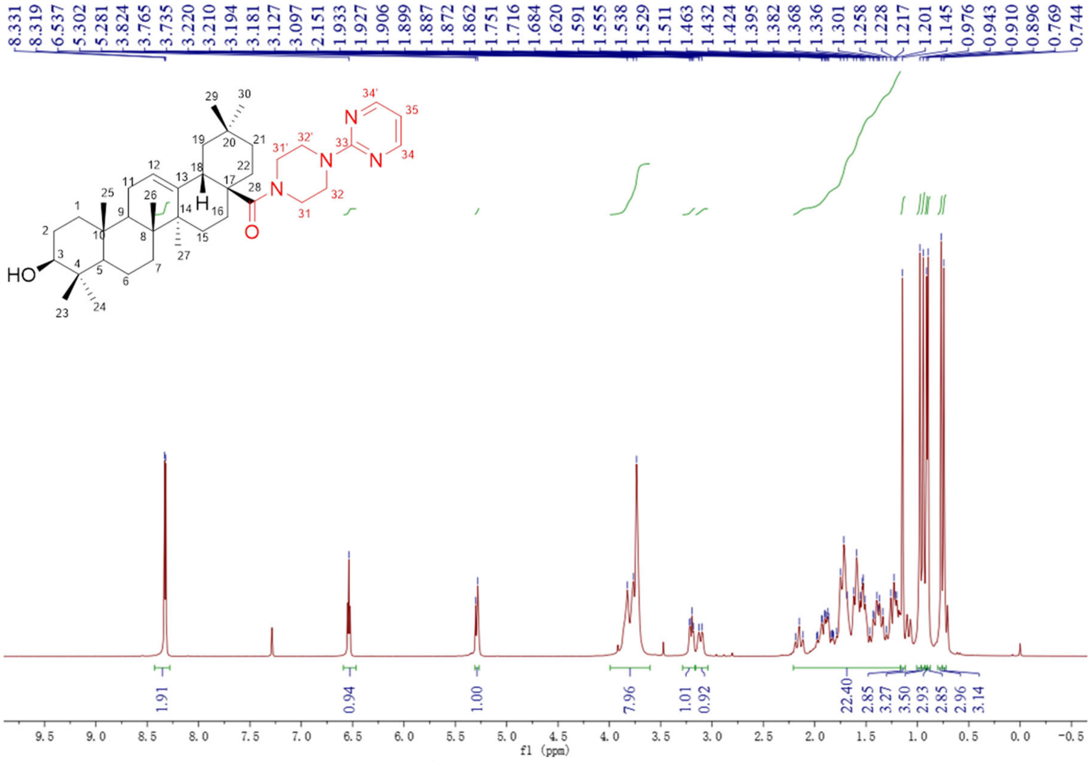

^{1}H NMR spectrum of **3k**

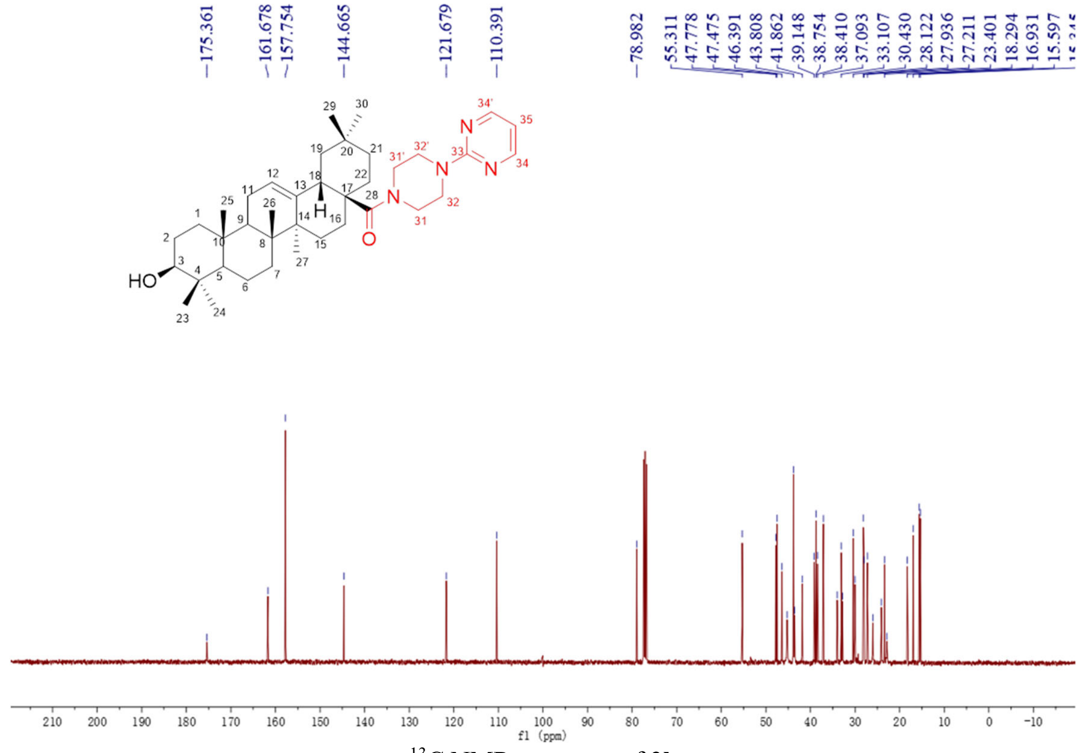

^{13}C NMR spectrum of **3k**

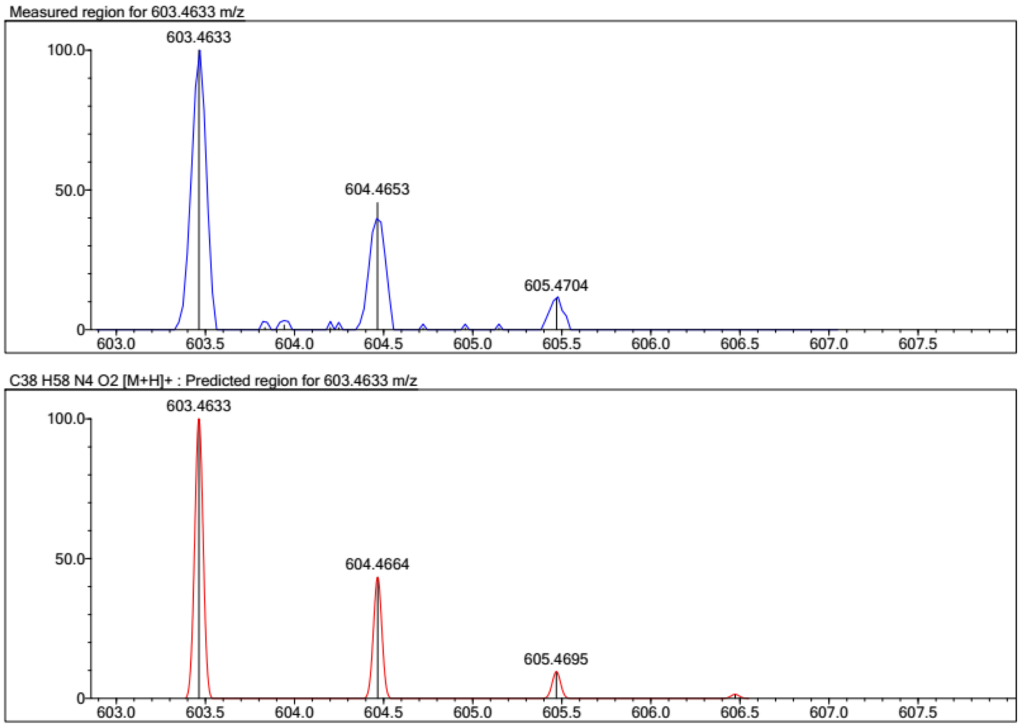

Rank	Score	Formula (M)	Ion	Meas. m/z	Pred. m/z	Df. (mDa)	Df. (ppm)	Iso	DBE
2	86.47	C38 H58 N4 O2	[M+H]+	603.4633	603.4633	0.0	0.00	86.47	12.0

HRMS spectrum of **3k**

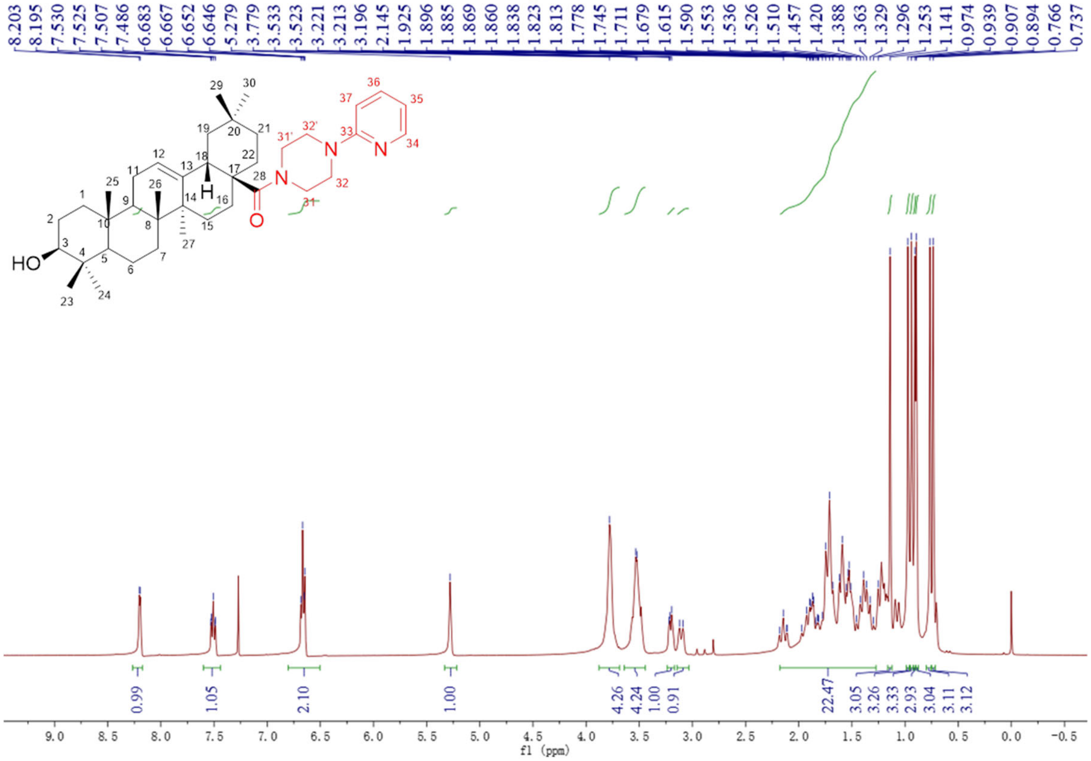

^{1}H spectrum of **3l**

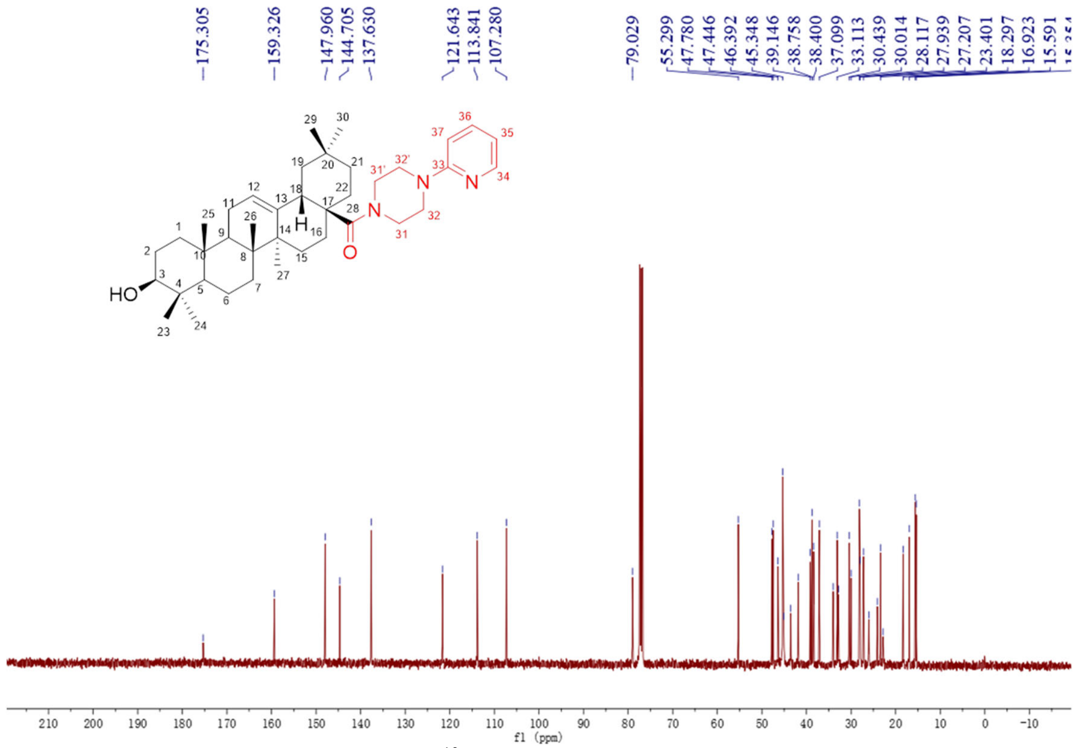

^{13}C spectrum of **3l**

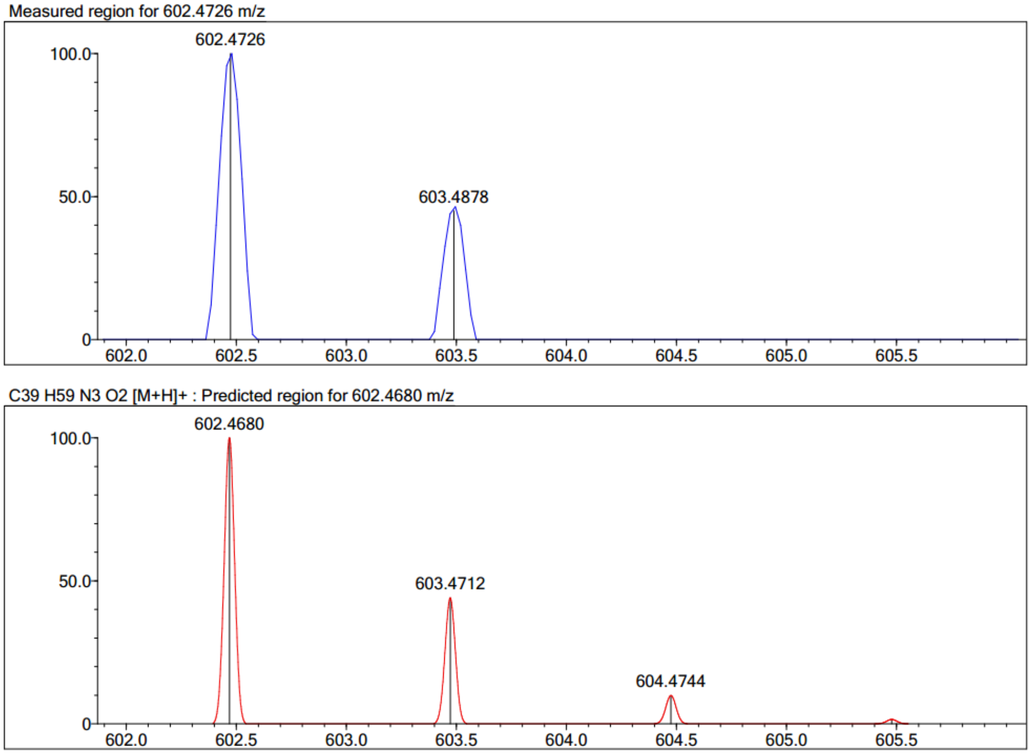

Rank	Score	Formula (M)	Ion	Meas. m/z	Pred. m/z	Df. (mDa)	Df. (ppm)	Iso	DBE
5	0.00	C39 H59 N3 O2	[M+H]+	602.4726	602.4680	4.6	7.64	0.00	12.0

HRMS spectrum of **3l**

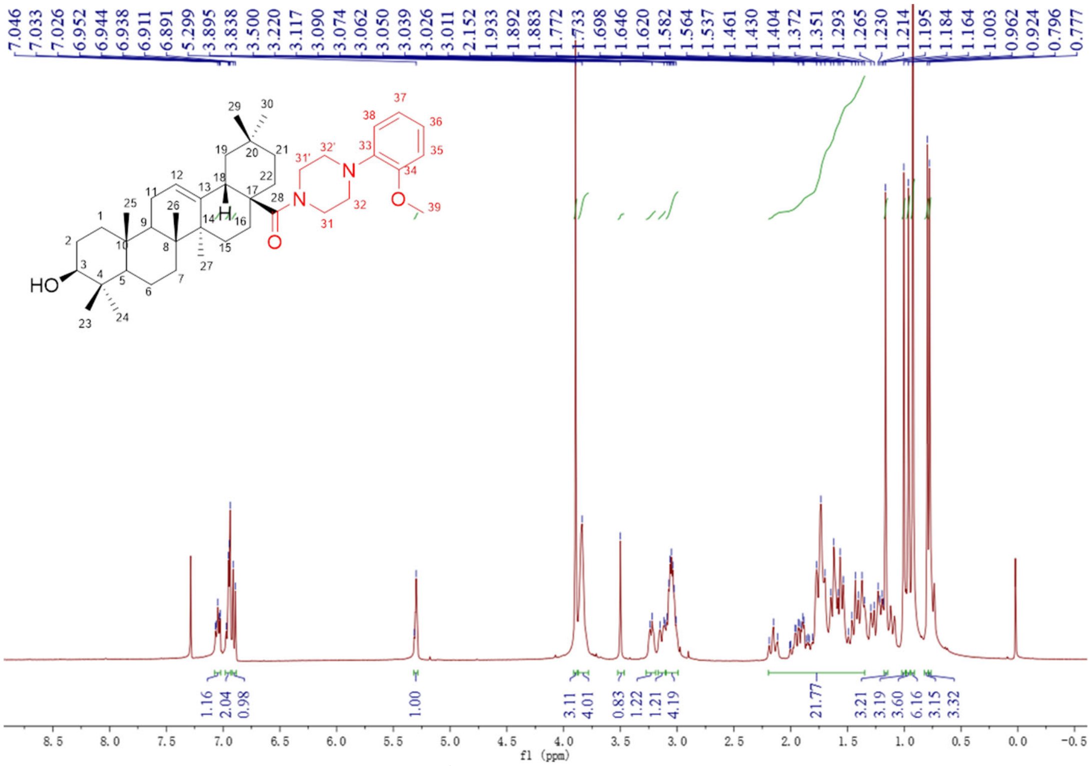

^{1}H spectrum of **3m**

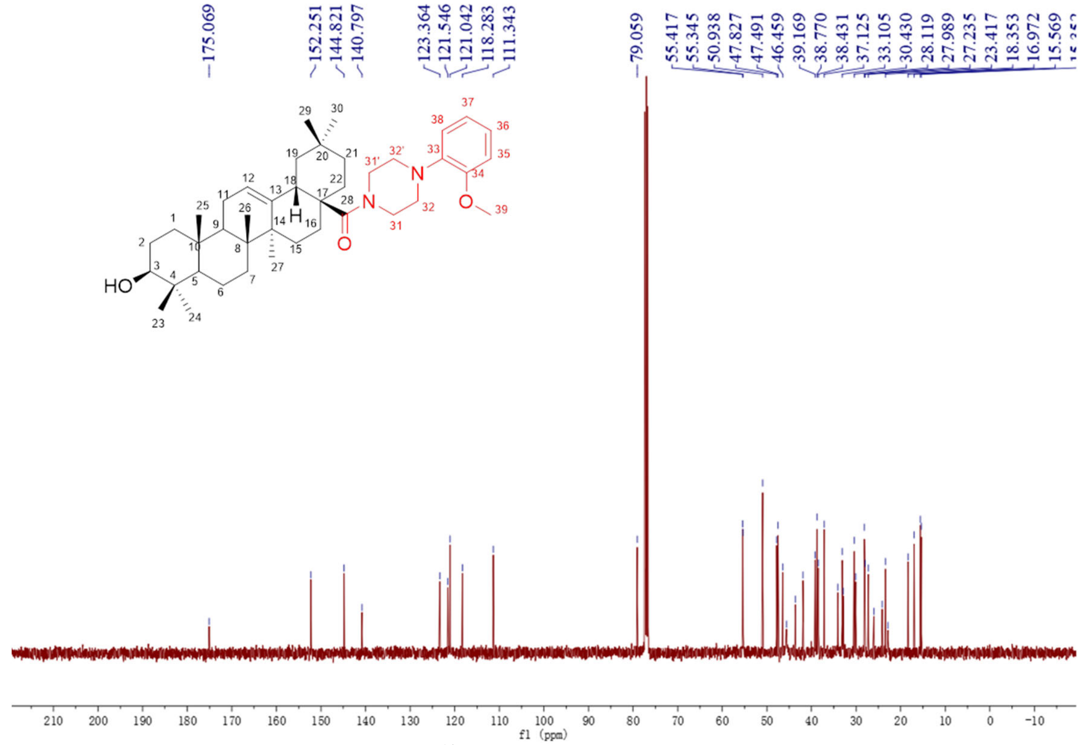

^{13}C spectrum of **3m**

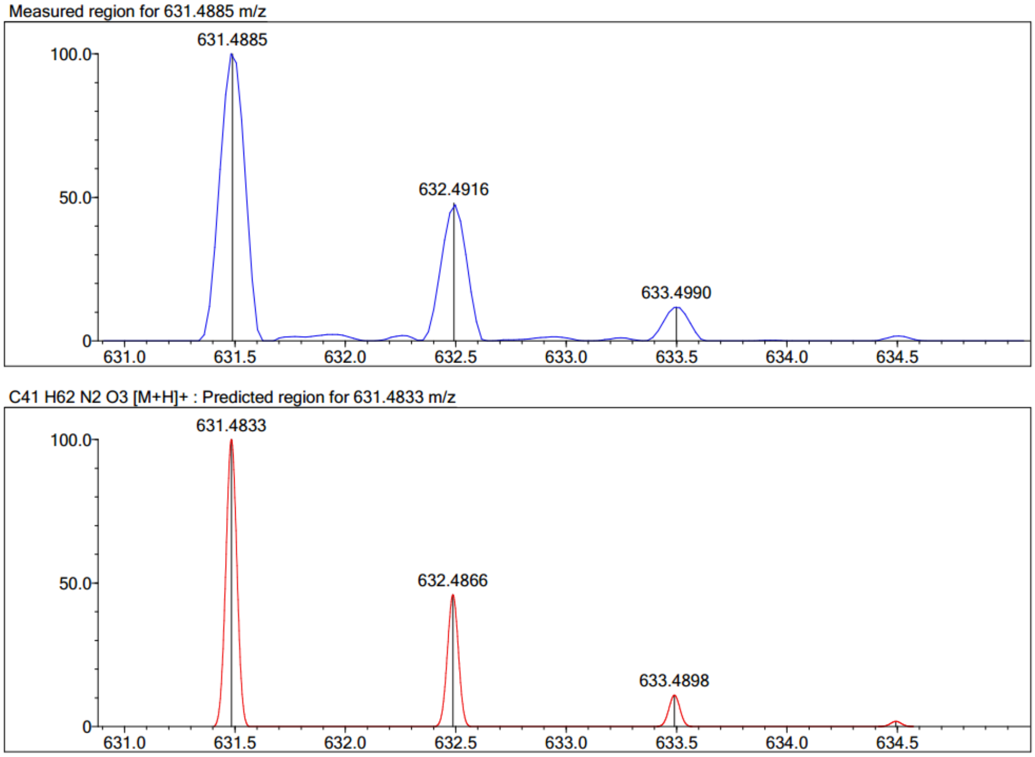

Rank	Score	Formula (M)	Ion	Meas. m/z	Pred. m/z	Df. (mDa)	Df. (ppm)	Iso	DBE
5	51.18	C41 H62 N2 O3	[M+H]+	631.4885	631.4833	5.2	8.23	88.70	12.0

HRMS spectrum of **3m**

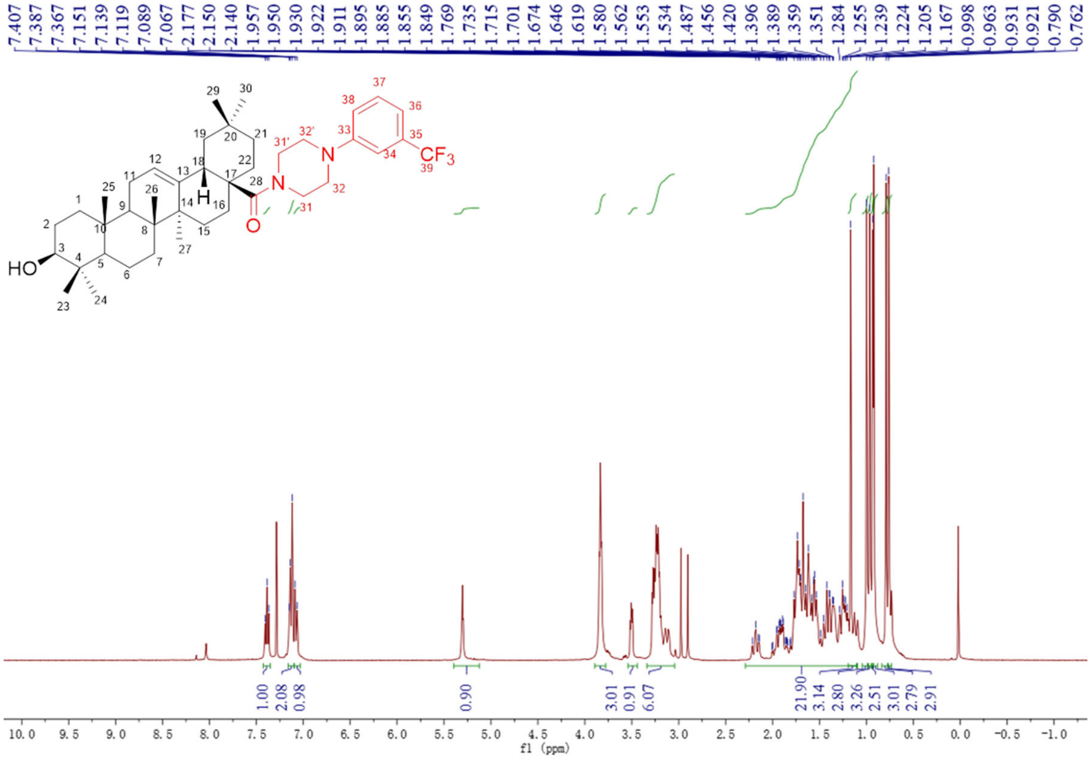

^{1}H spectrum of **3n**

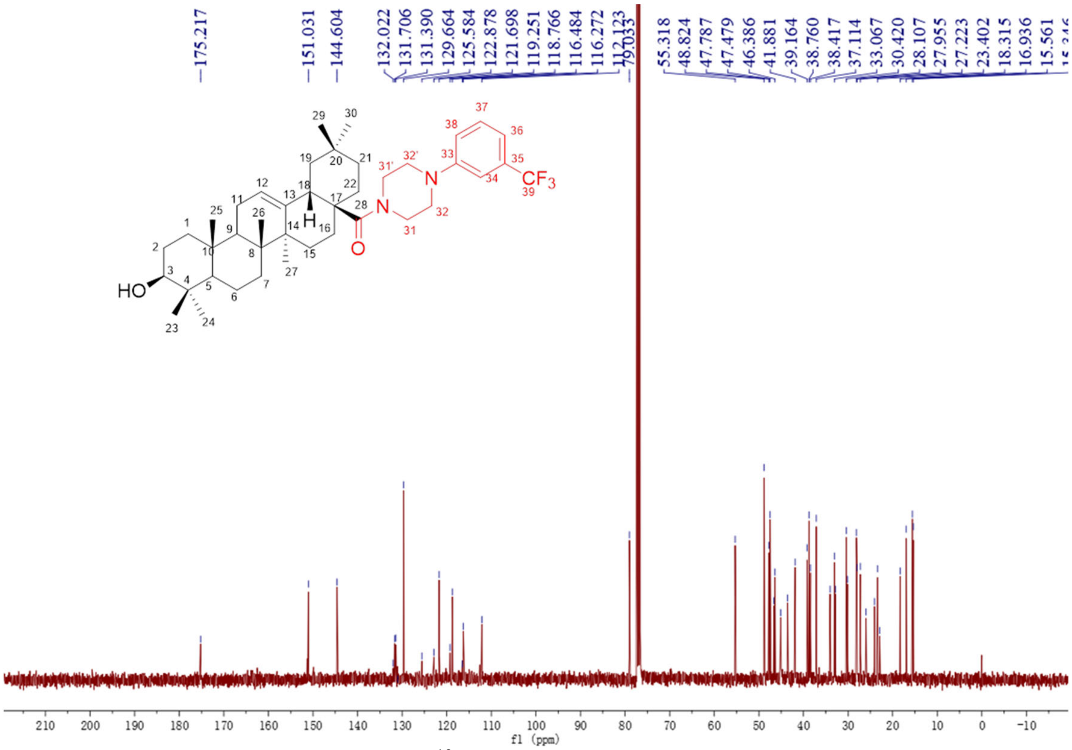

^{13}C spectrum of **3n**

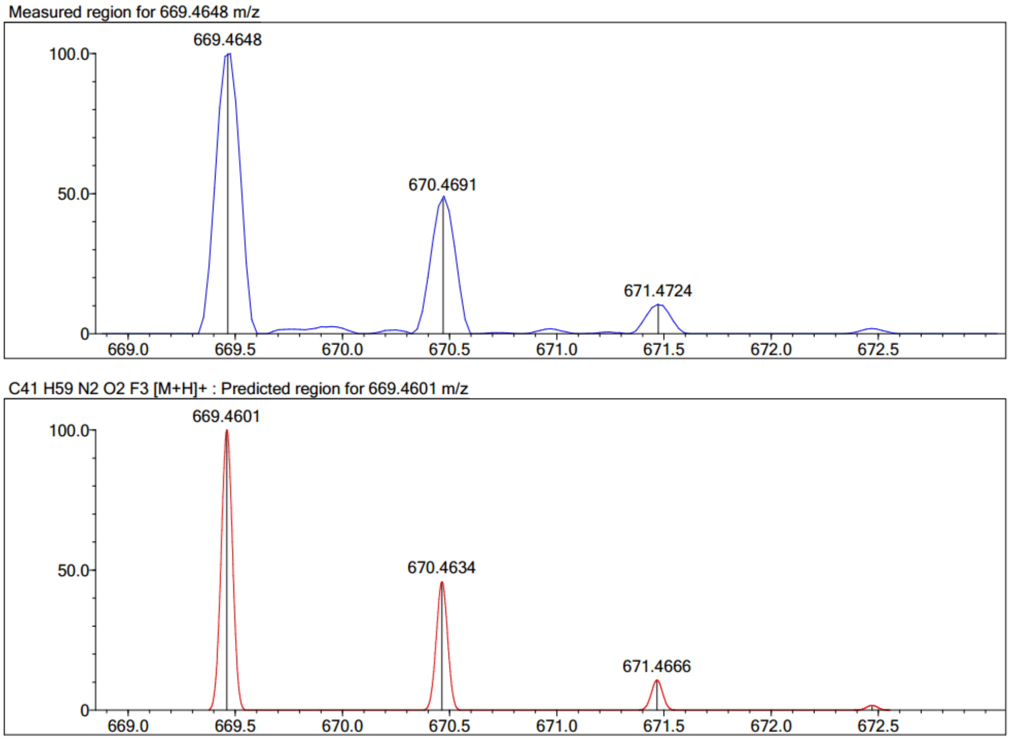

Rank	Score	Formula (M)	Ion	Meas. m/z	Pred. m/z	Df. (mDa)	Df. (ppm)	Iso	DBE
15	65.37	C41 H59 N2 O2 F3	[M+H]+	669.4648	669.4601	4.7	7.02	93.65	12.0

HRMS spectrum of **3n**

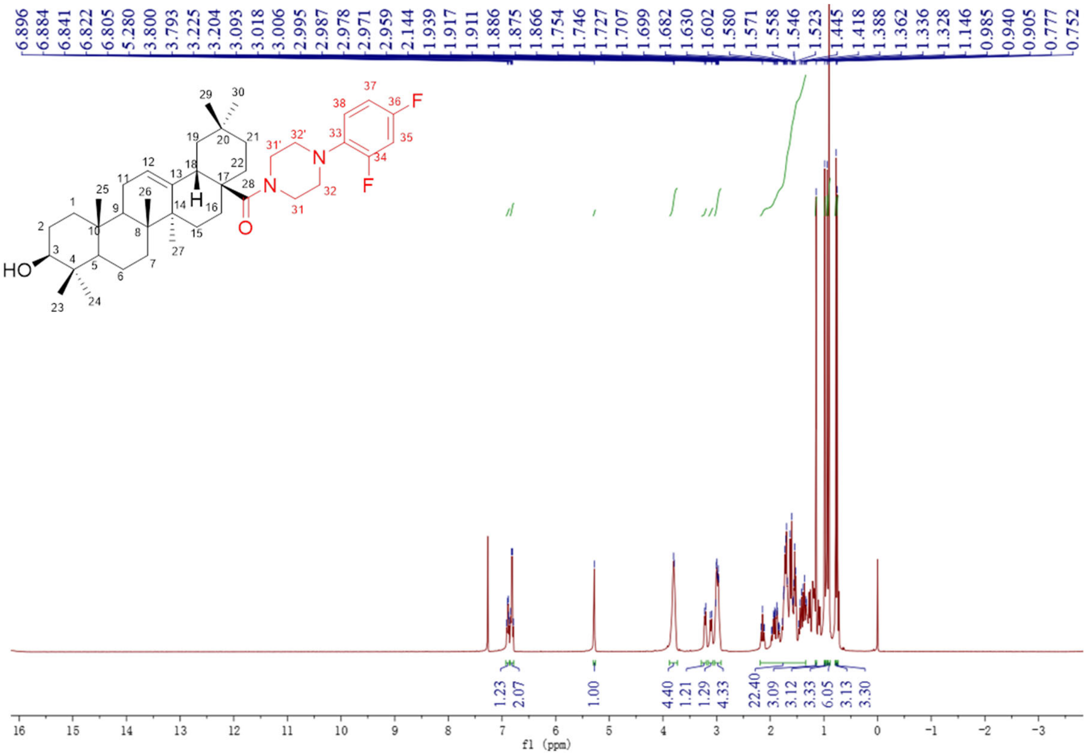

^{1}H NMR spectrum of **3o**

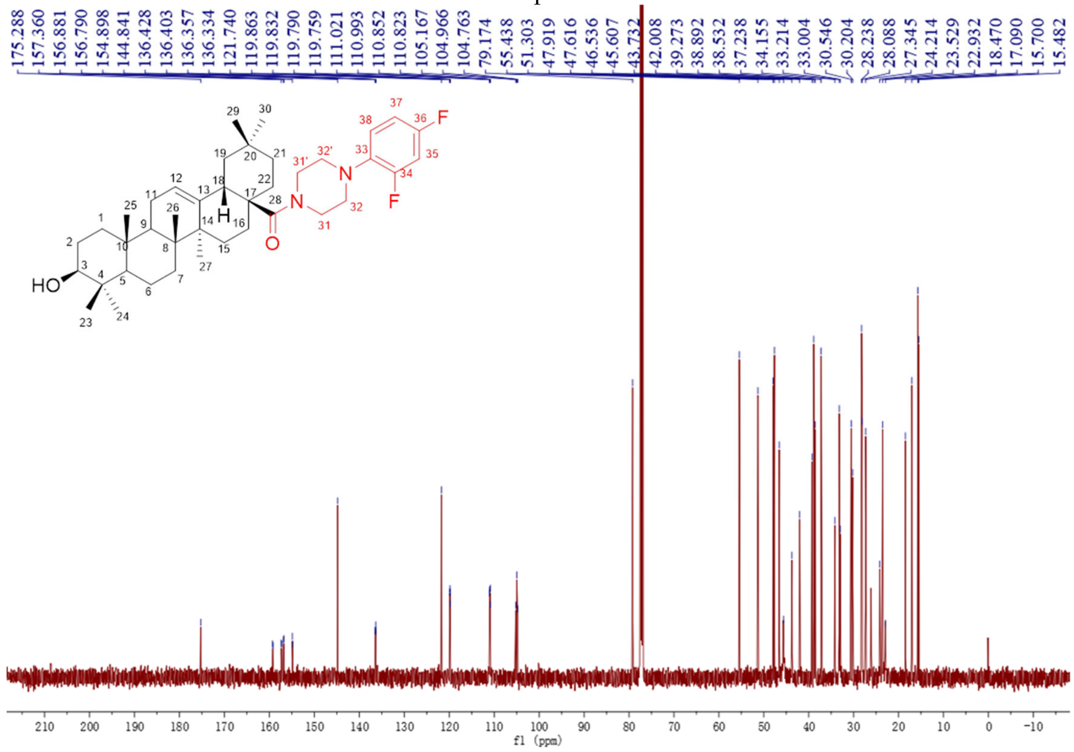

^{13}C NMR spectrum of **3o**

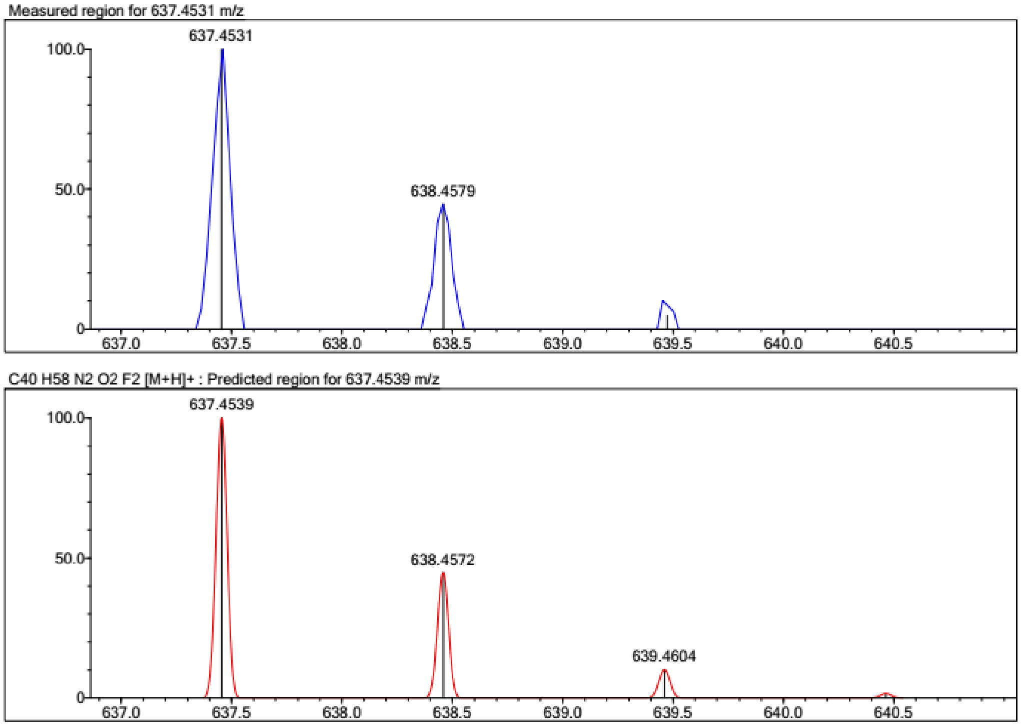

Rank	Score	Formula (M)	Ion	Meas. m/z	Pred. m/z	Df. (mDa)	Df. (ppm)	Iso	DBE
3	68.96	C40 H58 N2 O2 F2	[M+H]+	637.4531	637.4539	-0.8	-1.25	69.39	12.0

HRMS spectrum of **3o**

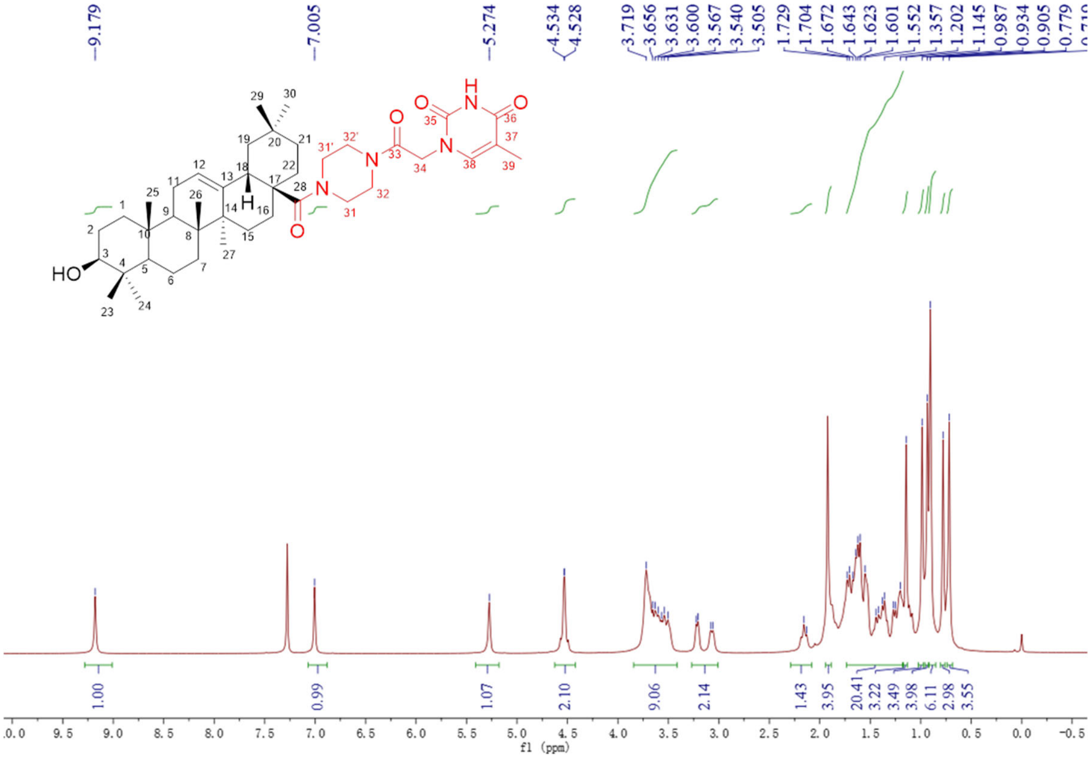

^{1}H spectrum of **3p**

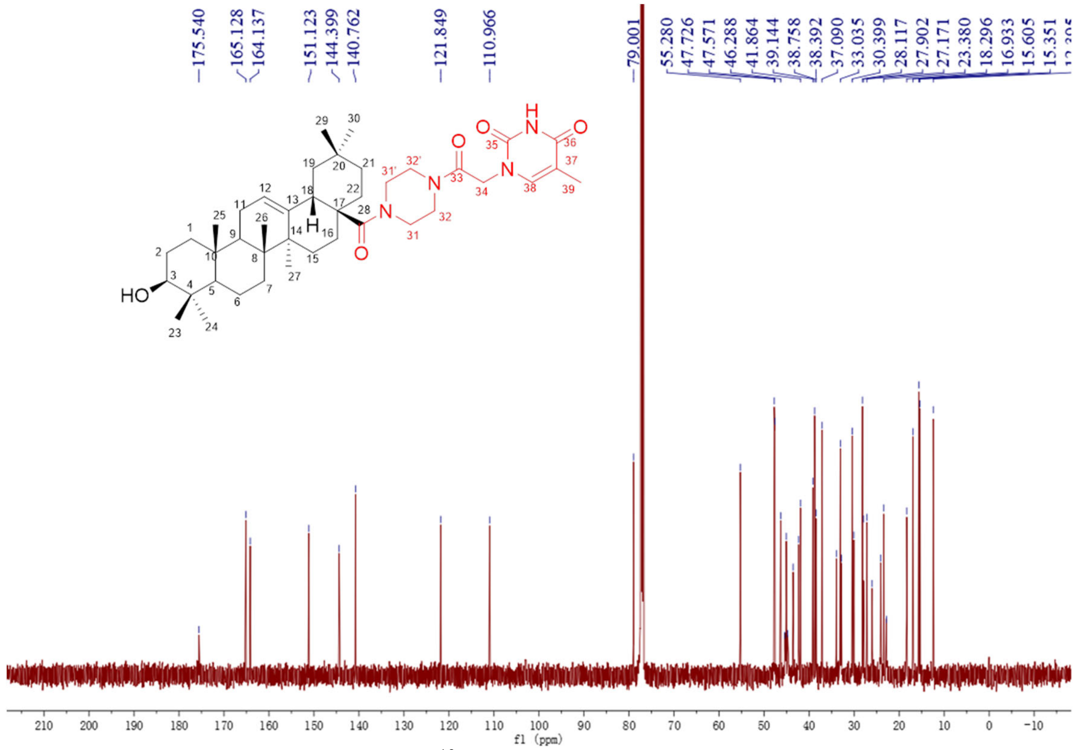

^{13}C spectrum of **3p**

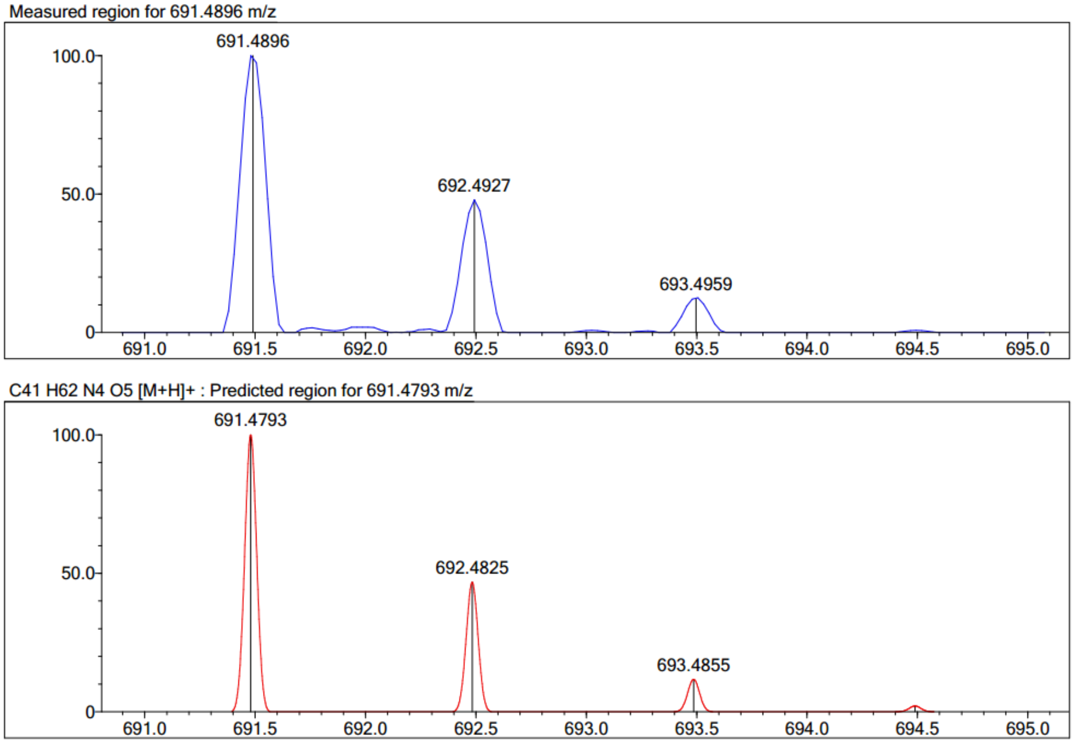

Rank	Score	Formula (M)	Ion	Meas. m/z	Pred. m/z	Df. (mDa)	Df. (ppm)	Iso	DBE
12	23.85	C41 H62 N4 O5	[M+H]+	691.4896	691.4793	10.3	14.90	88.53	13.0

HRMS spectrum of **3p**

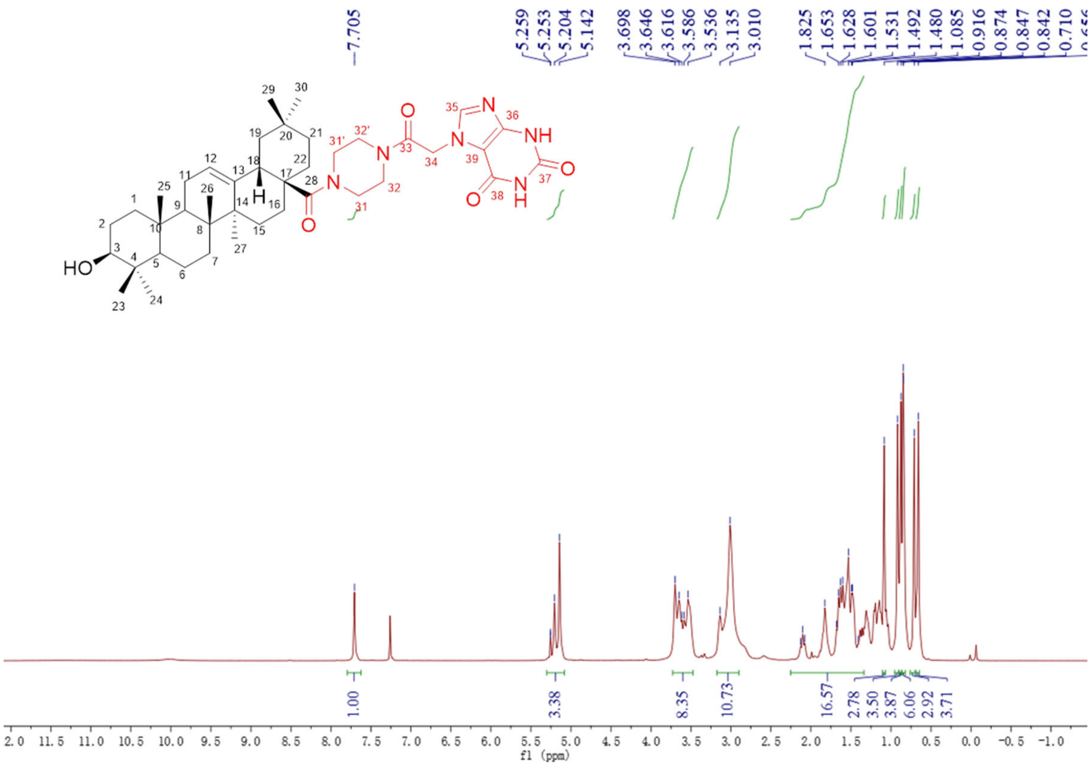

^{1}H spectrum of **3q**

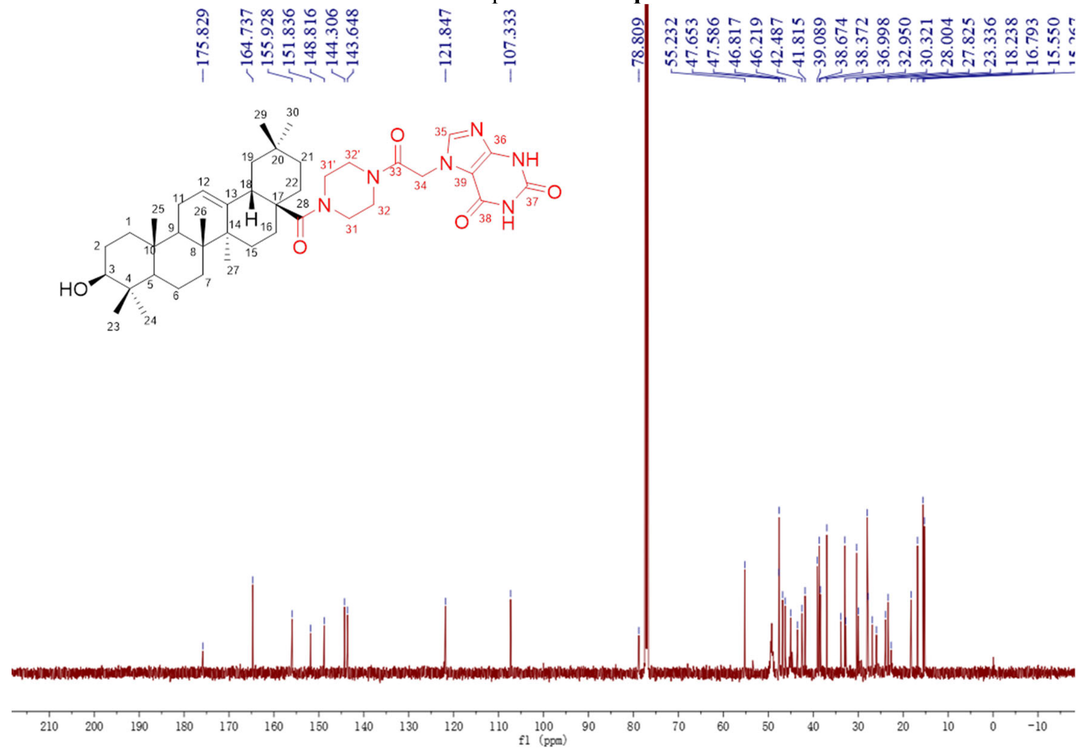

^{13}C spectrum of **3q**

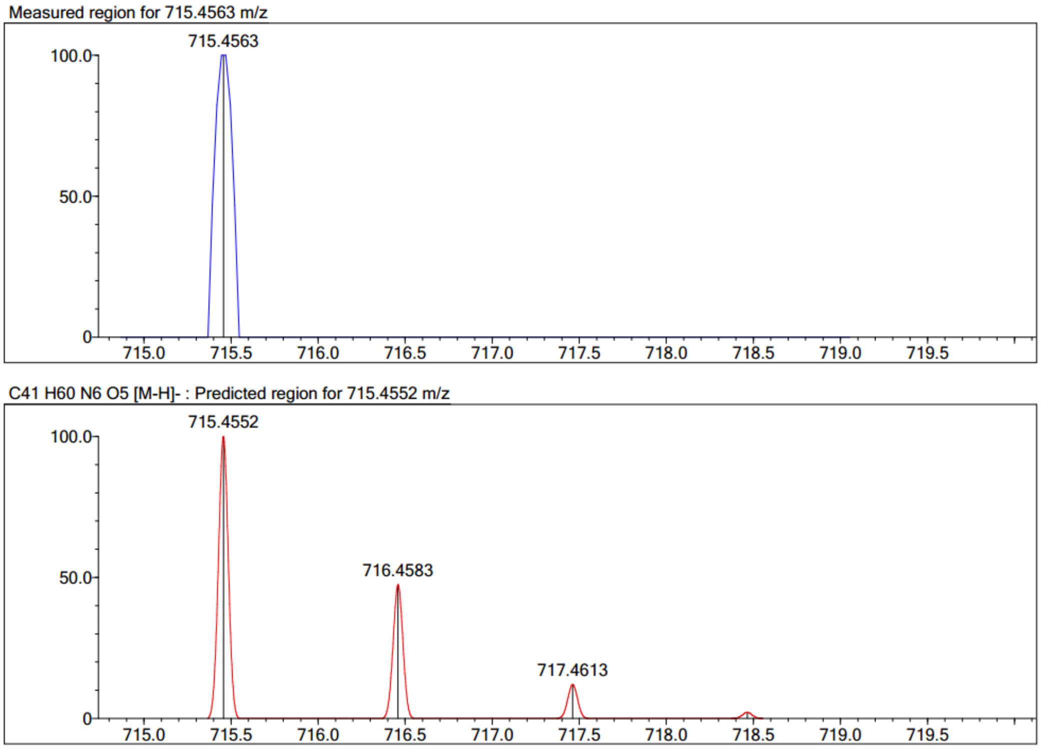

Rank	Score	Formula (M)	Ion	Meas. m/z	Pred. m/z	Df. (mDa)	Df. (ppm)	Iso	DBE
7	0.00	C41 H60 N6 O5	[M-H]-	715.4563	715.4552	1.1	1.54	0.00	15.0

HRMS spectrum of **3q**

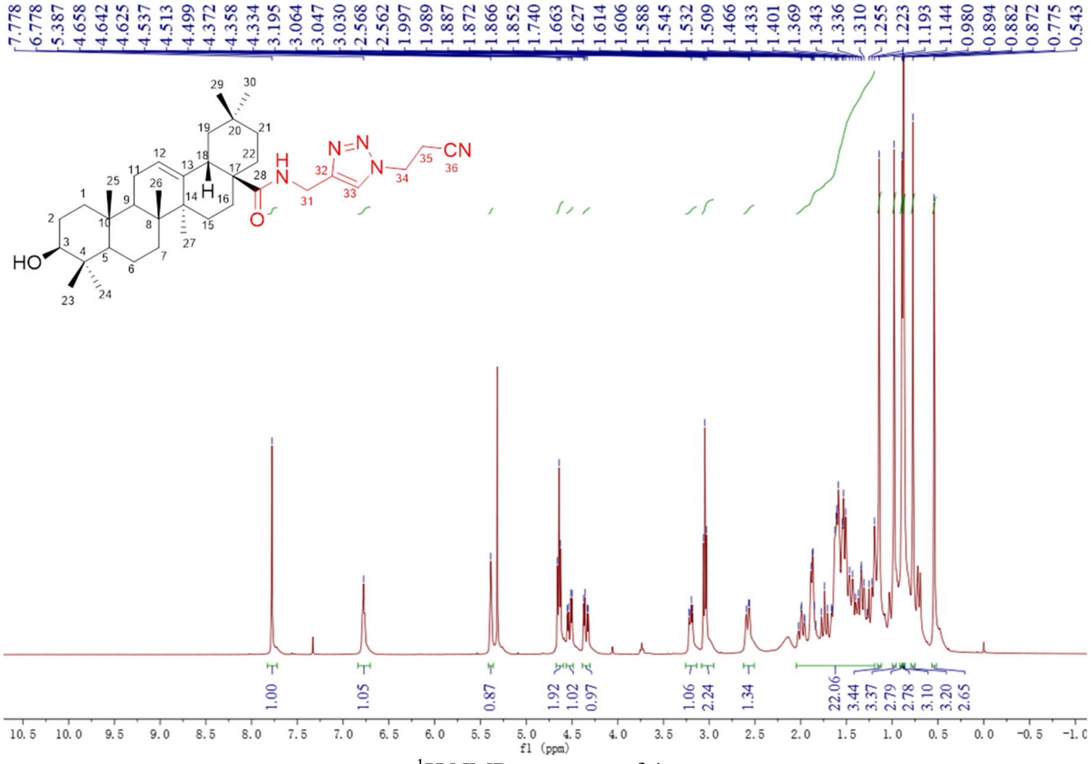

^{1}H NMR spectrum of **4a**

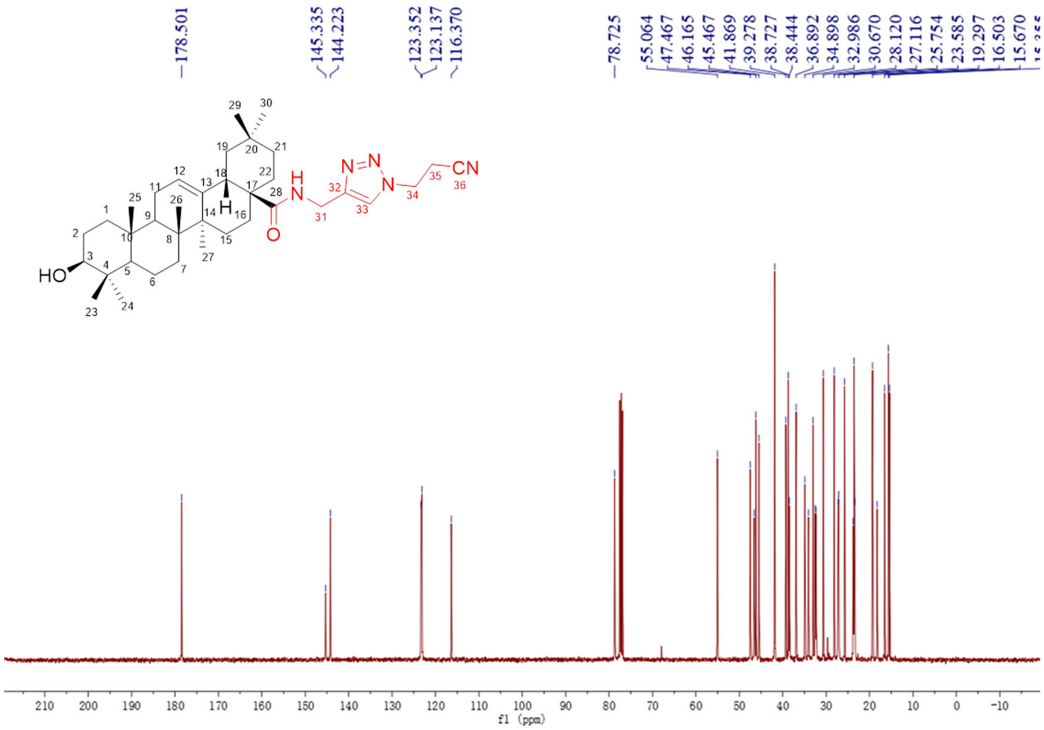

^{13}C NMR spectrum of **4a**

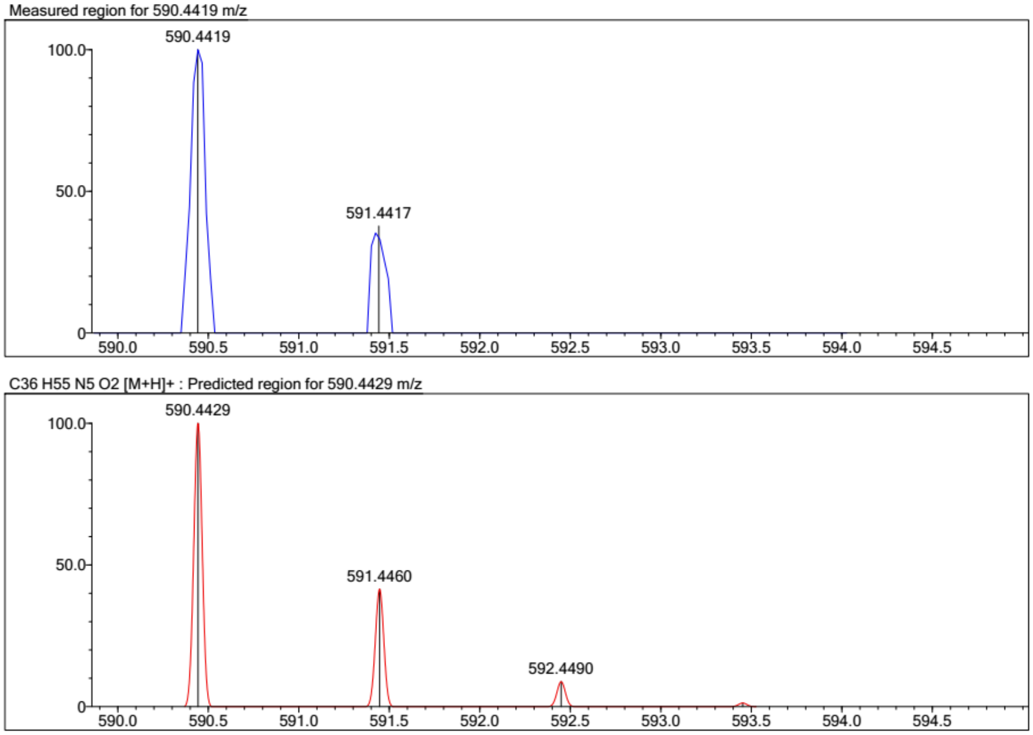

Rank	Score	Formula (M)	Ion	Meas. m/z	Pred. m/z	Df. (mDa)	Df. (ppm)	Iso	DBE
6	0.00	C36 H55 N5 O2	[M+H]+	590.4419	590.4429	-1.0	-1.69	0.00	12.0

HRMS spectrum of **4a**

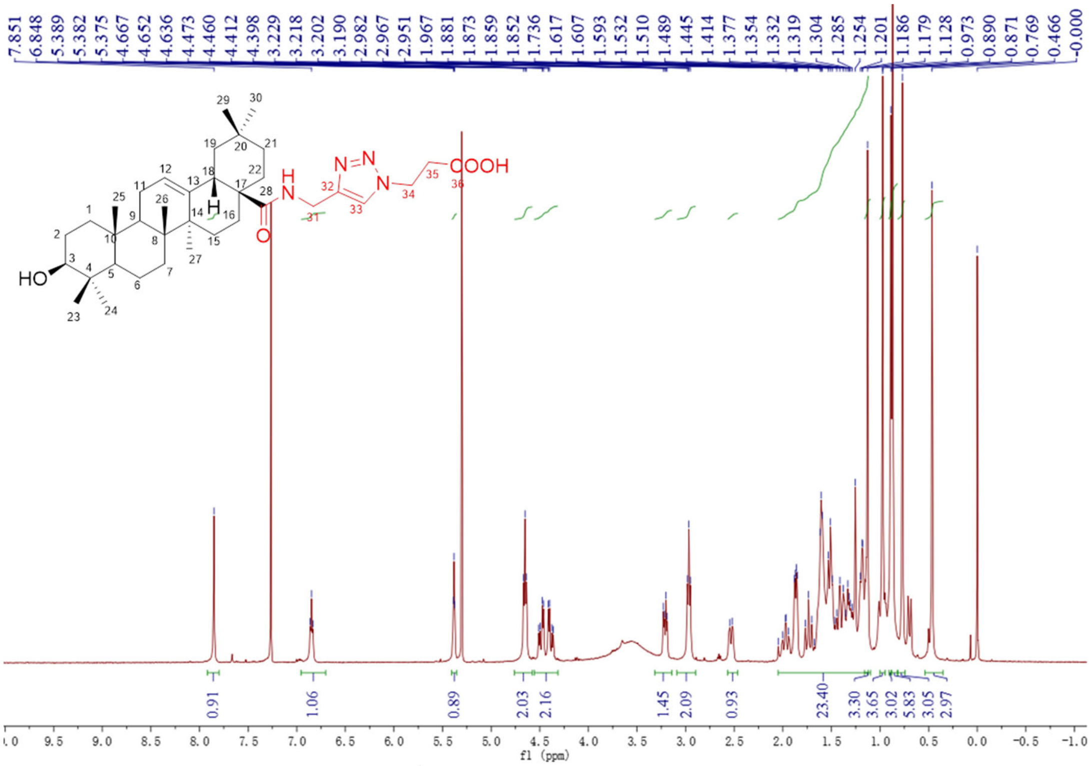

^{1}H NMR spectrum of **4b**

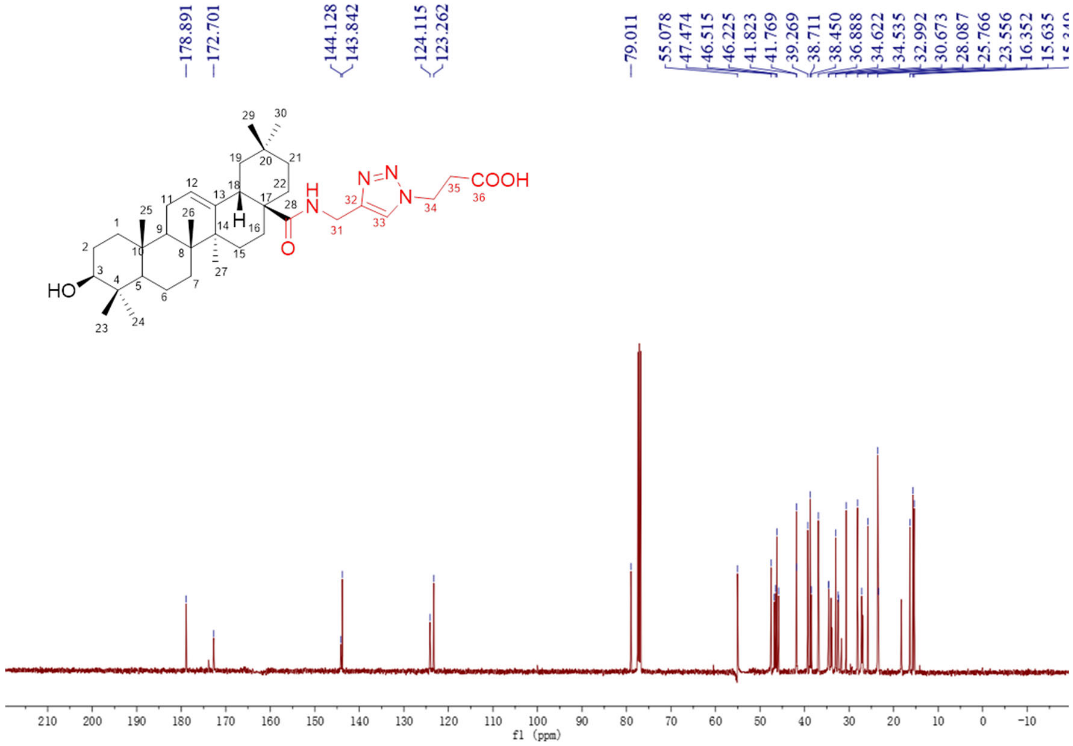

^{13}C NMR spectrum of **4b**

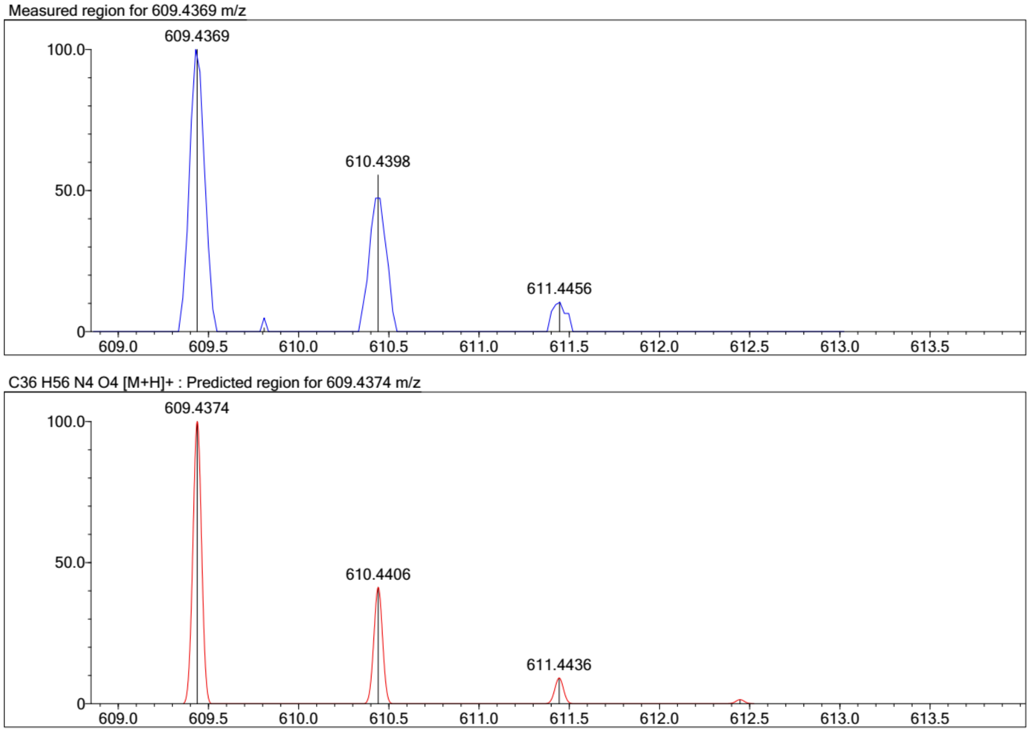

Rank	Score	Formula (M)	Ion	Meas. m/z	Pred. m/z	Df. (mDa)	Df. (ppm)	Iso	DBE
2	80.13	C36 H56 N4 O4	[M+H]+	609.4369	609.4374	-0.5	-0.82	80.13	11.0

HRMS spectrum of **4b**

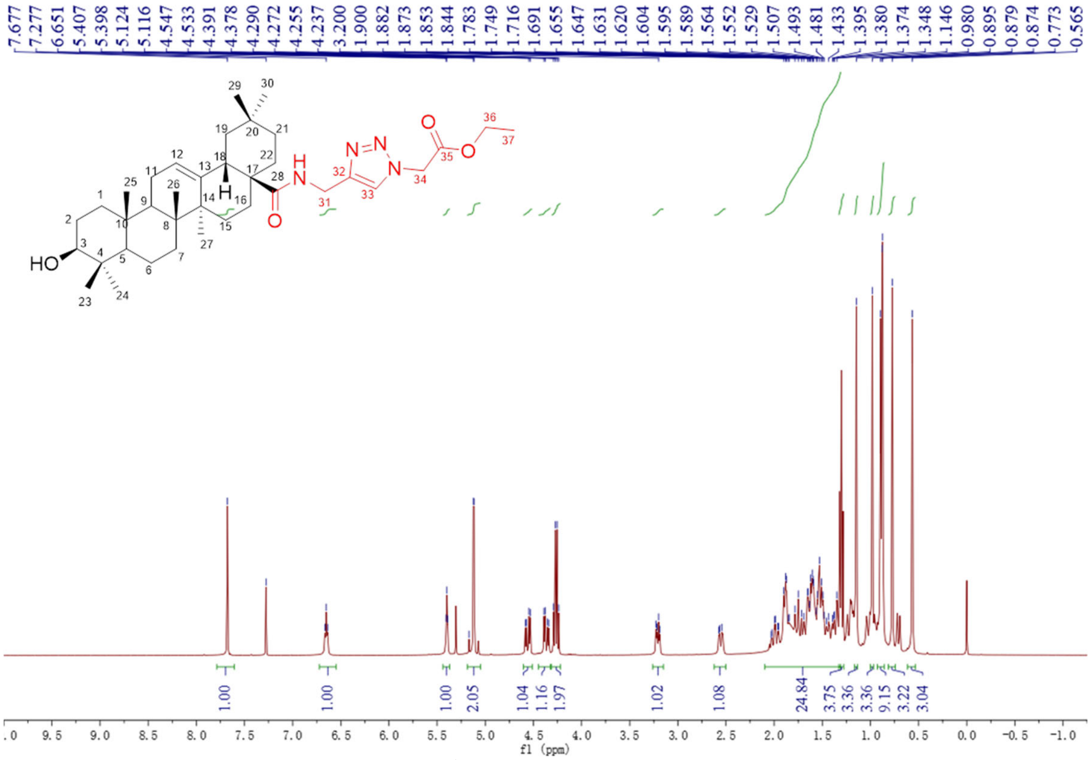

^{1}H spectrum of **4c**

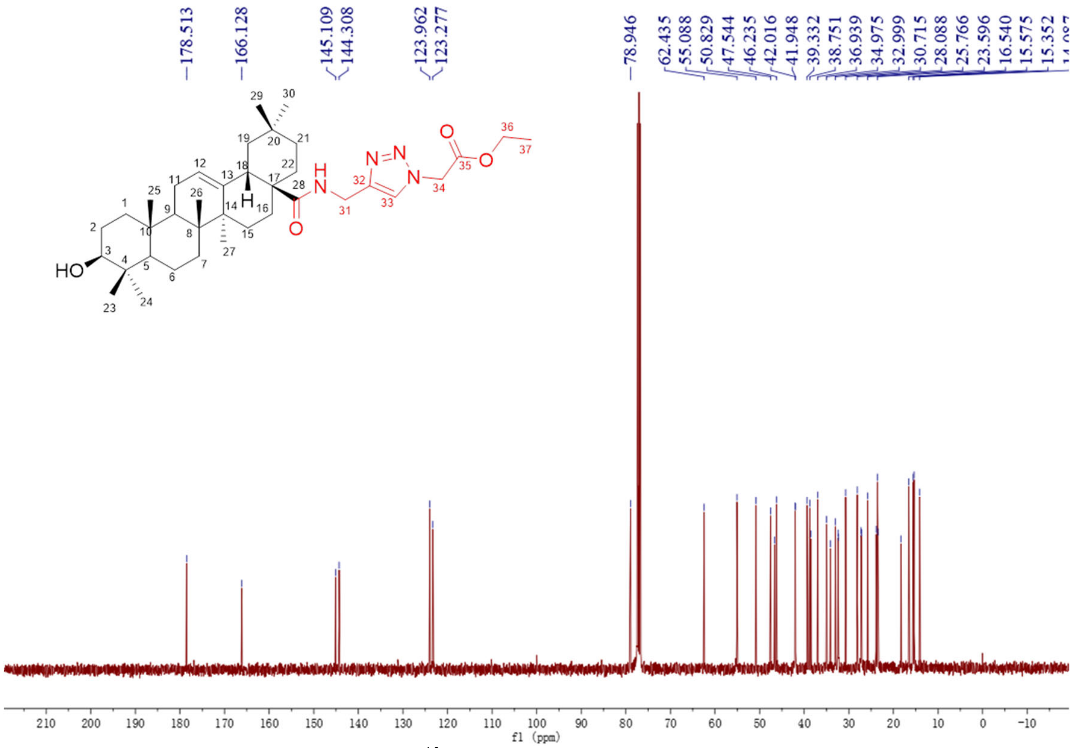

^{13}C spectrum of **4c**

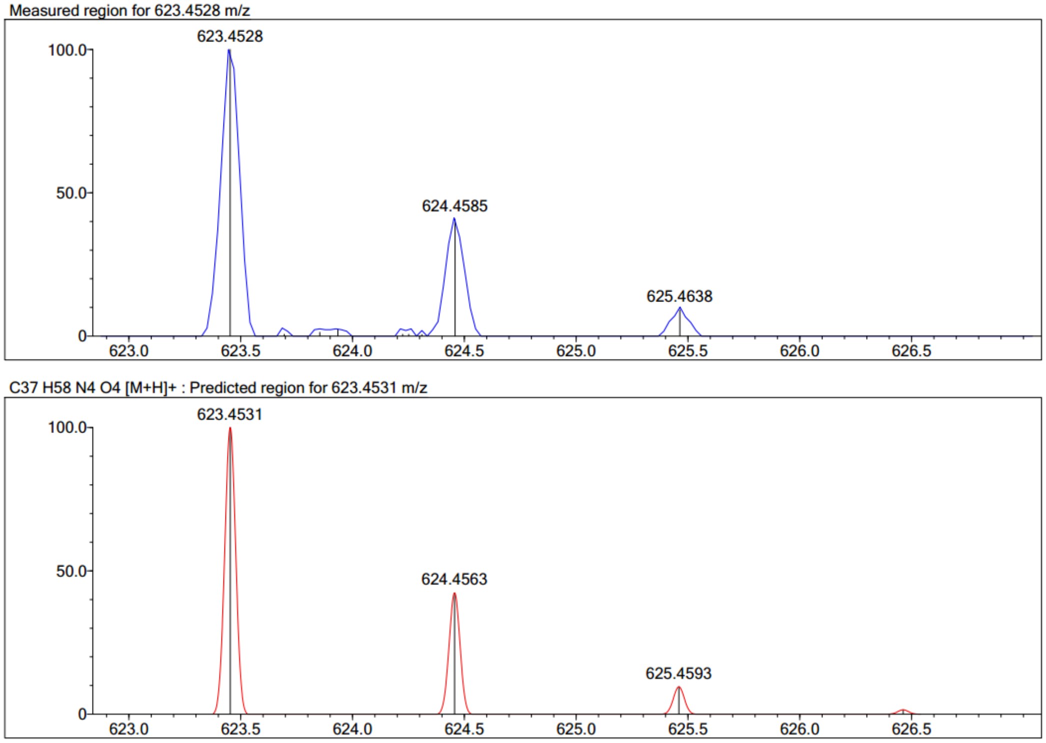

Rank	Score	Formula (M)	Ion	Meas. m/z	Pred. m/z	Df. (mDa)	Df. (ppm)	Iso	DBE
1	100.00	C37 H58 N4 O4	[M+H]+	623.4528	623.4531	-0.3	-0.48	100.00	11.0

HRMS spectrum of **4c**

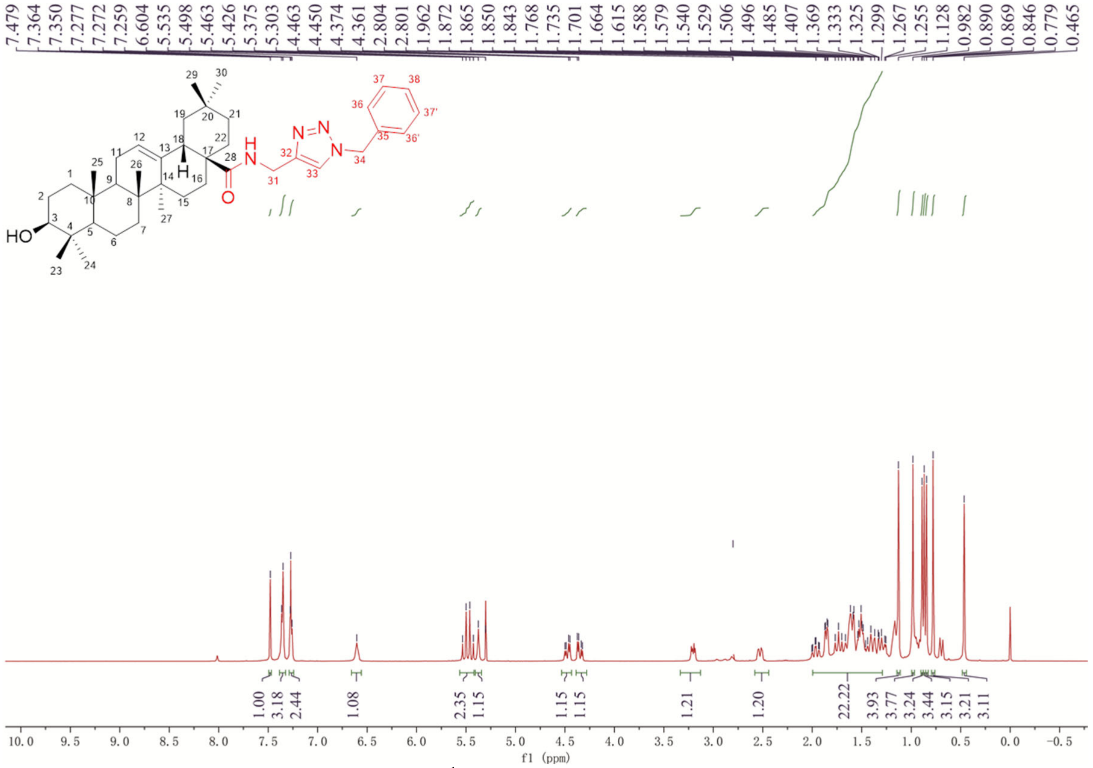

^{1}H spectrum of **4d**

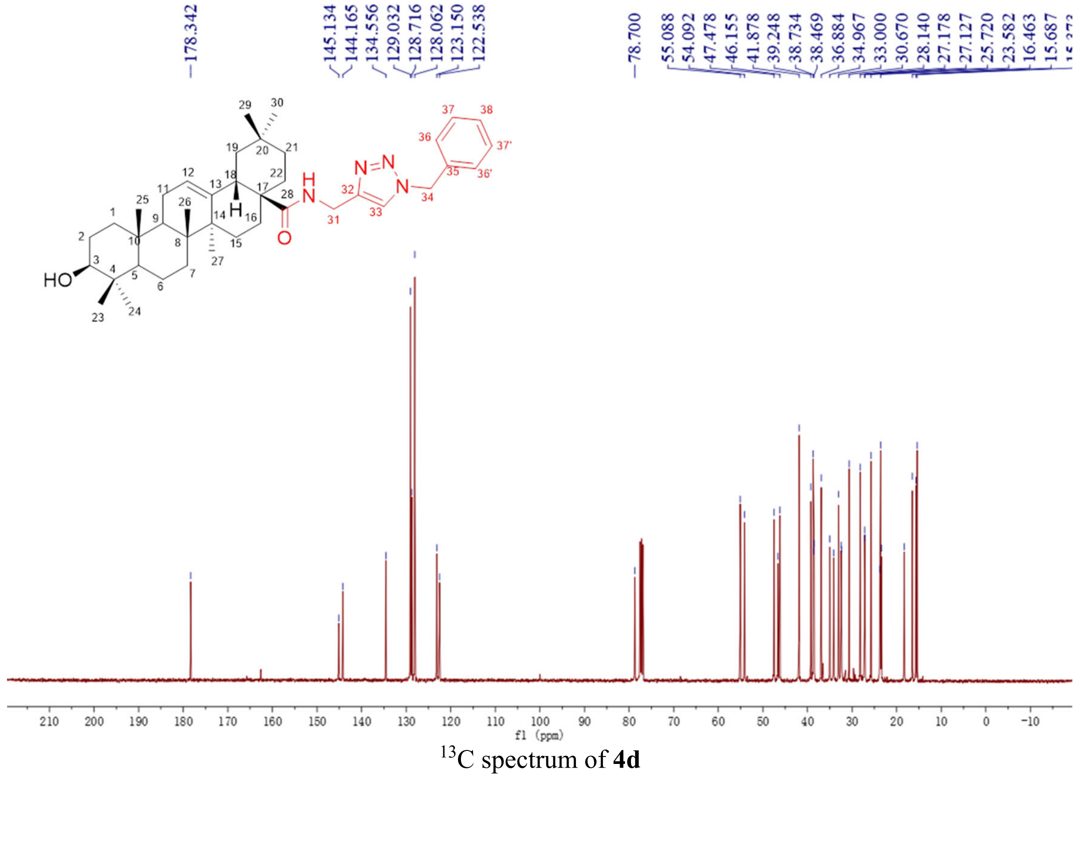

^{13}C spectrum of **4d**

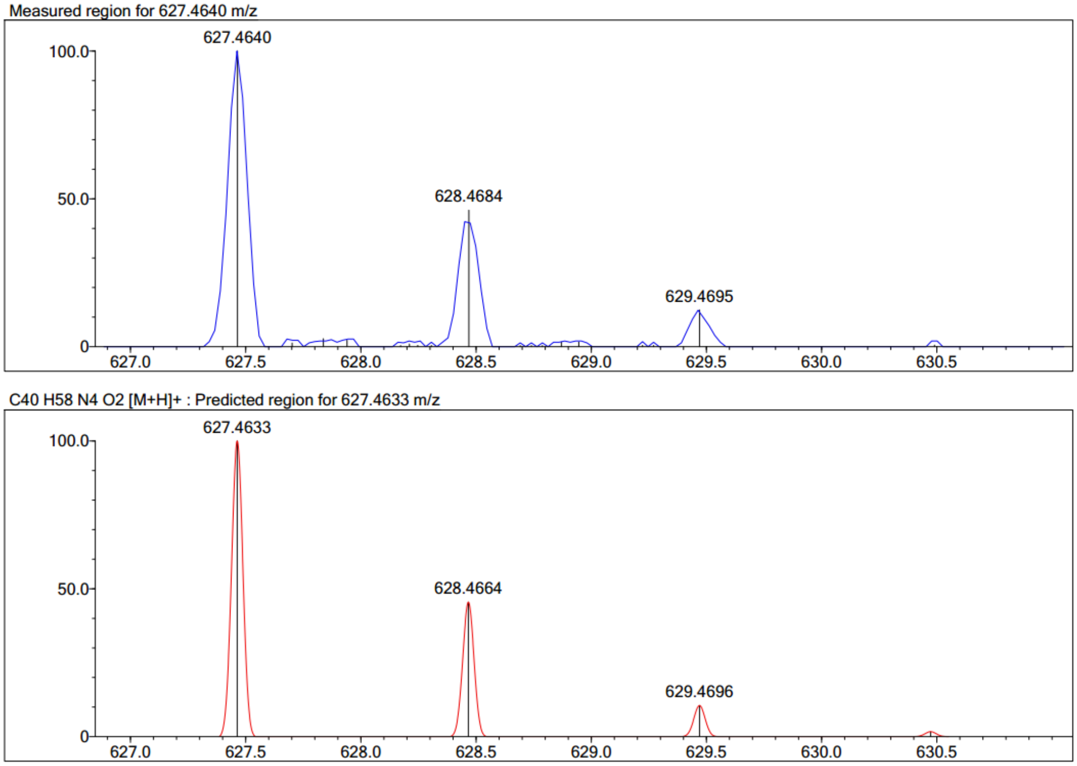

Rank	Score	Formula (M)	Ion	Meas. m/z	Pred. m/z	Df. (mDa)	Df. (ppm)	Iso	DBE
1	76.46	C40 H58 N4 O2	[M+H]+	627.4640	627.4633	0.7	1.12	76.69	14.0

HRMS spectrum of **4d**

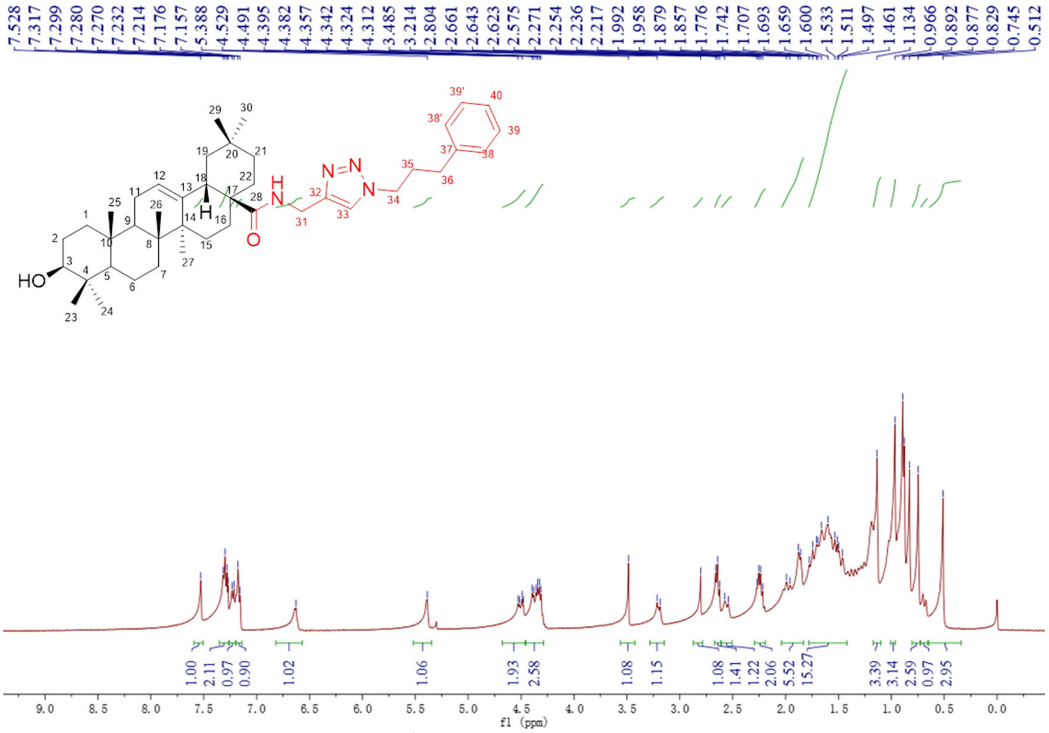

^{1}H NMR spectrum of **4e**

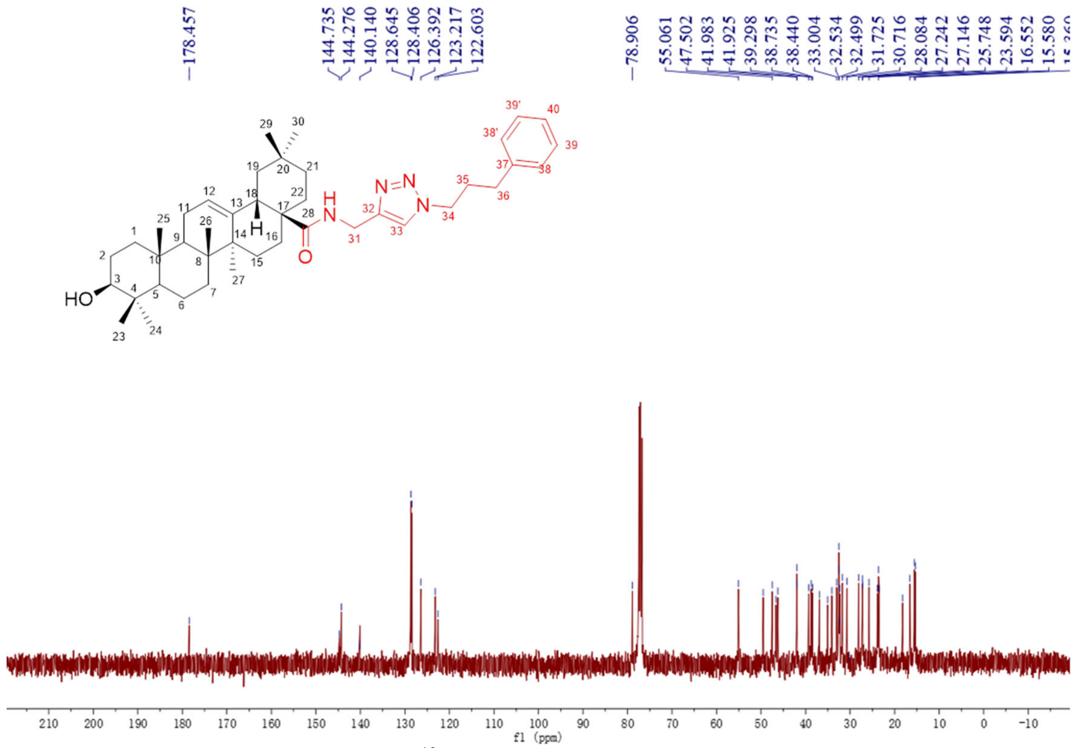

^{13}C NMR spectrum of **4e**

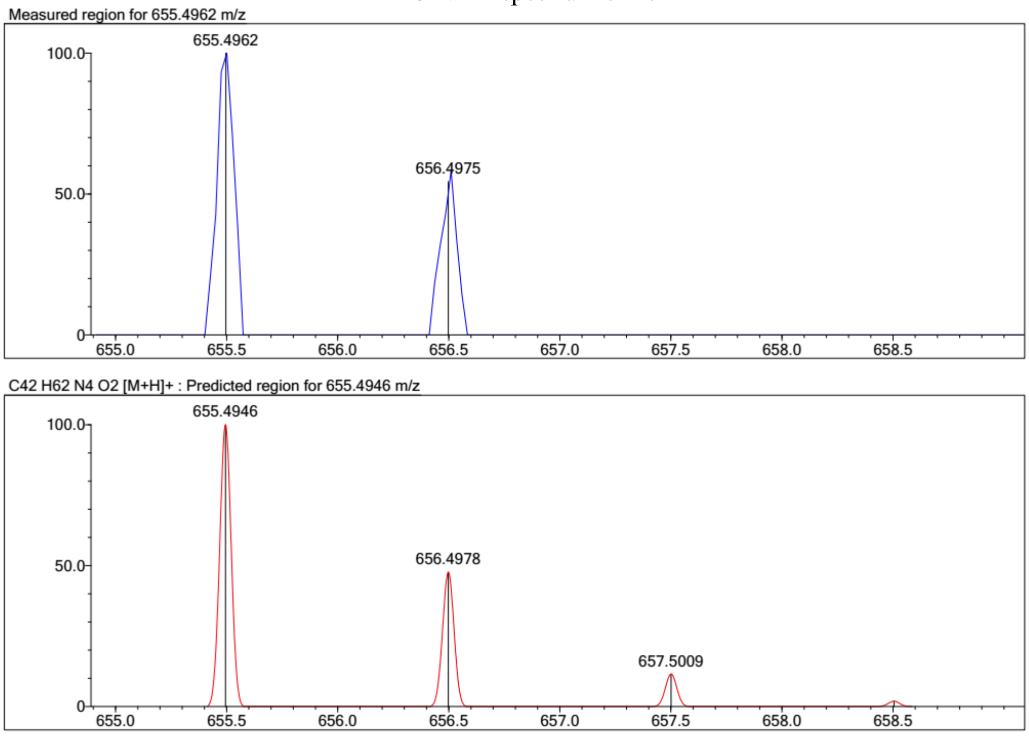

Rank	Score	Formula (M)	Ion	Meas. m/z	Pred. m/z	Df. (mDa)	Df. (ppm)	Iso	DBE
2	0.00	C42 H62 N4 O2	[M+H]+	655.4962	655.4946	1.6	2.44	0.00	14.0

HRMS spectrum of **4e**

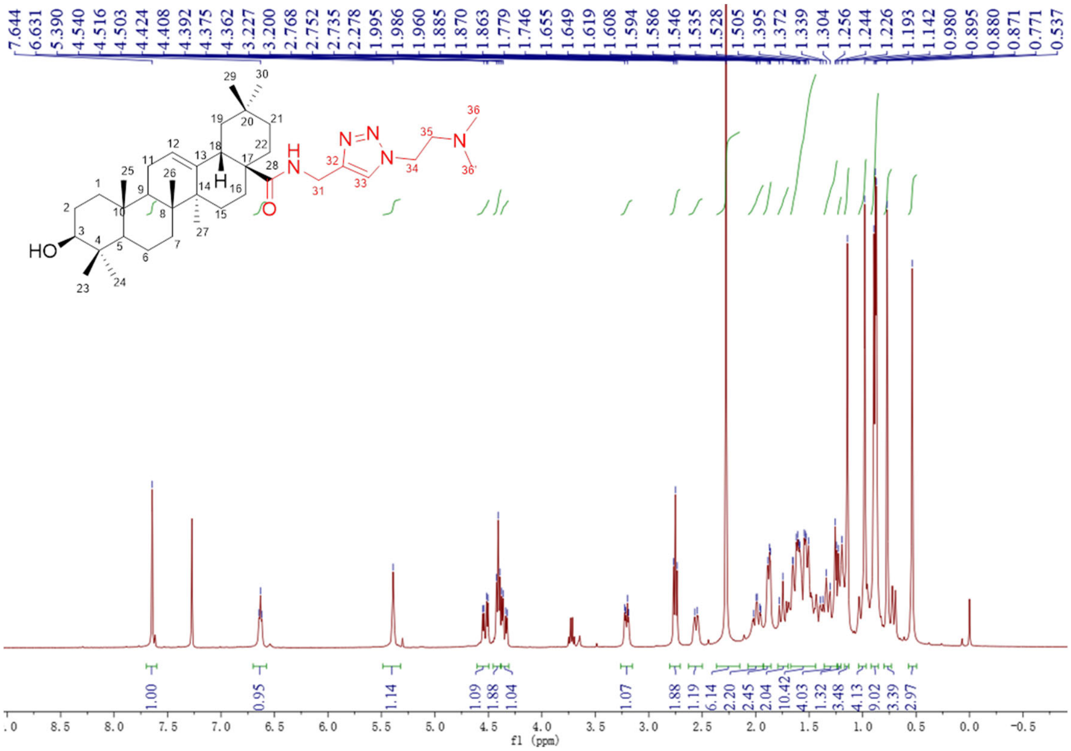

^{1}H spectrum of **4f**

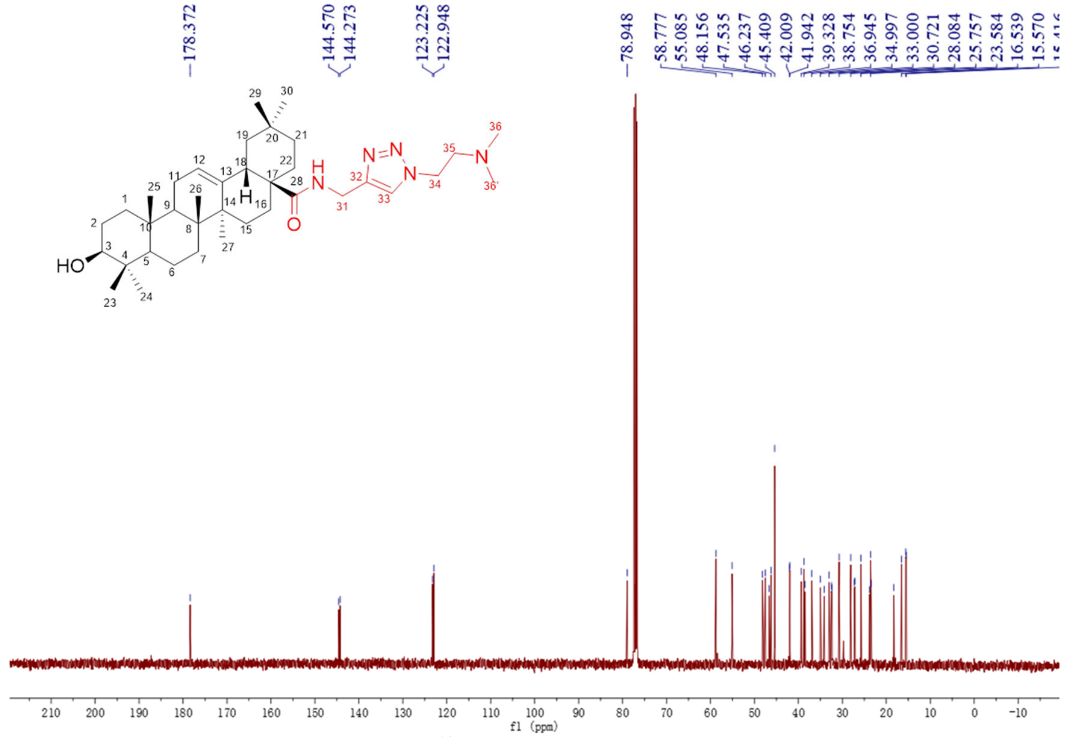

^{13}C spectrum of **4f**

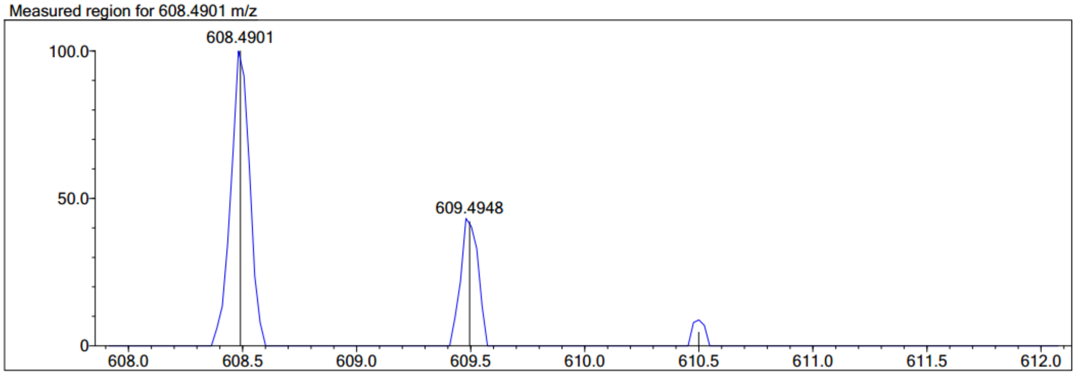

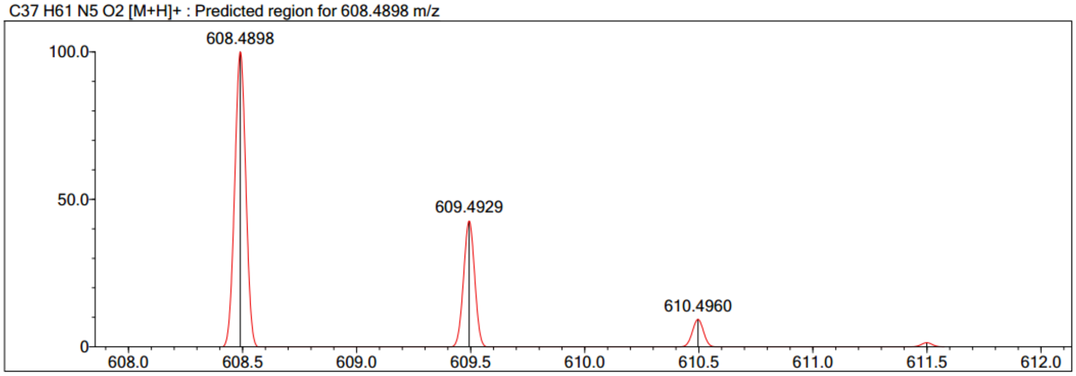

Rank	Score	Formula (M)	Ion	Meas. m/z	Pred. m/z	Df. (mDa)	Df. (ppm)	Iso	DBE
1	97.27	C37 H61 N5 O2	[M+H]+	608.4901	608.4898	0.3	0.49	97.27	10.0

HRMS spectrum of **4f**

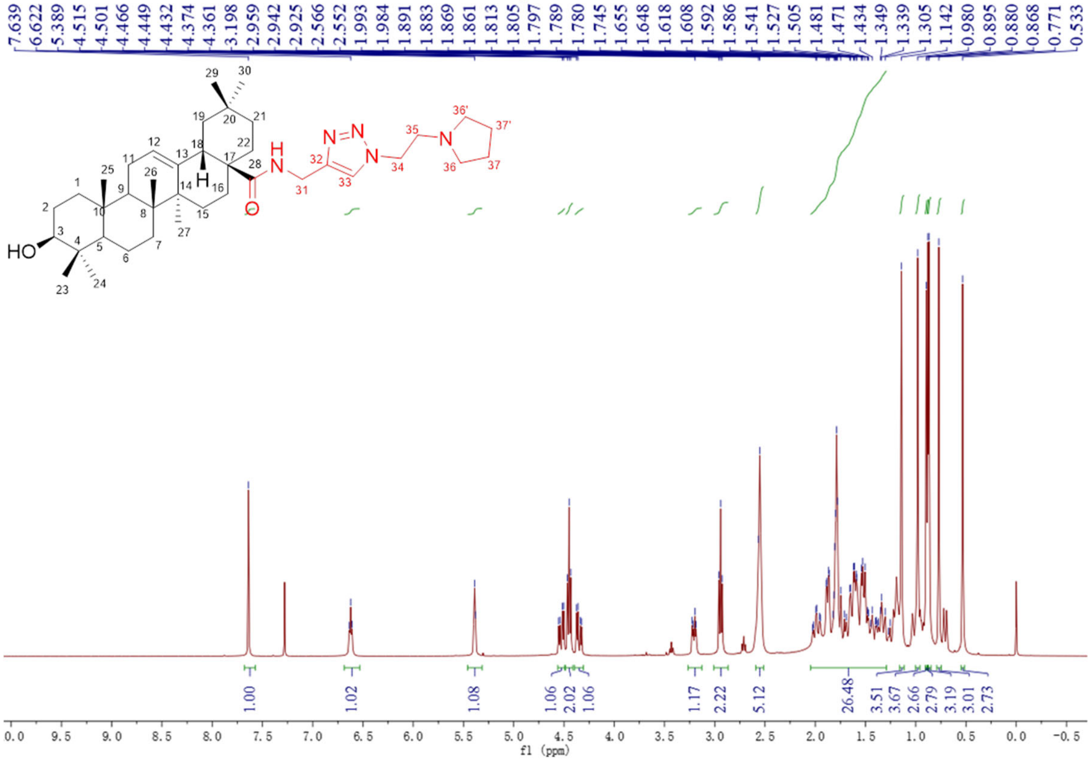

^{1}H spectrum of **4g**

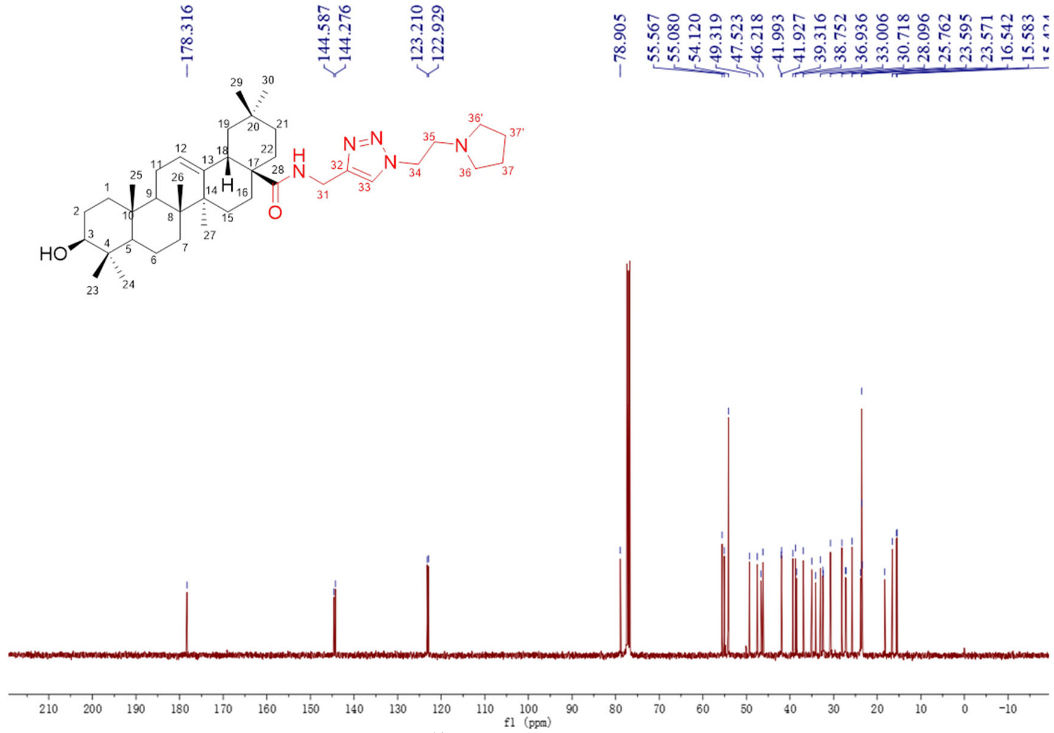

^{13}C spectrum of **4g**

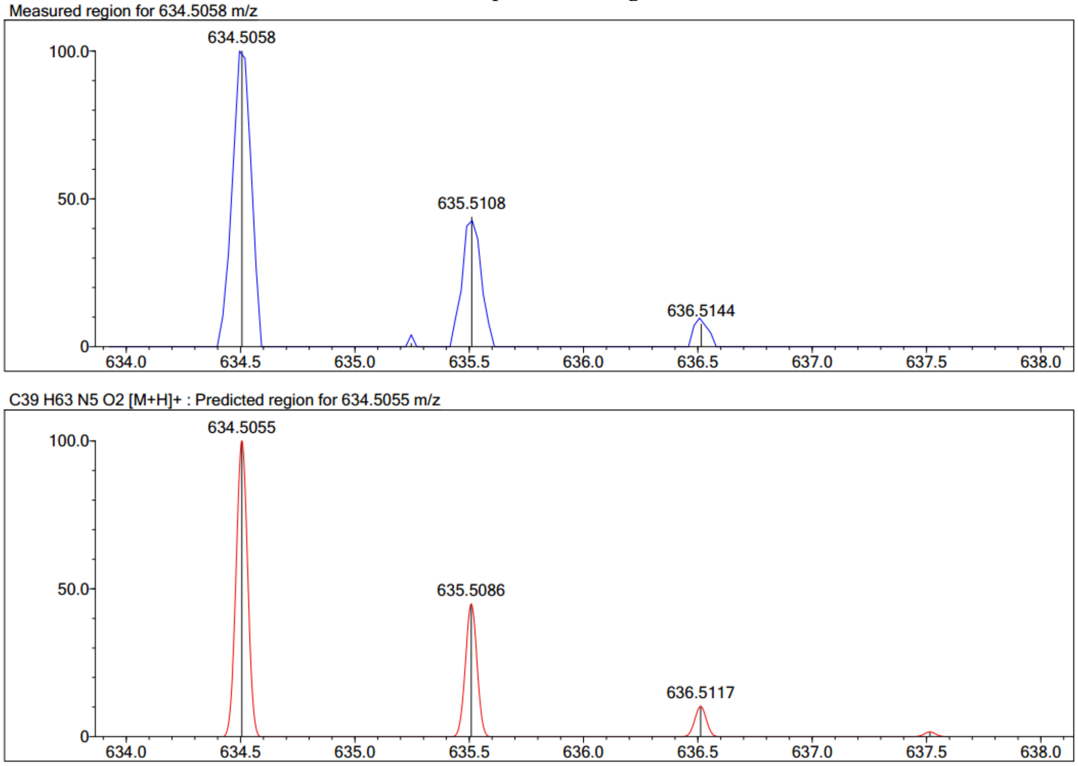

Rank	Score	Formula (M)	Ion	Meas. m/z	Pred. m/z	Df. (mDa)	Df. (ppm)	Iso	DBE
1	98.60	C39 H63 N5 O2	[M+H]+	634.5058	634.5055	0.3	0.47	98.60	11.0

HRMS spectrum of **4g**

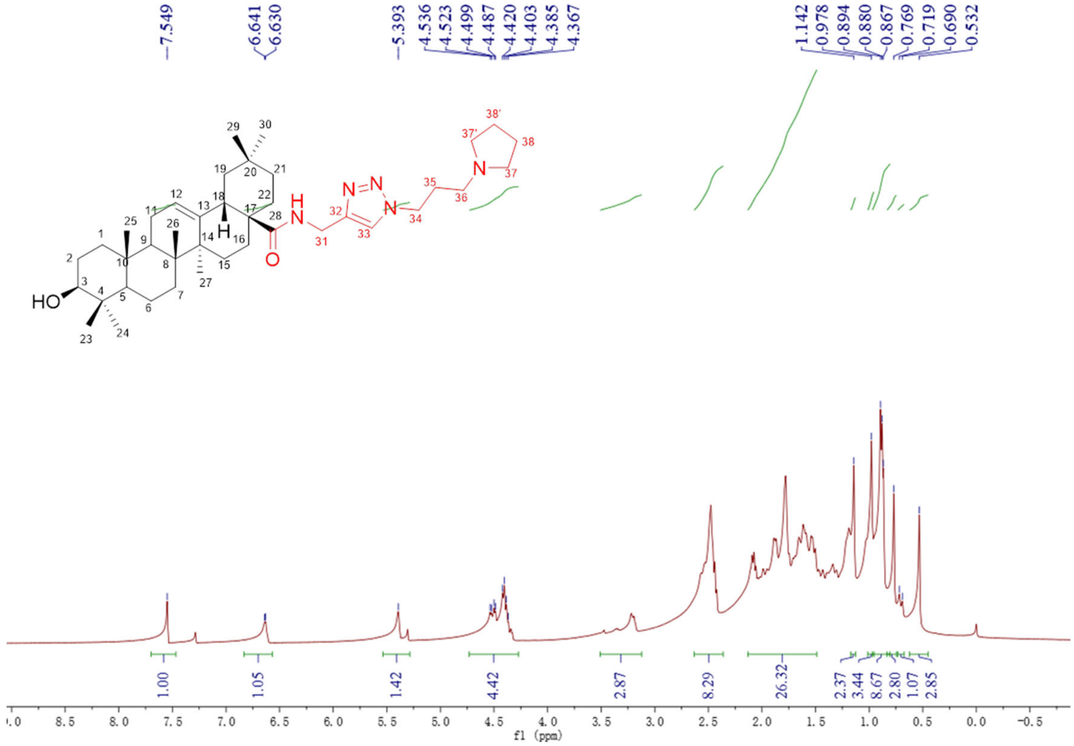

^{1}H NMR spectrum of **4h**

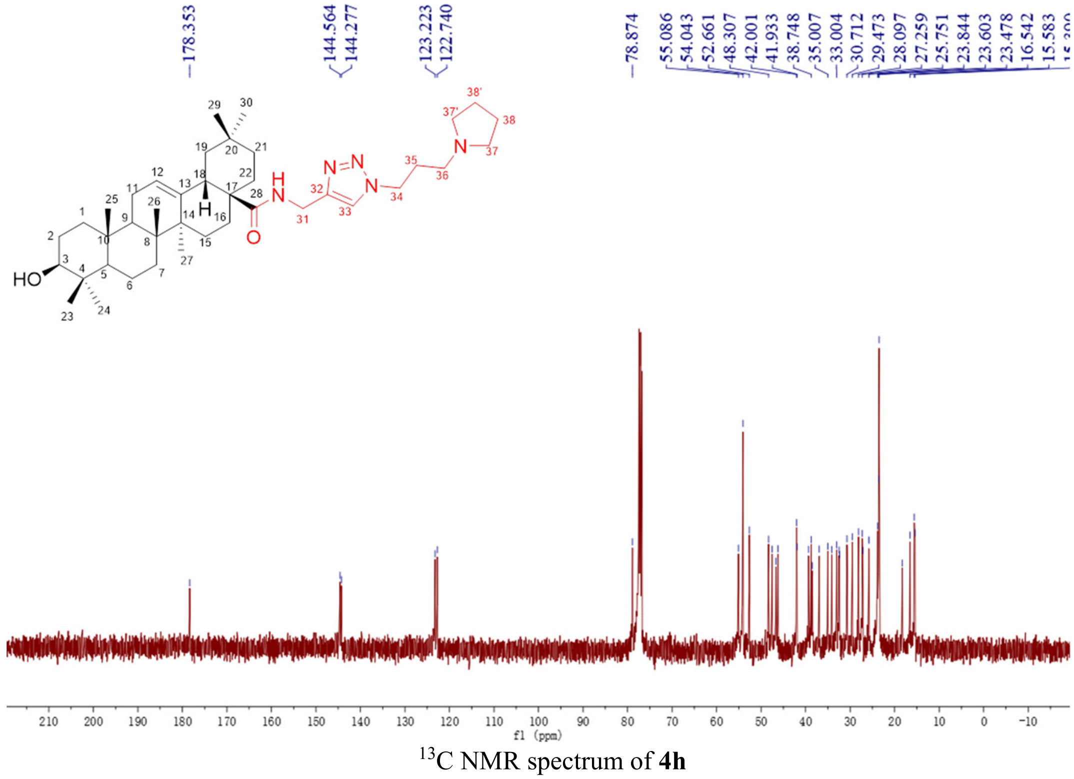

^{13}C NMR spectrum of **4h**

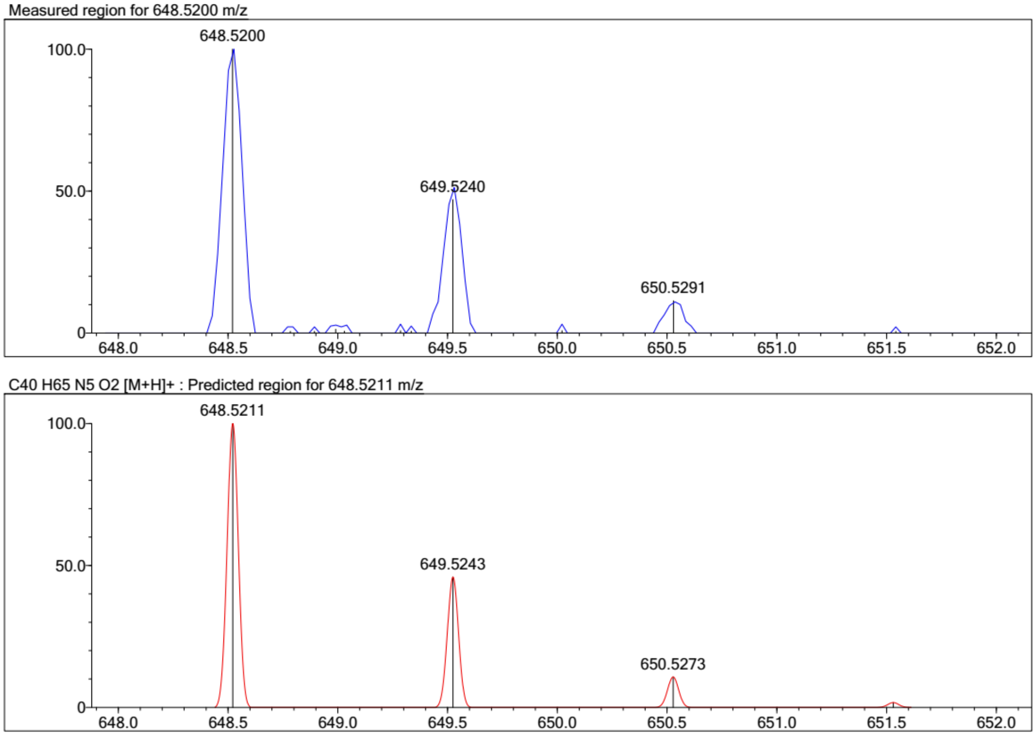

Rank	Score	Formula (M)	Ion	Meas. m/z	Pred. m/z	Df. (mDa)	Df. (ppm)	Iso	DBE
1	81.62	C40 H65 N5 O2	[M+H]+	648.5200	648.5211	-1.1	-1.70	83.07	11.0

HRMS spectrum of **4h**

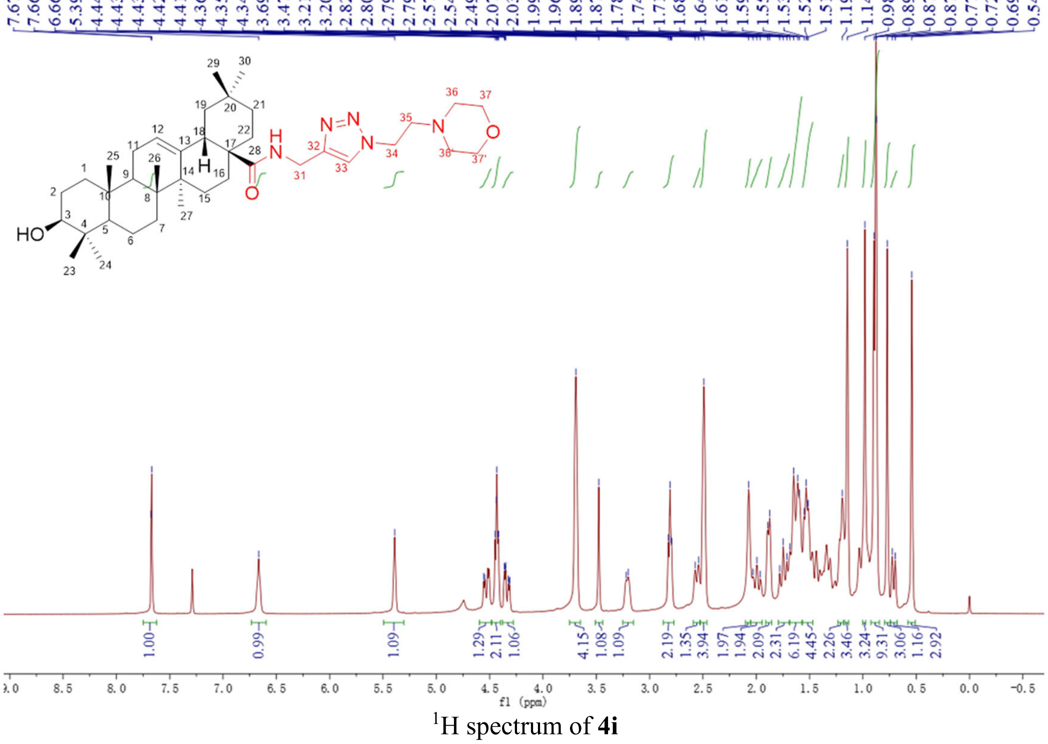

^{1}H spectrum of **4i**

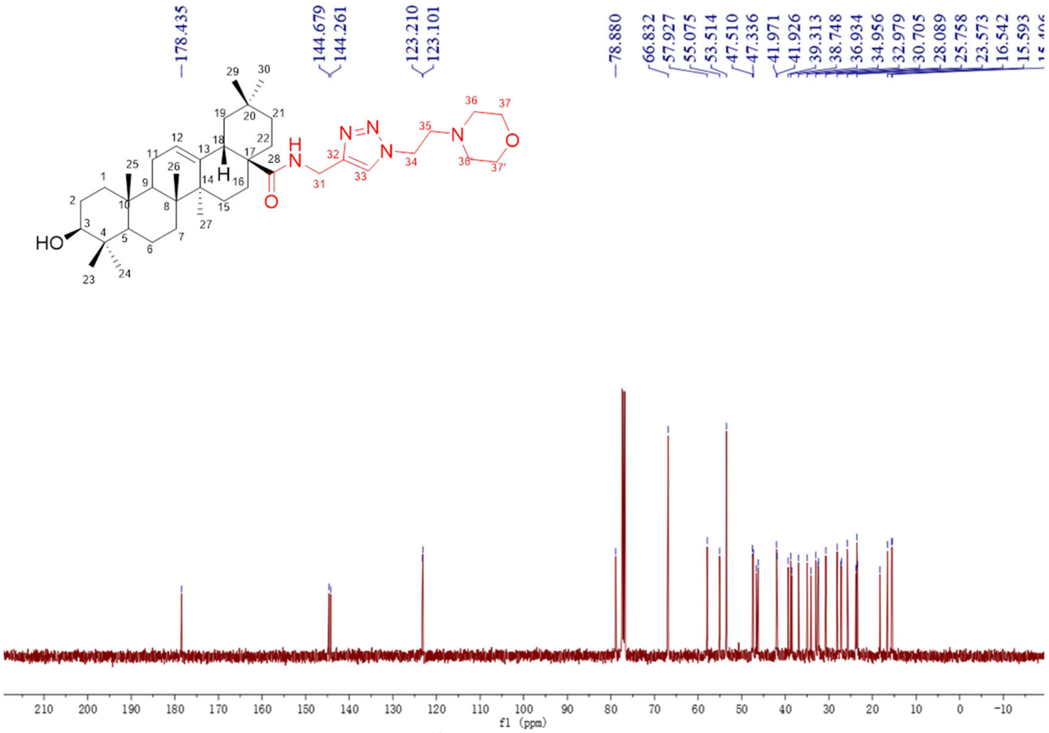

^{13}C spectrum of **4i**

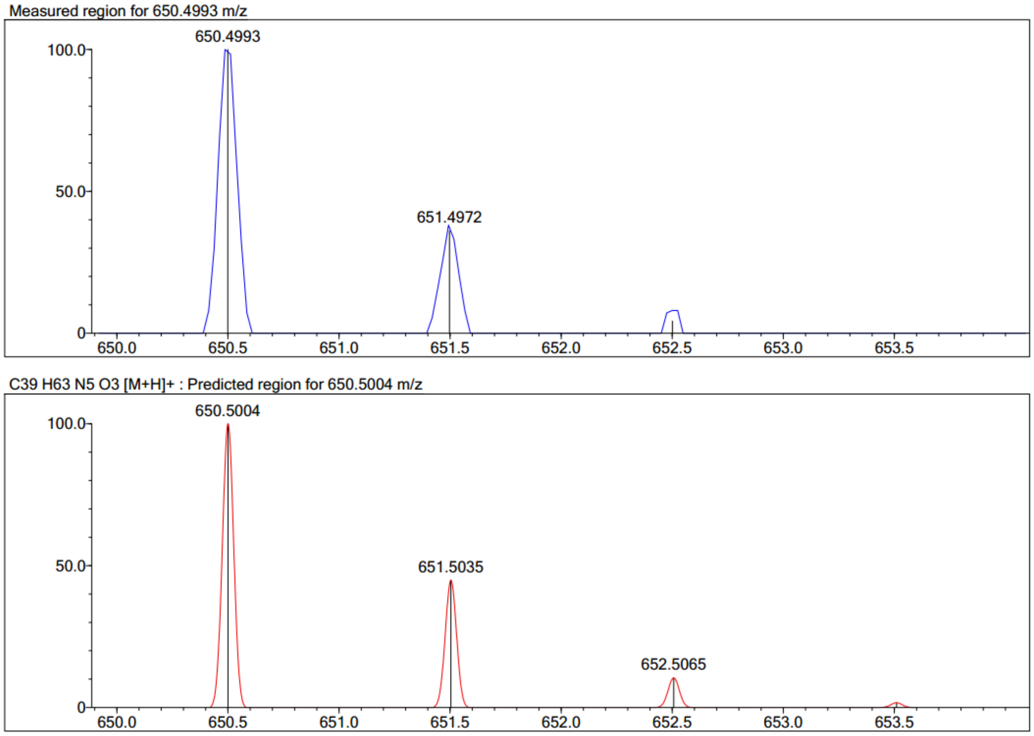

Rank	Score	Formula (M)	Ion	Meas. m/z	Pred. m/z	Df. (mDa)	Df. (ppm)	Iso	DBE
2	64.96	C39 H63 N5 O3	[M+H]+	650.4993	650.5004	-1.1	-1.69	66.10	11.0

HRMS spectrum of **4i**

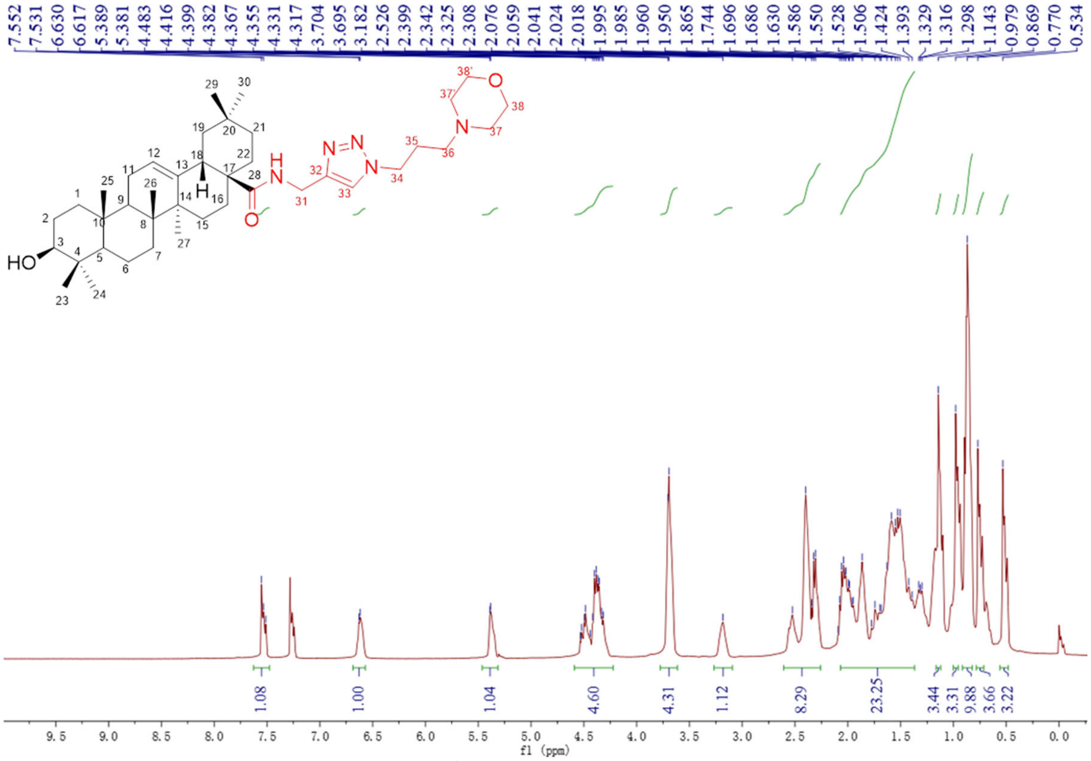

^{1}H spectrum of **4j**

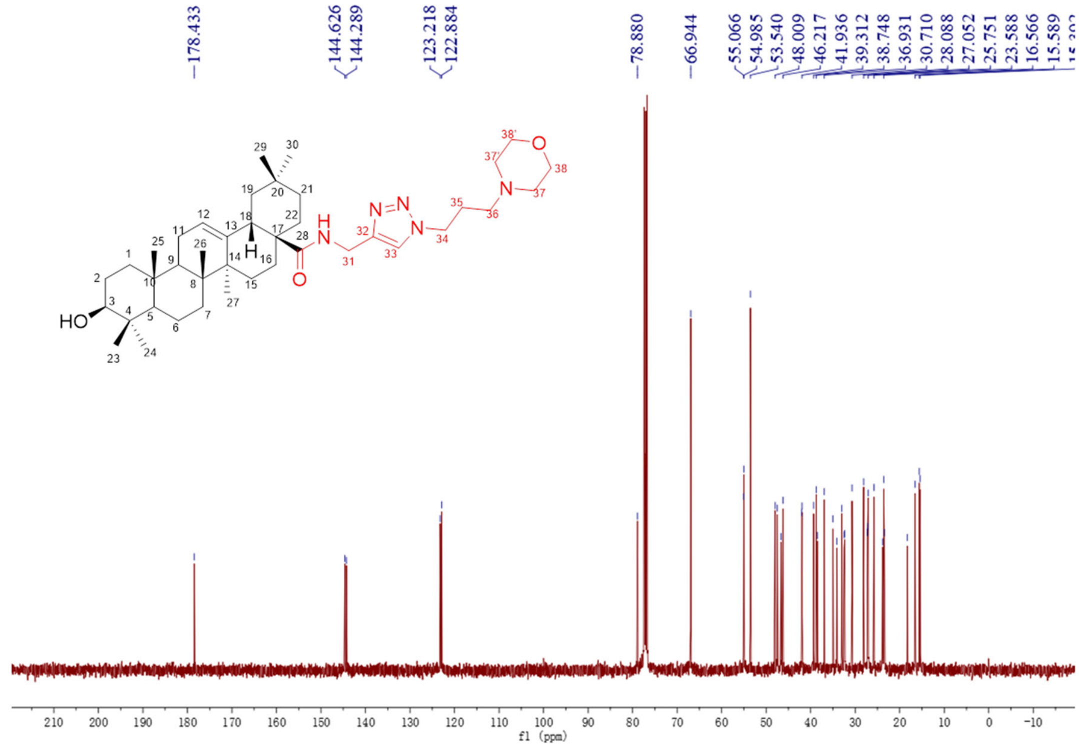

^{13}C spectrum of **4j**

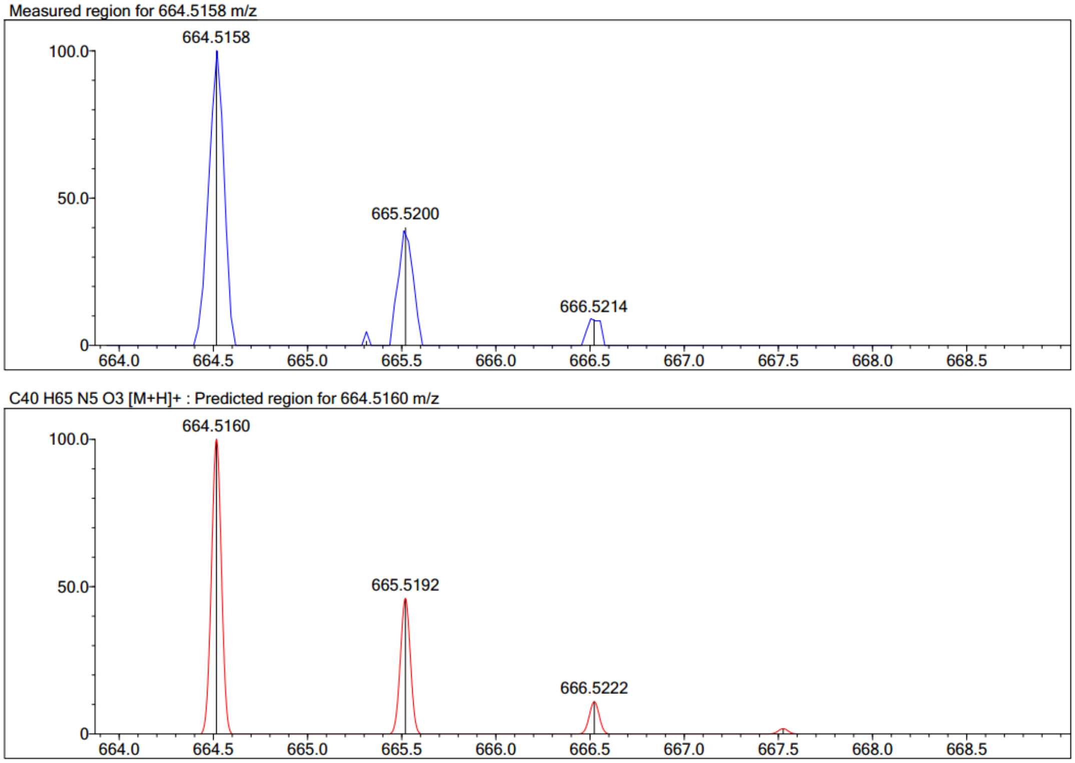

Rank	Score	Formula (M)	Ion	Meas. m/z	Pred. m/z	Df. (mDa)	Df. (ppm)	Iso	DBE
1	74.24	C40 H65 N5 O3	[M+H]+	664.5158	664.5160	-0.2	-0.30	74.24	11.0

HRMS spectrum of **4j**

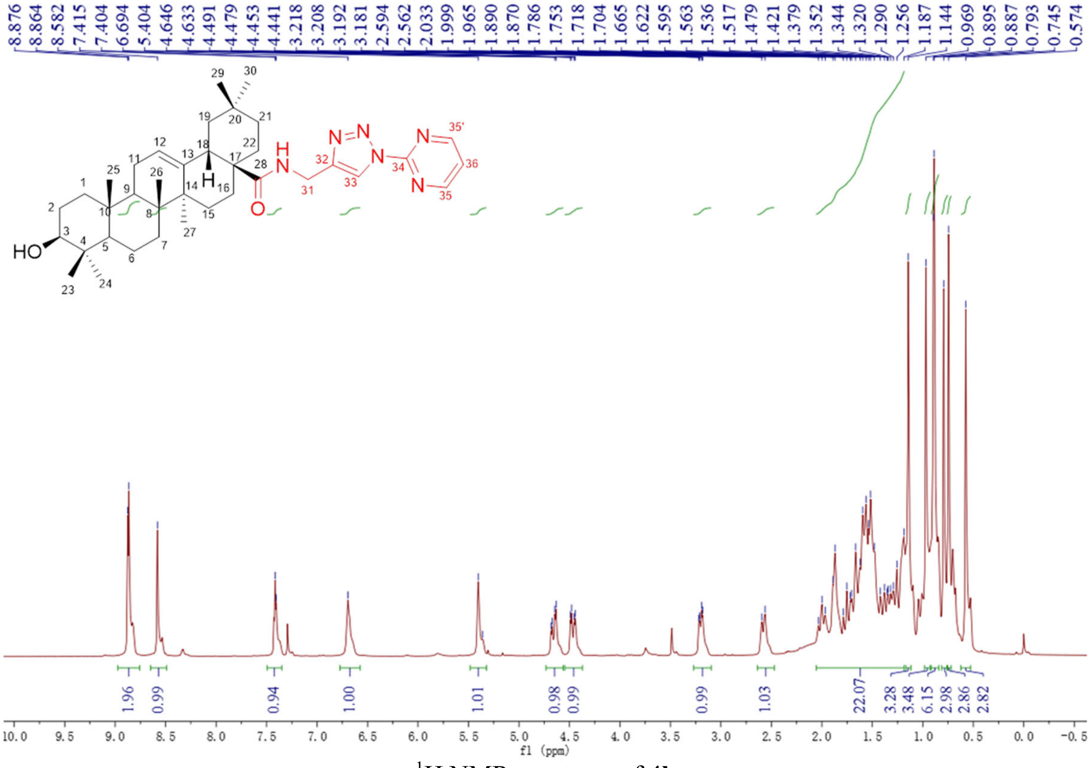

^{1}H NMR spectrum of **4k**

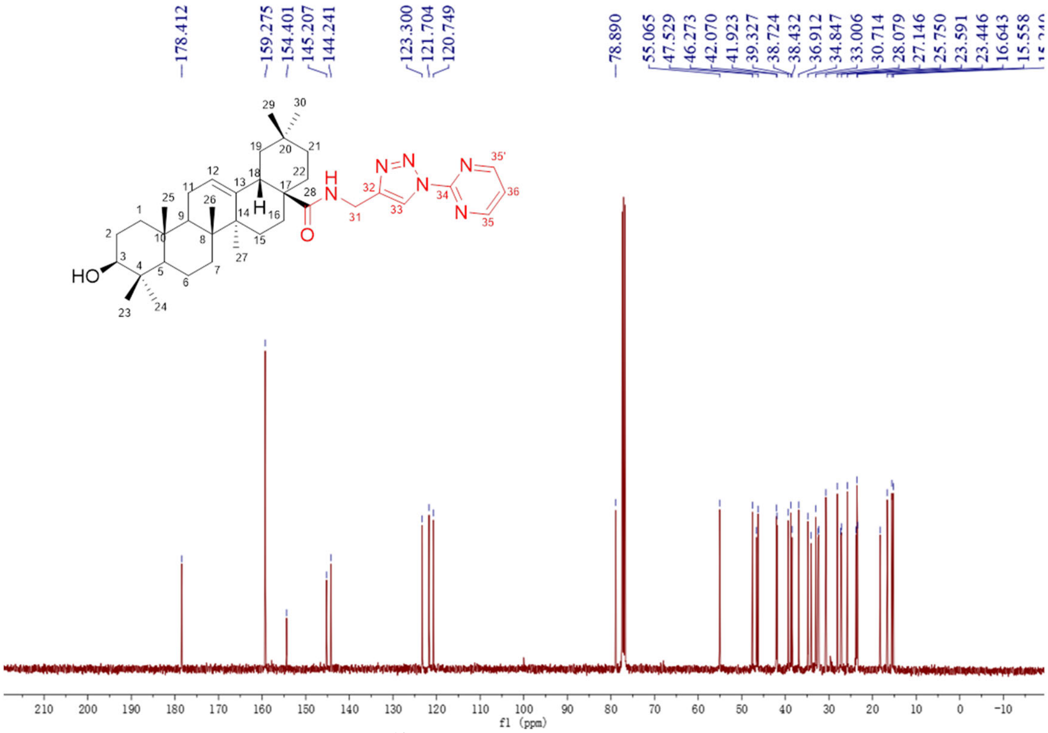

^{13}C NMR spectrum of **4k**

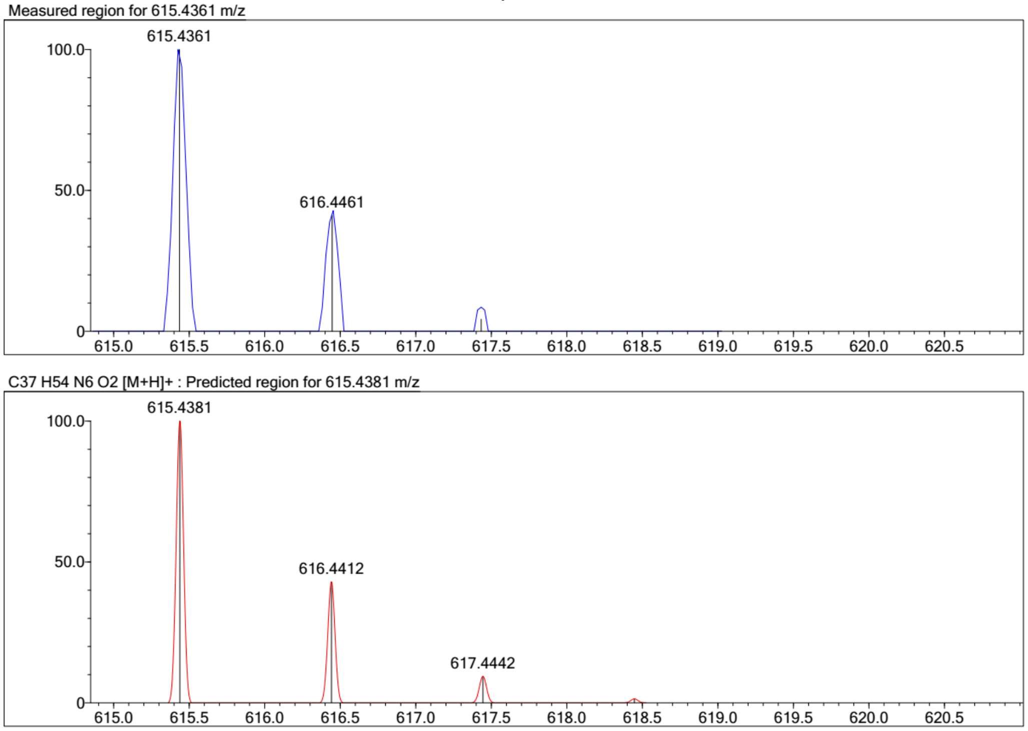

Rank	Score	Formula (M)	Ion	Meas. m/z	Pred. m/z	Df. (mDa)	Df. (ppm)	Iso	DBE
4	68.29	C37 H54 N6 O2	[M+H]+	615.4361	615.4381	-2.0	-3.25	72.36	14.0

HRMS spectrum of **4k**

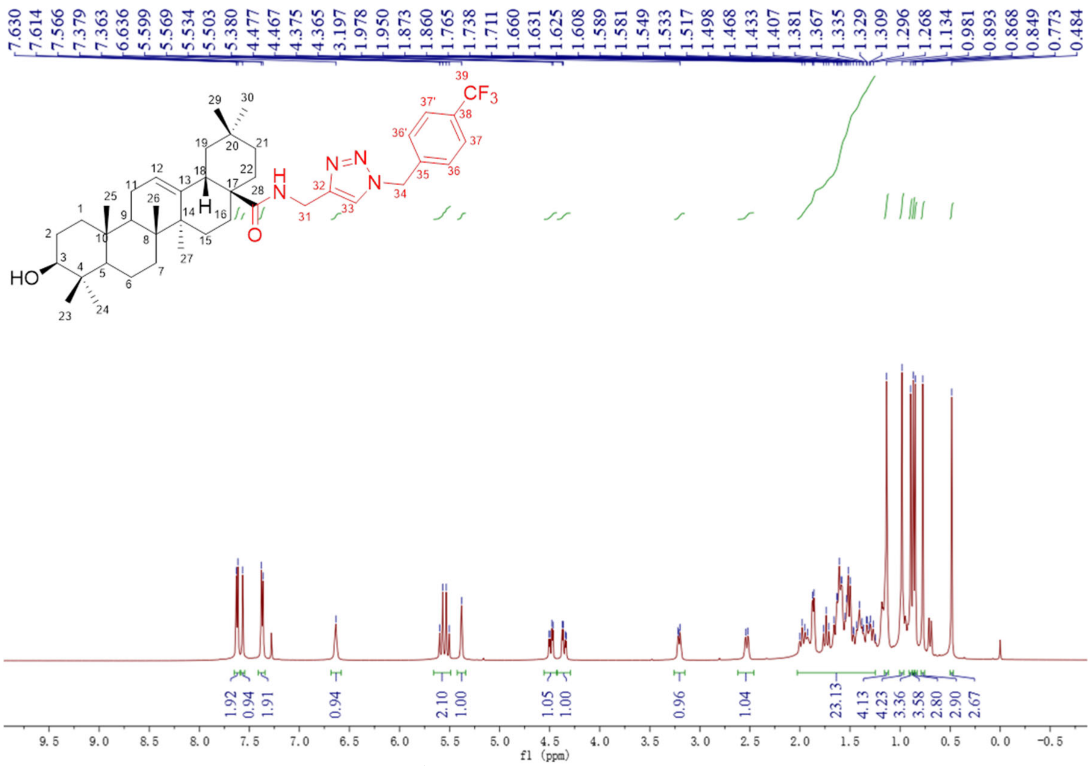

^{1}H NMR spectrum of **4l**

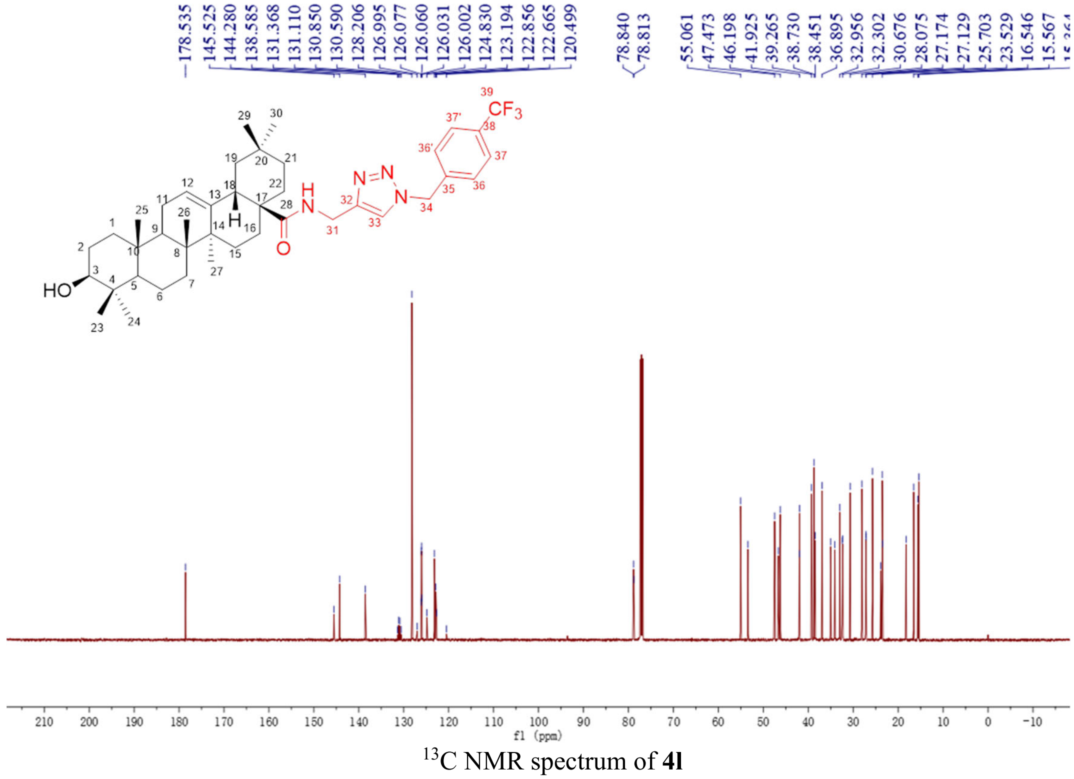

^{13}C NMR spectrum of **4l**

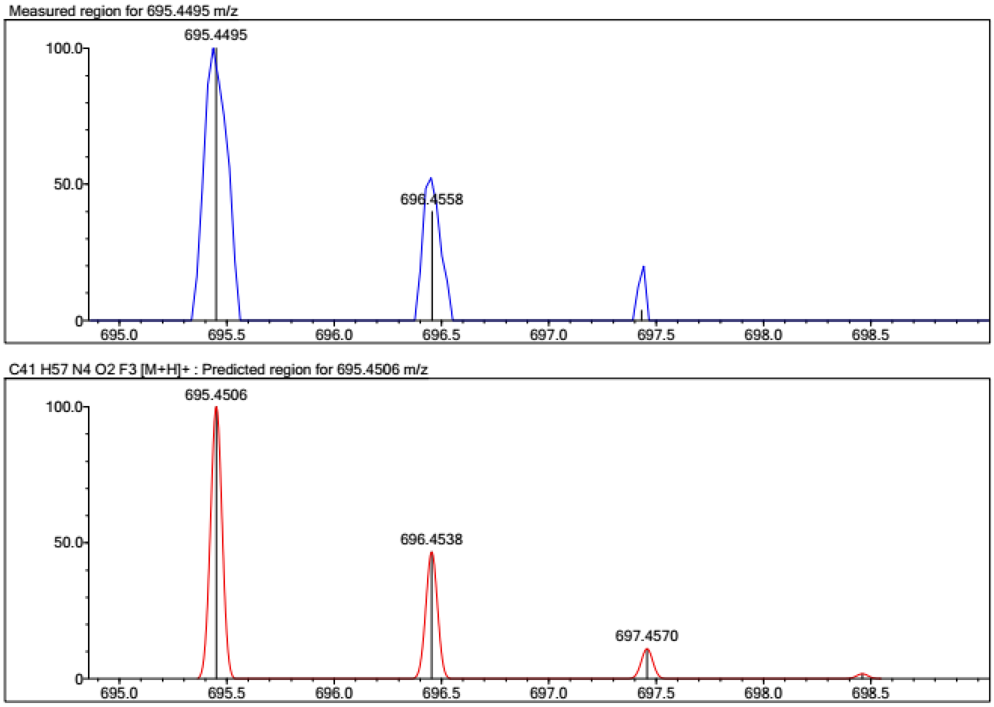

Rank	Score	Formula (M)	Ion	Meas. m/z	Pred. m/z	Df. (mDa)	Df. (ppm)	Iso	DBE
30	41.49	C41 H57 N4 O2 F3	[M+H]+	695.4495	695.4506	-1.1	-1.58	42.11	14.0

HRMS spectrum of **4l**

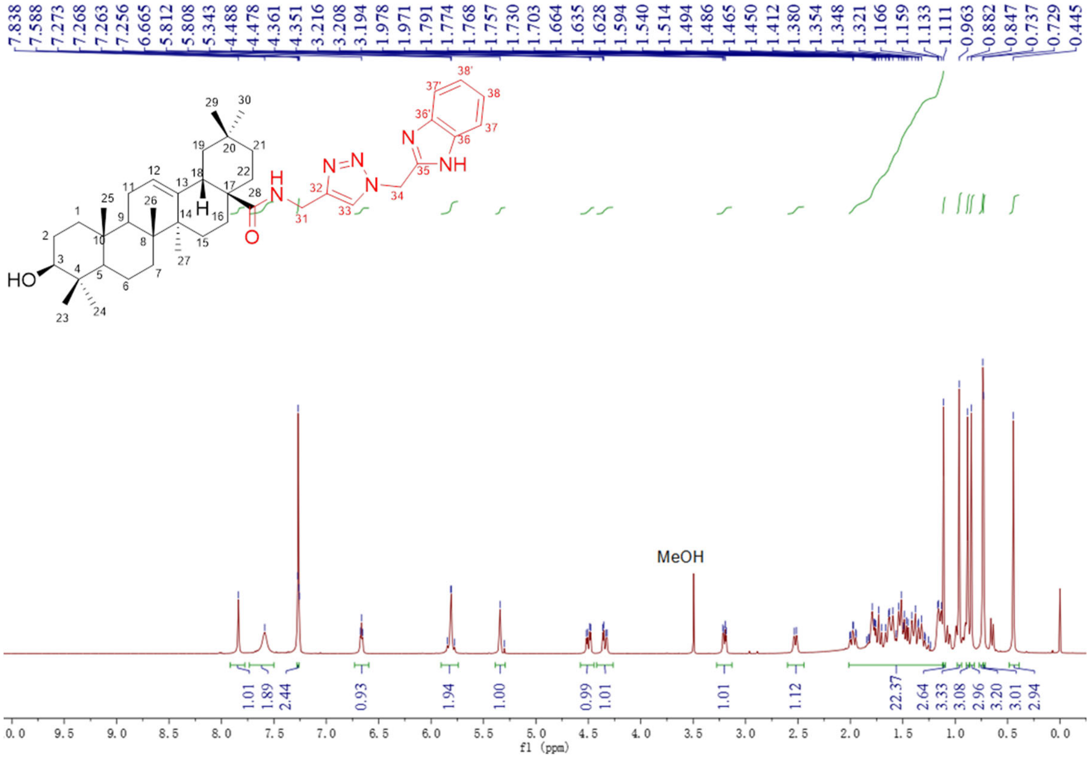

^{1}H NMR spectrum of **4m**

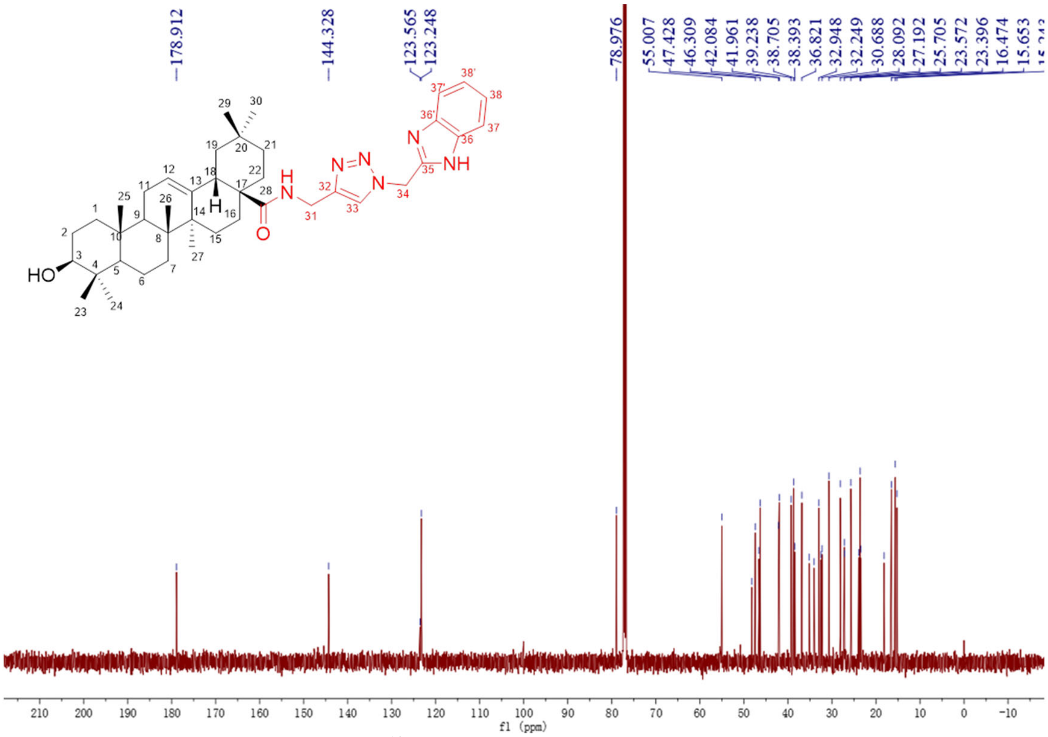

^{13}C NMR spectrum of **4m**

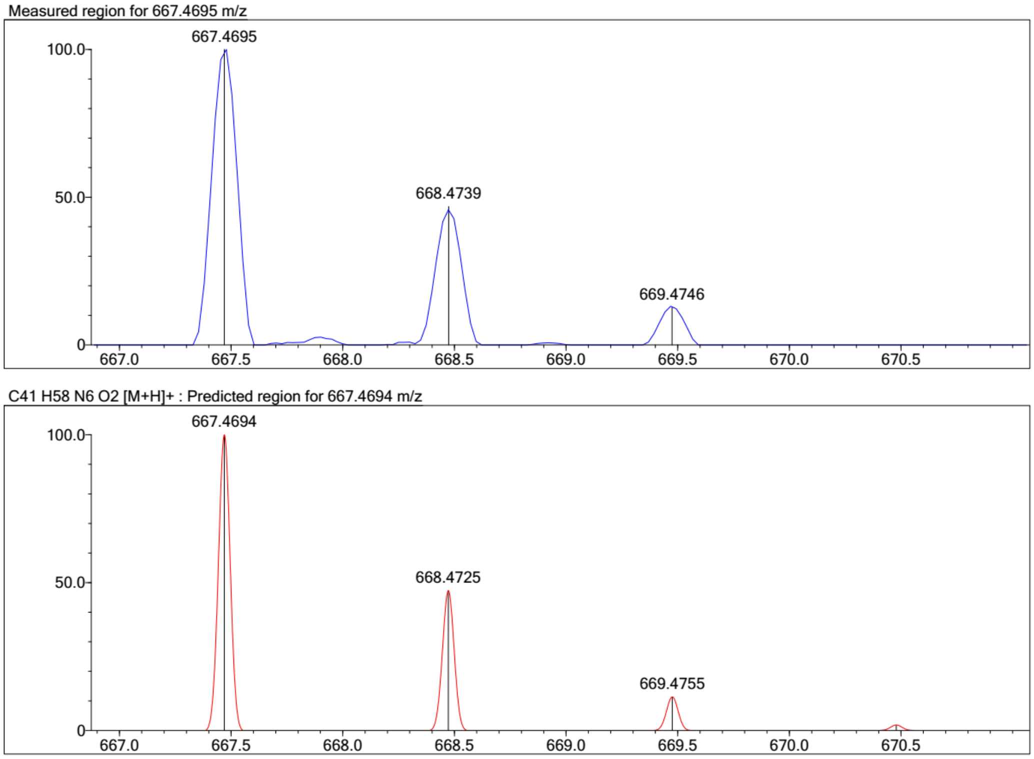

Rank	Score	Formula (M)	Ion	Meas. m/z	Pred. m/z	Df. (mDa)	Df. (ppm)	Iso	DBE
1	100.00	C41 H58 N6 O2	[M+H]+	667.4695	667.4694	0.1	0.15	100.00	16.0

HRMS spectrum of **4m**

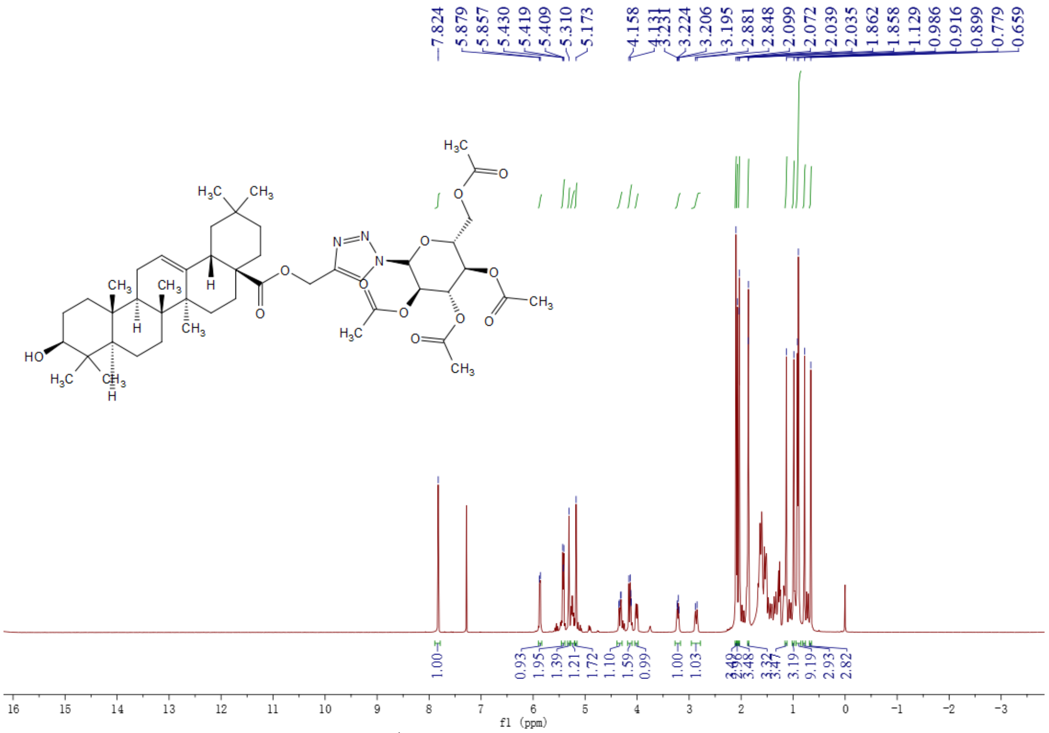

^{1}H NMR spectrum of **4n**

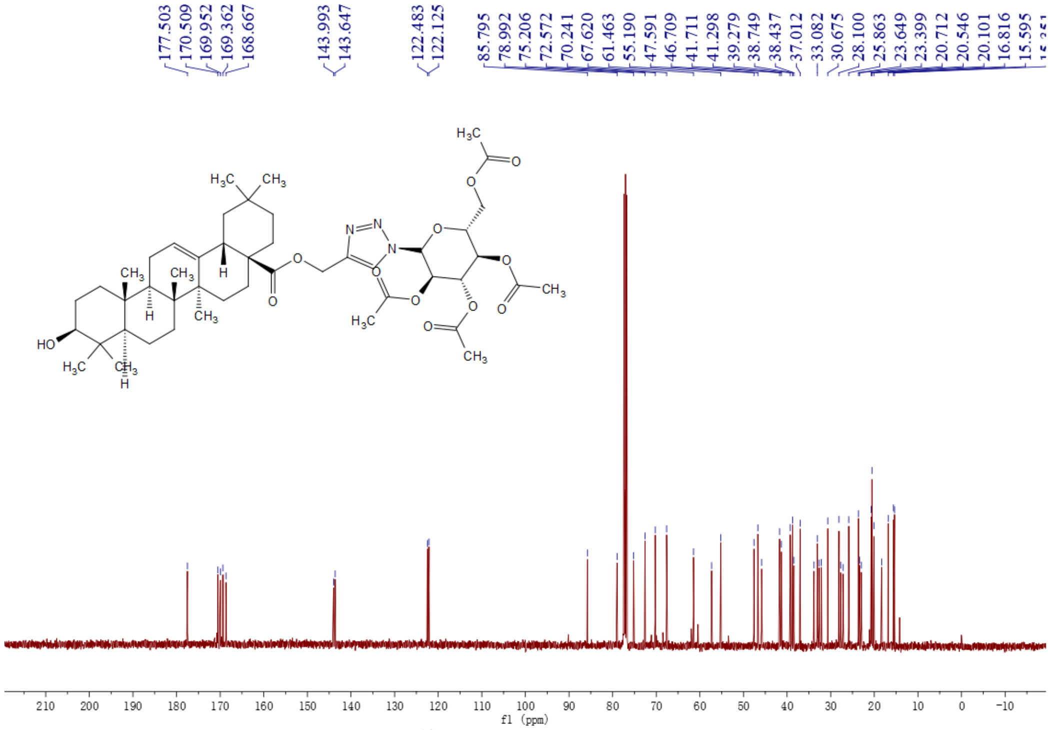

^{13}C NMR spectrum of **4n**

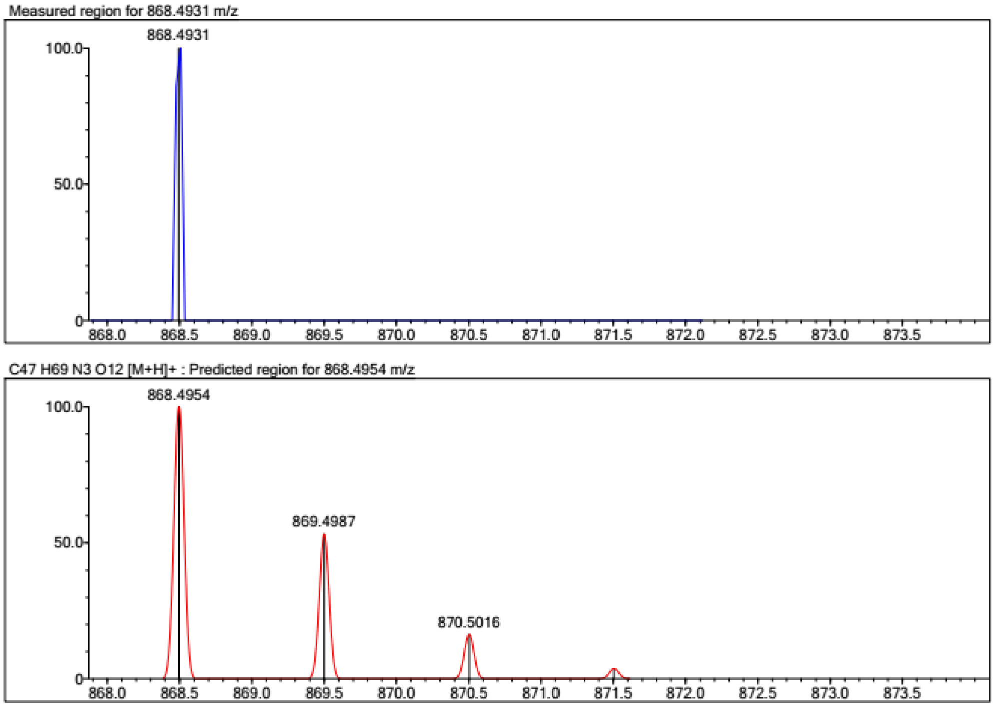

Rank	Score	Formula (M)	Ion	Meas. m/z	Pred. m/z	Df. (mDa)	Df. (ppm)	Iso	DBE
2	0.00	C47 H69 N3 O12	[M+H]+	868.4931	868.4954	-2.3	-2.65	0.00	15.0

HRMS spectrum of **4n**

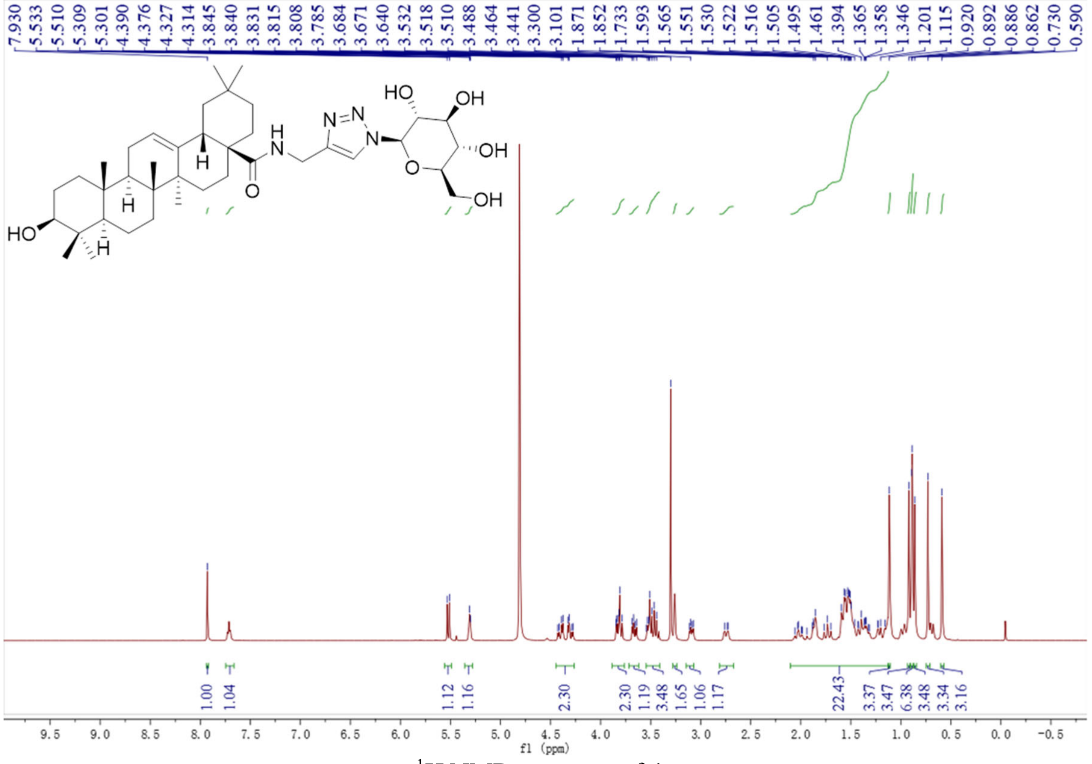

^{1}H NMR spectrum of **4o**

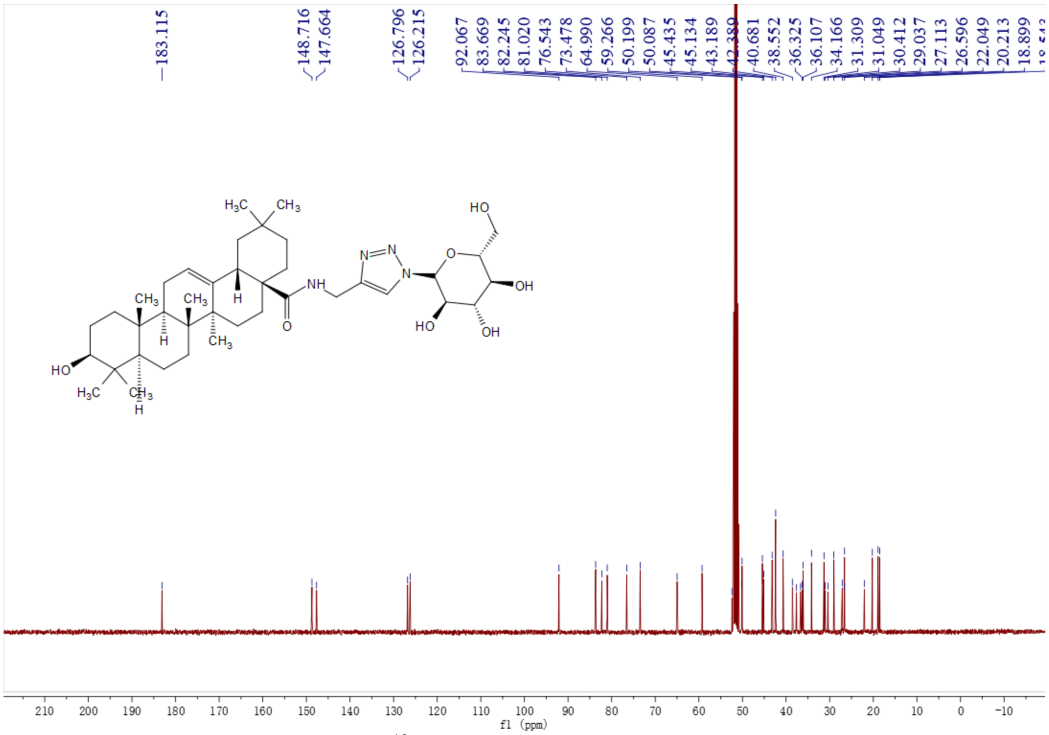

^{13}C NMR spectrum of **4o**

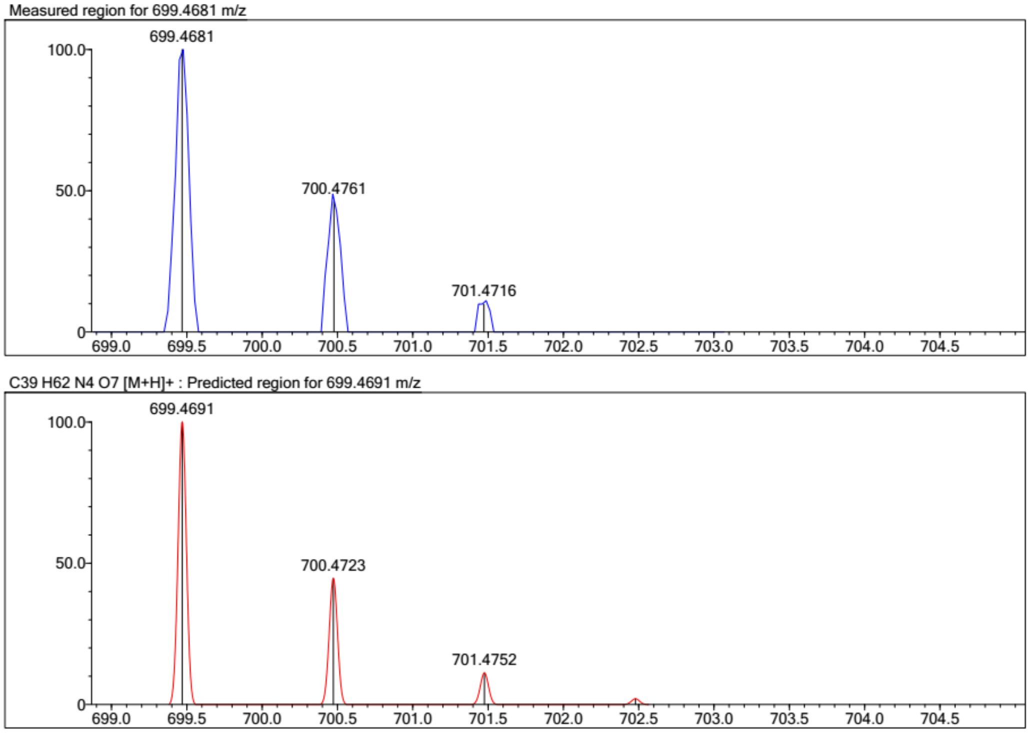

Rank	Score	Formula (M)	Ion	Meas. m/z	Pred. m/z	Df. (mDa)	Df. (ppm)	Iso	DBE
2	44.84	C39 H62 N4 O7	[M+H]+	699.4681	699.4691	-1.0	-1.43	45.33	11.0

HRMS spectrum of **4o**

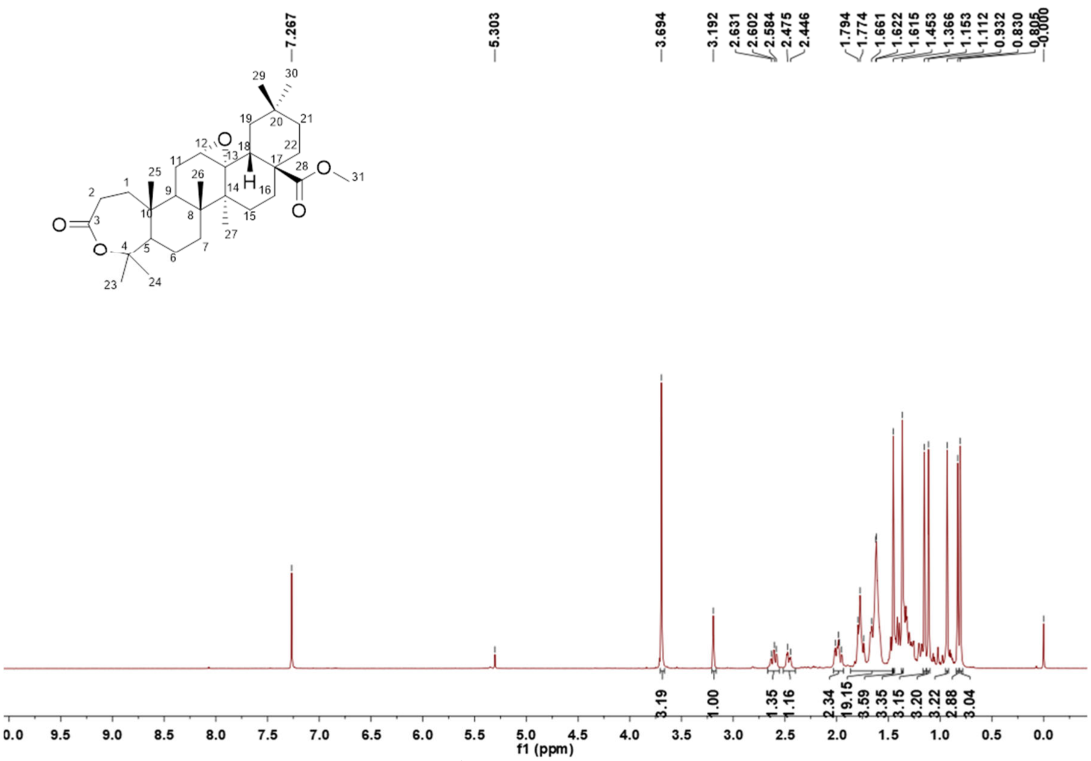

^{1}H spectrum of **7**

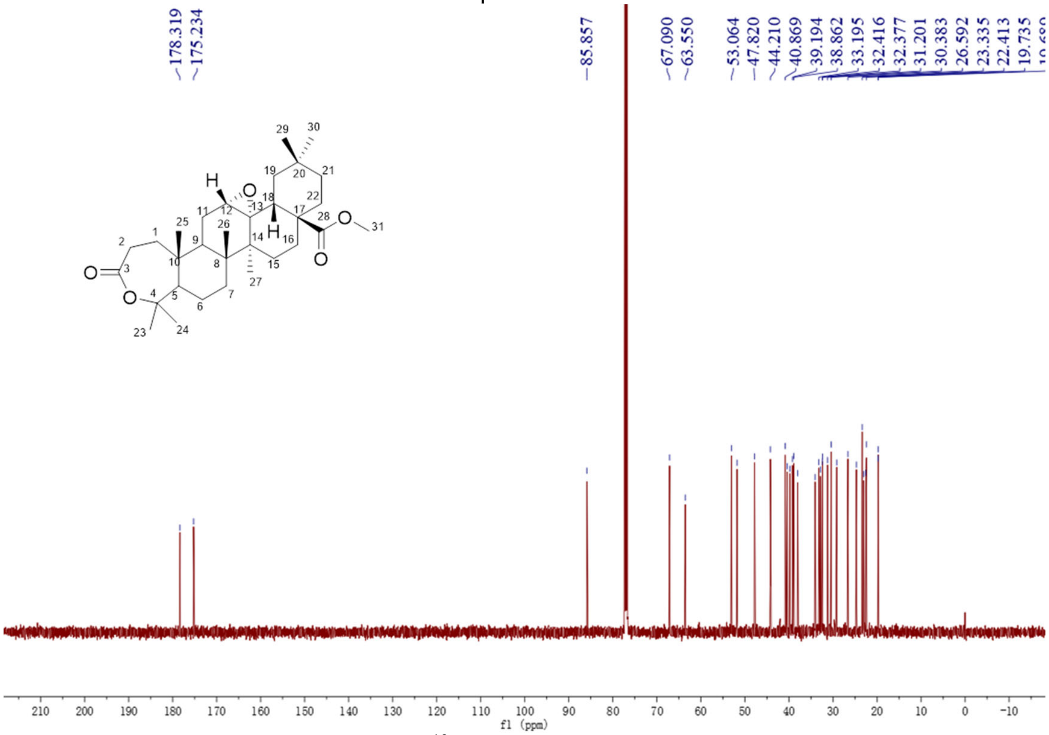

^{13}C spectrum of **7**

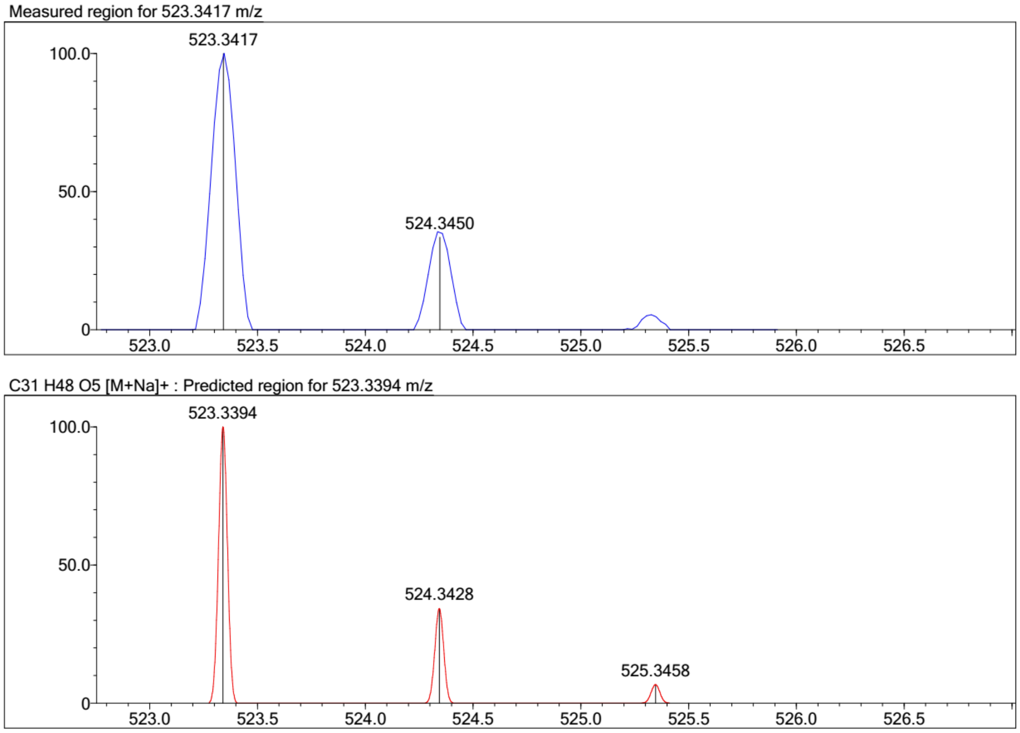

Rank	Score	Formula (M)	Ion	Meas. m/z	Pred. m/z	Df. (mDa)	Df. (ppm)	Iso	DBE
1	62.79	C31 H48 O5	[M+Na]+	523.3417	523.3394	2.3	4.39	68.61	8.0

HRMS spectrum of **7**

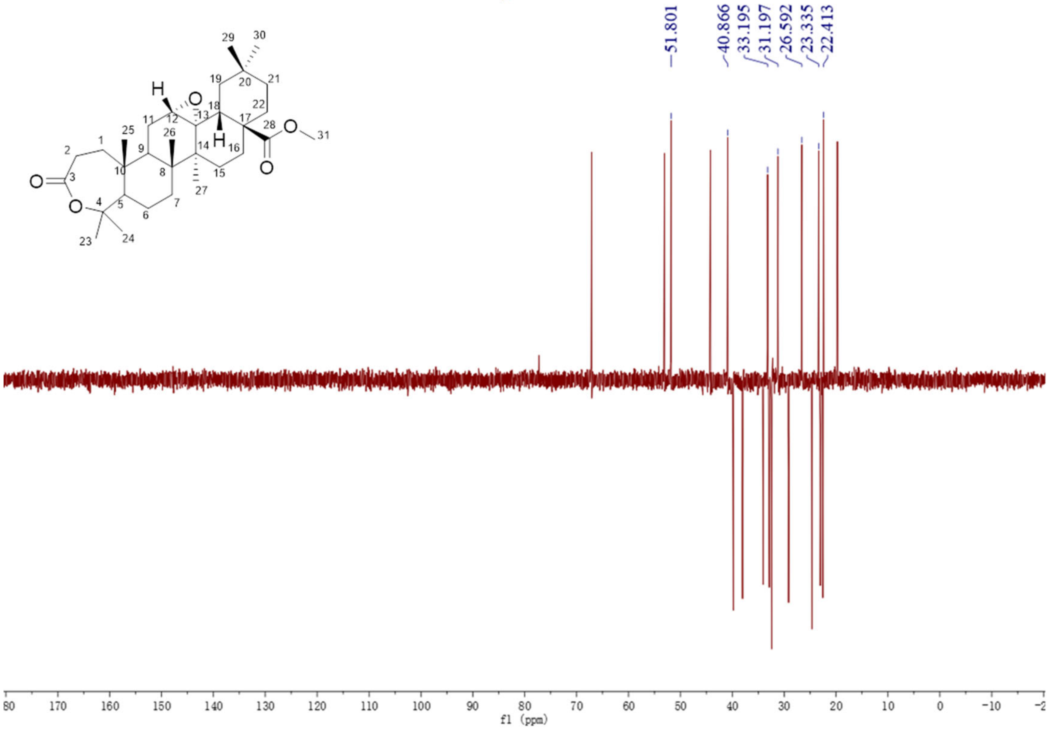

DEPT135 spectrum of **7**

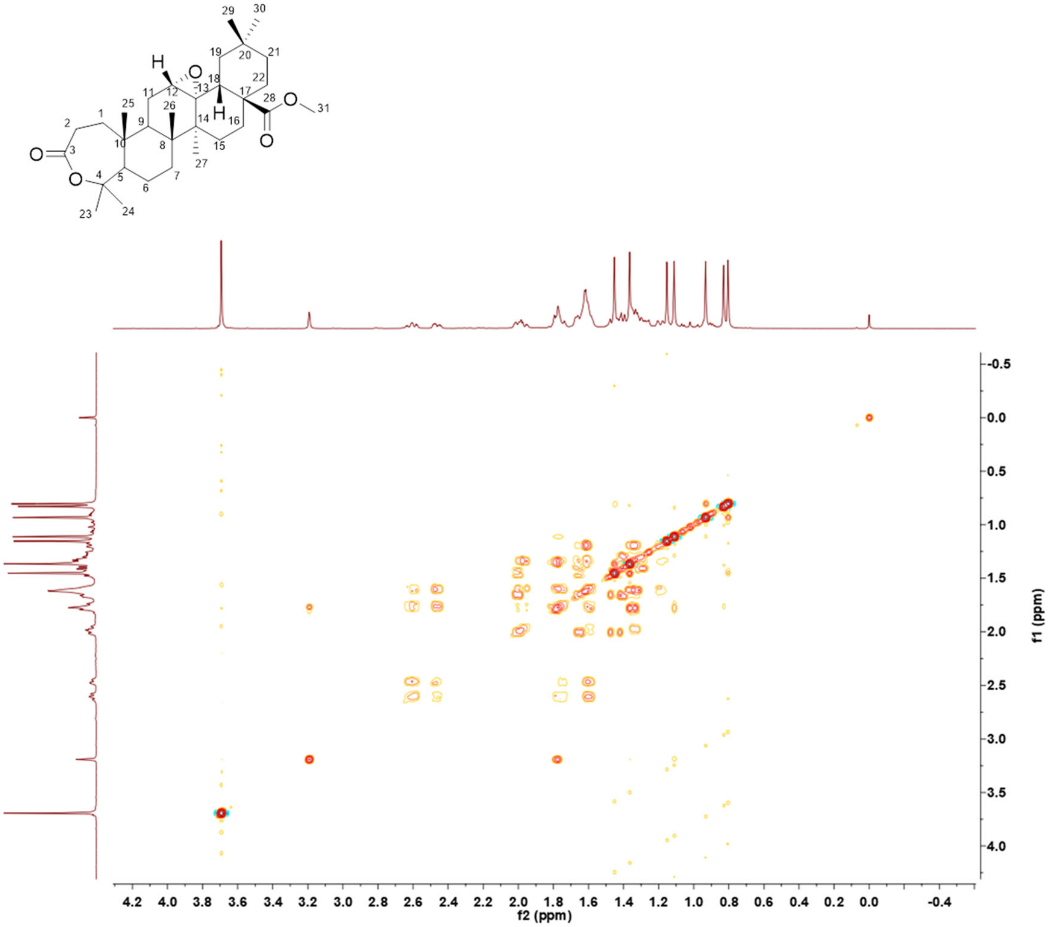

H,H-Cosy spectrum of **7**

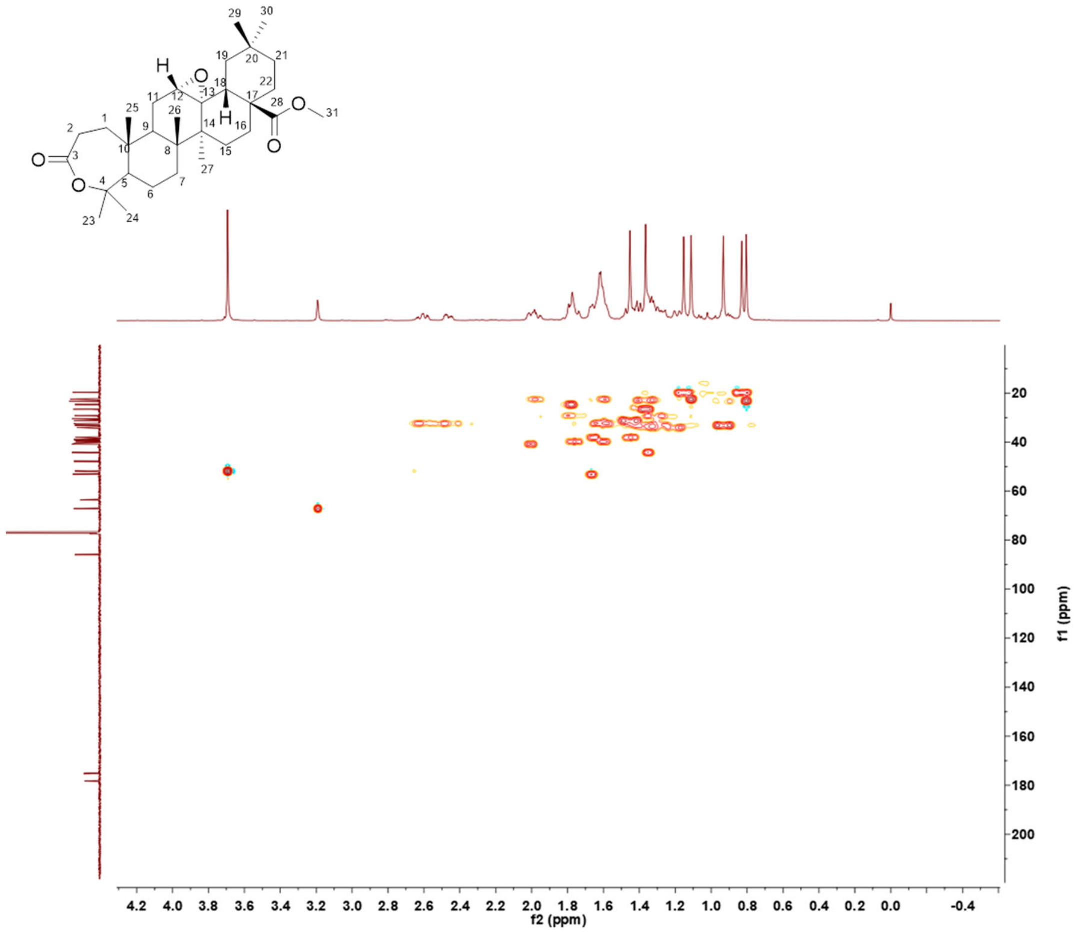

HSQC spectrum of **7**

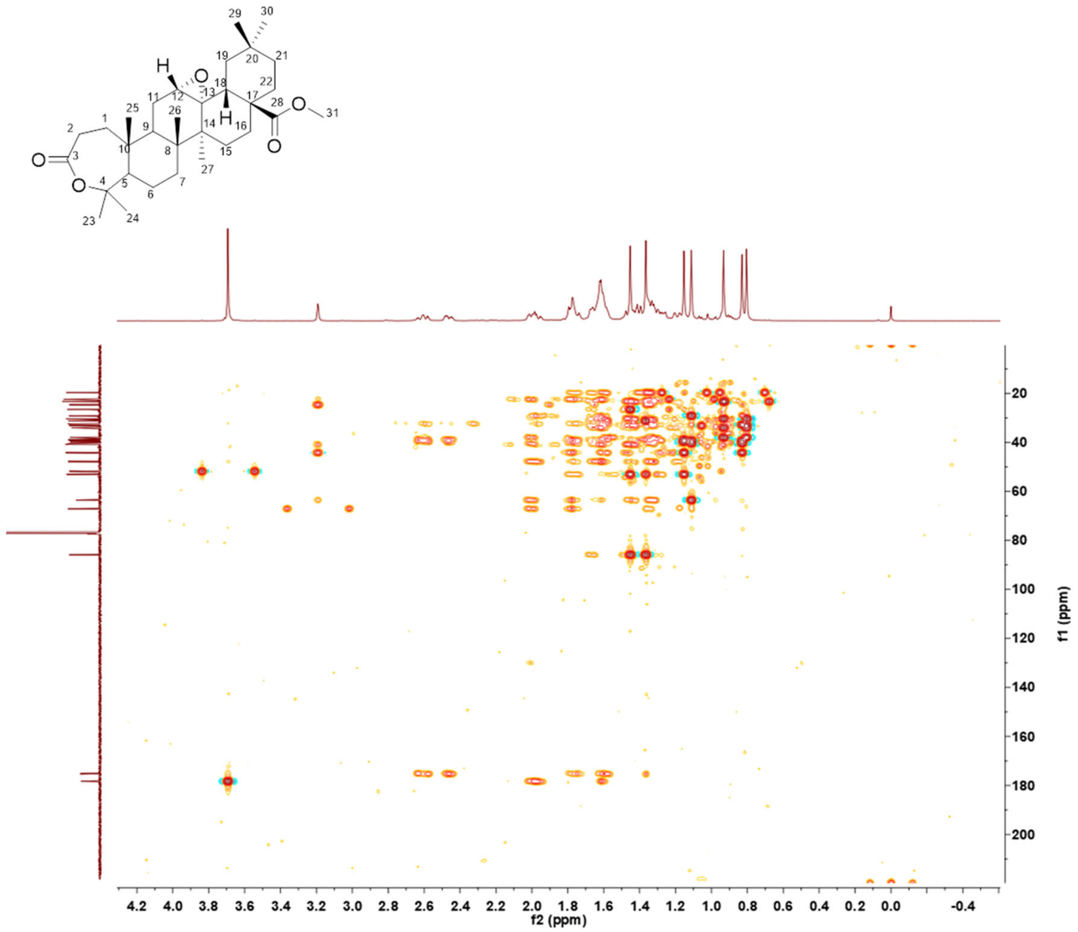

HMBC spectrum of **7**

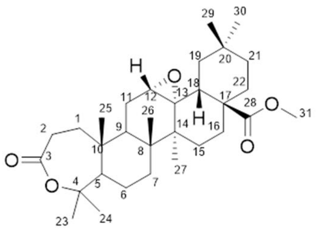

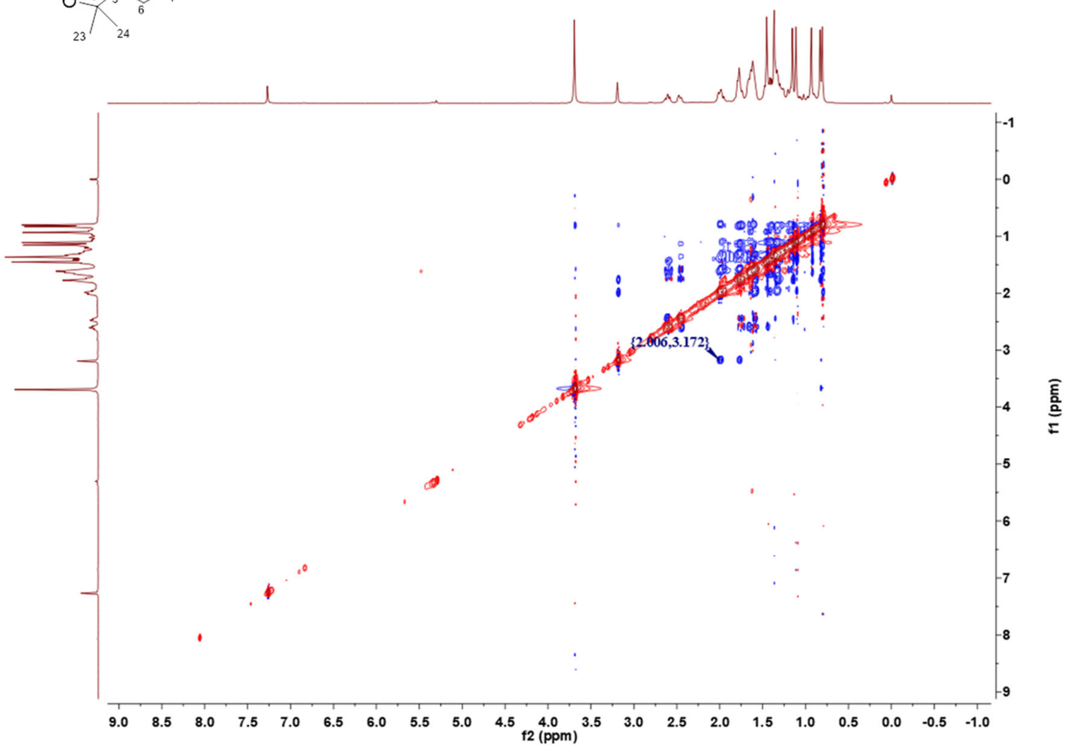

NOE spectrum of **7**

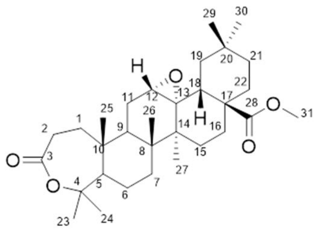

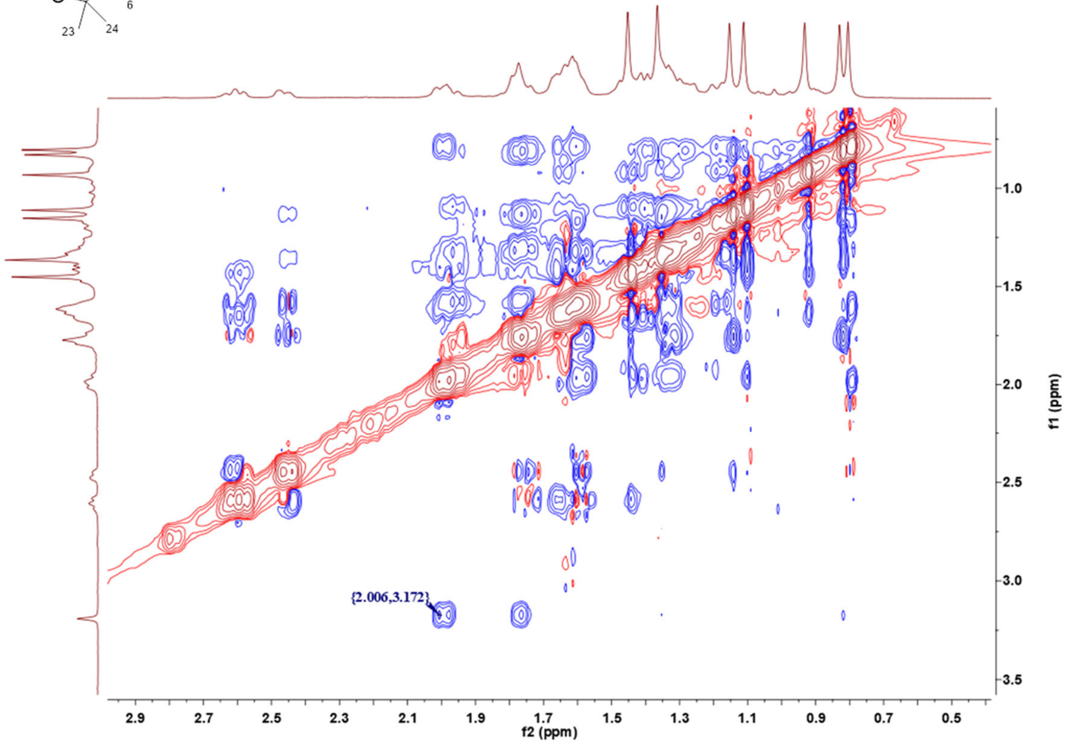

HMBC local zoom spectrum of **7**

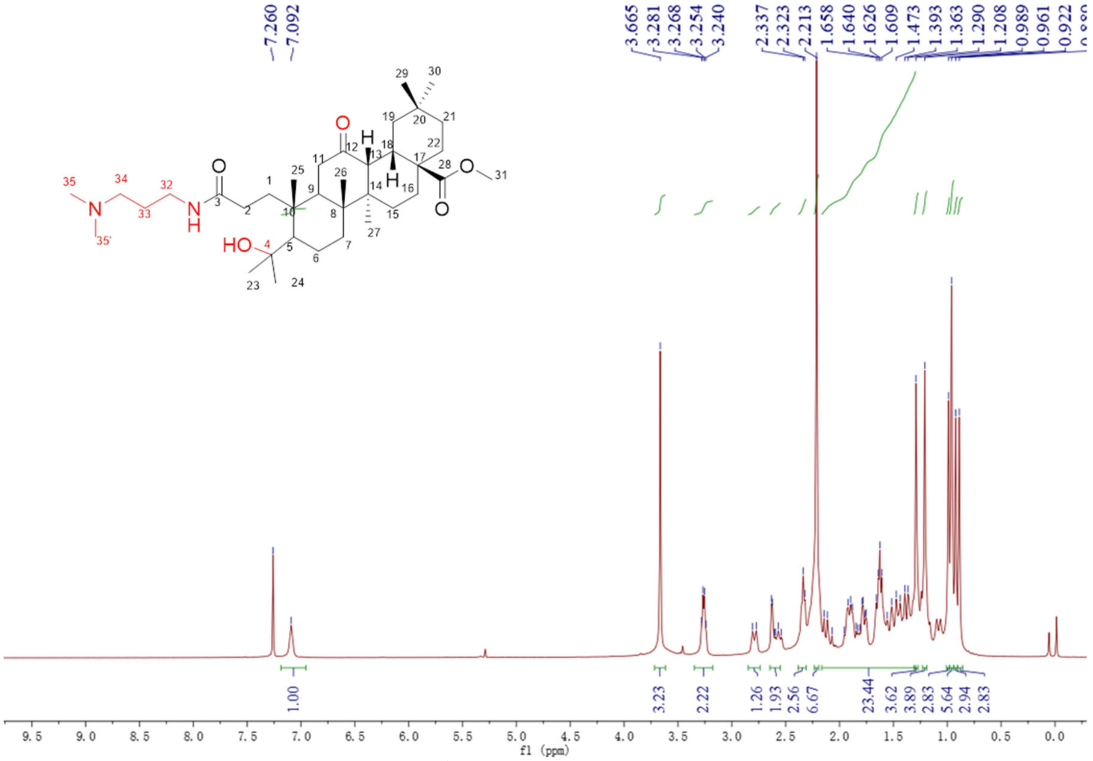

^{1}H NMR spectrum of **8a**

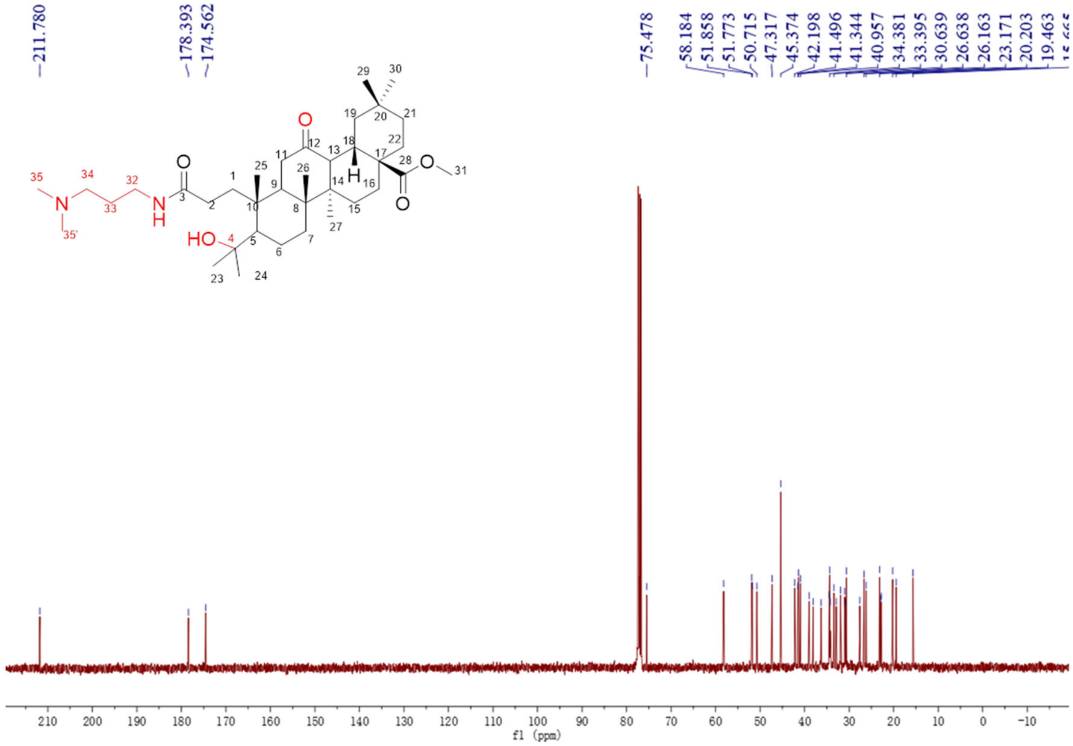

^{13}C NMR spectrum of **8a**

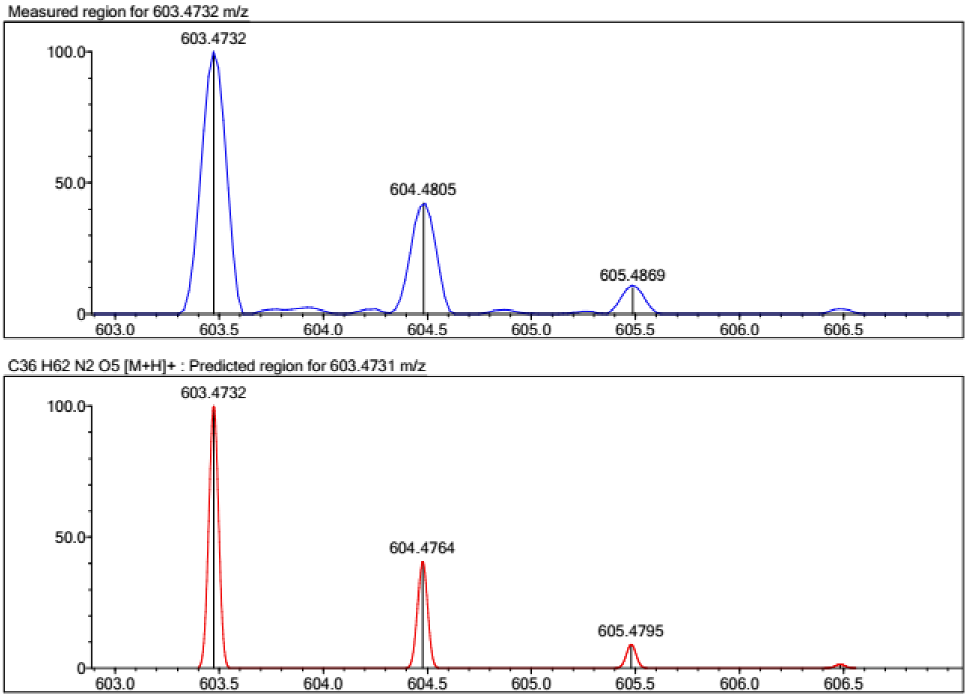

Rank	Score	Formula (M)	Ion	Meas. m/z	Pred. m/z	Df. (mDa)	Df. (ppm)	Iso	DBE
2	92.69	C36 H62 N2 O5	[M+H]+	603.4732	603.4731	0.1	0.17	92.69	7.0

HRMS spectrum of **8a**

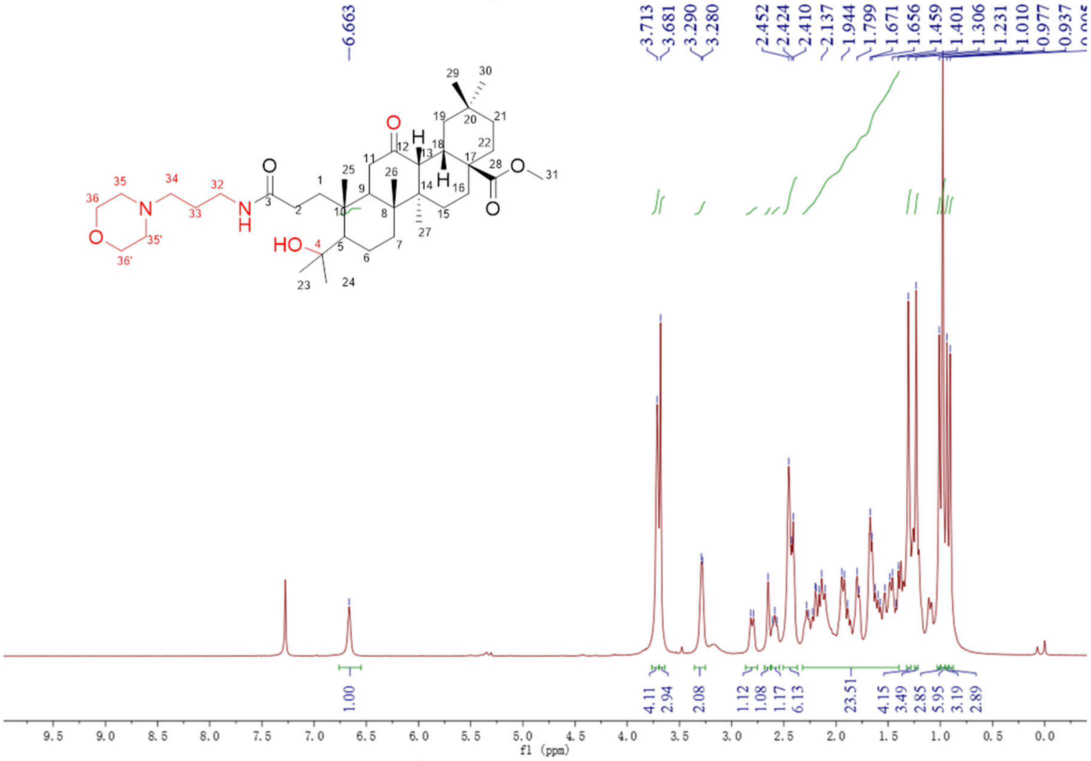

^{1}H NMR spectrum of **8b**

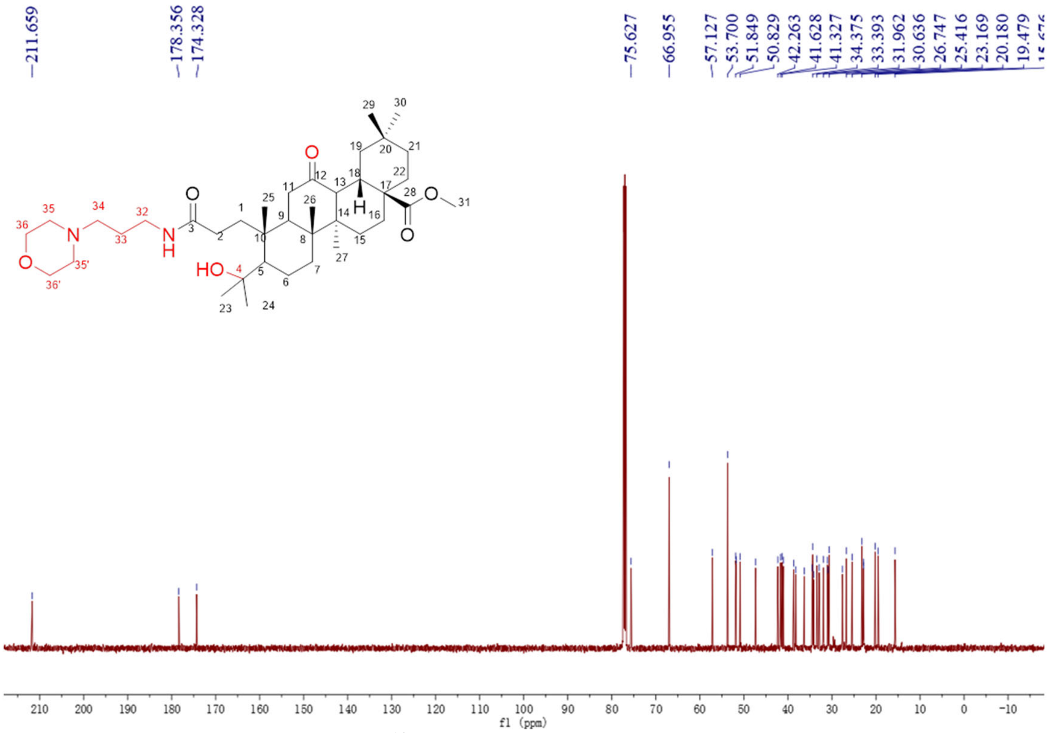

^{13}C NMR spectrum of **8b**

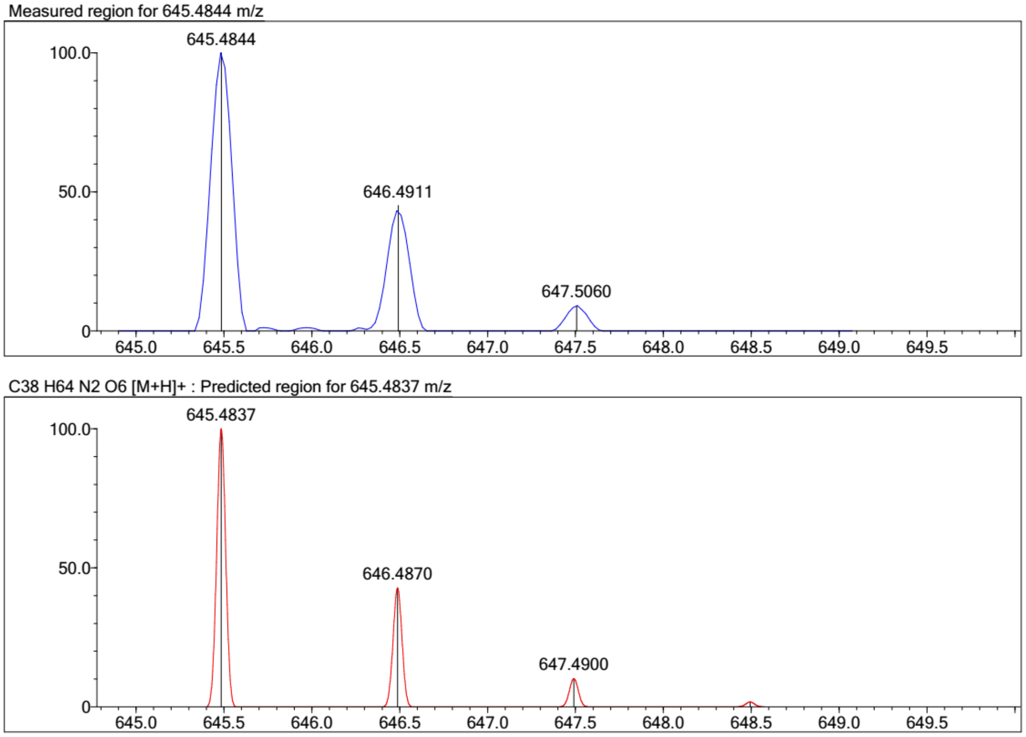

Rank	Score	Formula (M)	Ion	Meas. m/z	Pred. m/z	Df. (mDa)	Df. (ppm)	Iso	DBE
2	88.24	C38 H64 N2 O6	[M+H]+	645.4844	645.4837	0.7	1.08	88.42	8.0

HRMS spectrum of **8b**

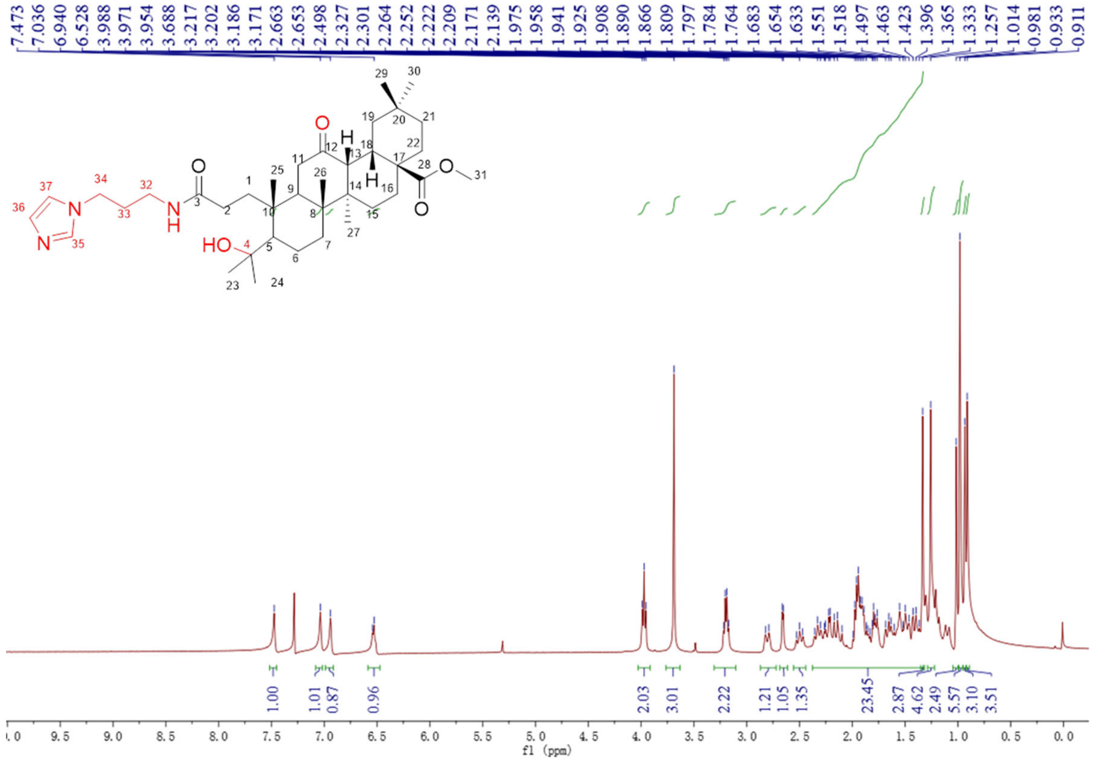

^{1}H NMR spectrum of **8c**

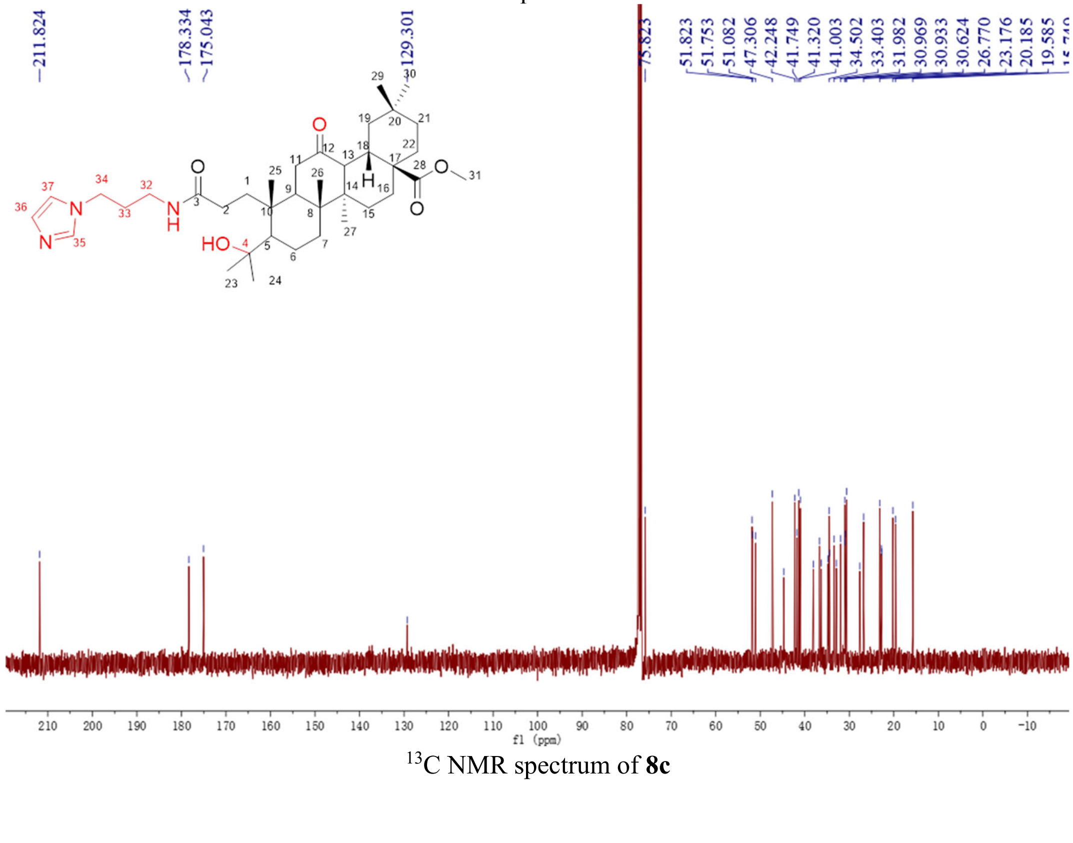

^{13}C NMR spectrum of **8c**

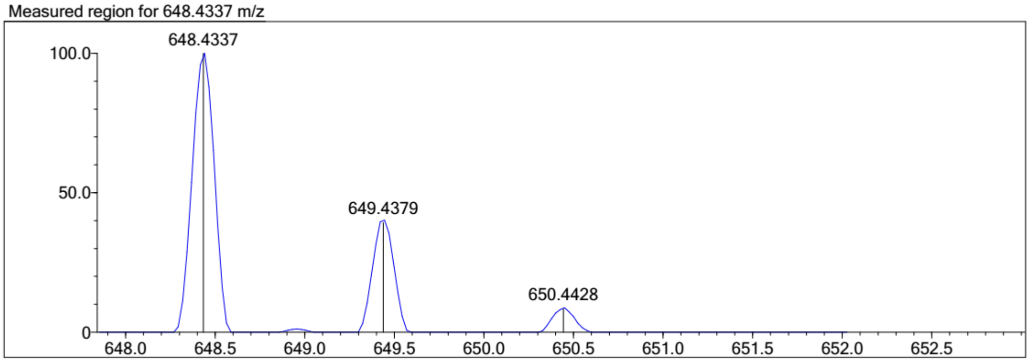

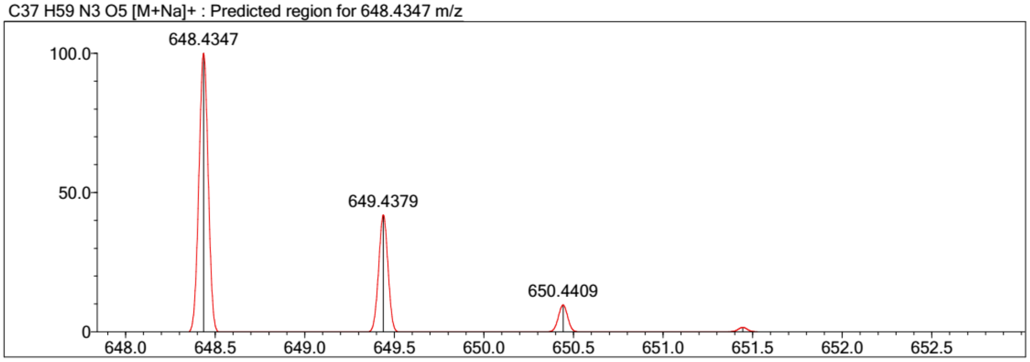

Rank	Score	Formula (M)	Ion	Meas. m/z	Pred. m/z	Df. (mDa)	Df. (ppm)	Iso	DBE
4	95.25	C37 H59 N3 O5	[M+Na]+	648.4337	648.4347	-1.0	-1.54	96.56	10.0

HRMS spectrum of **8c**

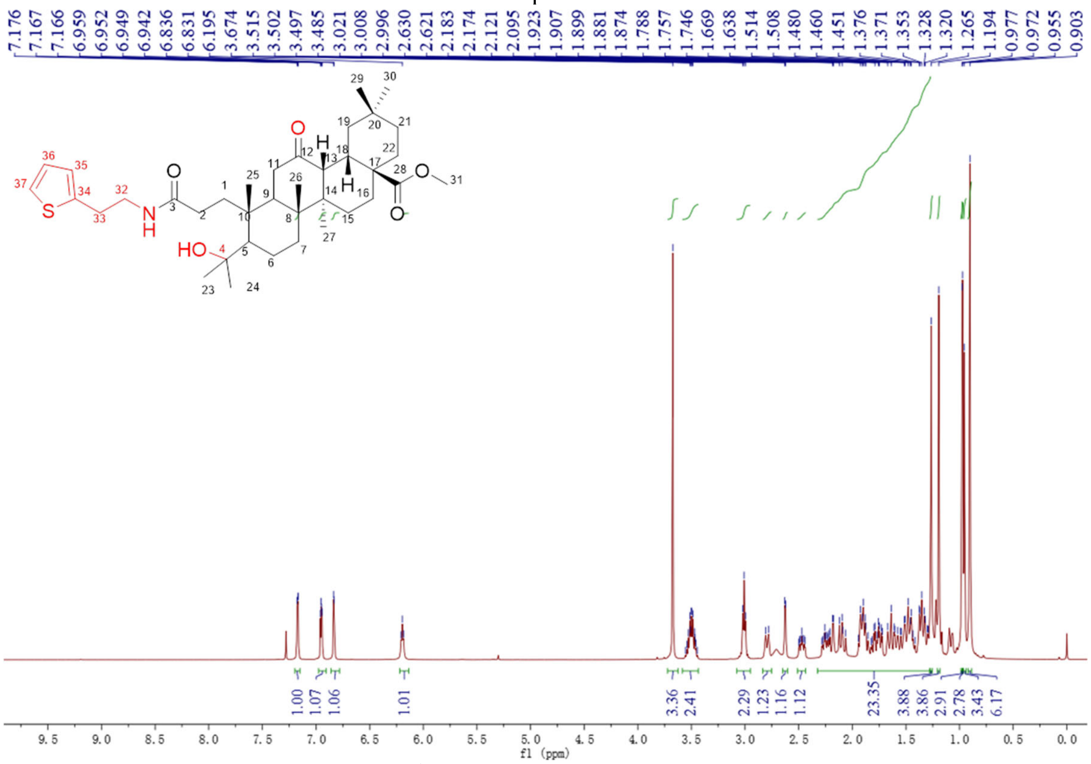

^{1}H NMR spectrum of **8d**

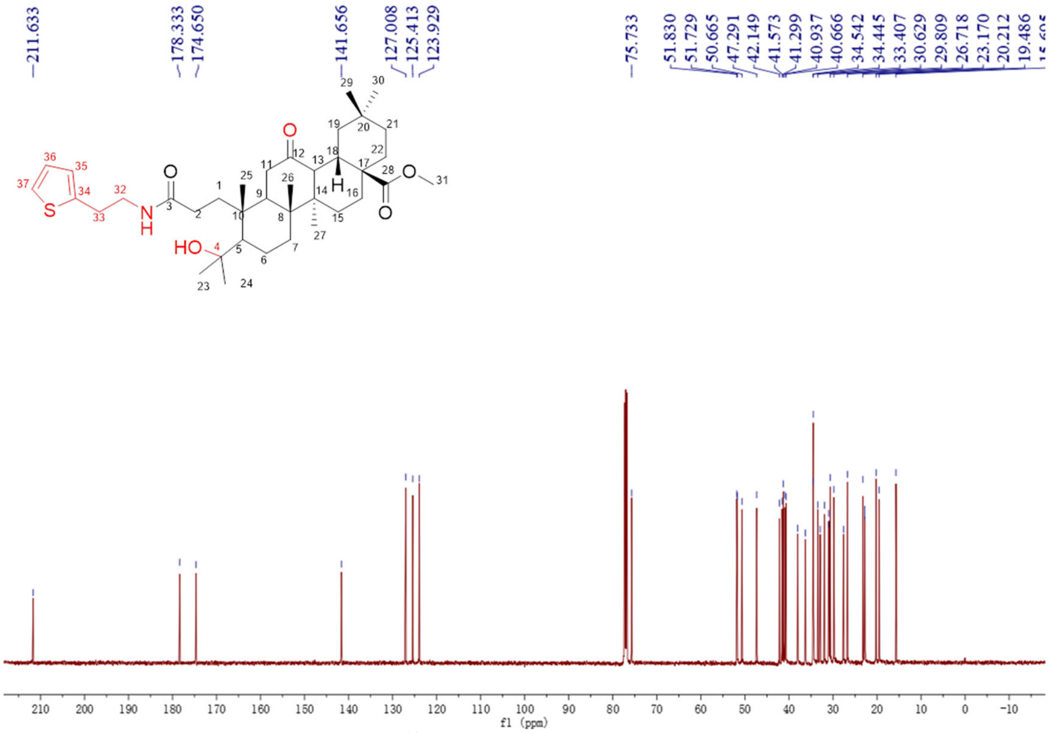

^{13}C NMR spectrum of **8d**

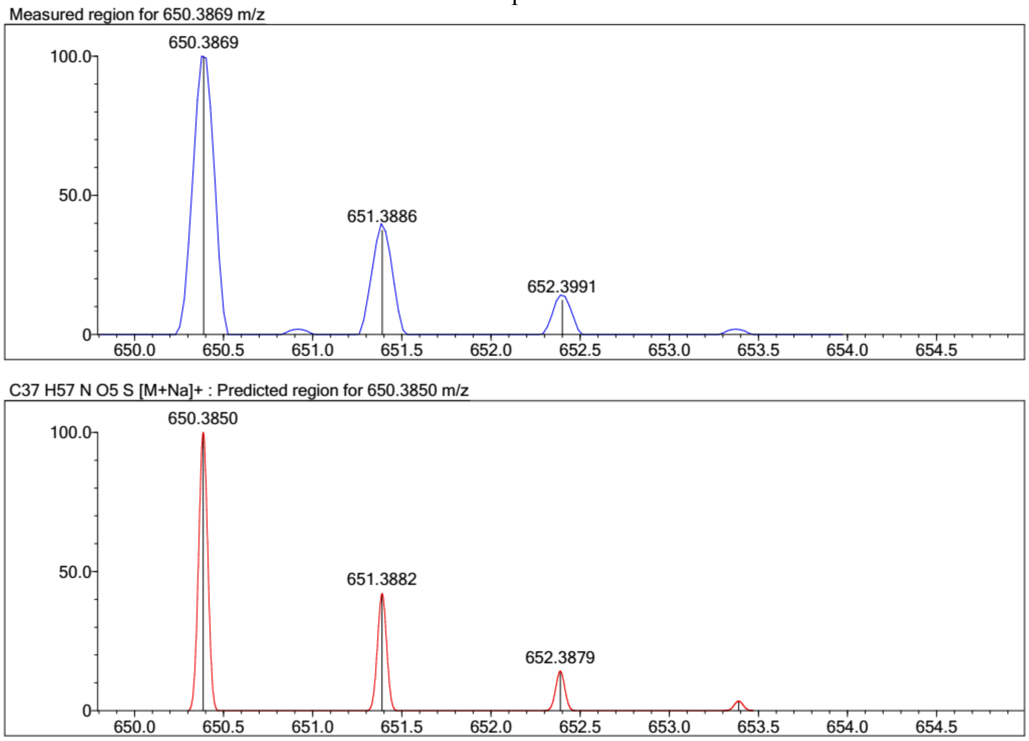

Rank	Score	Formula (M)	Ion	Meas. m/z	Pred. m/z	Df. (mDa)	Df. (ppm)	Iso	DBE
1	75.45	C37 H57 N O5 S	[M+Na]+	650.3869	650.3850	1.9	2.92	79.26	10.0

HRMS spectrum of **8d**

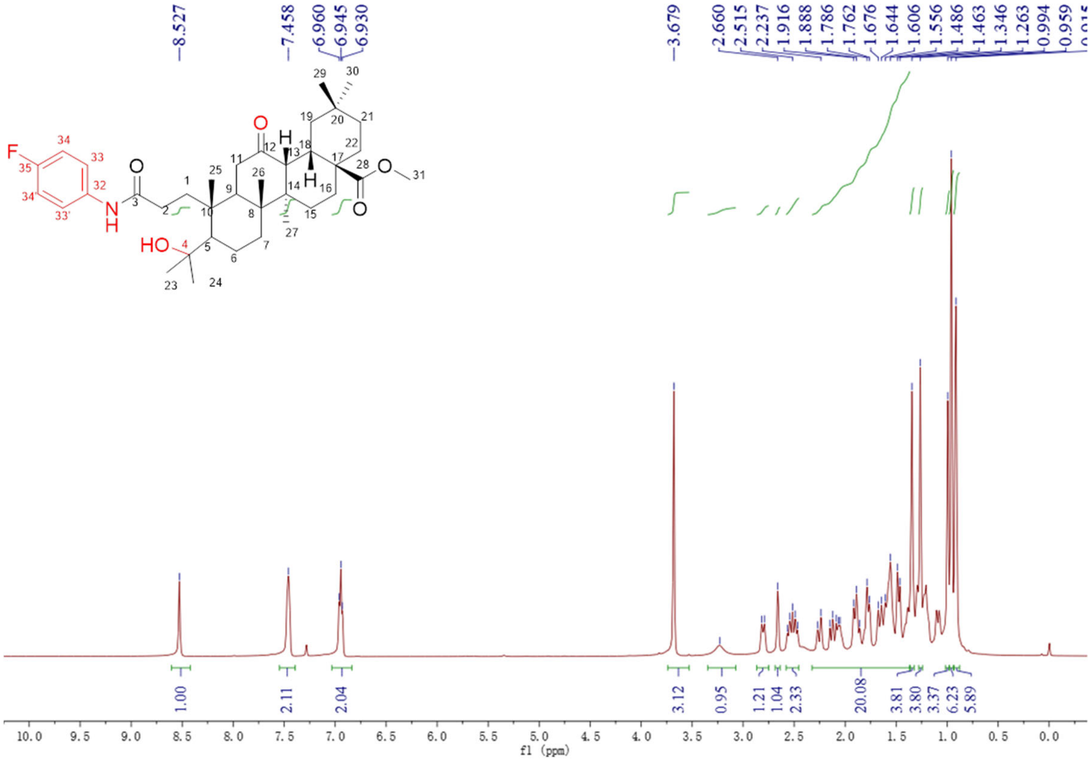

^{1}H spectrum of **8e**

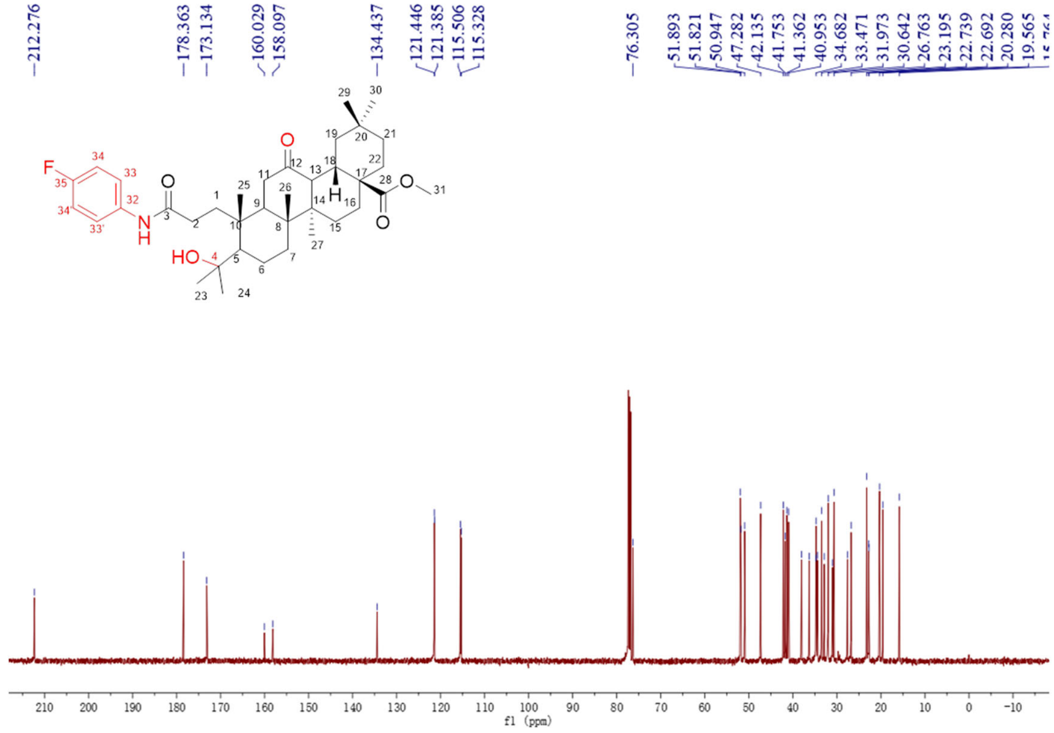

^{13}C spectrum of **8e**

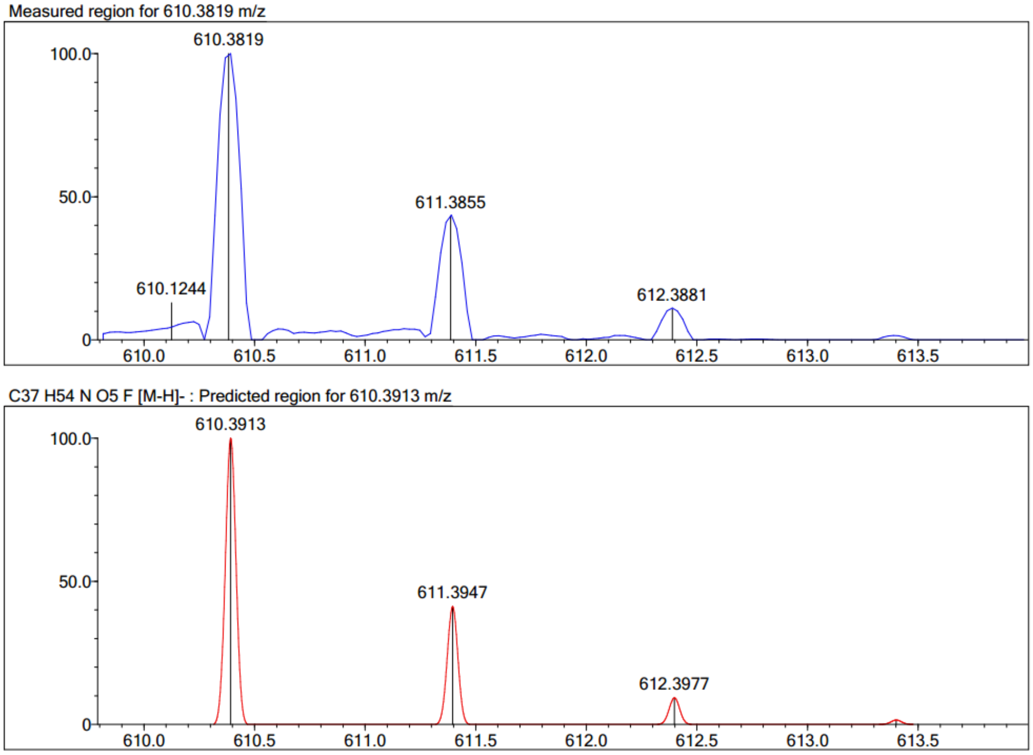

Rank	Score	Formula (M)	Ion	Meas. m/z	Pred. m/z	Df. (mDa)	Df. (ppm)	Iso	DBE
10	21.10	C37 H54 N O5 F	[M-H]-	610.3819	610.3913	-9.4	-15.40	82.43	11.0

HRMS spectrum of **8e**

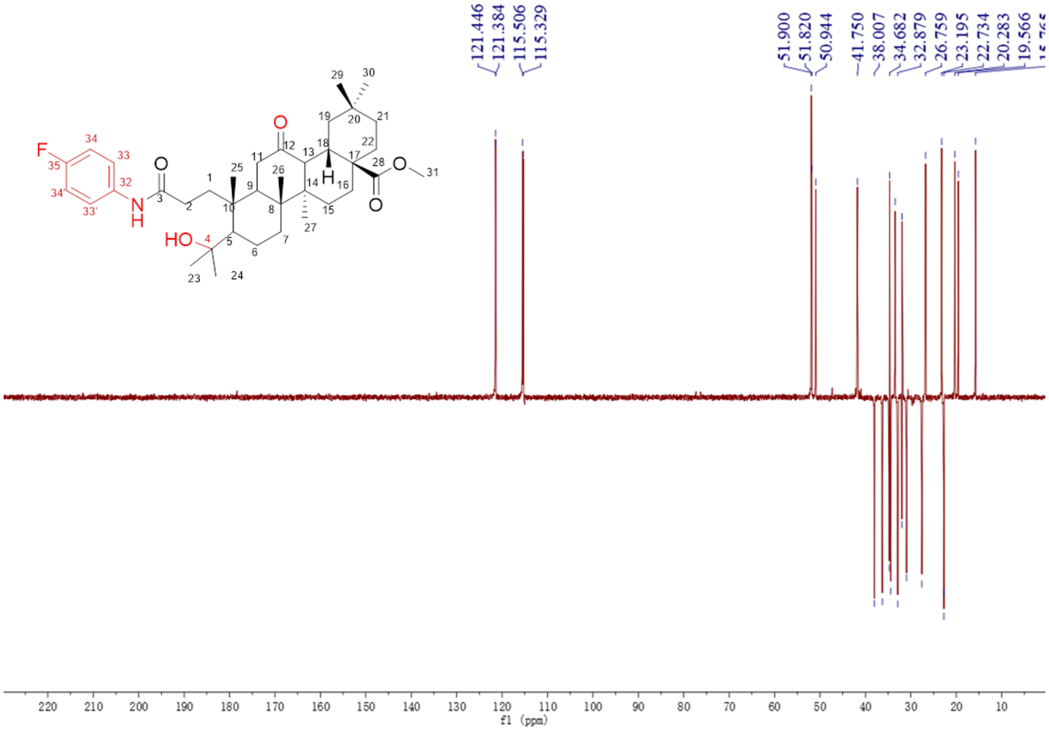

DEPT135 spectrum of **8e**

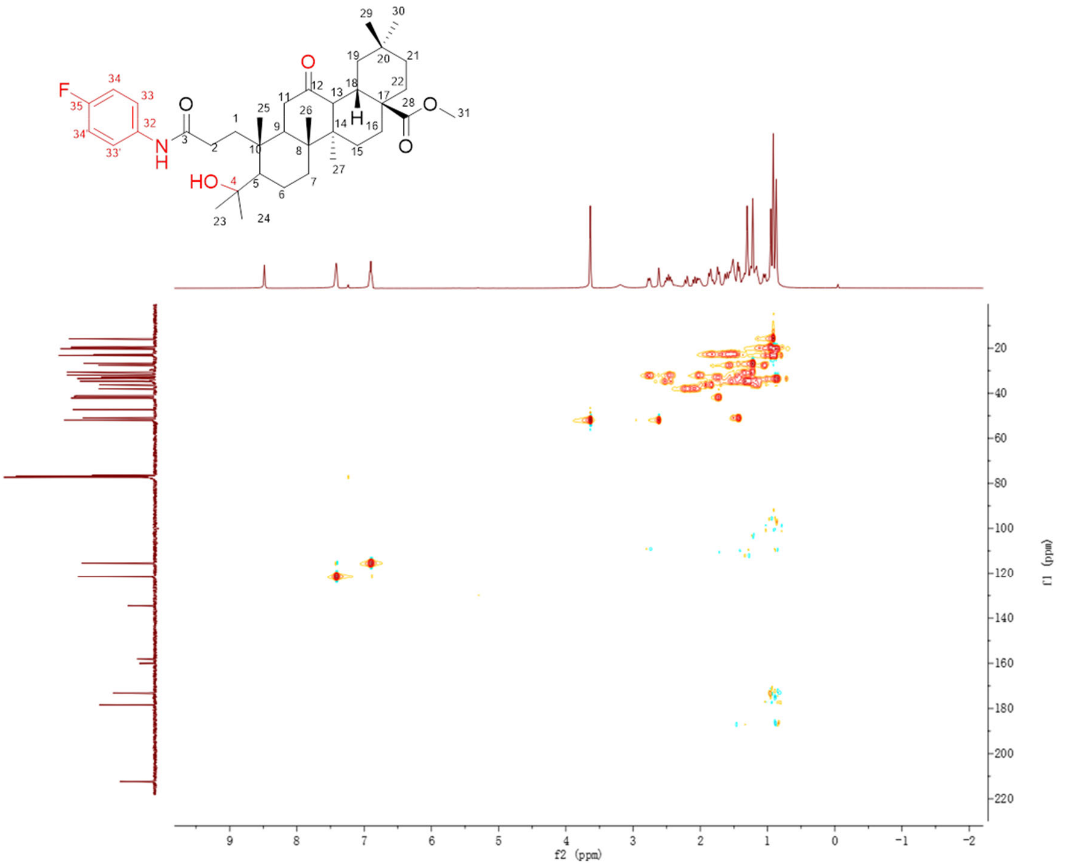

HSQC spectrum of **8e**

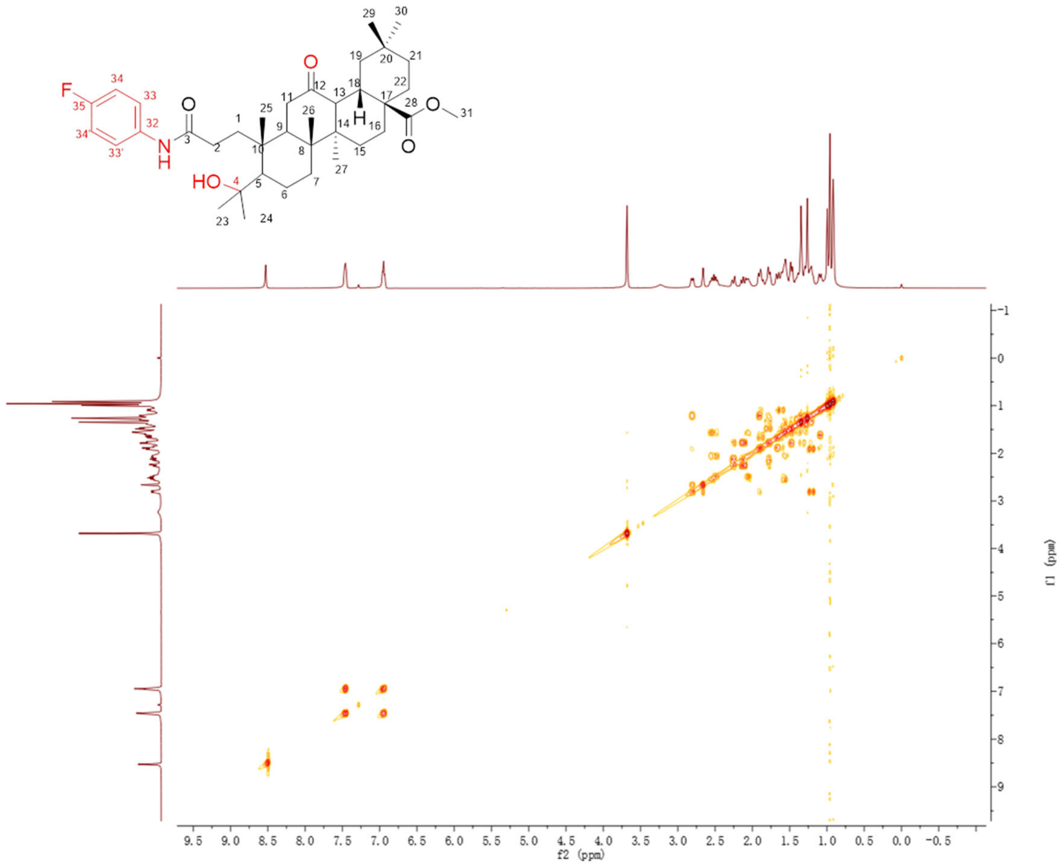

H,H-Cosy spectrum of **8e**

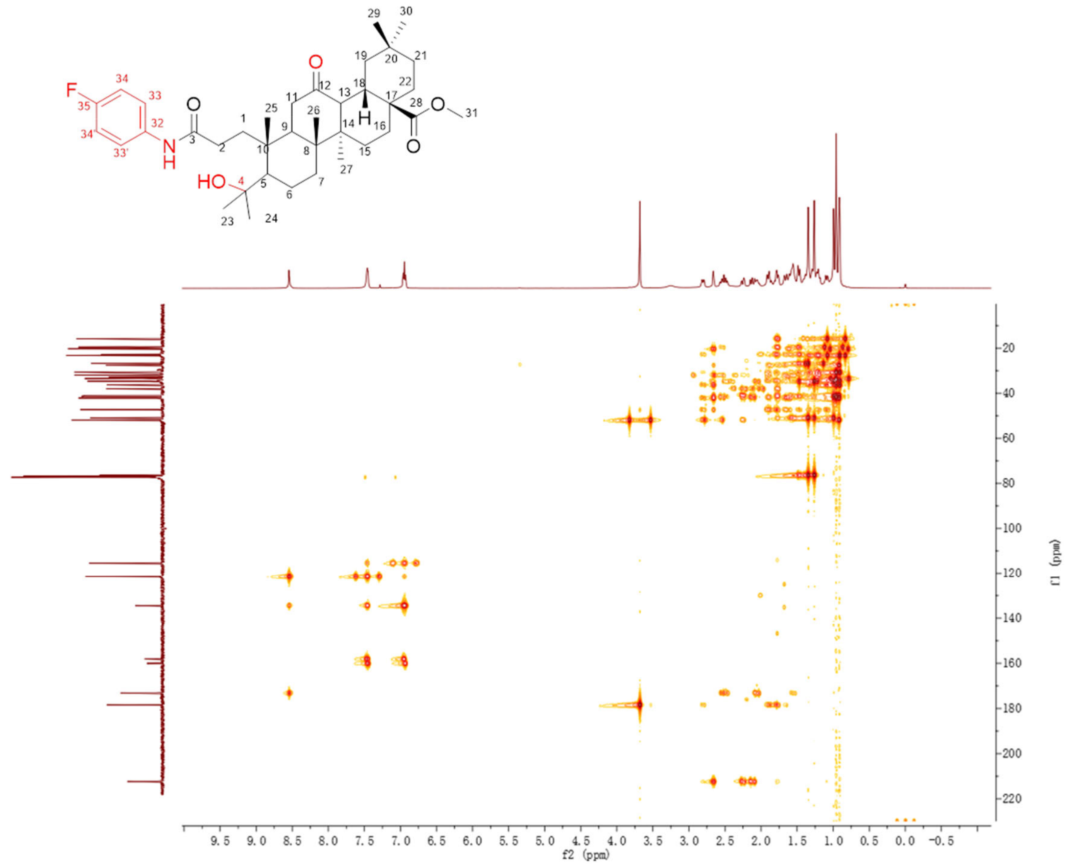

HMBC spectrum of **8e**

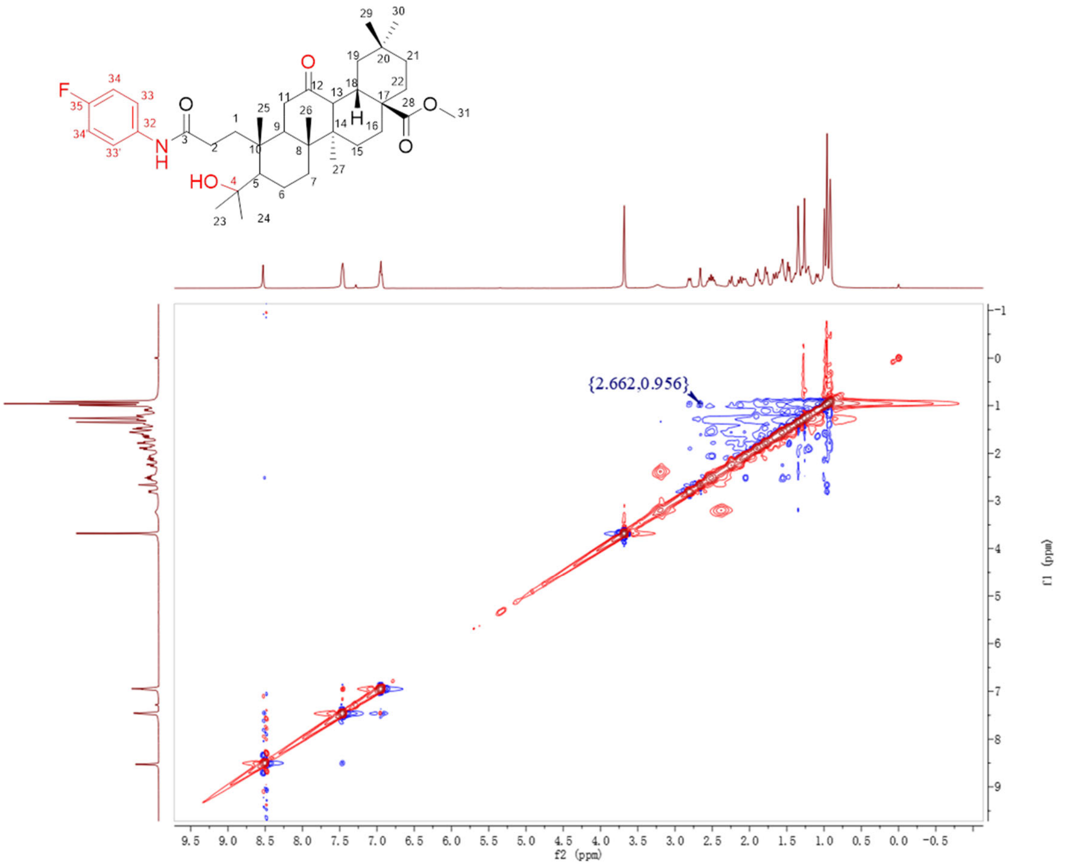

NOE spectrum of **8e**

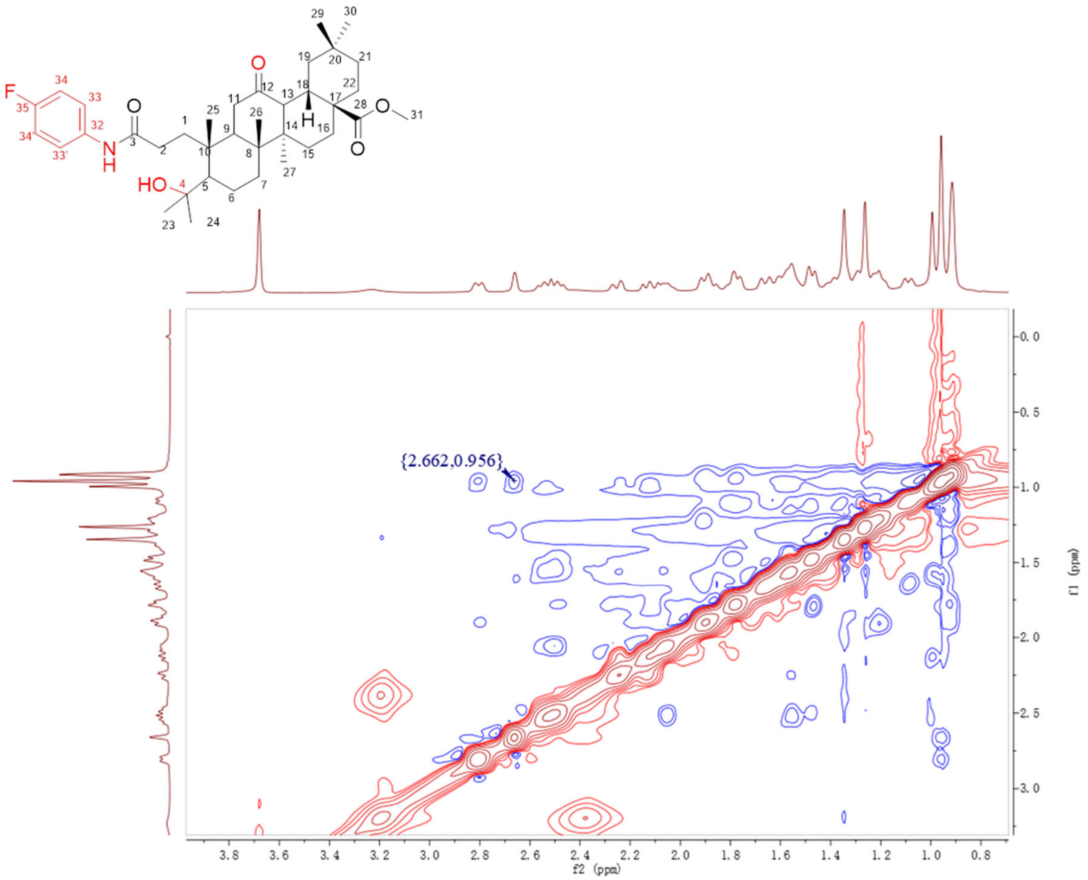

HMBC local zoom spectrum of **8e**

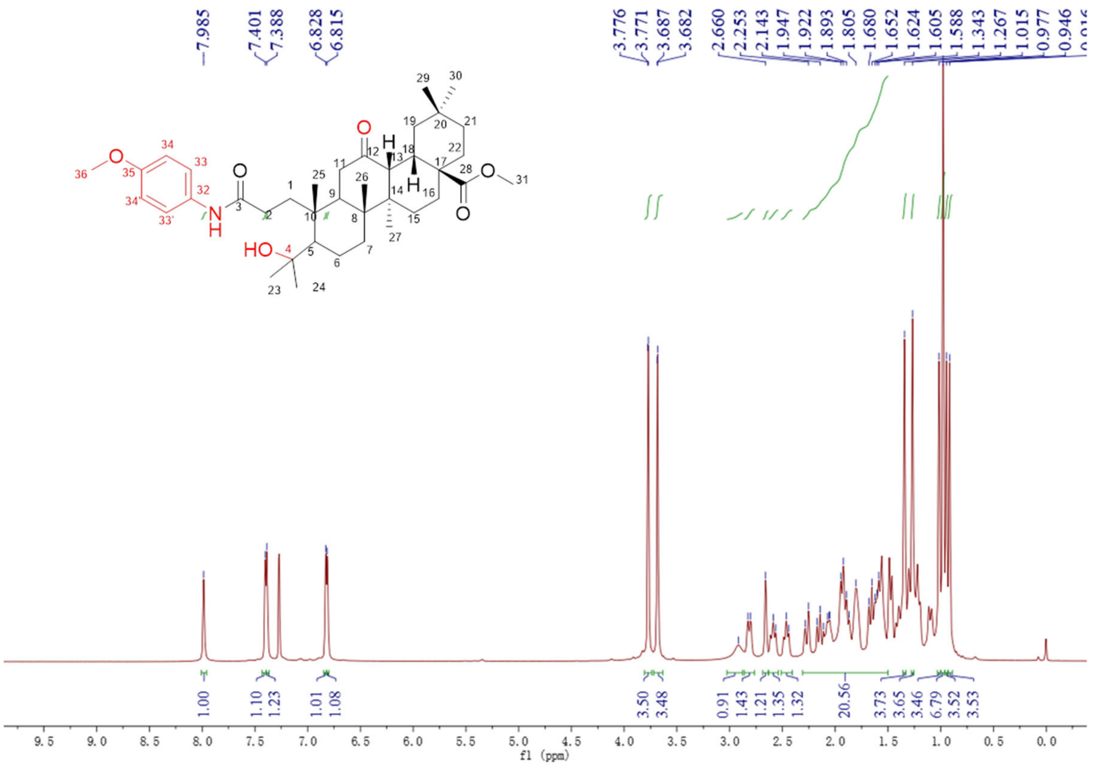

^{1}H NMR spectrum of **8f**

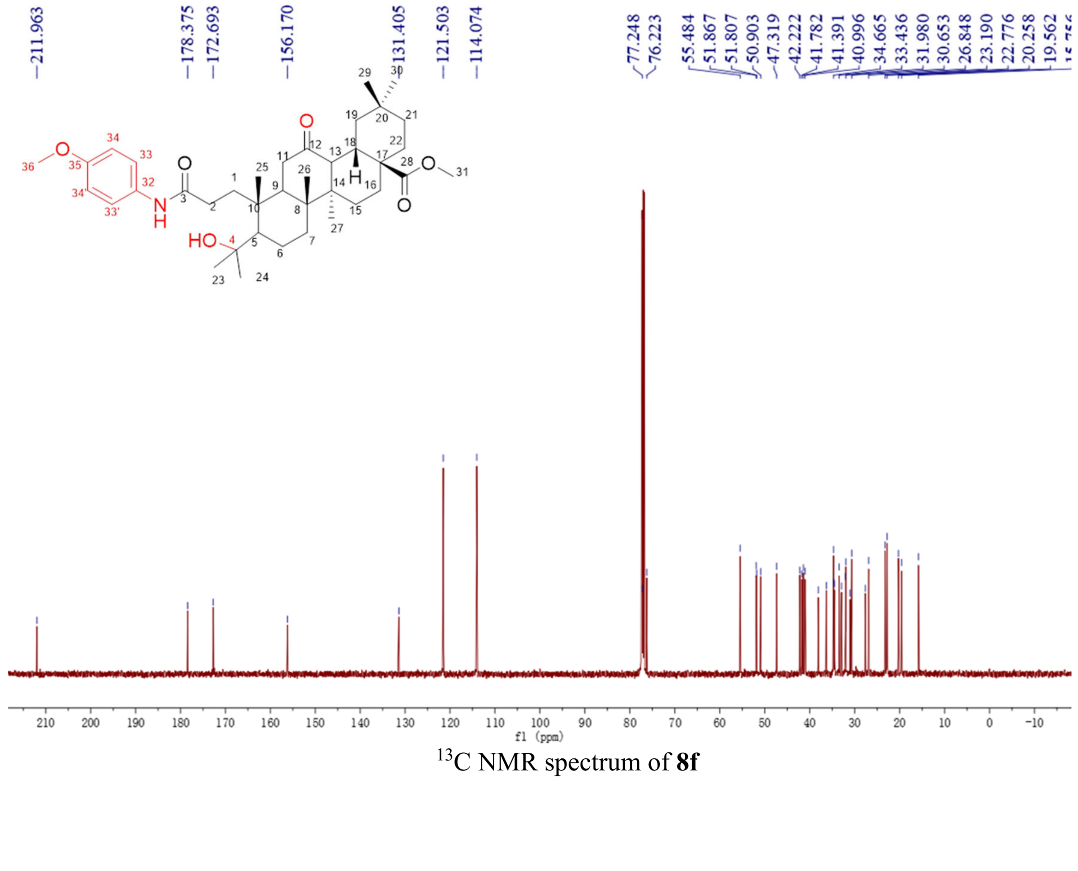

^{13}C NMR spectrum of **8f**

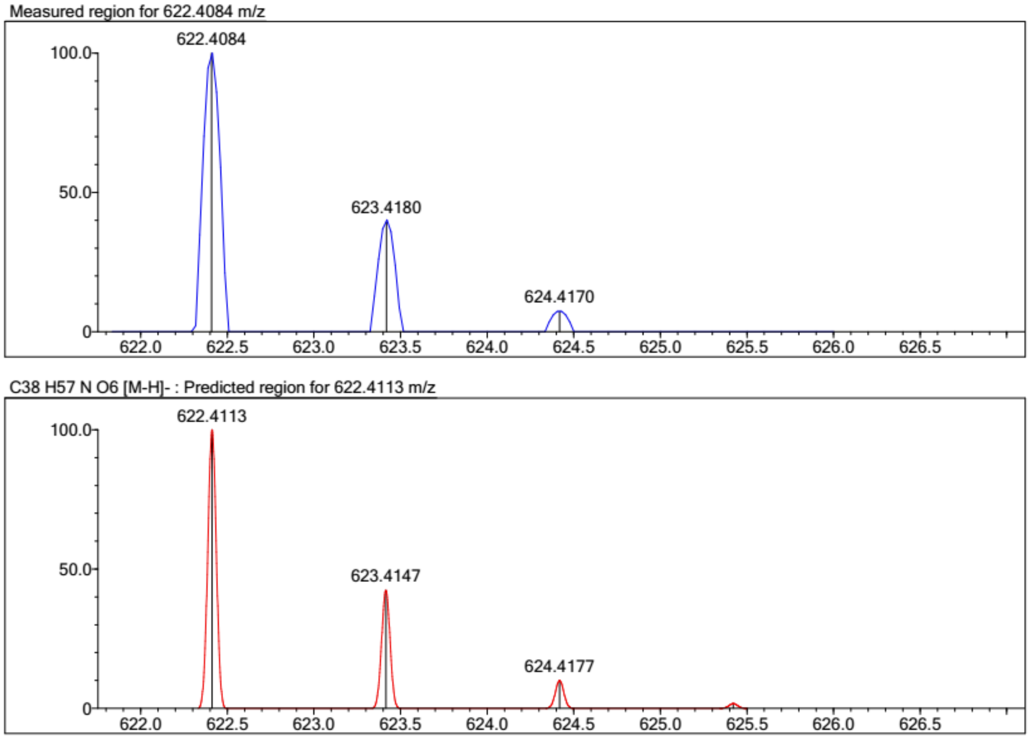

Rank	Score	Formula (M)	Ion	Meas. m/z	Pred. m/z	Df. (mDa)	Df. (ppm)	Iso	DBE
12	83.45	C38 H57 N O6	[M-H]-	622.4084	622.4113	-2.9	-4.66	91.85	11.0

HRMS spectrum of **8f**

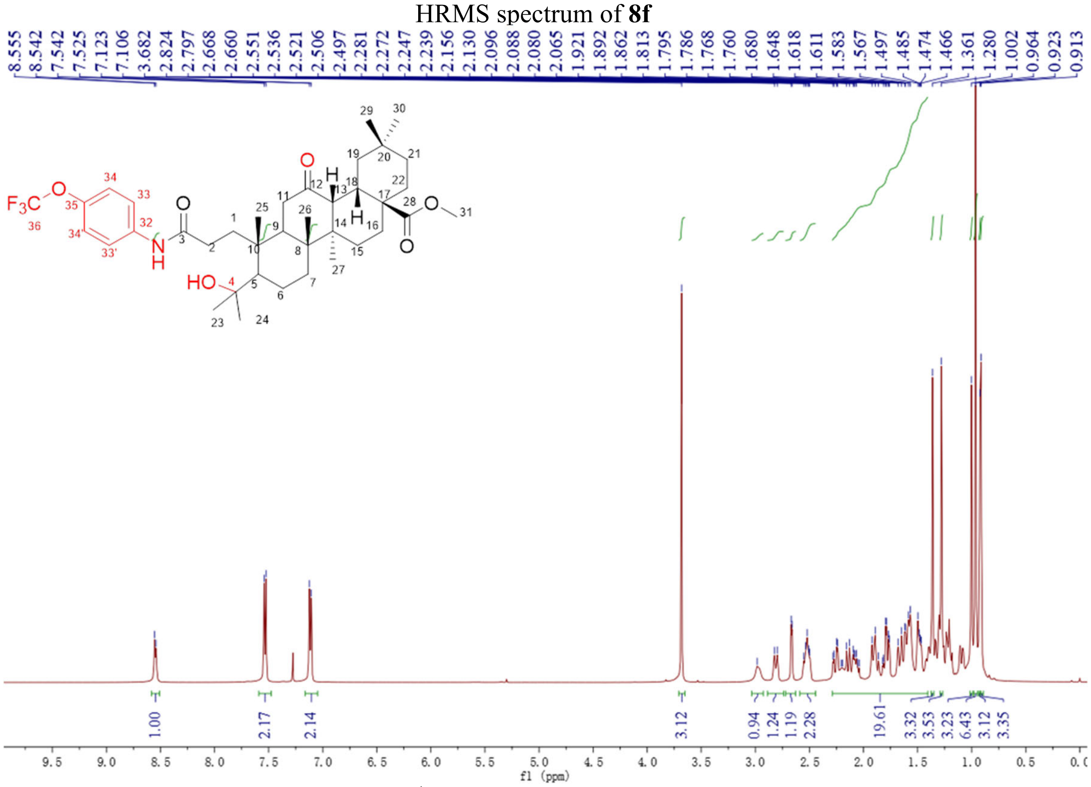

^{1}H NMR spectrum of **8g**

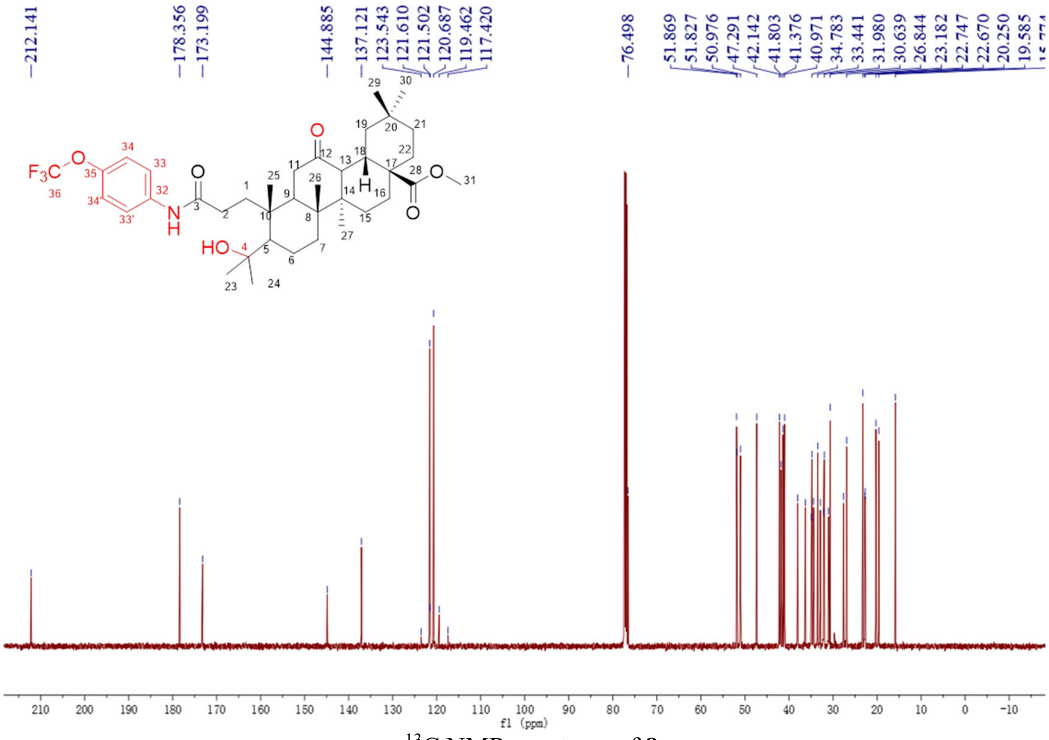

^{13}C NMR spectrum of **8g**

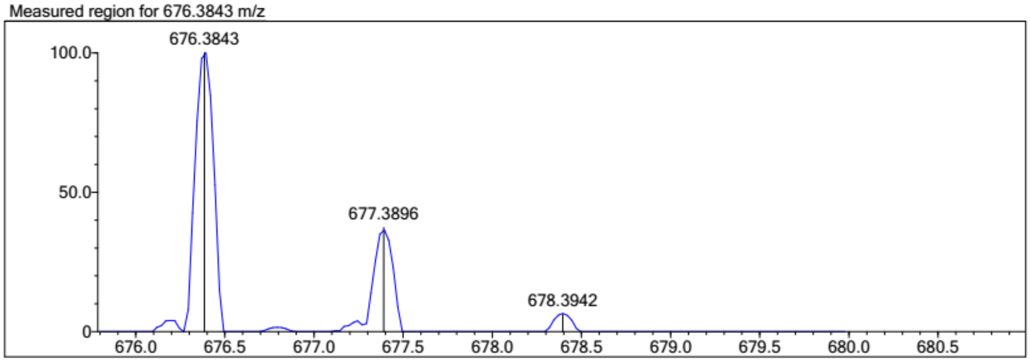

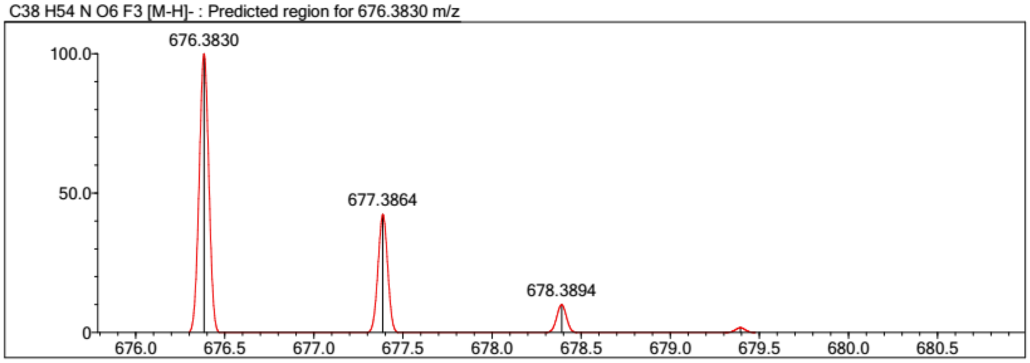

Rank	Score	Formula (M)	Ion	Meas. m/z	Pred. m/z	Df. (mDa)	Df. (ppm)	Iso	DBE
3	76.99	C38 H54 N O6 F3	[M-H]-	676.3843	676.3830	1.3	1.92	78.80	11.0

HRMS spectrum of **8g**

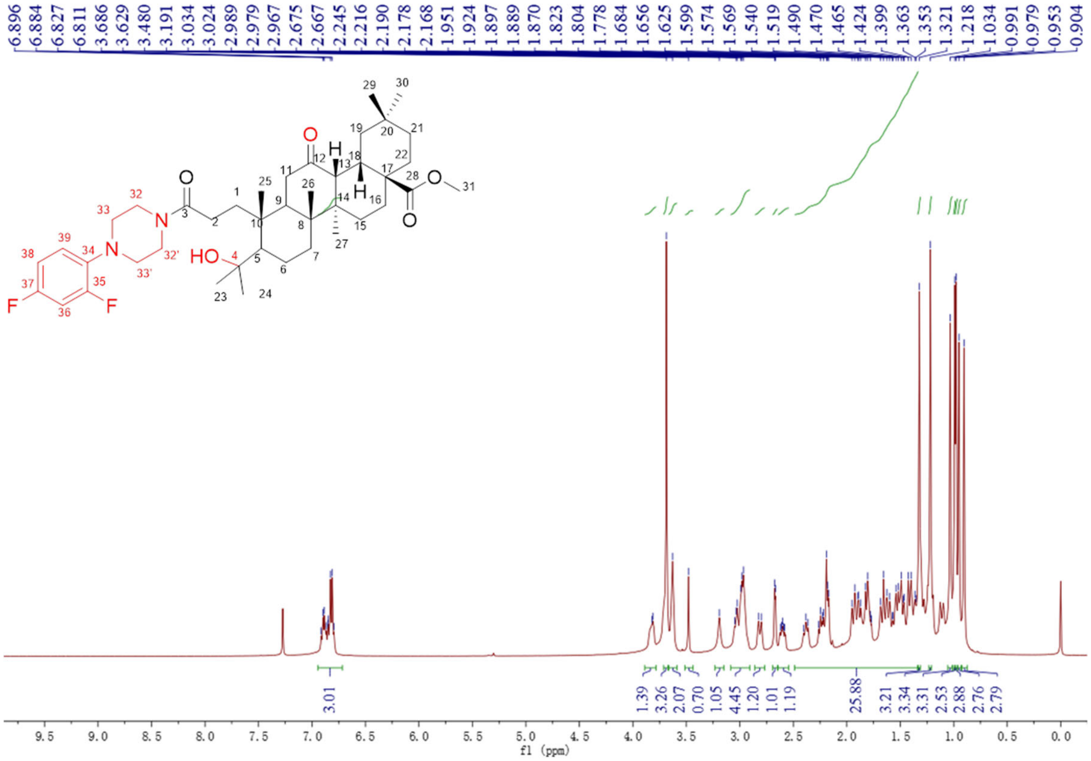

^{1}H spectrum of **8h**

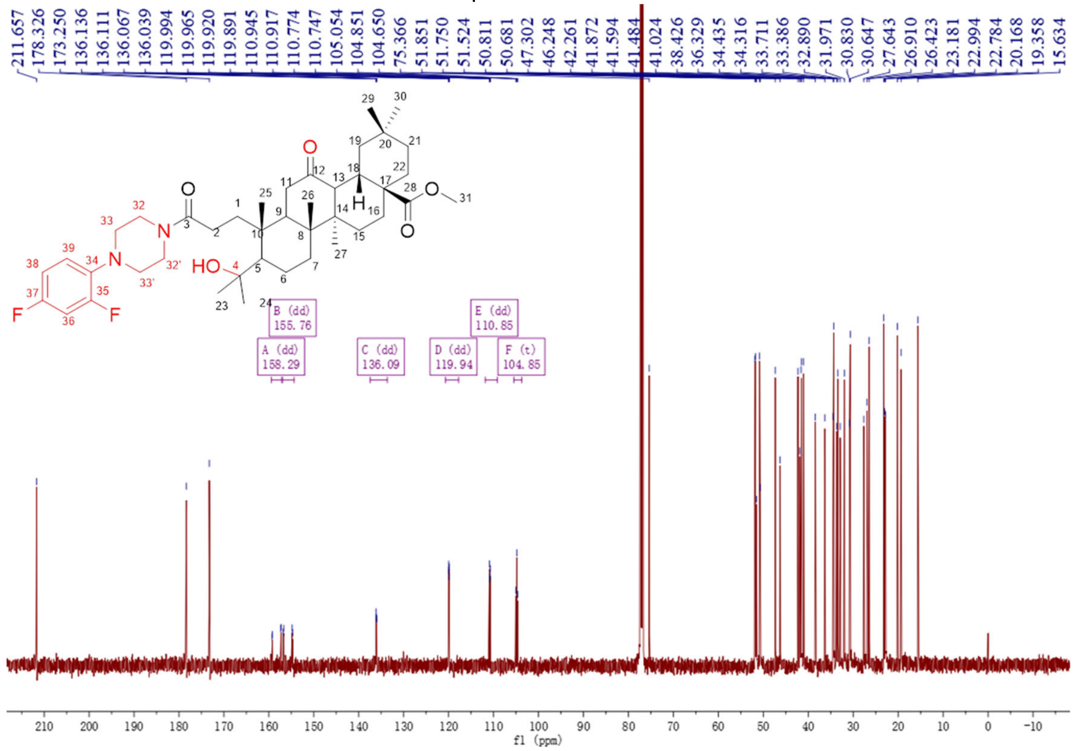

^{13}C spectrum of **8h**

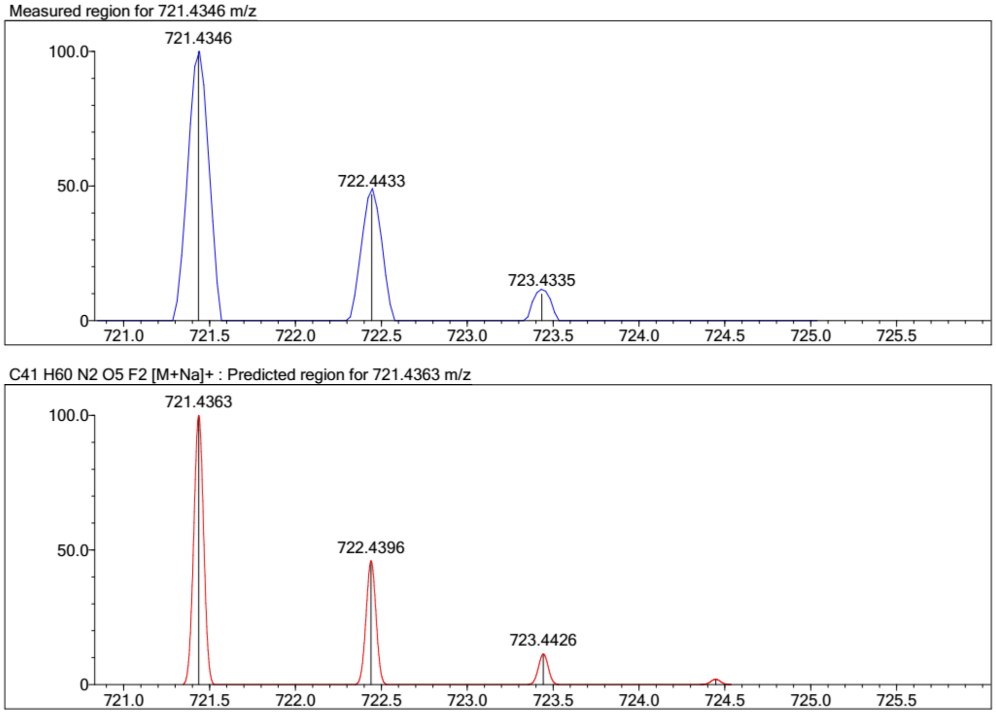

Rank	Score	Formula (M)	Ion	Meas. m/z	Pred. m/z	Df. (mDa)	Df. (ppm)	Iso	DBE
10	92.23	C41 H60 N2 O5 F2	[M+Na]+	721.4346	721.4363	-1.7	-2.36	95.48	12.0

HRMS spectrum of **8h**

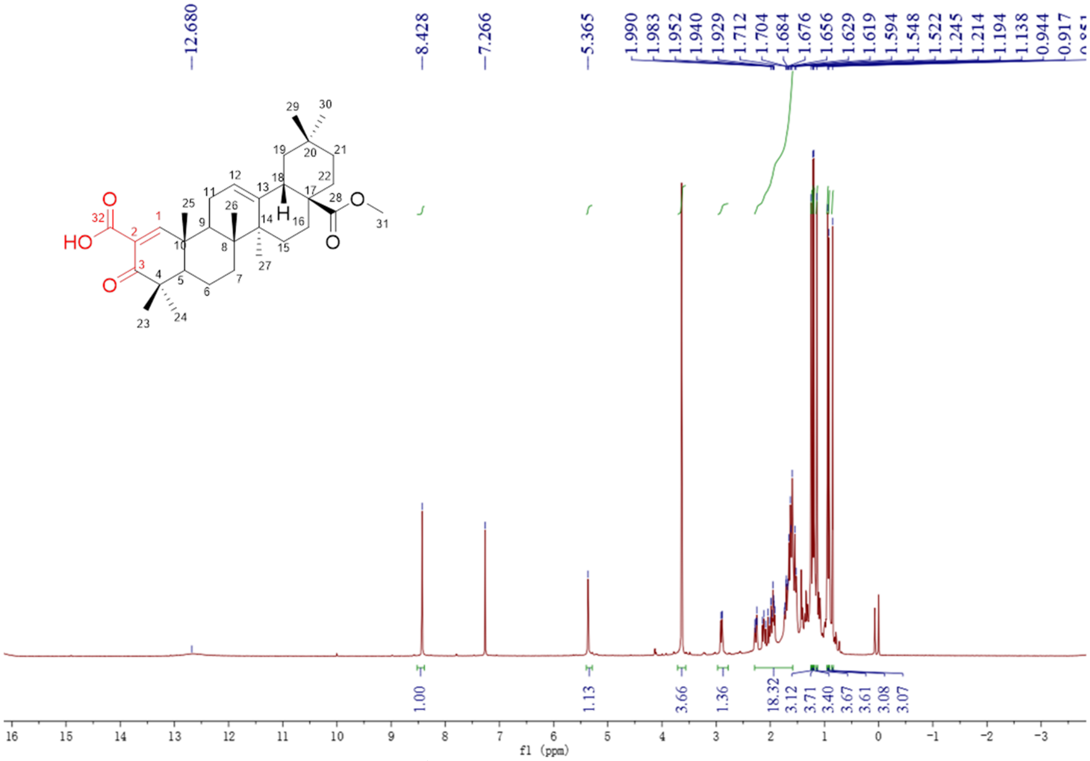

^{1}H NMR spectrum of **9**

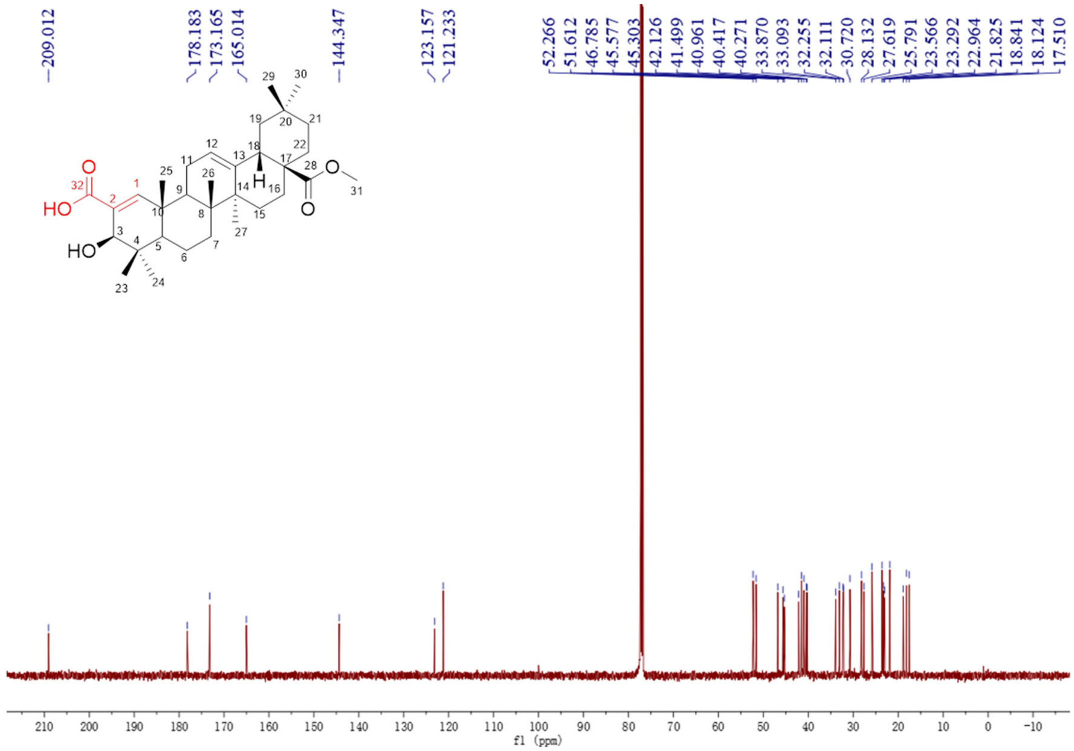

^{13}C spectrum of **9**

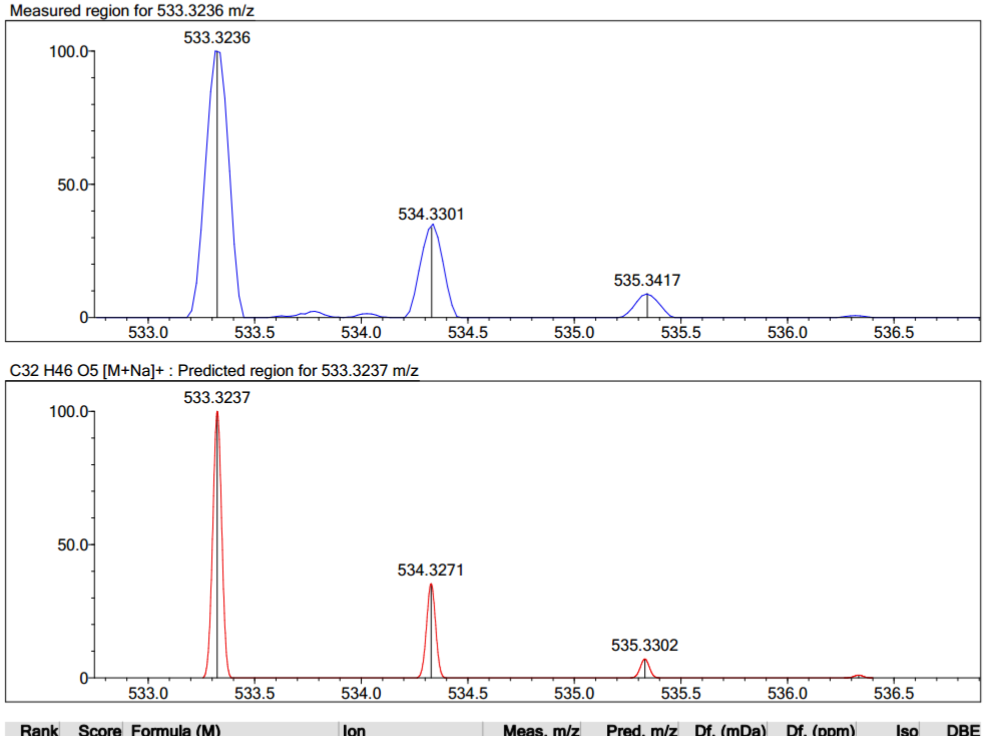

Rank	Score	Formula (M)	Ion	Meas. m/z	Pred. m/z	Df. (mDa)	Df. (ppm)	Iso	DBE
1	78.65	C32 H46 O5	[M+Na]+	533.3236	533.3237	-0.1	-0.19	78.65	10.0

HRMS spectrum of **9**

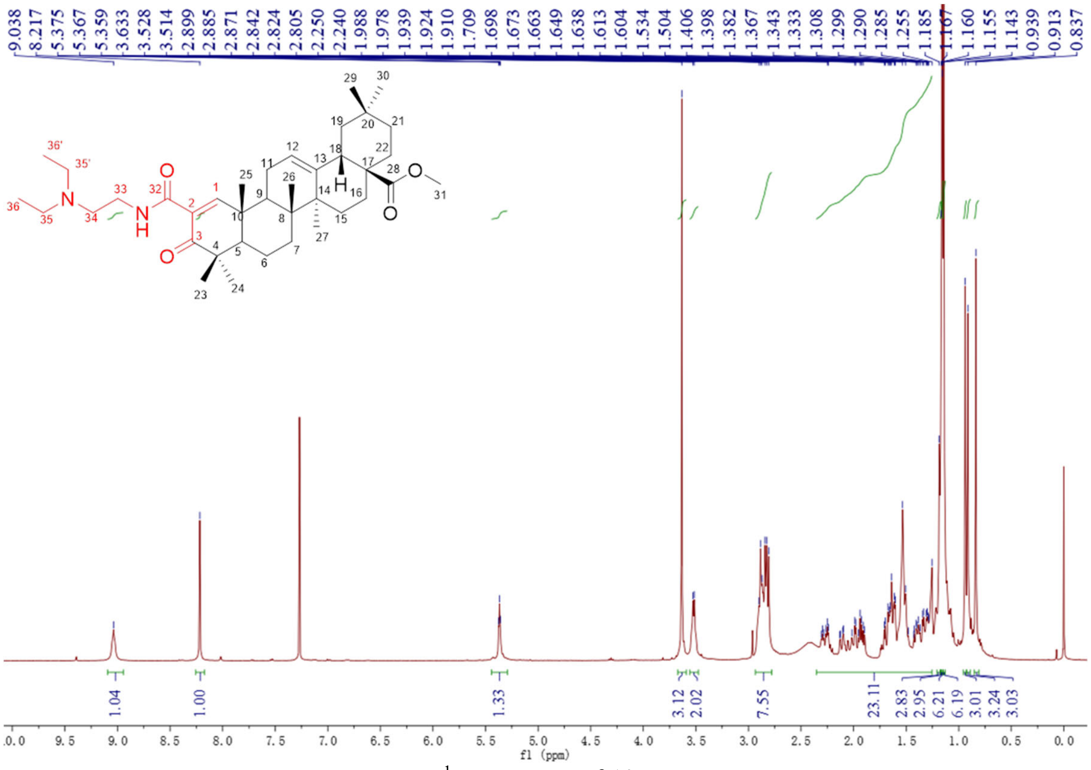

^{1}H spectrum of **10a**

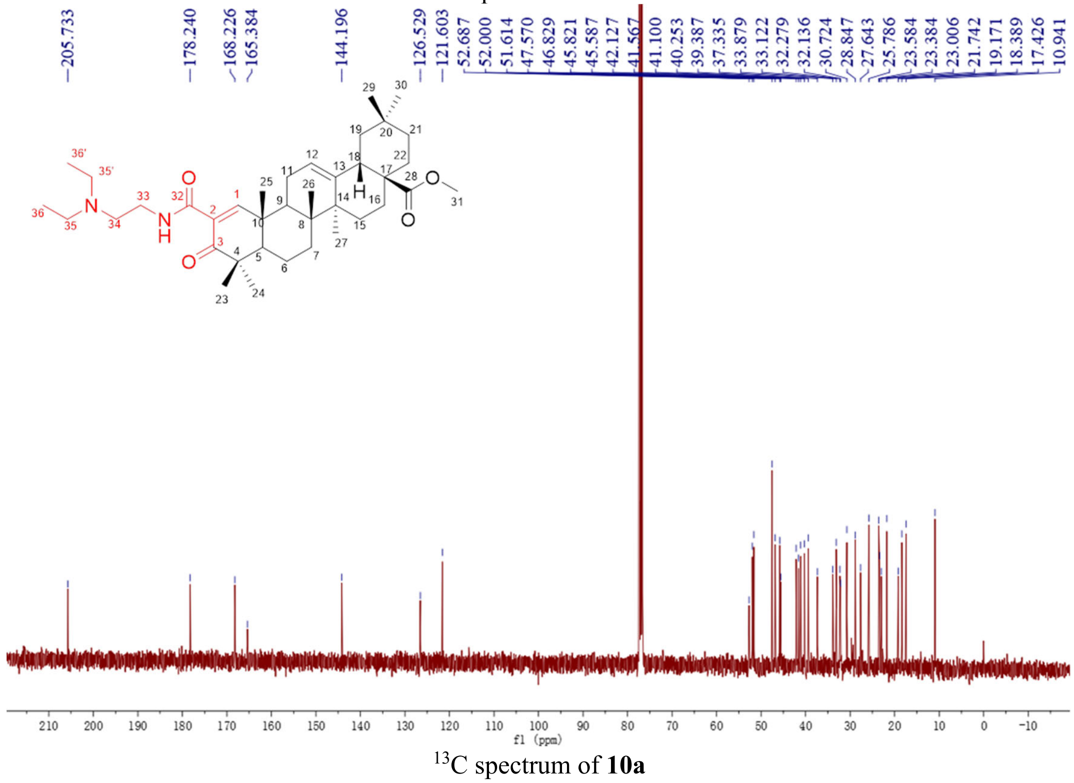

^{13}C spectrum of **10a**

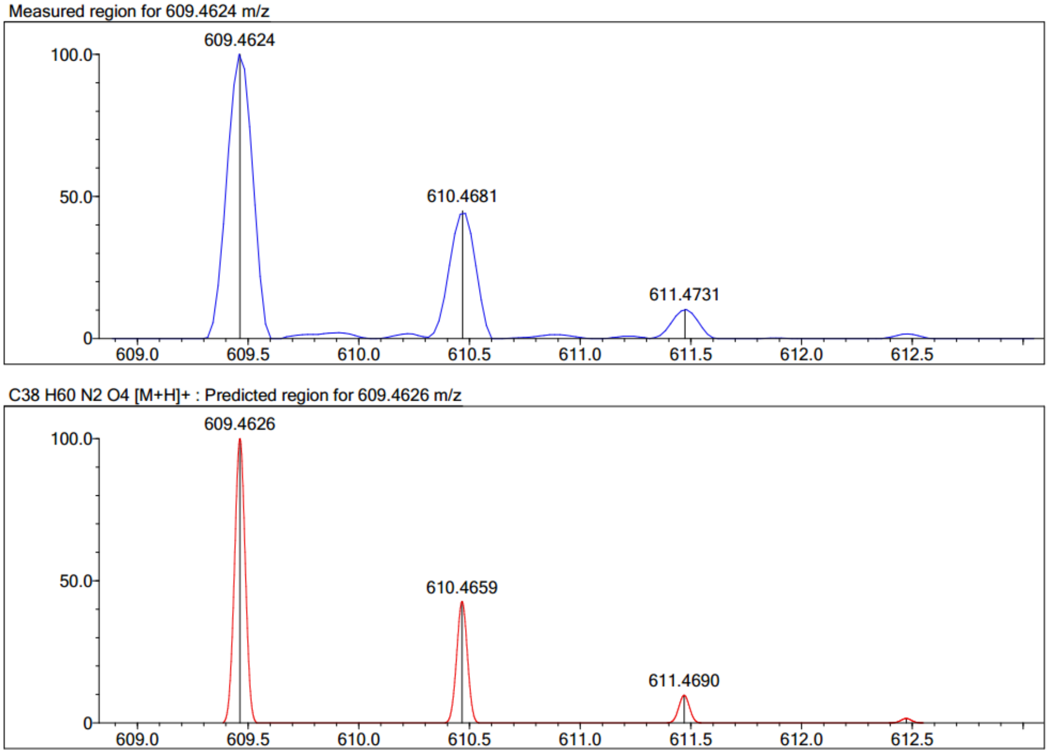

Rank	Score	Formula (M)	Ion	Meas. m/z	Pred. m/z	Df. (mDa)	Df. (ppm)	Iso	DBE
1	88.17	C38 H60 N2 O4	[M+H]+	609.4624	609.4626	-0.2	-0.33	88.17	10.0

HRMS spectrum of **10a**

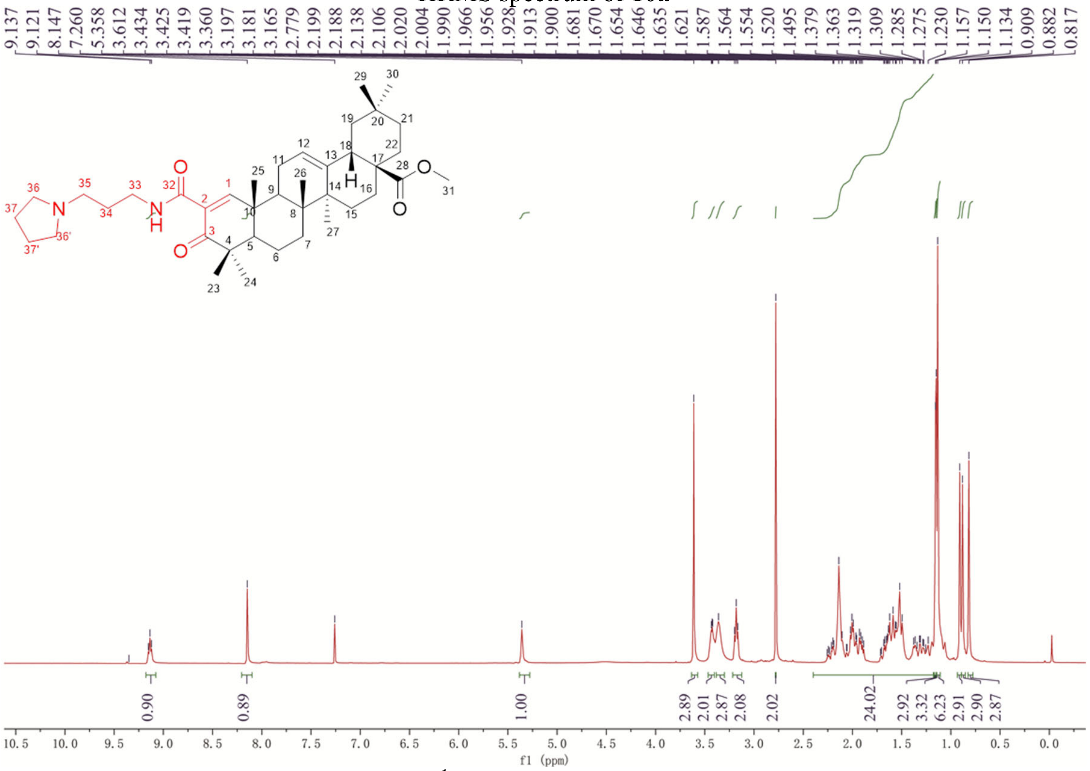

^{1}H spectrum of **10b**

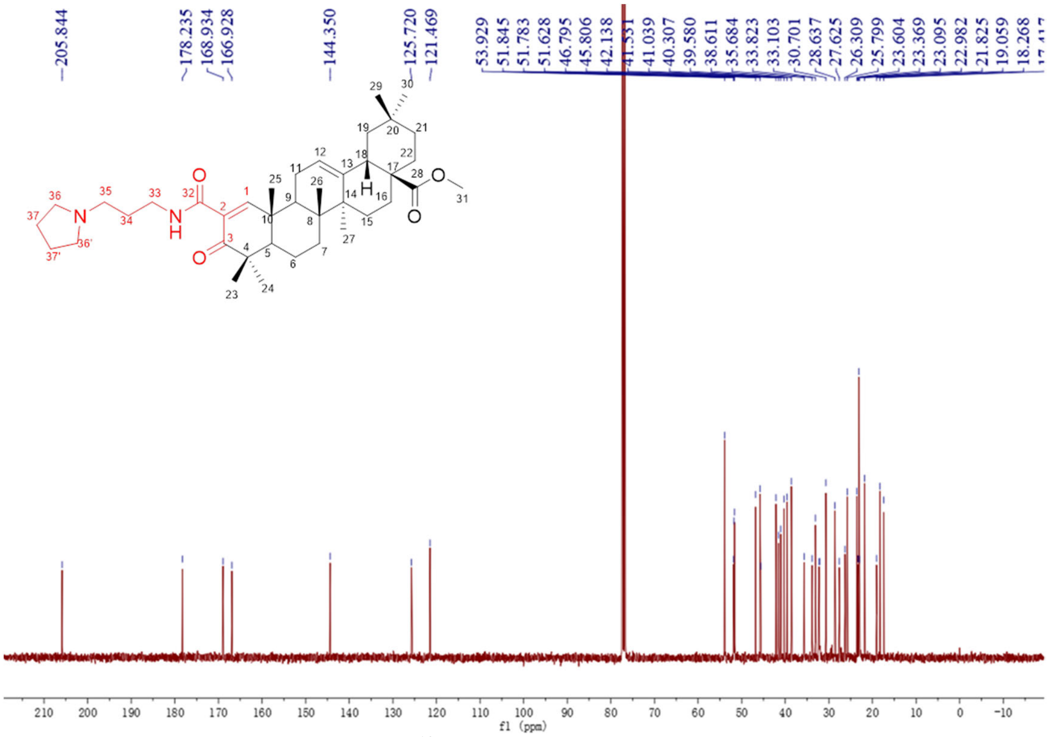

^{13}C spectrum of **10b**

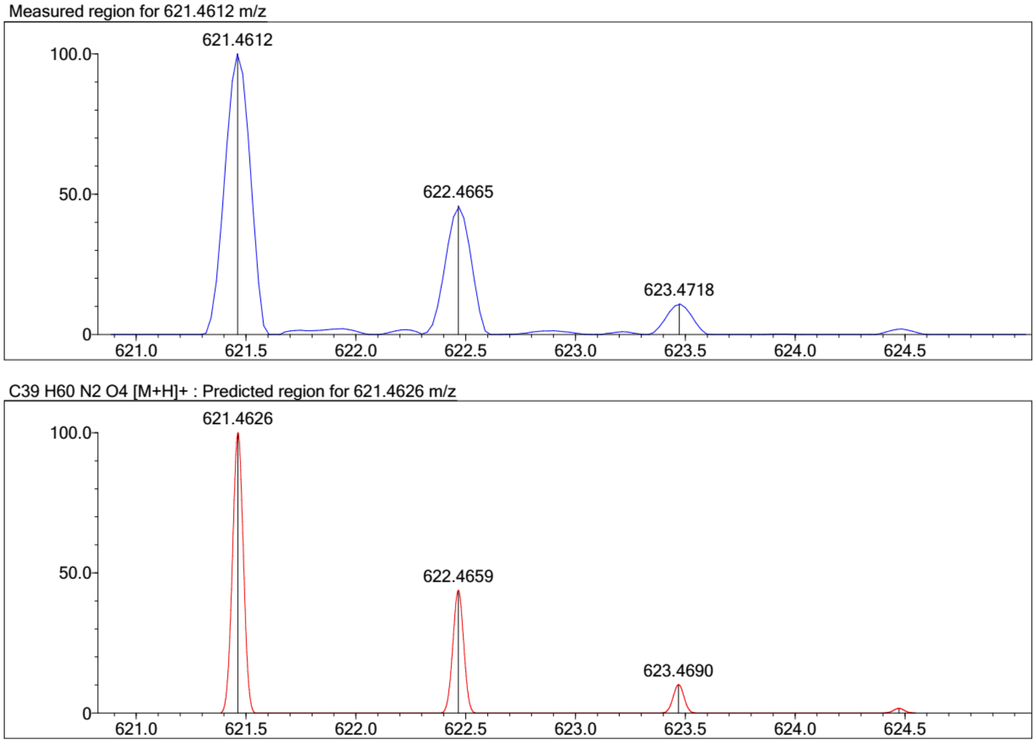

Rank	Score	Formula (M)	Ion	Meas. m/z	Pred. m/z	Df. (mDa)	Df. (ppm)	Iso	DBE
1	87.53	C39 H60 N2 O4	[M+H]+	621.4612	621.4626	-1.4	-2.25	90.36	11.0

HRMS spectrum of **10b**

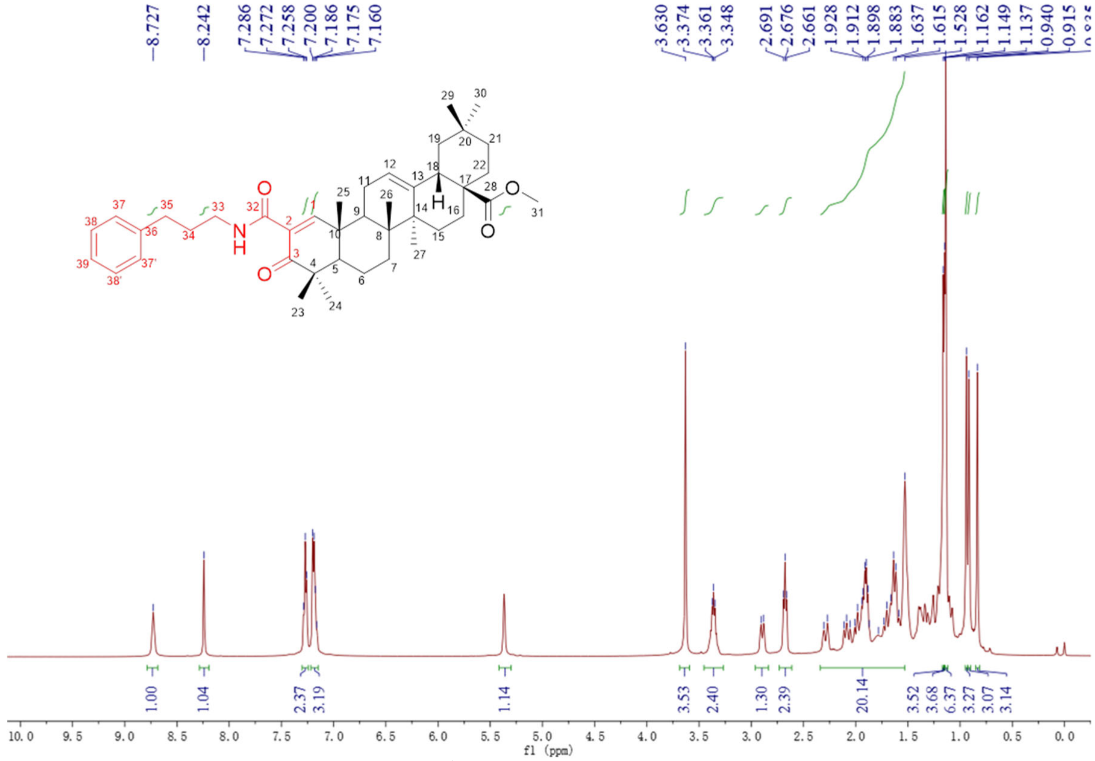

^{1}H spectrum of **10c**

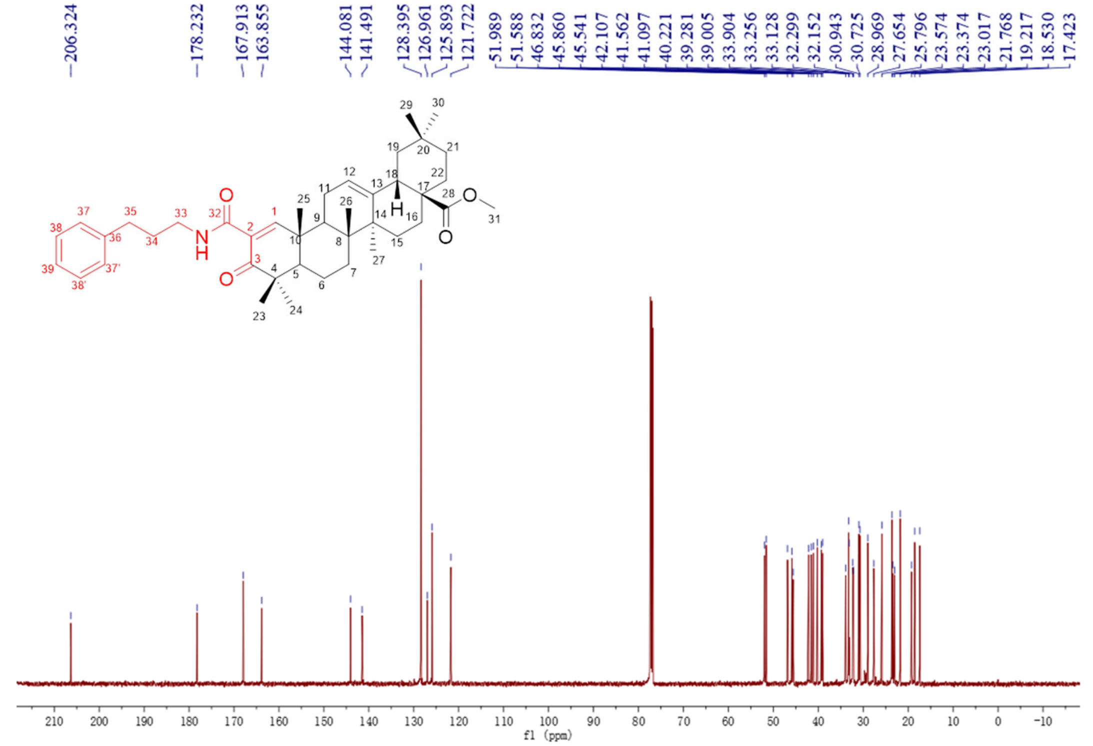

^{13}C spectrum of **10c**

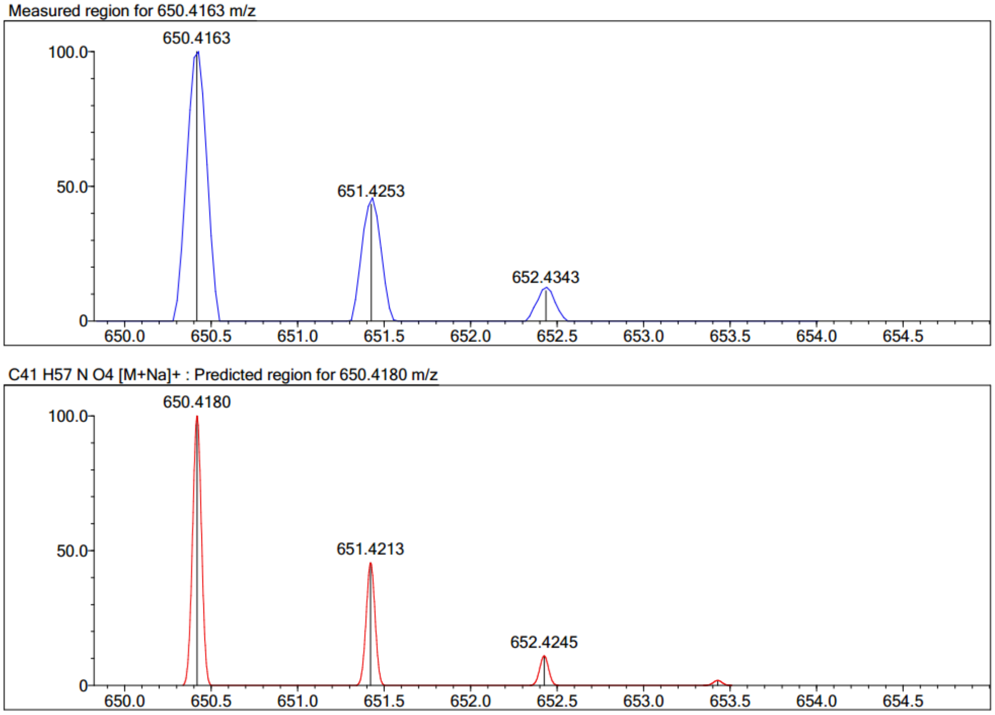

Rank	Score	Formula (M)	Ion	Meas. m/z	Pred. m/z	Df. (mDa)	Df. (ppm)	Iso	DBE
7	88.67	C41 H57 N O4	[M+Na]+	650.4163	650.4180	-1.7	-2.61	92.39	14.0

HRMS spectrum of **10c**

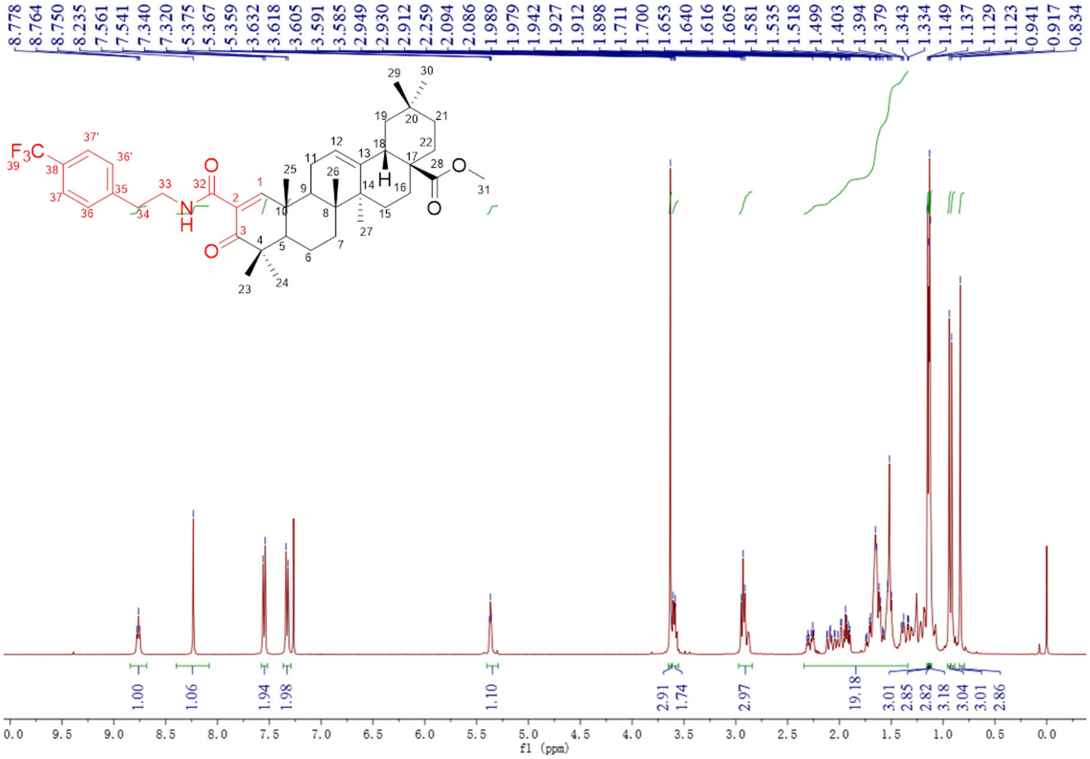

^{1}H spectrum of **10d**

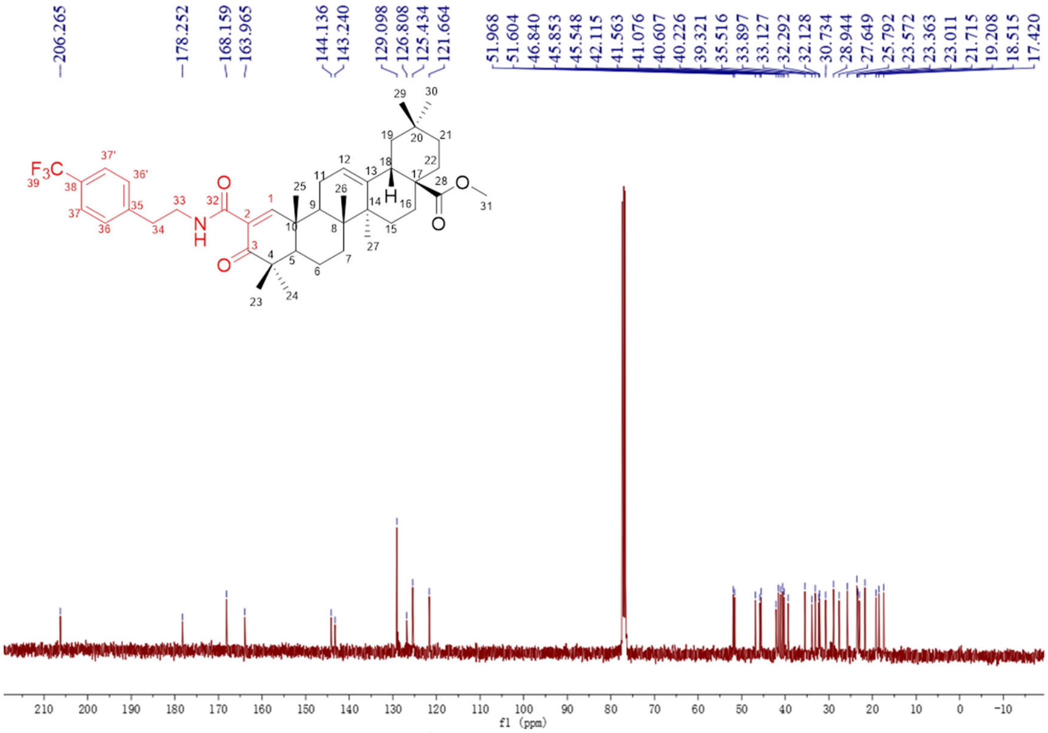

^{13}C spectrum of **10d**

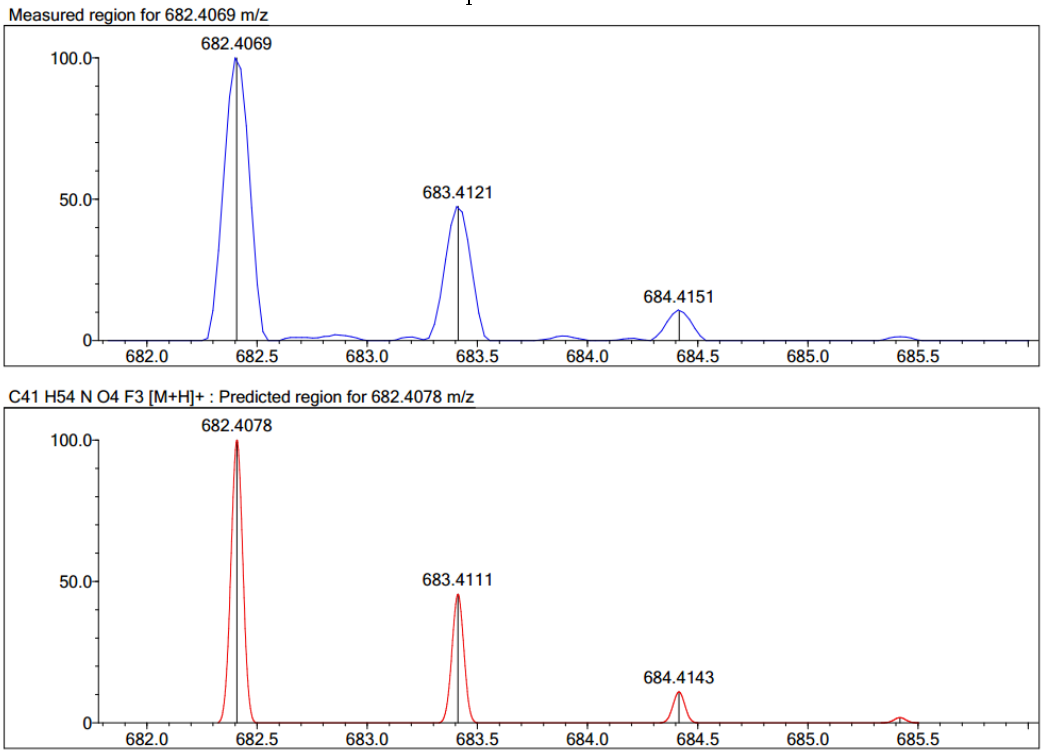

Rank	Score	Formula (M)	Ion	Meas. m/z	Pred. m/z	Df. (mDa)	Df. (ppm)	Iso	DBE
6	99.20	C41 H54 N O4 F3	[M+H]+	682.4069	682.4078	-0.9	-1.32	100.00	14.0

HRMS spectrum of **10d**
